# Systematic Optimization of Proteolysis-Targeting Chimeras for PIN1 Enables Selective Degradation and Antitumor Activity In Vivo

**DOI:** 10.3390/pharmaceutics18030288

**Published:** 2026-02-26

**Authors:** Yuying Ma, Yang Teng, Jinjin Liu, Yuke Deng, Lingbo Xu, Ruichen Gao, Tingyu Peng, Wei Li, Yue Wei, Linfeng Li, Zufeng Guo

**Affiliations:** 1 College of Pharmacy, Chongqing Medical University, Chongqing 400016, China; 2 Basic Medicine Research and Innovation Center for Novel Target and Therapeutic Intervention (Ministry of Education), Chongqing Medical University, Chongqing 400016, China; 3 Department of Medicinal Chemistry, School of Pharmacy, China Pharmaceutical University, Nanjing 211198, China

**Keywords:** PIN1, proteolysis-targeting chimeras, structure–activity relationship, antitumor efficacy

## Abstract

**Background**: The peptidyl–prolyl cis–trans isomerase PIN1 regulates multiple oncogenic and tumor-suppressive pathways and is frequently overexpressed in human cancers. Although pharmacological inhibition of PIN1 has shown antitumor potential, existing PIN1-targeting degraders lack systematic structure–activity relationship (SAR) analyses and display inconsistent cellular efficacy, leaving the therapeutic relevance of PIN1 degradation unclear. **Methods**: Two series of PIN1-targeting PROTACs were designed using the covalent inhibitor sulfopin as the PIN1 binder and ligands for either cereblon (CRBN) or von Hippel–Lindau (VHL). Systematic SAR studies focused on linker structure and jointing atom composition. PIN1 degradation was assessed by Western blotting in multiple cancer cell lines, and further investigated through a series of computational and mechanistic experiments. Antitumor efficacy and safety were evaluated in an MCF-7 xenograft mouse model with preliminary pharmacokinetic analysis. **Results**: SAR analysis revealed that short, linear linkers and reduced hydrogen bond donor content markedly enhanced PIN1 degradation, whereas VHL-recruiting PROTACs showed inferior cellular activity. These studies identified **PC2**, a CRBN-recruiting PROTAC, as a lead compound. **PC2** selectively induced ubiquitin–proteasome-dependent PIN1 degradation with minimal global proteomic or transcriptomic perturbation. Despite modest antiproliferative effects in vitro, **PC2** significantly suppressed tumor growth in vivo without observable toxicity and achieved effective intratumoral PIN1 degradation. **Conclusions**: This study defines SAR-guided design principles for PIN1-targeting PROTACs and demonstrates that selective PIN1 degradation can produce robust antitumor activity in vivo. **PC2** represents the first PIN1 degrader validated in animal models and supports targeted PIN1 degradation as a viable anticancer strategy.

## 1. Introduction

The human PIN1 protein is a peptidyl–prolyl cis–trans isomerase that specifically catalyzes the isomerization of phosphorylated serine/threonine-proline motifs, thereby modulating the stability and function of numerous proteins involved in cell cycle regulation, proliferation, and apoptosis [1]. Overexpression or aberrant activation of PIN1 is observed in various cancers (breast cancer, pancreatic adenocarcinoma, leukemia, etc.) and correlates with poorer prognosis [2,3,4,5]. By stabilizing oncogenic proteins (MYC, β-catenin, cyclin D1, etc.) and downregulating tumor suppressor proteins (SMAD, FBXW7, etc.), PIN1 supports tumor cell survival and promotes treatment resistance by maintaining cancer stem cell populations. PIN1 can also drive immunosuppressive tumor microenvironment [6]. In addition, mice with *PIN1* knock-out typically exhibited limited phenotypic defects [7]. As such, targeting PIN1 offers an opportunity to disrupt critical cancer pathways with minimal safety concerns, positioning it as a promising approach for developing novel anticancer treatments.

**Figure 1:**
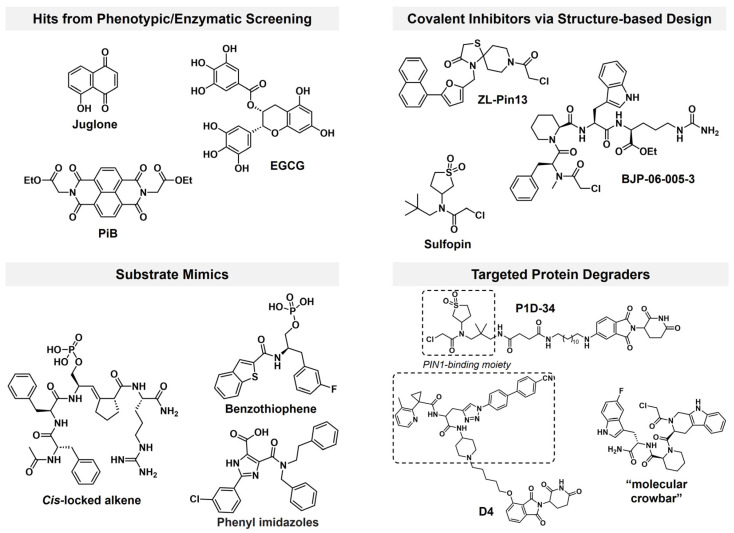
Representative PIN1 inhibitors and degraders.

Discovery of PIN1 inhibitor has progressed through various strategies (Figure 1) [8,9]. In the early time, phenotypic or enzymatic screens identified compounds such as Juglone [10], PiB [11], and EGCG [12], which engaged PIN1 but lacked specificity due to the promiscuity of their polyphenolic or polycyclic aromatic scaffolds, resulting in mediocre potency and potential off-target effects. Substrate-mimicking molecules incorporating acidic groups to emulate phospho-serine/threonine motifs [13,14,15] offered improved selectivity but were hindered by poor membrane permeability and limited cellular activity. The emergence of targeted covalent inhibitors [16,17] in recent decade facilitated mechanism-based rational design to target PIN1 by specifically reacting with Cys113 in its active site [18,19,20,21]. Among them, sulfopin is deemed a landmark in PIN1 covalent inhibitor development that potently inhibited PIN1’s enzymatic activity (*K*_i_^app^ = 0.211 μM) and demonstrated considerable anticancer efficacy in several in vivo models albeit at relatively high doses [19]. Despite encouraging progress, most investigational PIN1 inhibitors remain in preclinical development, underscoring the ongoing need to identify more efficacious modulators of PIN1.

Targeted degradation of PIN1 presents a compelling alternative to conventional inhibition (Figure 1), as it more accurately recapitulates the phenotypic effects of genetic knock-out by achieving ablation of PIN1 rather than solely blocking its enzymatic function [22]. While our study was in progress, Shi et al. reported **P1D-34** [23], the first PROTAC targeting PIN1. Shortly thereafter, Liu et al. developed **D4** [24], a PROTAC based on a neutral inhibitor identified through a DEL screen campaign which possessed slightly improved potency (IC_50_ = 0.15 μM) over sulfopin. Interestingly, these two studies reached contrasting conclusions about the feasibility of PIN1 degradation as an antitumor strategy: while **P1D-34**-induced degradation (DC_50_ = 0.18 μM, D_max_ = 95%) correlates reasonably well with its antiproliferative effect in cancer cells (IC_50_ = 2.2 μM), **D4**’s potent degradative capability (DC_50_ = 0.018 μM, D_max_ = 93%) fails to translate into antiproliferative activity (GI_50_ > 30 μM) in multiple cell lines. Recently, another team introduced monovalent “molecular crowbars” to degrade PIN1 [25]; however, these compounds are primarily characterized biophysically and biochemically, with their therapeutic potential yet to be explored. In brief, none of the aforementioned degraders have been tested in vivo, leaving critical questions about the causal relationship between PIN1 degradation and antitumor efficacy unanswered. Moreover, studies on PIN1 PROTACs have largely overlooked the structure–activity relationship (SAR), particularly the crucial role of linker design in determining the efficacy of these degraders.

To address these challenges, we leveraged insights from our linker-focused SAR study to refine design strategies for PIN1-targeting PROTACs, eventually leading to the identification of **PC2** as a lead compound. A series of mechanistic experiments demonstrated that **PC2** effectively and selectively degraded PIN1 via the ubiquitin–proteasome system (UPS) while causing minimal disruption to cellular homeostasis. Remarkably, despite its modest impact on cancer cell viability, **PC2** elicited a significant antitumor effect and demonstrated a favorable safety profile in a breast cancer mouse model. These findings provided key insights into the therapeutic potential of PIN1 degradation, which could help clarify ongoing debates in the field. Furthermore, preliminary pharmacokinetic studies provided valuable insights to guide the further optimization of **PC2** toward a preclinical candidate.

## 2. Materials and Methods

### 2.1. Chemistry General Information

Reagents and solvents were obtained from commercial suppliers and were used directly without further purification. Analytical thin layer chromatography (TLC) was carried out using precoated silica gel GF-254 plates, and was analyzed using UV light or potassium permanganate stain. Products were purified by flash column chromatography, which was performed using silica gel (230–400 mesh). In most cases, final compounds were purified using a reversed phase HPLC system (Agilent 1260II Prime, ZORBAX Extend-C18, 21.2 mm × 150 mm, Santa Clara, CA, USA). ^1^H NMR and ^13^C NMR spectra were recorded in CDCl_3_, CD_3_OD, or (CD_3_)_2_SO on a Bruker Avance III HD 600 MHz NMR-spectrometer (Billerica, MA, USA) operating at 600 MHz (^1^H), 150 MHz (^13^C), or on a JOEL 400 MHz NMR-spectrometer (Akishima, Tokyo, Japan) operating at 400 MHz (^1^H) and 100 MHz (^13^C). Chemical shifts (δ) are reported in ppm and are referenced to the chemical shift in the residual solvent proton(s). Data are reported as follows: chemical shift, multiplicity (s = singlet, d = doublet, t = triplet, q = quartet, p = quintet, m = multiplet, br = broad), coupling constants (Hz), and integration. The purity of compounds was assessed by analytical HPLC using a Shimadzu LC-2030 Plus system (Nakagyo-ku, Kyoto, Japan) equipped with a Waters SunFire C18 column (5 μm, 4.6 × 150 mm, Milford, MA, USA), and the purity was >95% for all tested compounds.

### 2.2. Compound Synthesis

Synthetic procedures and structural characterizations for the final products and representative intermediates (**5a–j**, **10a–b**, **13a–b**) are described below, while the remaining intermediates (**3a–e**, **4a–e**, **6a**, **7a**, **8a–b**, **9a–b**, **11a**, **12a**, **15a–g**, **16a–g**, **18a–h**, and **19a–h**) are detailed in the Supplementary Information. The control compounds, sulfopin and **P1D-34**, were purchased from MedChemExpress (HY-139361, HY 171334A). It should be noted that PC and PV compounds were generally synthesized and purified as diastereomeric mixtures, which were then subjected to biological testing without further separation.

**General Procedure 1: Ester coupling reaction.** To a solution of acid (1.0 equiv) and alcohol (1.0 equiv) in anhydrous DCM, DCC (1.5 equiv), DMAP (0.1 equiv), and TEA (3.0 equiv) were added. The reaction mixture was stirred overnight at room temperature (RT). After completion, the mixture was washed sequentially with 1.0 M HCl, aqueous NaHCO_3_, brine, and then dried over Na_2_SO_4_. The organic phase was concentrated, and the residue was purified by column chromatography.

**General Procedure 2: Amide coupling reaction.** To a solution of acid (1.0 equiv) in anhydrous DMF (5 mL), amine (1.0 equiv), HATU (1.5 equiv), and DIPEA (3.0 equiv) were added at 0 °C. The reaction mixture was allowed to warm to RT and stirred overnight. After completion, the mixture was extracted with DCM and washed sequentially with water, 1 M HCl, aqueous NaHCO_3_, and brine. The organic layer was then dried over Na_2_SO_4_. The solvent was removed under reduced pressure, and the residue was purified by column chromatography.

**General Procedure 3: Synthesizing ureido compound.** To a solution of amine (1.0 equiv) in anhydrous DCM, DIPEA (9.0 equiv) and BTC (0.3 equiv) were added. The reaction mixture was stirred at RT for 2 h. Once the starting material was completely consumed, (*S*, *R*, *S*)-AHPC-Me hydrochloride (1.0 equiv) was added, and the mixture was stirred overnight at room temperature. After completion, the reaction mixture was extracted with DCM and washed sequentially with water, aqueous NH_4_Cl, aqueous NaHCO_3_, and brine. The organic layer was dried over Na_2_SO_4_, concentrated under reduced pressure, and the residue was purified by column chromatography.

**General Procedure 4: Warhead incorporation.** To a solution of the corresponding intermediate (1.0 equiv) in anhydrous DCM (10 mL), DIPEA (3.0 equiv) was added. A solution of 2-chloroacetyl chloride (1.5 equiv) in anhydrous DCM (1 mL) was added dropwise at 0 °C. The reaction mixture was allowed to warm to room temperature and stirred for 2 h. After completion, the DCM layer was washed sequentially with 1.0 M HCl, aqueous NaHCO_3_, and brine, then dried over Na_2_SO_4_. The organic layer was concentrated, and the residue was purified by column chromatography.

3-((1,1-dioxidotetrahydrothiophen-3-yl)amino)-2,2-dimethylpropyl 6-((2-(2,6-dioxopiperidin-3-yl)-1,3-dioxoisoindolin-4-yl)oxy)hexanoate (**5a**). **5a** (61% yield, white solid) was synthesized according to **General Procedure 1**. ^1^H NMR (600 MHz, Chloroform-*d*) δ 7.67 (dd, *J* = 8.5, 7.3 Hz, 1H), 7.45 (d, *J* = 7.3 Hz, 1H), 7.22 (d, *J* = 8.5 Hz, 1H), 4.97 (dd, *J* = 12.3, 5.3 Hz, 1H), 4.18 (t, *J* = 6.3 Hz, 2H), 3.88 (d, *J* = 1.4 Hz, 2H), 3.57–3.49 (m, 1H), 3.32–3.24 (m, 2H), 3.03 (dtd, *J* = 13.1, 7.7, 2.5 Hz, 1H), 2.90–2.71 (m, 4H), 2.42–2.36 (m, 5H), 2.16–2.08 (m, 1H), 2.08–2.02 (m, 1H), 1.91 (p, *J* = 6.6 Hz, 2H), 1.73 (p, *J* = 7.5 Hz, 2H), 1.62–1.54 (m, 3H), 0.91 (s, 6H). ^13^C NMR (151 MHz, Chloroform-*d*) δ 173.59, 171.14, 168.30, 167.07, 165.69, 156.59, 136.55, 133.82, 118.97, 117.15, 115.83, 69.99, 69.08, 57.09, 55.74, 54.79, 50.59, 50.58, 49.10, 35.00, 34.13, 31.40, 29.56, 28.63, 25.48, 24.62, 22.92, 22.64. LC-MS (*m*/*z*): positive mode 592.2 [M + H]^+^.

3-(2-chloro-*N*-(1,1-dioxidotetrahydrothiophen-3-yl)acetamido)-2,2-dimethylpropyl 6-((2-(2,6-dioxopiperidin-3-yl)-1,3-dioxoisoindolin-4-yl)oxy)hexanoate (**PC1**). **PC1** (78% yield, white solid) was synthesized from **5a** according to **General Procedure 4**. ^1^H NMR (600 MHz, Chloroform-*d*) δ 8.43 (s, 1H), 7.67 (dd, *J* = 8.5, 7.3 Hz, 1H), 7.45 (d, *J* = 7.3 Hz, 1H), 7.22 (d, *J* = 8.5 Hz, 1H), 4.97 (dd, *J* = 12.3, 5.3 Hz, 1H), 4.18 (t, *J* = 6.3 Hz, 2H), 3.88 (d, *J* = 1.4 Hz, 2H), 3.57–3.49 (m, 1H), 3.32–3.24 (m, 2H), 3.03 (dtd, *J* = 13.1, 7.7, 2.5 Hz, 1H), 2.91–2.72 (m, 4H), 2.43–2.35 (m, 5H), 2.16–2.08 (m, 1H), 2.08–2.02 (m, 1H), 1.91 (p, *J* = 6.6 Hz, 2H), 1.73 (p, *J* = 7.5 Hz, 2H), 1.61–1.54 (m, 3H), 0.91 (s, 6H). ^13^C NMR (151 MHz, Chloroform-*d*) δ 173.02, 171.05, 168.30, 168.00, 167.05, 165.72, 156.57, 136.59, 133.81, 118.94, 117.14, 115.88, 69.45, 69.01, 57.97, 57.52, 50.88, 50.25, 49.11, 48.92, 41.82, 36.62, 34.05, 31.40, 28.61, 26.50, 25.51, 24.51, 23.26, 22.65. HRMS (ESI) *m*/*z*: calcd for C_30_H_38_ClN_3_O_10_S, [M + Na], 690.1864; found, 690.1868.

3-((1,1-dioxidotetrahydrothiophen-3-yl)amino)-2,2-dimethylpropyl 4-((2-(2,6-dioxopiperidin-3-yl)-1,3-dioxoisoindolin-4-yl)oxy) butanoate (**5b**). **5b** (22% yield, white solid) was synthesized according to **General Procedure 1**. ^1^H NMR (400 MHz, Chloroform-*d*) δ 9.00 (s, 1H), 7.63 (t, *J* = 7.9 Hz, 1H), 7.39 (d, *J* = 7.3 Hz, 1H), 7.19 (d, *J* = 8.6 Hz, 1H), 5.01–4.84 (m, 1H), 4.20 (q, *J* = 8.3 Hz, 2H), 3.83 (q, *J* = 4.6 Hz, 2H), 3.46 (p, *J* = 6.5 Hz, 1H), 3.22 (tt, *J* = 16.2, 7.6 Hz, 2H), 2.98 (q, *J* = 6.8 Hz, 1H), 2.77–2.70 (m, 2H), 2.59 (t, *J* = 7.4 Hz, 2H), 2.33–2.27 (m, 1H), 2.22–1.93 (m, 5H), 1.74–1.48 (m, 1H), 1.37–0.93 (m, 3H), 0.87–0.81 (m, 6H). LC-MS (*m*/*z*): positive mode 564.2 [M + H]^+^.

3-(2-chloro-N-(1,1-dioxidotetrahydrothiophen-3-yl)acetamido)-2,2-dimethylpropyl 4-((2-(2,6-dioxopiperidin-3-yl)-1,3-dioxoisoindolin-4-yl)oxy)butanoate (**PC2**). **PC2** (54% yield, white solid) was synthesized from **5b** according to **General Procedure 4**. ^1^H NMR (600 MHz, Chloroform-*d*) δ 8.74 (d, *J* = 12.9 Hz, 1H), 7.68–7.64 (m, 1H), 7.44 (d, *J* = 7.3 Hz, 1H), 7.21 (d, *J* = 8.5 Hz, 1H), 5.01–4.92 (m, 1H), 4.23 (t, *J* = 5.2 Hz, 2H), 4.11 (s, 1H), 3.86 (qd, *J* = 11.5, 4.0 Hz, 3H), 3.71–3.61 (m, 2H), 3.36 (dd, *J* = 15.8, 4.7 Hz, 1H), 3.25 (d, *J* = 15.8 Hz, 1H), 3.17–3.09 (m, 1H), 3.03–2.97 (m, 1H), 2.84 (dd, *J* = 12.5, 2.8 Hz, 1H), 2.79–2.73 (m, 2H), 2.67 (d, *J* = 7.4 Hz, 2H), 2.50–2.44 (m, 2H), 2.19 (p, *J* = 6.5 Hz, 2H), 2.10 (ddd, *J* = 7.9, 6.3, 3.7 Hz, 1H), 1.88 (s, 1H), 0.98 (t, *J* = 6.2 Hz, 6H). ^13^C NMR (151 MHz, Chloroform-*d*) δ 172.71, 171.49, 168.71, 168.19, 167.08, 165.79, 156.28, 136.72, 133.85, 119.09, 117.34, 116.18, 69.82, 67.81, 57.96, 57.56, 50.41, 49.20, 42.07, 36.64, 34.00, 31.45, 30.17, 26.56, 25.02, 24.15, 23.28, 22.69. HRMS (ESI) *m*/*z*: calcd for C_28_H_34_ClN_3_O_10_S, [M + Na], 662.1551; found, 662.1555.

3-((1,1-dioxidotetrahydrothiophen-3-yl)amino)-2,2-dimethylpropyl 3-(2-((2-(2,6-dioxopiperidin-3-yl)-1,3-dioxoisoindolin-4-yl)oxy)ethoxy) propanoate (**5c**). **5c** (21% yield, white solid) was synthesized according to **General Procedure 1**. ^1^H NMR (600 MHz, Chloroform-*d*) δ 8.35 (s, 1H), 7.67 (dd, *J* = 8.5, 7.3 Hz, 1H), 7.47 (d, *J* = 7.3 Hz, 1H), 7.26 (d, *J* = 8.4 Hz, 1H), 4.96 (dd, *J* = 12.2, 5.3 Hz, 1H), 4.33 (t, *J* = 4.7 Hz, 2H), 3.95–3.85 (m, 6H), 3.49 (qd, *J* = 6.6, 4.1 Hz, 1H), 3.31–3.21 (m, 2H), 3.01 (dtd, *J* = 13.1, 7.8, 2.5 Hz, 1H), 2.91–2.70 (m, 4H), 2.61 (t, *J* = 6.2 Hz, 2H), 2.41–2.33 (m, 3H), 2.12 (dtt, *J* = 10.6, 5.5, 2.6 Hz, 1H), 2.07–1.99 (m, 1H), 1.30–1.23 (m, 1H), 0.88 (d, *J* = 2.8 Hz, 6H). ^13^C NMR (151 MHz, Chloroform-*d*) δ 171.66, 171.16, 168.35, 167.10, 165.71, 156.51, 136.66, 133.90, 119.72, 117.49, 116.37, 70.21, 69.44, 69.36, 67.33, 57.20, 55.87, 54.80, 50.77, 49.25, 35.36, 35.14, 31.52, 29.68, 23.04, 22.99, 22.73. LC-MS (*m*/*z*): positive mode 594.2 [M + H]^+^.

3-(2-chloro-*N*-(1,1-dioxidotetrahydrothiophen-3-yl)acetamido)-2,2-dimethylpropyl 3-(2-((2-(2,6-dioxopiperidin-3-yl)-1,3-dioxoisoindolin-4-yl)oxy)ethoxy)propanoate (**PC3**). **PC3** (65% yield, white solid) was synthesized from **5c** according to **General Procedure 4**. ^1^H NMR (600 MHz, Chloroform-*d*) δ 8.73 (s, 1H), 7.66 (dd, *J* = 8.5, 7.3 Hz, 1H), 7.44 (d, *J* = 7.2 Hz, 1H), 7.23 (d, *J* = 8.5 Hz, 1H), 4.95 (ddd, *J* = 12.3, 5.5, 2.7 Hz, 1H), 4.31 (t, *J* = 4.5 Hz, 2H), 4.12–4.08 (m, 2H), 3.89 (q, *J* = 4.9 Hz, 5H), 3.84–3.79 (m, 2H), 3.64 (tt, *J* = 12.7, 7.8 Hz, 2H), 3.36 (d, *J* = 15.8 Hz, 1H), 3.23 (d, *J* = 15.7 Hz, 1H), 3.12 (dq, *J* = 14.4, 7.6 Hz, 1H), 3.03–2.96 (m, 1H), 2.86–2.73 (m, 3H), 2.63 (t, *J* = 6.0 Hz, 2H), 2.46 (d, *J* = 7.6 Hz, 2H), 2.12–2.08 (m, 1H), 0.98–0.93 (m, 6H). ^13^C NMR (151 MHz, Chloroform-*d*) δ 171.49, 171.21, 168.69, 168.14, 167.05, 165.74, 156.38, 136.68, 133.83, 119.53, 117.31, 116.28, 69.61, 69.36, 69.29, 67.12, 57.81, 57.54, 53.56, 50.42, 49.21, 42.09, 36.71, 35.36, 31.45, 26.59, 23.18, 22.66, 22.64. HRMS (ESI) *m*/*z*: calcd for C_29_H_36_ClN_3_O_11_S, [M + Na], 692.1657; found, 692.1661.

*N*-(3-((1,1-dioxidotetrahydrothiophen-3-yl)amino)-2,2-dimethylpropyl)-6-((2-(2,6-dioxopiperidin-3-yl)-1,3-dioxoisoindolin-4-yl)oxy)hexanamide (**5d**). **5d** (30% yield, white solid) was synthesized according to **General Procedure 2**. ^1^H NMR (600 MHz, Chloroform-*d*) δ 8.52 (s, 1H), 7.68 (dd, *J* = 8.5, 7.3 Hz, 1H), 7.45 (d, *J* = 7.2 Hz, 1H), 7.22 (d, *J* = 8.5 Hz, 1H), 6.52 (td, *J* = 6.5, 3.1 Hz, 1H), 5.00–4.92 (m, 1H), 4.18 (td, *J* = 5.2, 1.8 Hz, 2H), 3.51 (p, *J* = 5.7 Hz, 1H), 3.30 (dt, *J* = 12.7, 7.8 Hz, 1H), 3.25–3.15 (m, 2H), 3.09–3.00 (m, 2H), 2.96 (dd, *J* = 13.3, 5.2 Hz, 1H), 2.91–2.85 (m, 1H), 2.86–2.71 (m, 2H), 2.39 (dtd, *J* = 13.0, 7.8, 4.8 Hz, 1H), 2.36–2.29 (m, 2H), 2.25 (t, *J* = 7.4 Hz, 2H), 2.15–2.10 (m, 2H), 1.96–1.86 (m, 2H), 1.73 (p, *J* = 7.4 Hz, 3H), 1.58 (h, *J* = 6.7 Hz, 2H), 0.95–0.85 (m, 6H). LC-MS (*m*/*z*): positive mode 591.2 [M + H]^+^.

*N*-(3-(2-chloro-*N*-(1,1-dioxidotetrahydrothiophen-3-yl)acetamido)-2,2-dimethylpropyl)-6-((2-(2,6-dioxopiperidin-3-yl)-1,3-dioxoisoindolin-4-yl)oxy)hexanamide (**PC4**). **PC4** (23% yield, white solid) was synthesized from **5d** according to **General Procedure 4**. ^1^H NMR (600 MHz, Chloroform-*d*) δ 8.99 (s, 1H), 7.67 (t, *J* = 7.9 Hz, 1H), 7.43 (d, *J* = 7.3 Hz, 1H), 7.21 (d, *J* = 8.5 Hz, 1H), 6.31 (t, *J* = 6.6 Hz, 1H), 5.01–4.93 (m, 1H), 4.17 (t, *J* = 6.0 Hz, 2H), 4.07 (d, *J* = 5.4 Hz, 1H), 3.91 (p, *J* = 8.4 Hz, 1H), 3.64 (ddd, *J* = 22.8, 13.1, 9.5 Hz, 2H), 3.32–3.06 (m, 5H), 3.01 (dt, *J* = 12.2, 5.8 Hz, 1H), 2.87–2.72 (m, 3H), 2.47 (dd, *J* = 9.2, 6.1 Hz, 2H), 2.26 (t, *J* = 7.4 Hz, 2H), 2.15–2.06 (m, 1H), 1.87 (dd, *J* = 14.7, 6.3 Hz, 3H), 1.73 (p, *J* = 7.5 Hz, 2H), 1.56 (p, *J* = 7.5 Hz, 2H), 0.96–0.83 (m, 6H). ^13^C NMR (151 MHz, Chloroform-*d*) δ 174.01, 171.61, 168.89, 167.92, 167.04, 166.07, 156.62, 136.79, 133.67, 119.13, 116.99, 115.92, 69.28, 58.70, 57.61, 50.48, 49.12, 47.99, 42.31, 37.96, 36.46, 31.41, 29.69, 29.32, 28.40, 26.52, 25.69, 25.25, 23.81, 22.61. HRMS (ESI) *m*/*z*: calcd for C_30_H_39_ClN_4_O_9_S, [M + Na], 689.2024; found, 689.2028.

*N*-(3-((1,1-dioxidotetrahydrothiophen-3-yl)amino)-2,2-dimethylpropyl)-4-((2-(2,6-dioxopiperidin-3-yl)-1,3-dioxoisoindolin-4-yl)oxy)butanamide (**5e**). **5e** (52% yield, white solid) was synthesized according to **General Procedure 2**. ^1^H NMR (600 MHz, Chloroform-*d*) δ 8.91–8.66 (m, 1H), 7.67 (dd, *J* = 8.5, 7.3 Hz, 1H), 7.44 (d, *J* = 7.3 Hz, 1H), 7.23 (dd, *J* = 8.5, 1.9 Hz, 1H), 7.14–6.94 (m, 1H), 5.01–4.96 (m, 1H), 4.22 (h, *J* = 4.7 Hz, 2H), 3.46 (dddd, *J* = 11.8, 7.0, 5.8, 1.8 Hz, 1H), 3.26 (tt, *J* = 13.0, 6.5 Hz, 2H), 3.13 (ddd, *J* = 15.5, 13.6, 6.5 Hz, 1H), 3.07–2.90 (m, 3H), 2.88–2.73 (m, 3H), 2.48 (tdd, *J* = 10.4, 6.4, 4.2 Hz, 2H), 2.41–2.28 (m, 3H), 2.21–2.02 (m, 4H), 1.86 (s, 1H), 0.82 (s, 6H). ^13^C NMR (151 MHz, Chloroform-*d*) δ 172.63, 171.47, 168.94, 167.11, 166.24, 156.49, 136.89, 133.78, 119.19, 117.28, 116.14, 67.93, 56.77, 55.93, 50.96, 49.23, 48.47, 48.12, 34.95, 32.37, 31.48, 29.80, 24.92, 24.26, 24.10, 22.72. LC-MS (*m*/*z*): positive mode 563.2 [M + H]^+^.

*N*-(3-(2-chloro-*N*-(1,1-dioxidotetrahydrothiophen-3-yl)acetamido)-2,2-dimethylpropyl)-4-((2-(2,6-dioxopiperidin-3-yl)-1,3-dioxoisoindolin-4-yl)oxy)butanamide (**PC5**). **PC5** (90% yield, white solid) was synthesized from **5e** according to **General Procedure 4**. ^1^H NMR (600 MHz, Chloroform-*d*) δ 9.20 (d, *J* = 7.6 Hz, 1H), 7.68 (t, *J* = 7.9 Hz, 1H), 7.45 (d, *J* = 7.3 Hz, 1H), 7.25 (d, *J* = 8.5 Hz, 1H), 6.77 (d, *J* = 7.0 Hz, 1H), 4.98 (t, *J* = 8.8 Hz, 1H), 4.23 (t, *J* = 5.8 Hz, 2H), 4.06 (d, *J* = 5.5 Hz, 1H), 3.89 (q, *J* = 8.1 Hz, 1H), 3.70–3.53 (m, 2H), 3.27 (d, *J* = 15.8 Hz, 1H), 3.22–3.04 (m, 4H), 2.99 (dt, *J* = 12.0, 5.7 Hz, 1H), 2.85–2.73 (m, 3H), 2.48 (dq, *J* = 36.5, 7.1 Hz, 4H), 2.21–2.06 (m, 4H), 0.92 (d, *J* = 6.3 Hz, 6H). ^13^C NMR (151 MHz, Chloroform-*d*) δ 173.37, 171.92, 169.08, 168.04, 167.00, 166.64, 156.23, 137.09, 133.68, 119.41, 117.28, 116.39, 67.77, 58.71, 57.66, 50.57, 49.26, 49.20, 48.11, 42.45, 37.85, 32.31, 31.47, 26.57, 24.68, 23.91, 23.88, 22.67. HRMS (ESI) *m*/*z*: calcd for C_28_H_35_ClN_4_O_9_S, [M + Na], 661.1711; found, 661.1713.

*N*-(3-((1,1-dioxidotetrahydrothiophen-3-yl)amino)-2,2-dimethylpropyl)-3-(2-((2-(2,6-dioxopiperidin-3-yl)-1,3-dioxoisoindolin-4-yl)oxy)ethoxy)propanamide (**5f**). **5f** (50% yield, white solid) was synthesized according to **General Procedure 2**. ^1^H NMR (600 MHz, Chloroform-*d*) δ 7.69 (dd, *J* = 8.5, 7.3 Hz, 1H), 7.47 (d, *J* = 7.3 Hz, 1H), 7.24 (d, *J* = 8.5 Hz, 1H), 6.92–6.85 (m, 1H), 4.97 (ddd, *J* = 12.2, 5.4, 3.1 Hz, 1H), 4.31 (tt, *J* = 3.9, 1.9 Hz, 2H), 3.94–3.80 (m, 4H), 3.48–3.41 (m, 1H), 3.31–3.18 (m, 2H), 3.16–2.90 (m, 5H), 2.88–2.72 (m, 3H), 2.49 (td, *J* = 5.7, 3.0 Hz, 2H), 2.39–2.32 (m, 1H), 2.30–2.21 (m, 2H), 2.16–2.01 (m, 3H), 0.79 (dd, *J* = 7.3, 1.9 Hz, 6H). ^13^C NMR (151 MHz, Chloroform-*d*) δ 172.00, 171.43, 171.41, 168.69, 168.67, 167.04, 165.99, 156.39, 136.88, 133.86, 119.47, 117.33, 116.46, 69.23, 69.21, 69.17, 68.08, 56.82, 56.03, 55.95, 55.91, 55.89, 50.72, 50.68, 49.28, 47.43, 47.39, 37.40, 35.30, 31.50, 29.66, 29.64, 24.22, 24.17, 24.15, 24.11, 22.69. LC-MS (*m*/*z*): positive mode 593.2 [M + H]^+^.

*N*-(3-(2-chloro-*N*-(1,1-dioxidotetrahydrothiophen-3-yl)acetamido)-2,2-dimethylpropyl)-3-(2-((2-(2,6-dioxopiperidin-3-yl)-1,3-dioxoisoindolin-4-yl)oxy)ethoxy)propanamide (**PC6**). **PC6** (70% yield, white solid) was synthesized from **5f** according to **General Procedure 4**. ^1^H NMR (600 MHz, Chloroform-*d*) δ 8.96 (d, *J* = 54.7 Hz, 1H), 7.74–7.70 (m, 1H), 7.52–7.48 (m, 1H), 7.24 (s, 1H), 6.88 (dt, *J* = 27.2, 6.6 Hz, 1H), 5.00–4.95 (m, 1H), 4.38–4.26 (m, 2H), 4.10–4.01 (m, 2H), 3.95–3.84 (m, 5H), 3.63 (dtd, *J* = 17.6, 12.9, 9.4 Hz, 2H), 3.23 (dd, *J* = 15.7, 7.5 Hz, 1H), 3.14 (dd, *J* = 15.7, 3.1 Hz, 1H), 3.10–3.07 (m, 1H), 3.03–2.92 (m, 2H), 2.88 (d, *J* = 13.6 Hz, 1H), 2.82–2.73 (m, 2H), 2.60–2.25 (m, 5H), 2.12 (dq, *J* = 7.7, 2.6 Hz, 1H), 0.84–0.75 (m, 6H). ^13^C NMR (151 MHz, Chloroform-*d*) δ 172.37, 171.32, 168.97, 168.02, 166.91, 166.31, 156.26, 137.15, 133.83, 119.31, 117.29, 116.72, 69.12, 68.96, 67.99, 58.86, 57.65, 50.56, 49.40, 49.22, 47.74, 42.49, 38.35, 37.32, 31.56, 26.57, 23.54, 23.48, 22.67. HRMS (ESI) *m*/*z*: calcd for C_29_H_37_ClN_4_O_10_S, [M + Na], 691.1817; found, 691.1819.

*N*-(3-((1,1-dioxidotetrahydrothiophen-3-yl)amino)-2,2-dimethylpropyl)-3-(3-((2-(2,6-dioxopiperidin-3-yl)-1,3-dioxoisoindolin-4-yl)amino)propoxy)propanamide (**5g**). **5g** (16% yield, white solid) was synthesized according to **General Procedure 2**. ^1^H NMR (600 MHz, Chloroform-*d*) δ 7.44 (dd, *J* = 8.4, 7.1 Hz, 1H), 7.14 (d, *J* = 7.1 Hz, 1H), 7.01 (q, *J* = 6.2 Hz, 1H), 6.91 (d, *J* = 8.4 Hz, 1H), 5.39 (s, 2H), 4.94 (ddt, *J* = 9.4, 5.7, 3.5 Hz, 1H), 3.91 (tt, *J* = 7.3, 3.6 Hz, 2H), 3.65 (t, *J* = 5.7 Hz, 2H), 3.52–3.45 (m, 3H), 3.41 (q, *J* = 6.0 Hz, 1H), 3.32–3.26 (m, 1H), 3.19 (dd, *J* = 13.2, 6.4 Hz, 1H), 3.14–3.08 (m, 1H), 3.03 (td, *J* = 11.6, 6.2 Hz, 2H), 2.97–2.89 (m, 2H), 2.80–2.72 (m, 2H), 2.44 (t, *J* = 5.6 Hz, 2H), 2.38–2.30 (m, 1H), 2.26 (s, 2H), 2.09 (dt, *J* = 13.4, 7.3 Hz, 2H), 1.84–1.79 (m, 2H), 0.87–0.84 (m, 6H). LC-MS (*m*/*z*): positive mode 606.3 [M + H]^+^.

*N*-(3-(2-chloro-*N*-(1,1-dioxidotetrahydrothiophen-3-yl)acetamido)-2,2-dimethylpropyl)-3-(3-((2-(2,6-dioxopiperidin-3-yl)-1,3-dioxoisoindolin-4-yl)amino)propoxy)propenamide (**PC7**). **PC7** (73% yield, white solid) was synthesized from **5g** according to **General Procedure 4**. ^1^H NMR (600 MHz, Chloroform-*d*) δ 77.41 (t, *J* = 7.7 Hz, 1H), 7.10 (d, *J* = 7.1 Hz, 1H), 7.03–6.95 (m, 1H), 6.89 (d, *J* = 8.4 Hz, 1H), 5.42 (s, 2H), 4.91 (s, 1H), 4.04 (d, *J* = 3.1 Hz, 2H), 3.88 (d, *J* = 7.0 Hz, 3H), 3.63 (qd, *J* = 12.0, 7.4 Hz, 4H), 3.45 (d, *J* = 7.2 Hz, 2H), 3.24 (dd, *J* = 15.8, 5.5 Hz, 1H), 3.18–3.04 (m, 4H), 3.00 (dq, *J* = 11.7, 5.9 Hz, 1H), 2.96–2.90 (m, 1H), 2.81–2.68 (m, 2H), 2.45 (t, *J* = 5.8 Hz, 4H), 2.10–2.06 (m, 1H), 1.83–1.79 (m, 2H), 0.87 (s, 6H). ^13^C NMR (151 MHz, Chloroform-*d*) δ 172.71, 171.27, 169.39, 169.10, 167.91, 167.80, 145.95, 135.75, 132.20, 121.73, 112.97, 110.38, 68.31, 66.94, 58.69, 57.51, 50.64, 49.69, 49.08, 47.98, 42.23, 37.92, 37.78, 37.20, 31.97, 28.09, 26.54, 23.73, 23.70, 22.00. HRMS (ESI) *m*/*z*: calcd for C_30_H_40_ClN_5_O_9_S, [M + Na], 704.2133; found, 704.2137.

*N*-(3-((1,1-dioxidotetrahydrothiophen-3-yl)amino)-2,2-dimethylpropyl)-3-(3-((2-(2,6-dioxopiperidin-3-yl)-1,3-dioxoisoindolin-4-yl)oxy)propoxy)propanamide (**5h**). **5h** (33% yield, white solid) was synthesized according to **General Procedure 2**. ^1^H NMR (600 MHz, Chloroform-*d*) δ 8.95 (s, 1H), 7.69 (dd, *J* = 8.5, 7.3 Hz, 1H), 7.46 (d, *J* = 7.3 Hz, 1H), 7.25 (d, *J* = 8.5 Hz, 1H), 6.78 (q, *J* = 6.1 Hz, 1H), 4.98 (ddd, *J* = 12.2, 5.5, 1.4 Hz, 1H), 4.27 (t, *J* = 6.1 Hz, 2H), 3.71 (dtdd, *J* = 16.0, 9.9, 7.2, 3.7 Hz, 4H), 3.46 (s, 2H), 3.30–3.26 (m, 1H), 3.24–3.20 (m, 1H), 3.10 (dt, *J* = 14.1, 7.1 Hz, 1H), 3.06–2.99 (m, 2H), 2.93 (ddd, *J* = 13.2, 5.8, 3.5 Hz, 1H), 2.89–2.85 (m, 1H), 2.82–2.72 (m, 2H), 2.48–2.44 (m, 2H), 2.38 (dtd, *J* = 12.8, 7.5, 4.9 Hz, 1H), 2.32–2.24 (m, 2H), 2.16–2.05 (m, 4H), 0.85 (t, *J* = 3.0 Hz, 6H). ^13^C NMR (151 MHz, Chloroform-*d*) δ 171.92, 171.50, 168.68, 167.05, 165.82, 156.40, 136.67, 133.75, 119.10, 117.14, 115.94, 67.07, 66.81, 66.01, 56.76, 55.76, 55.68, 50.54, 49.14, 47.19, 37.01, 35.19, 31.40, 29.64, 29.08, 24.20, 24.17, 22.61. LC-MS (*m*/*z*): positive mode 607.2 [M + H]^+^.

*N*-(3-(2-chloro-*N*-(1,1-dioxidotetrahydrothiophen-3-yl)acetamido)-2,2-dimethylpropyl)-3-(3-((2-(2,6-dioxopiperidin-3-yl)-1,3-dioxoisoindolin-4-yl)oxy)propoxy)propanamide (**PC8**). **PC8** (80% yield, white solid) was synthesized from **5h** according to **General Procedure 4**. ^1^H NMR (600 MHz, Chloroform-*d*) δ 9.16 (d, *J* = 2.8 Hz, 1H), 7.70–7.63 (m, 1H), 7.44 (d, *J* = 7.3 Hz, 1H), 7.23 (d, *J* = 8.5 Hz, 1H), 6.77 (d, *J* = 6.6 Hz, 1H), 4.96 (ddd, *J* = 11.8, 5.6, 3.1 Hz, 1H), 4.26 (t, *J* = 5.9 Hz, 2H), 4.06 (d, *J* = 4.8 Hz, 1H), 3.89 (t, *J* = 8.1 Hz, 1H), 3.77–3.57 (m, 6H), 3.24 (s, 1H), 3.16 (d, *J* = 15.6 Hz, 1H), 3.05 (dddd, *J* = 34.8, 25.6, 11.2, 4.2 Hz, 3H), 2.84 (d, *J* = 12.4 Hz, 1H), 2.76 (t, *J* = 11.2 Hz, 2H), 2.46 (d, *J* = 7.4 Hz, 4H), 2.09 (dd, *J* = 12.7, 6.8 Hz, 3H), 1.67 (s, 2H), 0.91–0.79 (m, 6H). ^13^C NMR (151 MHz, Chloroform-*d*) δ 172.43, 171.71, 168.97, 167.99, 167.03, 166.03, 156.24, 136.78, 133.75, 119.14, 117.18, 116.10, 66.89, 66.74, 65.83, 58.60, 57.56, 50.62, 49.20, 49.09, 47.90, 42.28, 37.80, 36.71, 31.40, 28.95, 26.51, 23.69, 23.60, 22.58. HRMS (ESI) *m*/*z*: calcd for C_30_H_39_ClN_4_O_10_S, [M + Na], 705.1973; found, 705.1974.

3-((1,1-dioxidotetrahydrothiophen-3-yl)amino)-2,2-dimethylpropyl 3-(3-((2-(2,6-dioxopiperidin-3-yl)-1,3-dioxoisoindolin-4-yl)amino)propoxy)propanoate (**5i**). **5i** (16% yield, white solid) was synthesized according to **General Procedure 1**. ^1^H NMR (600 MHz, Chloroform-*d*) δ 7.40 (dd, *J* = 8.3, 7.1 Hz, 1H), 7.12 (d, *J* = 7.1 Hz, 1H), 6.86 (d, *J* = 8.4 Hz, 1H), 5.30 (s, 2H), 4.91 (dd, *J* = 11.2, 5.6 Hz, 1H), 3.88 (d, *J* = 6.7 Hz, 4H), 3.65 (t, *J* = 6.2 Hz, 2H), 3.48 (d, *J* = 6.2 Hz, 1H), 3.46–3.44 (m, 3H), 3.24 (td, *J* = 12.3, 6.9 Hz, 2H), 3.00 (dtd, *J* = 13.0, 7.7, 1.9 Hz, 1H), 2.94–2.88 (m, 1H), 2.85–2.79 (m, 1H), 2.77–2.67 (m, 2H), 2.56 (t, *J* = 6.2 Hz, 2H), 2.41–2.31 (m, 3H), 2.09–1.98 (m, 2H), 1.80 (dq, *J* = 9.8, 6.7 Hz, 2H), 0.89 (s, 6H). ^13^C NMR (151 MHz, Chloroform-*d*) δ 171.85, 171.22, 169.20, 169.04, 167.79, 145.82, 135.63, 132.43, 121.56, 113.13, 110.84, 70.07, 68.92, 66.28, 57.25, 55.84, 54.67, 50.72, 49.80, 38.33, 35.32, 35.15, 32.09, 29.64, 28.01, 23.04, 22.97, 22.17. LC-MS (*m*/*z*): positive mode 607.2 [M + H]^+^.

3-(2-chloro-*N*-(1,1-dioxidotetrahydrothiophen-3-yl)acetamido)-2,2-dimethylpropyl 3-(3-((2-(2,6-dioxopiperidin-3-yl)-1,3-dioxoisoindolin-4-yl)amino)propoxy)propanoate (**PC9**). **PC9** (45% yield, white solid) was synthesized from **5i** according to **General Procedure 4**. ^1^H NMR (600 MHz, Chloroform-*d*) δ 7.42–7.38 (m, 1H), 7.11 (d, *J* = 7.1 Hz, 1H), 6.87 (d, *J* = 8.4 Hz, 1H), 5.33 (s, 2H), 4.90 (dd, *J* = 11.3, 5.5 Hz, 1H), 4.11 (d, *J* = 3.7 Hz, 2H), 3.92–3.82 (m, 5H), 3.70–3.61 (m, 4H), 3.45 (d, *J* = 6.5 Hz, 2H), 3.37 (d, *J* = 15.8 Hz, 1H), 3.24 (dd, *J* = 15.8, 5.2 Hz, 1H), 3.14–3.06 (m, 1H), 3.01 (d, *J* = 5.4 Hz, 1H), 2.95–2.87 (m, 1H), 2.77–2.68 (m, 2H), 2.58 (t, *J* = 6.1 Hz, 2H), 2.51–2.43 (m, 2H), 2.09–2.02 (m, 1H), 1.81–1.75 (m, 2H), 1.02–0.94 (m, 6H). ^13^C NMR (151 MHz, Chloroform-*d*) δ 171.38, 171.17, 169.09, 169.00, 168.04, 167.71, 145.79, 135.59, 132.30, 121.56, 113.02, 110.66, 69.50, 68.85, 66.10, 57.84, 57.52, 50.32, 49.69, 49.01, 42.01, 38.26, 36.73, 35.26, 31.98, 27.90, 26.55, 23.20, 23.16, 22.08. HRMS (ESI) *m*/*z*: calcd for C_30_H_39_ClN_4_O_10_S, [M + Na], 705.1973; found, 705.1975.

3-((1,1-dioxidotetrahydrothiophen-3-yl)amino)-2,2-dimethylpropyl 3-(3-((2-(2,6-dioxopiperidin-3-yl)-1,3-dioxoisoindolin-4-yl)oxy)propoxy)propanoate (**5j**). **5j** (26% yield, white solid) was synthesized according to **General Procedure 1**. ^1^H NMR (600 MHz, Chloroform-*d*) δ 8.56 (s, 1H), 7.66 (dd, *J* = 8.5, 7.3 Hz, 1H), 7.44–7.42 (m, 1H), 7.23 (d, *J* = 8.5 Hz, 1H), 4.95 (dd, *J* = 12.3, 5.4 Hz, 1H), 4.24 (t, *J* = 6.1 Hz, 2H), 3.85 (s, 2H), 3.71 (t, *J* = 6.2 Hz, 2H), 3.67 (t, *J* = 6.0 Hz, 2H), 3.51–3.45 (m, 1H), 3.28–3.21 (m, 2H), 3.01 (dtd, *J* = 13.1, 7.7, 2.5 Hz, 1H), 2.88–2.83 (m, 2H), 2.83–2.69 (m, 3H), 2.56 (t, *J* = 6.2 Hz, 2H), 2.40–2.31 (m, 3H), 2.14–2.06 (m, 3H), 2.02 (dq, *J* = 13.4, 7.6 Hz, 1H), 0.86 (d, *J* = 2.3 Hz, 6H). LC-MS (*m*/*z*): positive mode 608.2 [M + H]^+^.

3-(2-chloro-*N*-(1,1-dioxidotetrahydrothiophen-3-yl)acetamido)-2,2-dimethylpropyl 3-(3-((2-(2,6-dioxopiperidin-3-yl)-1,3-dioxoisoindolin-4-yl)oxy)propoxy)propanoate (**PC10**). **PC10** (90% yield, white solid) was synthesized from **5j** according to **General Procedure 4**. ^1^H NMR (600 MHz, Chloroform-*d*) δ 8.79 (s, 1H), 7.65 (dd, *J* = 8.5, 7.3 Hz, 1H), 7.41 (d, *J* = 7.3 Hz, 1H), 7.21 (d, *J* = 8.5 Hz, 1H), 4.94 (dd, *J* = 12.0, 5.4 Hz, 1H), 4.22 (t, *J* = 6.1 Hz, 2H), 4.12–4.04 (m, 2H), 3.93–3.86 (m, 1H), 3.82 (q, *J* = 10.5 Hz, 2H), 3.71 (t, *J* = 6.1 Hz, 2H), 3.67 (t, *J* = 6.0 Hz, 3H), 3.43 (s, 1H), 3.35 (dd, *J* = 15.8, 4.0 Hz, 1H), 3.22 (dd, *J* = 15.7, 3.9 Hz, 1H), 3.14–3.07 (m, 1H), 3.00 (d, *J* = 3.3 Hz, 1H), 2.83 (d, *J* = 14.9 Hz, 1H), 2.79–2.72 (m, 2H), 2.58 (t, *J* = 6.0 Hz, 2H), 2.46 (d, *J* = 6.5 Hz, 2H), 2.11–2.05 (m, 3H), 1.00–0.90 (m, 6H). ^13^C NMR (151 MHz, Chloroform-*d*) δ 171.65, 171.28, 168.41, 167.09, 165.73, 156.56, 136.59, 133.76, 119.05, 117.15, 115.84, 70.07, 66.88, 66.29, 66.07, 57.08, 55.74, 54.66, 50.60, 49.11, 35.18, 35.03, 31.39, 29.55, 29.22, 22.86, 22.83, 22.62. HRMS (ESI) *m*/*z*: calcd for C_30_H_38_ClN_3_O_11_S, [M + Na], 706.1813; found, 706.1815.

*N*-(3-((1,1-dioxidotetrahydrothiophen-3-yl)amino)-2,2-dimethylpropyl)-4-(4-(2-(2,6-dioxopiperidin-3-yl)-1,3-dioxoisoindolin-5-yl)piperazin-1-yl)-4-oxobutanamide (**10a**). **10a** (52% yield, green solid) was synthesized according to **General Procedure 2**. ^1^H NMR (600 MHz, Chloroform-*d*) δ 8.96 (s, 1H), 7.66 (d, *J* = 8.5 Hz, 1H), 7.23 (d, *J* = 2.4 Hz, 1H), 7.01 (dd, *J* = 8.6, 2.3 Hz, 1H), 6.73 (t, *J* = 6.4 Hz, 1H), 4.93 (dd, *J* = 12.2, 5.4 Hz, 1H), 3.75 (d, *J* = 5.8 Hz, 2H), 3.68 (t, *J* = 5.3 Hz, 2H), 3.50–3.38 (m, 5H), 3.28 (dt, *J* = 13.0, 7.6 Hz, 1H), 3.20 (dd, *J* = 13.3, 6.3 Hz, 1H), 3.15–2.98 (m, 4H), 2.95 (dd, *J* = 13.3, 5.8 Hz, 1H), 2.86–2.68 (m, 5H), 2.52 (t, *J* = 6.4 Hz, 2H), 2.38 (d, *J* = 11.5 Hz, 2H), 2.29 (d, *J* = 11.5 Hz, 1H), 2.13–2.06 (m, 2H), 0.88 (d, *J* = 8.8 Hz, 6H). ^13^C NMR (151 MHz, Chloroform-*d*) δ 172.86, 171.60, 171.00, 168.83, 167.88, 167.29, 155.13, 134.42, 125.48, 120.16, 118.17, 108.70, 56.90, 56.24, 56.03, 50.77, 49.38, 47.74, 47.29, 44.61, 41.18, 35.35, 31.58, 31.39, 29.87, 28.75, 24.48, 24.26, 22.85, 18.38. LC-MS (*m*/*z*): positive mode 645.3 [M + H]^+^.

*N*-(3-(2-chloro-*N*-(1,1-dioxidotetrahydrothiophen-3-yl)acetamido)-2,2-dimethylpropyl)-4-(4-(2-(2,6-dioxopiperidin-3-yl)-1,3-dioxoisoindolin-5-yl)piperazin-1-yl)-4-oxobutanamide (**PC11**). **PC11** (67% yield, green solid) was synthesized from **10a** according to **General Procedure 4**. ^1^H NMR (600 MHz, Chloroform-*d*) δ 9.31 (s, 1H), 7.65 (d, *J* = 8.4 Hz, 1H), 7.21 (s, 1H), 6.99 (d, *J* = 8.5 Hz, 1H), 6.58 (t, *J* = 6.6 Hz, 1H), 4.94 (dd, *J* = 12.1, 5.4 Hz, 1H), 4.21–4.12 (m, 2H), 3.91 (t, *J* = 8.1 Hz, 1H), 3.75 (d, *J* = 5.8 Hz, 2H), 3.65 (s, 3H), 3.47–3.37 (m, 4H), 3.32 (d, *J* = 15.8 Hz, 1H), 3.24 (d, *J* = 15.7 Hz, 1H), 3.21–3.12 (m, 2H), 3.08 (dd, *J* = 13.9, 6.1 Hz, 1H), 3.01 (p, *J* = 5.1 Hz, 1H), 2.85 (d, *J* = 12.9 Hz, 1H), 2.80–2.70 (m, 4H), 2.51 (d, *J* = 8.0 Hz, 4H), 2.10 (t, *J* = 4.2 Hz, 1H), 1.91 (s, 1H), 0.98 (d, *J* = 5.6 Hz, 6H). ^13^C NMR (151 MHz, Chloroform-*d*) δ 173.35, 171.92, 171.15, 169.10, 168.09, 167.89, 167.31, 155.00, 134.34, 125.51, 120.03, 118.11, 108.67, 58.15, 57.68, 50.62, 49.32, 49.16, 48.15, 47.17, 47.05, 44.55, 42.73, 41.20, 38.09, 31.61, 31.50, 28.78, 26.75, 24.10, 23.99, 22.83. HRMS (ESI) *m*/*z*: calcd for C_32_H_41_ClN_6_O_9_S, [M + Na], 743.2242; found, 743.2236.

*N*-(3-((1,1-dioxidotetrahydrothiophen-3-yl)amino)-2,2-dimethylpropyl)-4-(4-(2-(2,6-dioxopiperidin-3-yl)-1,3-dioxoisoindolin-5-yl)piperazin-1-yl)cyclohexane-1-carboxamide (**13a**). **13a** (35% yield, green solid) was synthesized according to **General Procedure 2**. ^1^H NMR (400 MHz, Methanol-*D*_4_) δ 7.66 (dd, *J* = 8.5, 3.9 Hz, 1H), 7.33 (d, *J* = 2.3 Hz, 1H), 7.22 (dt, *J* = 8.6, 2.8 Hz, 1H), 5.07 (dd, *J* = 12.5, 5.5 Hz, 1H), 3.54–3.49 (m, 4H), 3.34 (d, *J* = 6.4 Hz, 1H), 3.29–3.21 (m, 1H), 3.14–3.00 (m, 3H), 2.98–2.80 (m, 6H), 2.76 (dd, *J* = 4.3, 2.6 Hz, 1H), 2.73–2.68 (m, 1H), 2.57 (s, 1H), 2.46 (ddd, *J* = 26.5, 11.8, 5.6 Hz, 2H), 2.37 (d, *J* = 3.9 Hz, 2H), 2.11 (ddd, *J* = 13.3, 6.6, 4.1 Hz, 4H), 1.91 (q, *J* = 10.5 Hz, 2H), 1.76 (s, 1H), 1.61 (ddd, *J* = 13.5, 9.5, 4.0 Hz, 2H), 1.39–1.36 (m, 1H), 1.27 (d, *J* = 8.5 Hz, 1H), 0.92 (d, *J* = 5.5 Hz, 6H). LC-MS (*m*/*z*): positive mode 671.3 [M + H]^+^.

*N*-(3-(2-chloro-*N*-(1,1-dioxidotetrahydrothiophen-3-yl)acetamido)-2,2-dimethylpropyl)-4-(4-(2-(2,6-dioxopiperidin-3-yl)-1,3-dioxoisoindolin-5-yl)piperazin-1-yl)cyclohexane-1-carboxamide (**PC12**). **PC12** (73% yield, green solid) was synthesized from **13a** according to **General Procedure 4**. ^1^H NMR (600 MHz, Chloroform-*d*) δ 9.29 (s, 1H), 7.60 (dd, *J* = 8.5, 2.6 Hz, 1H), 7.21 (t, *J* = 2.7 Hz, 1H), 6.99 (d, *J* = 8.0 Hz, 1H), 6.21 (s, 1H), 4.91 (dd, *J* = 11.8, 5.6 Hz, 1H), 4.06 (d, *J* = 6.8 Hz, 1H), 3.89 (p, *J* = 8.3 Hz, 1H), 3.70–3.53 (m, 2H), 3.37 (s, 4H), 3.27 (d, *J* = 15.6 Hz, 1H), 3.18–3.07 (m, 3H), 3.06–2.93 (m, 2H), 2.78 (t, *J* = 10.7 Hz, 1H), 2.75–2.70 (m, 2H), 2.61 (s, 4H), 2.46 (d, *J* = 8.1 Hz, 2H), 2.37–2.20 (m, 3H), 2.08–1.91 (m, 3H), 1.81 (s, 2H), 1.52 (s, 4H), 0.97–0.82 (m, 6H). ^13^C NMR (151 MHz, Chloroform-*d*) δ 176.27, 171.95, 169.11, 168.02, 167.88, 167.36, 155.44, 134.17, 125.30, 119.05, 117.70, 108.43, 60.15, 58.76, 57.62, 56.80, 56.20, 55.78, 50.51, 49.11, 49.00, 47.75, 47.51, 42.35, 38.05, 35.17, 31.47, 29.72, 26.59, 26.24, 25.55, 24.31, 23.84, 23.77, 22.71. HRMS (ESI) *m*/*z*: calcd for C_35_H_47_ClN_6_O_8_S, [M + H], 747.2943; found, 747.2946.

3-((1,1-dioxidotetrahydrothiophen-3-yl)amino)-2,2-dimethylpropyl 4-(4-(2-(2,6-dioxopiperidin-3-yl)-1,3-dioxoisoindolin-5-yl)piperazin-1-yl)-4-oxobutanoate (**10b**). **10b** (66% yield, green solid) was synthesized according to **General Procedure 1**. ^1^H NMR (600 MHz, Chloroform-*d*) δ 8.71 (s, 1H), 7.68 (d, *J* = 8.5 Hz, 1H), 7.24 (d, *J* = 2.3 Hz, 1H), 7.03 (dd, *J* = 8.5, 2.4 Hz, 1H), 4.93 (dd, *J* = 12.4, 5.3 Hz, 1H), 3.94 (d, *J* = 10.9 Hz, 1H), 3.82 (d, *J* = 10.8 Hz, 1H), 3.79–3.75 (m, 2H), 3.68 (t, *J* = 4.4 Hz, 2H), 3.52–3.48 (m, 1H), 3.46 (dd, *J* = 6.6, 4.3 Hz, 2H), 3.41 (dt, *J* = 5.0, 2.7 Hz, 2H), 3.31–3.22 (m, 2H), 3.05–2.96 (m, 1H), 2.96–2.85 (m, 2H), 2.85–2.70 (m, 3H), 2.70–2.63 (m, 4H), 2.46–2.34 (m, 3H), 2.12–2.08 (m, 1H), 2.07–2.00 (m, 1H), 0.91 (s, 6H). LC-MS (*m*/*z*): positive mode 646.3 [M + H]^+^.

3-(2-chloro-*N*-(1,1-dioxidotetrahydrothiophen-3-yl)acetamido)-2,2-dimethylpropyl 4-(4-(2-(2,6-dioxopiperidin-3-yl)-1,3-dioxoisoindolin-5-yl)piperazin-1-yl)-4-oxobutanoate (**PC13**). **PC13** (64% yield, green solid) was synthesized from **10b** according to **General Procedure 4**. ^1^H NMR (600 MHz, Chloroform-*d*) δ 8.65 (s, 1H), 7.67 (d, *J* = 8.5 Hz, 1H), 7.24 (d, *J* = 2.3 Hz, 1H), 7.02 (dd, *J* = 8.6, 2.3 Hz, 1H), 4.94 (dd, *J* = 12.3, 5.3 Hz, 1H), 4.18–4.11 (m, 2H), 3.93 (t, *J* = 8.5 Hz, 2H), 3.84 (d, *J* = 11.4 Hz, 1H), 3.77 (t, *J* = 5.4 Hz, 2H), 3.69–3.60 (m, 4H), 3.48–3.46 (m, 1H), 3.43–3.40 (m, 2H), 3.38 (s, 1H), 3.32 (d, *J* = 15.8 Hz, 1H), 3.16 (dd, *J* = 13.1, 8.1 Hz, 1H), 3.02 (dt, *J* = 12.5, 6.1 Hz, 1H), 2.87–2.83 (m, 1H), 2.82–2.77 (m, 1H), 2.77–2.73 (m, 1H), 2.73–2.64 (m, 5H), 2.50 (td, *J* = 8.8, 5.3 Hz, 2H), 2.10 (ddd, *J* = 9.9, 5.1, 2.4 Hz, 1H), 1.02 (d, *J* = 6.8 Hz, 6H). ^13^C NMR (151 MHz, Chloroform-*d*) δ 173.08, 171.48, 170.02, 168.67, 168.17, 167.84, 167.24, 155.06, 134.35, 125.51, 120.23, 118.24, 108.80, 69.60, 57.57, 50.87, 50.51, 49.29, 49.24, 47.29, 47.25, 44.55, 42.22, 41.18, 36.89, 31.53, 29.21, 28.15, 26.65, 23.48, 23.25, 22.79. HRMS (ESI) *m*/*z*: calcd for C_32_H_40_ClN_5_O_10_S, [M + Na], 744.2082; found, 744.2086.

3-((1,1-dioxidotetrahydrothiophen-3-yl)amino)-2,2-dimethylpropyl 4-(4-(2-(2,6-dioxopiperidin-3-yl)-1,3-dioxoisoindolin-5-yl)piperazin-1-yl)cyclohexane-1-carboxylate (**13b**). **13b** (10% yield, green solid) was synthesized according to **General Procedure 1**. ^1^H NMR (400 MHz, Chloroform-*d*) δ 8.24 (s, 1H), 7.67 (d, *J* = 8.6 Hz, 1H), 7.26 (d, *J* = 2.4 Hz, 1H), 7.03 (dd, *J* = 8.6, 2.4 Hz, 1H), 4.92 (dd, *J* = 12.3, 5.3 Hz, 1H), 3.93–3.83 (m, 2H), 3.50 (dd, *J* = 6.9, 5.2 Hz, 1H), 3.41 (t, *J* = 5.1 Hz, 4H), 3.27 (dd, *J* = 13.3, 6.7 Hz, 2H), 3.06–2.98 (m, 1H), 2.90–2.83 (m, 2H), 2.82–2.74 (m, 2H), 2.67 (d, *J* = 6.1 Hz, 4H), 2.60–2.54 (m, 1H), 2.41–2.31 (m, 4H), 2.14–1.98 (m, 4H), 1.70 (s, 2H), 1.57 (t, *J* = 8.8 Hz, 5H), 0.91 (s, 6H). LC-MS (*m*/*z*): positive mode 672.3 [M + H]^+^.

3-(2-chloro-*N*-(1,1-dioxidotetrahydrothiophen-3-yl)acetamido)-2,2-dimethylpropyl 4-(4-(2-(2,6-dioxopiperidin-3-yl)-1,3-dioxoisoindolin-5-yl)piperazin-1-yl)cyclohexane-1-carboxylate (**PC14**). **PC14** (20% yield, green solid) was synthesized from **13b** according to **General Procedure 4**. ^1^H NMR (600 MHz, Chloroform-*d*) δ 8.60 (d, *J* = 4.1 Hz, 1H), 7.66 (d, *J* = 8.5 Hz, 1H), 7.25 (d, *J* = 2.3 Hz, 1H), 7.03 (dd, *J* = 8.6, 2.4 Hz, 1H), 4.93 (dd, *J* = 12.3, 5.4 Hz, 1H), 4.13–4.04 (m, 2H), 3.94–3.82 (m, 3H), 3.73–3.64 (m, 2H), 3.40 (t, *J* = 5.3 Hz, 5H), 3.26 (d, *J* = 15.7 Hz, 1H), 3.13–3.07 (m, 1H), 3.05–2.98 (m, 1H), 2.87–2.83 (m, 1H), 2.79 (dd, *J* = 12.8, 4.2 Hz, 1H), 2.76–2.73 (m, 1H), 2.73–2.69 (m, 1H), 2.66 (t, *J* = 5.1 Hz, 3H), 2.60 (t, *J* = 4.5 Hz, 1H), 2.54–2.44 (m, 2H), 2.32 (d, *J* = 11.9 Hz, 1H), 2.14–2.05 (m, 3H), 1.71 (d, *J* = 11.0 Hz, 2H), 1.64–1.56 (m, 4H), 1.02 (d, *J* = 4.0 Hz, 6H). ^13^C NMR (151 MHz, Chloroform-*d*) δ 174.55, 171.42, 168.63, 168.05, 168.00, 167.36, 155.55, 134.33, 125.42, 119.44, 117.87, 108.62, 69.33, 61.14, 58.03, 57.62, 53.56, 50.33, 49.22, 49.01, 48.91, 47.84, 41.88, 40.30, 36.85, 31.54, 26.64, 26.14, 25.69, 25.65, 23.33, 23.30, 22.83. HRMS (ESI) *m*/*z*: calcd for C_35_H_46_ClN_5_O_9_S, [M + H], 748.2783; found, 748.2789.

3-(2-chloro-*N*-(1,1-dioxidotetrahydrothiophen-3-yl)acetamido)-2,2-dimethylpropyl 4-((2-(1-methyl-2,6-dioxopiperidin-3-yl)-1,3-dioxoisoindolin-4-yl)oxy)butanoate (**PC2-Neg**). **PC2-Neg** (34% yield, white solid) ^1^H NMR (600 MHz, Chloroform-*d*) δ 7.67 (dd, *J* = 8.5, 7.3 Hz, 1H), 7.45 (d, *J* = 7.3 Hz, 1H), 7.22 (d, *J* = 8.5 Hz, 1H), 5.01–4.92 (m, 1H), 4.24 (t, *J* = 5.9 Hz, 2H), 4.13–4.03 (m, 2H), 3.87 (q, *J* = 11.5 Hz, 3H), 3.75–3.60 (m, 2H), 3.37 (d, *J* = 15.8 Hz, 1H), 3.26 (dd, *J* = 15.7, 4.3 Hz, 1H), 3.18 (s, 3H), 3.09 (dd, *J* = 12.8, 8.4 Hz, 1H), 3.02–2.92 (m, 2H), 2.81–2.74 (m, 2H), 2.67 (t, *J* = 7.1 Hz, 2H), 2.52–2.44 (m, 2H), 2.20 (p, *J* = 6.5 Hz, 2H), 2.11–2.04 (m, 1H), 1.00 (t, *J* = 2.3 Hz, 6H). ^13^C NMR (151 MHz, Chloroform-*d*) δ 172.66, 171.29, 169.04, 168.02, 167.21, 165.90, 156.28, 136.67, 133.95, 119.03, 117.44, 116.17, 69.74, 67.88, 58.01, 57.58, 50.37, 49.97, 49.08, 42.00, 36.68, 31.97, 30.26, 27.35, 26.54, 24.24, 23.38, 23.34, 22.02. HRMS (ESI) *m*/*z*: calcd for C_29_H_36_ClN_3_O_10_S, [M + Na], 676.1708; found, 676.1710.

*N*1-(3-(2-chloro-*N*-(1,1-dioxidotetrahydrothiophen-3-yl)acetamido)-2,2-dimethylpropyl)-*N*5-((S)-1-((2S,4R)-4-hydroxy-2-(((S)-1-(4-(4-methylthiazol-5-yl)phenyl)ethyl)carbamoyl)pyrrolidin-1-yl)-3,3-dimethyl-1-oxobutan-2-yl)glutaramide (**PV1**). **PV1** (40% yield, white solid) was synthesized from **16a** according to **General Procedure 2**. ^1^H NMR (600 MHz, Chloroform-*d*) δ 8.66 (s, 1H), 7.51 (dd, *J* = 7.9, 3.4 Hz, 1H), 7.36 (q, *J* = 8.3 Hz, 4H), 7.10 (d, *J* = 9.7 Hz, 1H), 7.02 (t, *J* = 6.1 Hz, 1H), 5.07 (p, *J* = 7.1 Hz, 1H), 4.66 (t, *J* = 8.2 Hz, 1H), 4.57 (d, *J* = 8.7 Hz, 1H), 4.47 (s, 1H), 4.14–4.03 (m, 3H), 3.93 (q, *J* = 7.5 Hz, 1H), 3.69–3.60 (m, 3H), 3.33–3.10 (m, 5H), 3.03 (dt, *J* = 23.4, 6.8 Hz, 2H), 2.50 (s, 3H), 2.29 (ddd, *J* = 20.1, 11.3, 5.4 Hz, 2H), 2.22–2.12 (m, 4H), 1.89 (ddt, *J* = 27.6, 13.8, 6.7 Hz, 2H), 1.46 (d, *J* = 6.9 Hz, 3H), 1.40 (ddd, *J* = 15.1, 11.1, 6.9 Hz, 2H), 1.03 (s, 9H), 0.99–0.92 (m, 6H). ^13^C NMR (151 MHz, Chloroform-*d*) δ 173.76, 173.57, 171.92, 170.03, 168.09, 150.41, 148.41, 143.31, 131.60, 130.79, 129.51, 126.44, 70.02, 58.83, 58.36, 57.94, 57.03, 54.88, 50.59, 49.26, 48.75, 47.97, 42.40, 37.54, 36.45, 35.26, 35.10, 35.00, 26.63, 26.54, 24.15, 23.99, 22.28, 22.24, 16.11. HRMS (ESI) *m*/*z*: calcd for C_39_H_57_ClN_6_O_8_S_2_, [M + Na], 859.3266; found, 859.3265.

*N*1-(3-(2-chloro-*N*-(1,1-dioxidotetrahydrothiophen-3-yl)acetamido)-2,2-dimethylpropyl)-*N*6-((*S*)-1-((2*S*,4*R*)-4-hydroxy-2-(((*S*)-1-(4-(4-methylthiazol-5-yl)phenyl)ethyl)carbamoyl)pyrrolidin-1-yl)-3,3-dimethyl-1-oxobutan-2-yl)adipamide (**PV2**). **PV2** (18% yield, white solid) was synthesized from **16b** according to **General Procedure 2**. ^1^H NMR (600 MHz, Chloroform-*d*) δ 8.66 (s, 1H), 7.50–7.45 (m, 1H), 7.39–7.32 (m, 4H), 6.79 (dd, *J* = 13.1, 9.2 Hz, 2H), 5.06 (p, *J* = 7.1 Hz, 1H), 4.66 (t, *J* = 8.1 Hz, 1H), 4.59 (d, *J* = 9.0 Hz, 1H), 4.47 (d, *J* = 5.0 Hz, 1H), 4.16–3.85 (m, 4H), 3.72–3.56 (m, 3H), 3.30 (dd, *J* = 15.7, 5.5 Hz, 1H), 3.24–3.17 (m, 2H), 3.10 (dt, *J* = 11.7, 5.5 Hz, 2H), 3.04–2.98 (m, 1H), 2.78 (s, 1H), 2.49 (s, 5H), 2.33 (q, *J* = 6.6 Hz, 1H), 2.25–2.12 (m, 4H), 2.09 (dd, *J* = 13.4, 8.1 Hz, 1H), 1.62–1.54 (m, 4H), 1.45 (d, *J* = 6.9 Hz, 3H), 1.01 (s, 9H), 0.95 (d, J = 10.7 Hz, 6H). ^13^C NMR (151 MHz, Chloroform-*d*) δ 174.03, 173.56, 171.92, 170.11, 168.12, 150.50, 148.49, 143.34, 131.65, 130.90, 129.60, 126.51, 69.96, 58.95, 58.59, 57.72, 57.60, 57.05, 50.62, 49.30, 49.21, 48.84, 48.17, 42.43, 37.86, 36.40, 36.03, 35.60, 35.48, 26.66, 25.14, 24.90, 24.07, 24.00, 22.32, 16.17. HRMS (ESI) *m*/*z*: calcd for C_40_H_59_ClN_6_O_8_S_2_, [M + Na], 873.3422; found, 873.3424.

3-(2-chloro-*N*-(1,1-dioxidotetrahydrothiophen-3-yl)acetamido)-2,2-dimethylpropyl 5-(((*S*)-1-((2*S*,4*R*)-4-hydroxy-2-(((*S*)-1-(4-(4-methylthiazol-5-yl)phenyl)ethyl)carbamoyl)pyrrolidin-1-yl)-3,3-dimethyl-1-oxobutan-2-yl)amino)-5-oxopentanoate (**PV3**). **PV3** (30% yield, colorless oil) was synthesized from **16c** according to **General Procedure 2**. ^1^H NMR (600 MHz, Chloroform-*d*) δ 8.62 (s, 1H), 7.41 (dd, *J* = 7.8, 1.9 Hz, 1H), 7.36–7.27 (m, 4H), 6.70–6.58 (m, 1H), 5.01 (p, *J* = 7.1 Hz, 1H), 4.62 (t, *J* = 7.8 Hz, 1H), 4.54 (d, *J* = 8.9 Hz, 1H), 4.43 (s, 1H), 4.13–4.01 (m, 3H), 3.95–3.75 (m, 4H), 3.68–3.55 (m, 3H), 3.36–3.21 (m, 2H), 3.14 (ddd, *J* = 21.0, 12.8, 8.3 Hz, 1H), 2.99 (dt, *J* = 17.3, 7.9 Hz, 1H), 2.44 (s, 5H), 2.33 (t, *J* = 6.6 Hz, 2H), 2.19 (tq, *J* = 15.0, 7.4 Hz, 2H), 2.05–1.95 (m, 2H), 1.87 (p, *J* = 7.1 Hz, 2H), 1.40 (d, *J* = 6.9 Hz, 3H), 0.96 (d, *J* = 8.9 Hz, 15H). ^13^C NMR (101 MHz, Chloroform-d) δ 172.61, 172.33, 171.68, 169.96, 167.99, 150.38, 148.28, 143.21, 131.52, 130.66, 129.41, 126.37, 69.79, 69.55, 60.31, 58.68, 57.48, 57.30, 56.69, 50.30, 49.03, 48.65, 41.92, 36.45, 35.99, 35.32, 34.77, 33.09, 26.46, 23.23, 23.17, 22.15, 20.62, 16.01, 14.12. HRMS (ESI) *m*/*z*: calcd for C_39_H_56_ClN_5_O_9_S_2_, [M + Na], 860.3106; found, 860.3111.

3-(2-chloro-*N*-(1,1-dioxidotetrahydrothiophen-3-yl)acetamido)-2,2-dimethylpropyl 6-(((S)-1-((2S,4R)-4-hydroxy-2-(((S)-1-(4-(4-methylthiazol-5-yl)phenyl)ethyl)carbamoyl)pyrrolidin-1-yl)-3,3-dimethyl-1-oxobutan-2-yl)amino)-6-oxohexanoate (**PV4**). **PV4** (56% yield, colorless oil) was synthesized from **16d** according to **General Procedure 2**. ^1^H NMR (600 MHz, Chloroform-*d*) δ 8.63 (s, 1H), 7.42 (dd, *J* = 7.9, 3.5 Hz, 1H), 7.35–7.29 (m, 4H), 6.48 (dd, *J* = 8.9, 3.3 Hz, 1H), 5.02 (p, *J* = 7.0 Hz, 1H), 4.63 (t, *J* = 7.8 Hz, 1H), 4.54 (d, *J* = 8.8 Hz, 1H), 4.44 (s, 1H), 4.16–3.73 (m, 7H), 3.70–3.54 (m, 3H), 3.39–2.93 (m, 4H), 2.46 (s, 5H), 2.32 (q, *J* = 7.4 Hz, 3H), 2.22–2.09 (m, 2H), 2.05–1.96 (m, 1H), 1.59 (q, *J* = 7.3 Hz, 4H), 1.41 (d, *J* = 6.9 Hz, 3H), 0.97 (d, *J* = 7.4 Hz, 15H). ^13^C NMR (151 MHz, Chloroform-*d*) δ 173.03, 172.97, 171.88, 169.96, 168.06, 150.44, 148.44, 143.27, 131.62, 130.85, 129.56, 126.49, 69.94, 69.59, 58.73, 57.91, 57.56, 57.48, 56.80, 50.33, 49.04, 48.94, 48.80, 41.95, 36.58, 35.88, 35.74, 35.33, 33.84, 26.56, 24.96, 24.35, 23.33, 23.26, 22.24, 16.12. HRMS (ESI) *m*/*z*: calcd for C_40_H_58_ClN_5_O_9_S_2_, [M + Na], 874.3262; found, 874.3266.

(2*S*,4*R*)-1-((2*S*)-2-(*tert*-butyl)-19-chloro-17-(1,1-dioxidotetrahydrothiophen-3-yl)-15,15-dimethyl-4,12,18-trioxo-3,5,13,17-tetraazanonadecanoyl)-4-hydroxy-*N*-((*S*)-1-(4-(4-methylthiazol-5-yl)phenyl)ethyl)pyrrolidine-2-carboxamide (**PV5**). **PV5** (36% yield, white solid) was synthesized from **19a** according to **General Procedure 3**. ^1^H NMR (600 MHz, Chloroform-*d*) δ 8.66 (s, 1H), 7.54–7.48 (m, 1H), 7.34 (q, *J* = 8.4 Hz, 4H), 6.67 (q, *J* = 5.9 Hz, 1H), 5.80 (d, *J* = 8.8 Hz, 1H), 5.47 (s, 1H), 5.05 (p, *J* = 7.0 Hz, 1H), 4.66 (t, *J* = 8.0 Hz, 1H), 4.46 (d, *J* = 19.8 Hz, 2H), 4.38 (d, *J* = 9.1 Hz, 1H), 4.18–4.02 (m, 3H), 3.97–3.90 (m, 1H), 3.76–3.53 (m, 3H), 3.34–3.26 (m, 1H), 3.22 (dd, *J* = 15.8, 12.8 Hz, 1H), 3.18–3.10 (m, 2H), 3.10–3.00 (m, 4H), 2.67 (s, 2H), 2.49 (s, 3H), 2.30 (s, 1H), 2.17 (t, *J* = 7.5 Hz, 2H), 2.08 (dd, *J* = 13.2, 8.2 Hz, 1H), 1.65–1.54 (m, 2H), 1.42 (dd, *J* = 21.0, 7.5 Hz, 5H), 1.27 (s, 4H), 1.05–0.92 (m, 15H). ^13^C NMR (151 MHz, Chloroform-*d*) δ 174.46, 173.72, 170.33, 168.07, 158.97, 150.53, 148.52, 143.39, 131.64, 130.92, 129.60, 126.53, 69.92, 58.84, 58.67, 57.67, 56.83, 53.55, 50.60, 49.35, 48.78, 48.13, 42.37, 40.19, 37.98, 36.41, 36.32, 35.05, 29.81, 28.62, 26.76, 26.68, 26.34, 25.52, 24.05, 23.98, 22.32, 16.21. HRMS (ESI) *m*/*z*: calcd for C_42_H_64_ClN_7_O_8_S_2_, [M + Na], 916.3844; found, 916.3843.

(2*S*,4*R*)-1-((2*S*)-2-(*tert*-butyl)-18-chloro-16-(1,1-dioxidotetrahydrothiophen-3-yl)-14,14-dimethyl-4,11,17-trioxo-8-oxa-3,5,12,16-tetraazaoctadecanoyl)-4-hydroxy-*N*-((*S*)-1-(4-(4-methylthiazol-5-yl)phenyl)ethyl)pyrrolidine-2-carboxamide (**PV6**). **PV6** (10% yield, white solid) was synthesized from **19b** according to **General Procedure 3**. ^1^H NMR (600 MHz, Chloroform-*d*) δ 8.66 (s, 1H), 7.46–7.41 (m, 1H), 7.38–7.33 (m, 4H), 7.12 (s, 1H), 6.07 (s, 1H), 5.89–5.76 (m, 1H), 5.10–5.03 (m, 1H), 4.63 (td, *J* = 8.3, 4.5 Hz, 1H), 4.45 (s, 1H), 4.36 (dd, *J* = 9.2, 3.0 Hz, 1H), 4.13–4.09 (m, 2H), 3.96 (q, *J* = 8.5 Hz, 1H), 3.75–3.58 (m, 5H), 3.47 (hept, *J* = 5.5 Hz, 2H), 3.31–3.21 (m, 4H), 3.11–3.00 (m, 2H), 2.83 (s, 4H), 2.53–2.41 (m, 7H), 2.28–2.22 (m, 1H), 2.14 (dd, *J* = 13.4, 8.2 Hz, 1H), 1.47–1.42 (m, 3H), 1.03–0.91 (m, 15H). ^13^C NMR (151 MHz, Chloroform-*d*) δ 173.45, 172.54, 170.34, 168.14, 159.27, 150.52, 148.48, 143.35, 131.64, 130.87, 129.57, 126.51, 70.00, 67.21, 58.97, 58.75, 58.51, 57.59, 57.05, 53.54, 50.66, 50.59, 49.34, 48.76, 48.05, 42.46, 37.91, 36.65, 35.19, 26.72, 26.62, 23.99, 23.92, 23.77, 22.28, 16.17. HRMS (ESI) *m*/*z*: calcd for C_40_H_60_ClN_7_O_9_S_2_, [M + Na], 904.3480; found, 904.3483.

(2*S*,4*R*)-1-((2*S*)-2-(*tert*-butyl)-18-chloro-16-(1,1-dioxidotetrahydrothiophen-3-yl)-14,14-dimethyl-4,11,17-trioxo-3,5,12,16-tetraazaoctadecanoyl)-4-hydroxy-*N*-((*S*)-1-(4-(4-methylthiazol-5-yl)phenyl)ethyl)pyrrolidine-2-carboxamide (**PV7**). **PV7** (50% yield, white solid) was synthesized from **19c** according to **General Procedure 3**. ^1^H NMR (600 MHz, Chloroform-*d*) δ 8.66 (s, 1H), 7.46 (d, *J* = 8.0 Hz, 1H), 7.39–7.33 (m, 4H), 6.69 (d, *J* = 7.2 Hz, 1H), 5.74 (s, 1H), 5.41 (d, *J* = 6.2 Hz, 1H), 5.07 (p, *J* = 7.0 Hz, 1H), 4.68 (t, *J* = 8.3 Hz, 1H), 4.45 (s, 1H), 4.40–4.31 (m, 2H), 4.20 (d, *J* = 11.5 Hz, 1H), 4.08 (d, *J* = 10.8 Hz, 2H), 3.92 (q, *J* = 8.2 Hz, 1H), 3.74–3.62 (m, 2H), 3.57 (dd, *J* = 11.5, 3.4 Hz, 1H), 3.30 (dd, *J* = 15.7, 7.2 Hz, 1H), 3.22 (dd, *J* = 15.7, 8.6 Hz, 1H), 3.11 (qd, *J* = 13.9, 8.8 Hz, 3H), 3.03 (t, *J* = 7.2 Hz, 2H), 2.50 (s, 5H), 2.38 (t, *J* = 3.9 Hz, 2H), 2.17 (s, 2H), 2.09 (dd, *J* = 13.4, 8.6 Hz, 1H), 1.61 (p, *J* = 6.8 Hz, 2H), 1.45 (d, *J* = 6.9 Hz, 5H), 1.31–1.24 (m, 2H), 1.06–0.95 (m, 15H). ^13^C NMR (151 MHz, Chloroform-*d*) δ 174.38, 174.07, 170.09, 168.08, 159.08, 150.50, 148.61, 143.29, 131.63, 131.03, 129.66, 126.54, 69.98, 58.87, 58.67, 57.69, 56.79, 53.56, 50.63, 49.35, 49.25, 48.85, 48.15, 42.38, 37.93, 36.37, 36.30, 34.86, 29.74, 26.81, 26.70, 26.30, 25.42, 24.15, 24.10, 22.37, 16.24. HRMS (ESI) *m*/*z*: calcd for C_41_H_62_ClN_7_O_8_S_2_, [M + Na], 902.3688; found, 902.3684.

(2*S*,4*R*)-1-((2*S*)-2-(*tert*-butyl)-17-chloro-15-(1,1-dioxidotetrahydrothiophen-3-yl)-13,13-dimethyl-4,10,16-trioxo-3,5,11,15-tetraazaheptadecanoyl)-4-hydroxy-*N*-((*S*)-1-(4-(4-methylthiazol-5-yl)phenyl)ethyl)pyrrolidine-2-carboxamide (**PV8**). **PV8** (18% yield, white solid) was synthesized from **19d** according to **General Procedure 3**. ^1^H NMR (600 MHz, Chloroform-*d*) δ ^1^H NMR (600 MHz, CDCl_3_) δ 8.66 (s, 1H), 7.53 (d, *J* = 7.7 Hz, 1H), 7.35 (t, *J* = 6.7 Hz, 4H), 7.00 (s, 1H), 5.99 (s, 1H), 5.69 (s, 1H), 5.06 (p, *J* = 7.1 Hz, 1H), 4.84 (s, 1H), 4.65 (t, *J* = 8.5 Hz, 1H), 4.48–4.35 (m, 2H), 4.17–4.04 (m, 3H), 3.98–3.88 (m, 1H), 3.72–3.55 (m, 3H), 3.31–3.18 (m, 3H), 3.08 (s, 5H), 2.48 (s, 5H), 2.29–2.10 (m, 4H), 1.62–1.54 (m, 2H), 1.46–1.39 (m, 5H), 1.04–0.91 (m, 15H). ^13^C NMR (151 MHz, Chloroform-*d*) δ 174.54, 173.67, 170.33, 168.17, 159.06, 150.55, 148.50, 143.38, 131.64, 130.89, 129.58, 126.55, 70.01, 59.03, 58.82, 58.47, 57.58, 57.00, 50.69, 49.37, 48.77, 48.02, 42.48, 39.65, 37.81, 36.67, 35.88, 35.25, 29.78, 26.80, 26.67, 24.19, 24.13, 22.99, 22.31, 16.21. HRMS (ESI) *m*/*z*: calcd for C_40_H_60_ClN_7_O_8_S_2_, [M + Na], 888.3531; found, 888.3531.

3-(2-chloro-*N*-(1,1-dioxidotetrahydrothiophen-3-yl)acetamido)-2,2-dimethylpropyl 7-(3-((*S*)-1-((2*S*,4*R*)-4-hydroxy-2-(((*S*)-1-(4-(4-methylthiazol-5-yl)phenyl)ethyl)carbamoyl)pyrrolidin-1-yl)-3,3-dimethyl-1-oxobutan-2-yl)ureido)heptanoate (**PV9**). **PV9** (30% yield, white solid) was synthesized from **19e** according to **General Procedure 3**. ^1^H NMR (600 MHz, Chloroform-*d*) δ 8.66 (s, 1H), 7.56 (dd, *J* = 7.9, 2.7 Hz, 1H), 7.36 (q, *J* = 8.3 Hz, 4H), 5.54 (dd, *J* = 9.1, 4.9 Hz, 1H), 5.13 (t, *J* = 5.6 Hz, 1H), 5.08–5.01 (m, 1H), 4.71 (t, *J* = 8.1 Hz, 1H), 4.44 (s, 1H), 4.36 (d, *J* = 9.0 Hz, 1H), 4.23 (d, *J* = 11.6 Hz, 1H), 4.14–4.04 (m, 2H), 3.94–3.80 (m, 4H), 3.69 (ddd, *J* = 31.3, 12.8, 9.6 Hz, 2H), 3.54 (dd, *J* = 11.6, 3.4 Hz, 1H), 3.38 (dd, *J* = 15.9, 2.4 Hz, 1H), 3.27 (d, *J* = 15.7 Hz, 1H), 3.12 (dtd, *J* = 12.5, 7.2, 2.7 Hz, 2H), 3.06–2.99 (m, 2H), 2.50 (s, 4H), 2.42 (ddd, *J* = 12.9, 8.0, 4.5 Hz, 1H), 2.34 (t, *J* = 7.5 Hz, 2H), 2.10–2.00 (m, 1H), 1.92 (d, *J* = 4.1 Hz, 1H), 1.67 (dp, *J* = 12.1, 3.9 Hz, 1H), 1.61 (p, *J* = 7.3 Hz, 2H), 1.43 (d, *J* = 7.0 Hz, 3H), 1.35–1.27 (m, 5H), 1.02 (d, *J* = 5.2 Hz, 15H). ^13^C NMR (151 MHz, Chloroform-*d*) δ 173.97, 173.35, 170.02, 168.15, 158.92, 150.44, 148.58, 143.40, 131.67, 130.94, 129.64, 126.54, 69.99, 58.75, 58.39, 58.10, 57.56, 56.68, 50.38, 49.05, 48.80, 41.91, 40.38, 36.70, 35.80, 34.69, 34.17, 34.04, 29.85, 28.73, 26.74, 26.49, 25.71, 25.06, 24.81, 23.45, 22.33, 16.22. HRMS (ESI) *m*/*z*: calcd for C_42_H_63_ClN_6_O_9_S_2_, [M + Na], 917.3684; found, 917.3690.

3-(2-chloro-*N*-(1,1-dioxidotetrahydrothiophen-3-yl)acetamido)-2,2-dimethylpropyl 3-(2-(3-((*S*)-1-((2*S*,4*R*)-4-hydroxy-2-(((*S*)-1-(4-(4-methylthiazol-5-yl)phenyl)ethyl)carbamoyl)pyrrolidin-1-yl)-3,3-dimethyl-1-oxobutan-2-yl)ureido)ethoxy)propanoate (**PV10**). **PV10** (33% yield, white solid) was synthesized from **19f** according to **General Procedure 3**. ^1^H NMR (600 MHz, Chloroform-*d*) δ 8.66 (s, 1H), 7.62 (d, *J* = 7.7 Hz, 1H), 7.39–7.34 (m, 4H), 5.73 (s, 1H), 5.59 (s, 1H), 5.06 (p, *J* = 7.1 Hz, 1H), 4.74 (q, *J* = 7.9 Hz, 1H), 4.45 (s, 1H), 4.35 (dd, *J* = 8.9, 5.7 Hz, 1H), 4.21 (d, *J* = 11.5 Hz, 1H), 4.09 (d, *J* = 10.9 Hz, 2H), 3.95–3.85 (m, 3H), 3.77–3.66 (m, 4H), 3.55 (dd, *J* = 11.5, 3.4 Hz, 1H), 3.48 (d, *J* = 5.3 Hz, 2H), 3.37 (dd, *J* = 15.8, 5.3 Hz, 1H), 3.30 (dt, *J* = 13.7, 5.4 Hz, 3H), 3.18 (td, *J* = 12.9, 8.1 Hz, 1H), 3.05 (ddd, *J* = 17.9, 12.2, 6.0 Hz, 1H), 2.60 (q, *J* = 5.5 Hz, 2H), 2.51 (s, 7H), 2.09 (dd, *J* = 12.5, 8.4 Hz, 1H), 1.44 (dd, *J* = 7.0, 1.2 Hz, 3H), 1.02 (d, *J* = 5.6 Hz, 15H). ^13^C NMR (151 MHz, Chloroform-*d*) δ 173.74, 171.63, 170.07, 168.35, 158.96, 150.41, 148.59, 143.43, 131.70, 130.91, 129.63, 126.57, 70.42, 70.14, 66.19, 58.79, 58.40, 58.17, 57.55, 56.71, 50.46, 49.32, 49.15, 48.86, 41.94, 40.12, 36.68, 35.84, 35.14, 34.83, 26.74, 26.62, 23.56, 23.48, 22.39, 16.23. HRMS (ESI) *m*/*z*: calcd for C_40_H_59_ClN_6_O_10_S_2_, [M + Na], 905.3320; found, 905.3320.

3-(2-chloro-*N*-(1,1-dioxidotetrahydrothiophen-3-yl)acetamido)-2,2-dimethylpropyl 6-(3-((*S*)-1-((2*S*,4*R*)-4-hydroxy-2-(((*S*)-1-(4-(4-methylthiazol-5-yl)phenyl)ethyl)carbamoyl)pyrrolidin-1-yl)-3,3-dimethyl-1-oxobutan-2-yl)ureido)hexanoate (**PV11**). **PV11** (6% yield, white solid) was synthesized from **19g** according to **General Procedure 3**. ^1^H NMR (600 MHz, Chloroform-*d*) 8.67 (s, 1H), 7.49 (d, *J* = 7.5 Hz, 1H), 7.41–7.33 (m, 4H), 5.51 (d, *J* = 9.0 Hz, 1H), 5.14 (q, *J* = 5.7 Hz, 1H), 5.07 (p, *J* = 7.1 Hz, 1H), 4.70 (td, *J* = 8.2, 2.3 Hz, 1H), 4.45 (s, 1H), 4.38 (d, *J* = 9.1 Hz, 1H), 4.21 (d, *J* = 11.5 Hz, 1H), 4.09 (dd, *J* = 7.1, 3.7 Hz, 1H), 3.93–3.80 (m, 3H), 3.69 (dd, *J* = 16.7, 10.1 Hz, 2H), 3.55 (dd, *J* = 11.5, 3.3 Hz, 1H), 3.38 (d, *J* = 15.8 Hz, 1H), 3.31–3.24 (m, 1H), 3.17–3.00 (m, 4H), 2.51 (d, *J* = 1.7 Hz, 5H), 2.35 (td, *J* = 7.4, 2.0 Hz, 2H), 2.11–2.06 (m, 2H), 1.63 (dt, *J* = 15.2, 7.5 Hz, 2H), 1.45 (d, *J* = 6.9 Hz, 5H), 1.34–1.29 (m, 2H), 1.25 (s, 2H), 1.01 (d, *J* = 7.1 Hz, 15H). ^13^C NMR (151 MHz, Chloroform-*d*) δ 173.83, 173.32, 170.07, 168.20, 158.93, 150.50, 148.59, 143.40, 131.71, 130.96, 129.67, 126.59, 70.04, 69.51, 58.73, 58.58, 58.05, 57.53, 56.87, 50.41, 49.15, 48.85, 41.94, 40.18, 36.66, 35.90, 34.87, 34.12, 29.75, 26.73, 26.53, 26.38, 24.57, 23.51, 22.31, 16.21. HRMS (ESI) *m*/*z*: calcd for C_41_H_61_ClN_6_O_9_S_2_, [M + Na], 905.3528; found, 905.3523.

3-(2-chloro-*N*-(1,1-dioxidotetrahydrothiophen-3-yl)acetamido)-2,2-dimethylpropyl 5-(3-((*S*)-1-((2*S*,4*R*)-4-hydroxy-2-(((*S*)-1-(4-(4-methylthiazol-5-yl)phenyl)ethyl)carbamoyl)pyrrolidin-1-yl)-3,3-dimethyl-1-oxobutan-2-yl)ureido)pentanoate (**PV12**). **PV12** (16% yield, white solid) was synthesized from **19h** according to **General Procedure 3**. ^1^H NMR (600 MHz, Chloroform-*d*) 8.67 (s, 1H), 7.53–7.51 (m, 1H), 7.40–7.34 (m, 4H), 5.58 (dd, *J* = 16.8, 9.0 Hz, 1H), 5.25 (q, *J* = 4.4 Hz, 1H), 5.11–5.02 (m, 1H), 4.71 (t, *J* = 8.2 Hz, 1H), 4.45 (t, *J* = 3.6 Hz, 1H), 4.36 (dd, *J* = 9.1, 3.2 Hz, 1H), 4.21 (qdd, *J* = 10.9, 5.6, 2.2 Hz, 2H), 4.14–4.05 (m, 2H), 3.92–3.87 (m, 1H), 3.86–3.81 (m, 2H), 3.75–3.65 (m, 2H), 3.54 (dd, *J* = 11.5, 3.3 Hz, 1H), 3.40–3.25 (m, 2H), 3.12 (dq, *J* = 12.6, 5.9 Hz, 3H), 2.51 (s, 5H), 2.45–2.40 (m, 1H), 2.37 (td, *J* = 7.4, 3.4 Hz, 2H), 2.12–2.05 (m, 2H), 1.69–1.59 (m, 3H), 1.50–1.43 (m, 5H), 1.01 (m, 15H). ^13^C NMR (151 MHz, Chloroform-*d*) δ 173.96, 173.24, 170.06, 168.21, 158.93, 150.46, 148.55, 143.36, 131.65, 130.93, 129.61, 126.55, 69.98, 69.43, 58.79, 58.54, 57.91, 57.45, 56.82, 50.41, 48.76, 41.98, 39.80, 36.60, 35.98, 34.79, 33.87, 31.74, 29.78, 29.67, 26.75, 23.49, 23.37, 22.29, 22.27, 16.20. HRMS (ESI) *m*/*z*: calcd for C_40_H_59_ClN_6_O_9_S_2_, [M + Na], 889.3371; found, 889.3372.

(2S,4R)-1-((2S)-2-(tert-butyl)-21-chloro-19-(1,1-dioxidotetrahydrothiophen-3-yl)-17,17-dimethyl-4,14,20-trioxo-6,9,12-trioxa-3,15,19-triazahenicosanoyl)-4-hydroxy-N-((S)-1-(4-(4-methylthiazol-5-yl)phenyl)ethyl)pyrrolidine-2-carboxamide (**PV13**). **PV13** (72% yield, colorless oil) was synthesized from **16e** according to **General Procedure 2**. ^1^H NMR (600 MHz, Chloroform-*d*) δ 8.66 (s, 1H), 7.38 (q, *J* = 8.4 Hz, 5H), 7.30 (dd, *J* = 9.3, 2.9 Hz, 1H), 7.15 (dt, *J* = 14.4, 6.7 Hz, 1H), 5.08 (t, *J* = 6.6 Hz, 1H), 4.65–4.59 (m, 2H), 4.50–4.48 (m, 1H), 4.11–3.91 (m, 8H), 3.80–3.61 (m, 12H), 3.34–3.14 (m, 4H), 3.09 (dd, *J* = 13.6, 6.1 Hz, 1H), 3.04 (t, *J* = 10.3 Hz, 1H), 2.52 (s, 5H), 2.35 (tt, *J* = 8.4, 3.7 Hz, 1H), 2.13–2.09 (m, 1H), 1.49 (dd, *J* = 7.0, 2.1 Hz, 3H), 1.03 (s, 9H), 1.01–0.94 (m, 6H). ^13^C NMR (151 MHz, Chloroform-*d*) δ 171.25, 169.69, 162.68, 150.41, 148.59, 143.48, 131.75, 130.91, 129.65, 126.54, 71.02, 70.59, 70.53, 70.38, 70.33, 70.28, 70.26, 58.80, 57.65, 57.12, 56.91, 50.55, 49.25, 49.18, 48.94, 47.55, 42.26, 38.05, 36.61, 35.93, 31.57, 26.59, 23.99, 23.82, 22.46, 16.23. HRMS (ESI) *m*/*z*: calcd for C_42_H_63_ClN_6_O_11_S_2_, [M + Na], 949.3582; found, 949.3582.

4-(4-((3-(2-chloro-*N*-(1,1-dioxidotetrahydrothiophen-3-yl)acetamido)-2,2-dimethylpropyl)carbamoyl)phenyl)-*N*-((*S*)-1-((2*S*,4*R*)-4-hydroxy-2-(((*S*)-1-(4-(4-methylthiazol-5-yl)phenyl)ethyl)carbamoyl)pyrrolidin-1-yl)-3,3-dimethyl-1-oxobutan-2-yl)piperazine-1-carboxamide (**PV14**). **PV14** (24% yield, white solid) was synthesized from **19i** according to **General Procedure 3**. ^1^H NMR (600 MHz, Chloroform-*d*) δ 8.66 (s, 1H), 7.76 (d, *J* = 8.2 Hz, 1H), 7.67 (d, *J* = 8.4 Hz, 2H), 7.57 (d, *J* = 7.9 Hz, 1H), 7.37 (d, *J* = 2.2 Hz, 4H), 6.79 (d, *J* = 8.6 Hz, 2H), 6.62 (t, *J* = 6.7 Hz, 1H), 5.16 (d, *J* = 8.8 Hz, 1H), 5.09–5.06 (m, 1H), 4.70 (t, *J* = 8.1 Hz, 1H), 4.45 (s, 1H), 4.40 (d, *J* = 8.9 Hz, 1H), 4.13–4.03 (m, 3H), 3.97–3.90 (m, 1H), 3.71–3.61 (m, 2H), 3.57 (dd, *J* = 11.5, 3.5 Hz, 1H), 3.48 (qd, *J* = 13.0, 6.4 Hz, 4H), 3.39 (dd, *J* = 15.8, 2.6 Hz, 1H), 3.31 (dd, *J* = 21.1, 5.3 Hz, 3H), 3.25 (d, *J* = 5.9 Hz, 5H), 3.11 (dt, *J* = 13.3, 6.8 Hz, 1H), 3.02–2.96 (m, 1H), 2.48 (s, 5H), 2.08–1.99 (m, 1H), 1.47 (d, *J* = 6.9 Hz, 3H), 1.05 (s, 15H). ^13^C NMR (151 MHz, Chloroform-*d*) δ 173.23, 169.96, 167.95, 167.88, 157.86, 152.95, 150.49, 148.54, 143.38, 131.65, 130.94, 129.62, 128.67, 126.57, 124.02, 114.35, 70.07, 59.12, 58.94, 58.58, 57.70, 56.65, 50.51, 49.20, 48.91, 48.59, 47.29, 43.33, 42.30, 38.45, 35.97, 35.09, 26.73, 26.65, 24.13, 22.43, 16.17. HRMS (ESI) *m*/*z*: calcd for C_46_H_63_ClN_8_O_8_S_2_, [M + Na], 977.3796; found, 977.3795.

3-(2-chloro-*N*-(1,1-dioxidotetrahydrothiophen-3-yl)acetamido)-2,2-dimethylpropyl 4-(4-(((*S*)-1-((2*S*,4*R*)-4-hydroxy-2-(((*S*)-1-(4-(4-methylthiazol-5-yl)phenyl)ethyl)carbamoyl)pyrrolidin-1-yl)-3,3-dimethyl-1-oxobutan-2-yl)carbamoyl)piperazin-1-yl)benzoate (**PV15**). **PV15** (26% yield, white solid) was synthesized from **19l** according to **General Procedure 3**. ^1^H NMR (600 MHz, Chloroform-*d*) δ 8.65 (s, 1H), 7.87 (d, *J* = 8.6 Hz, 2H), 7.55 (d, *J* = 7.9 Hz, 1H), 7.40–7.34 (m, 4H), 6.84–6.76 (m, 2H), 5.14 (d, *J* = 8.6 Hz, 1H), 5.07 (t, *J* = 7.2 Hz, 1H), 4.76–4.69 (m, 1H), 4.47 (s, 1H), 4.40 (d, *J* = 8.9 Hz, 1H), 4.16–4.10 (m, 2H), 4.08 (s, 1H), 4.05 (d, *J* = 3.0 Hz, 2H), 3.97–3.93 (m, 1H), 3.72–3.64 (m, 2H), 3.59–3.47 (m, 6H), 3.33 (d, *J* = 5.0 Hz, 4H), 3.12 (dd, *J* = 12.8, 8.4 Hz, 1H), 3.03–2.97 (m, 1H), 2.89 (d, *J* = 46.3 Hz, 1H), 2.49 (d, *J* = 1.2 Hz, 5H), 2.44 (dd, *J* = 10.9, 6.2 Hz, 1H), 2.04 (dd, *J* = 13.5, 8.3 Hz, 1H), 1.46 (d, *J* = 7.0 Hz, 3H), 1.39 (dd, *J* = 8.4, 3.7 Hz, 1H), 1.06 (d, *J* = 22.7 Hz, 15H). ^13^C NMR (151 MHz, Chloroform-*d*) δ 173.28, 169.82, 168.09, 165.94, 157.82, 153.82, 150.43, 148.52, 143.30, 131.60, 131.36, 130.94, 129.60, 126.54, 119.00, 113.66, 70.06, 69.39, 59.11, 58.45, 58.25, 57.73, 56.55, 50.39, 49.06, 48.91, 46.72, 43.20, 41.89, 37.13, 35.74, 34.97, 26.70, 26.64, 23.40, 22.41, 16.16. HRMS (ESI) *m*/*z*: calcd for C_46_H_62_ClN_7_O_9_S_2_, [M + Na], 978.3637; found, 978.3630.

3-(2-chloro-*N*-(1,1-dioxidotetrahydrothiophen-3-yl)acetamido)-2,2-dimethylpropyl 2-(4-(((*S*)-1-((2*S*,4*R*)-4-hydroxy-2-(((*S*)-1-(4-(4-methylthiazol-5-yl)phenyl)ethyl)carbamoyl)pyrrolidin-1-yl)-3,3-dimethyl-1-oxobutan-2-yl)carbamoyl)piperazin-1-yl)acetate (**PV16**). **PV16** (17% yield, white solid) was synthesized from **19m** according to **General Procedure 3**. ^1^H NMR (600 MHz, Chloroform-*d*) δ 8.67 (s, 1H), 7.55 (d, *J* = 7.9 Hz, 1H), 7.41–7.34 (m, 4H), 5.08 (dq, *J* = 21.5, 8.0 Hz, 2H), 4.73 (t, *J* = 8.1 Hz, 1H), 4.46 (t, *J* = 2.2 Hz, 1H), 4.35 (d, *J* = 9.0 Hz, 1H), 4.19 (dd, *J* = 11.6, 1.8 Hz, 1H), 4.09 (d, *J* = 4.6 Hz, 2H), 3.90 (q, *J* = 11.5 Hz, 3H), 3.68 (dt, *J* = 38.8, 12.0 Hz, 2H), 3.53 (dd, *J* = 11.5, 3.4 Hz, 1H), 3.45–3.36 (m, 5H), 3.32–3.25 (m, 3H), 3.11 (s, 1H), 3.03 (dp, *J* = 12.2, 5.0 Hz, 1H), 2.58 (d, *J* = 5.4 Hz, 4H), 2.54–2.47 (m, 6H), 2.09–2.04 (m, 1H), 1.46 (d, *J* = 7.0 Hz, 3H), 1.40 (s, 1H), 1.03 (d, *J* = 8.2 Hz, 15H). ^13^C NMR (151 MHz, Chloroform-*d*) δ 173.58, 169.82, 169.66, 168.06, 158.04, 150.46, 148.59, 143.33, 131.72, 130.98, 129.69, 126.58, 70.11, 69.82, 59.22, 59.00, 58.33, 58.05, 57.62, 56.60, 52.42, 50.33, 48.98, 43.83, 41.91, 36.74, 35.57, 34.83, 31.75, 29.77, 26.77, 26.58, 23.41, 22.45, 16.20. HRMS (ESI) *m*/*z*: calcd for C_41_H_60_ClN_7_O_9_S_2_, [M + Na], 916.3480; found, 916.3486.

3-(2-chloro-*N*-(1,1-dioxidotetrahydrothiophen-3-yl)acetamido)-2,2-dimethylpropyl 3-(1-(((*S*)-1-((2*S*,4*R*)-4-hydroxy-2-(((*S*)-1-(4-(4-methylthiazol-5-yl)phenyl)ethyl)carbamoyl)pyrrolidin-1-yl)-3,3-dimethyl-1-oxobutan-2-yl)carbamoyl)piperidin-4-yl)propanoate (**PV17**). **PV17** (13% yield, white solid) was synthesized from **19n** according to **General Procedure 3**. ^1^H NMR (600 MHz, Chloroform-*d*) δ 8.67 (s, 1H), 7.59 (d, *J* = 8.3 Hz, 1H), 7.39–7.35 (m, 4H), 5.12–5.02 (m, 2H), 4.71 (t, *J* = 8.1 Hz, 1H), 4.43 (tt, *J* = 3.4, 1.4 Hz, 1H), 4.35 (d, *J* = 9.0 Hz, 1H), 4.19 (d, *J* = 11.7 Hz, 1H), 4.13–4.03 (m, 2H), 3.96–3.79 (m, 5H), 3.73–3.63 (m, 2H), 3.53 (dd, *J* = 11.5, 3.4 Hz, 1H), 3.38 (d, *J* = 15.8 Hz, 1H), 3.26 (d, *J* = 15.7 Hz, 1H), 3.09 (dd, *J* = 12.4, 8.6 Hz, 1H), 3.01 (dd, *J* = 13.2, 6.5 Hz, 1H), 2.72 (dtd, *J* = 33.7, 13.0, 2.7 Hz, 2H), 2.52–2.42 (m, 6H), 2.38–2.35 (m, 2H), 2.05 (dd, *J* = 13.8, 8.3 Hz, 1H), 1.66 (d, *J* = 11.3 Hz, 2H), 1.58 (q, *J* = 7.4 Hz, 2H), 1.46 (d, *J* = 7.0 Hz, 4H), 1.24 (s, 1H), 1.14–1.08 (m, 2H), 1.05–1.00 (m, 15H). ^13^C NMR (151 MHz, Chloroform-*d*) δ 173.53, 172.96, 169.86, 167.93, 157.90, 150.39, 148.44, 143.29, 131.64, 130.81, 129.55, 126.47, 69.97, 69.51, 59.05, 58.28, 57.99, 57.54, 56.49, 50.24, 48.94, 48.85, 44.24, 41.76, 36.62, 35.56, 35.24, 34.74, 31.65, 31.52, 31.42, 31.28, 31.10, 29.65, 26.65, 26.34, 23.29, 22.34, 16.07. HRMS (ESI) *m*/*z*: calcd for C_43_H_63_ClN_6_O_9_S_2_, [M + Na], 929.3684; found, 929.3687.

(2*S*,4*R*)-1-((2*S*)-2-(2-(3-(2-((3-(2-chloro-*N*-(1,1-dioxidotetrahydrothiophen-3-yl)acetamido)-2,2-dimethylpropyl)amino)-2-oxoethyl)phenyl)acetamido)-3,3-dimethylbutanoyl)-4-hydroxy-*N*-((*S*)-1-(4-(4-methylthiazol-5-yl)phenyl)ethyl)pyrrolidine-2-carboxamide (**PV18**). **PV18** (16% yield, white solid) was synthesized from **16f** according to **General Procedure 2**. ^1^H NMR (600 MHz, Chloroform-*d*) δ 8.65 (s, 1H), 7.38–7.33 (m, 5H), 7.28–7.26 (m, 1H), 7.17–7.09 (m, 3H), 6.68 (dd, *J* = 19.7, 8.9 Hz, 1H), 6.47 (dt, *J* = 22.2, 6.5 Hz, 1H), 5.09–5.03 (m, 1H), 4.64 (dt, *J* = 11.3, 8.0 Hz, 1H), 4.56 (dd, *J* = 9.0, 3.2 Hz, 1H), 4.43 (s, 1H), 4.23–4.14 (m, 1H), 3.92 (dt, *J* = 20.1, 12.3 Hz, 2H), 3.80 (s, 1H), 3.65–3.56 (m, 3H), 3.50–3.47 (m, 3H), 3.13–2.95 (m, 6H), 2.56 (s, 2H), 2.49 (d, *J* = 1.1 Hz, 3H), 2.45–2.37 (m, 2H), 2.29 (dddd, *J* = 18.6, 13.0, 8.3, 4.7 Hz, 1H), 2.04 (dt, *J* = 13.6, 7.0 Hz, 1H), 1.45 (d, *J* = 6.9 Hz, 3H), 0.98 (s, 9H), 0.89–0.82 (m, 6H). ^13^C NMR (151 MHz, Chloroform-*d*) δ 171.97, 171.63, 171.06, 170.12, 168.31, 150.46, 148.52, 143.35, 135.76, 135.72, 131.65, 130.92, 130.32, 129.61, 129.39, 128.46, 127.79, 126.52, 69.99, 58.96, 58.30, 57.76, 57.67, 57.03, 50.55, 49.35, 48.86, 48.15, 43.79, 42.72, 42.41, 38.12, 36.17, 35.63, 26.67, 26.57, 23.93, 23.85, 22.31, 16.17. HRMS (ESI) *m*/*z*: calcd for C_44_H_59_ClN_6_O_8_S_2_, [M + Na], 921.3422; found, 921.3423.

(2*S*,4*R*)-1-((2*S*)-2-(2-(4-(2-((3-(2-chloro-*N*-(1,1-dioxidotetrahydrothiophen-3-yl)acetamido)-2,2-dimethylpropyl)amino)-2-oxoethyl)phenyl)acetamido)-3,3-dimethylbutanoyl)-4-hydroxy-*N*-((*S*)-1-(4-(4-methylthiazol-5-yl)phenyl)ethyl)pyrrolidine-2-carboxamide (**PV19**). **PV19** (20% yield, white solid) was synthesized from **16g** according to **General Procedure 4**. ^1^H NMR (600 MHz, Chloroform-*d*) δ 8.65 (s, 1H), 7.41–7.33 (m, 5H), 7.20 (q, *J* = 7.8 Hz, 4H), 6.58 (dd, *J* = 16.0, 8.8 Hz, 1H), 6.33–6.23 (m, 1H), 5.05 (p, *J* = 7.1 Hz, 1H), 4.62 (t, *J* = 7.9 Hz, 1H), 4.57–4.53 (m, 1H), 4.42 (s, 1H), 4.04–3.85 (m, 4H), 3.77 (q, *J* = 8.9 Hz, 1H), 3.59 (dt, *J* = 11.3, 5.5 Hz, 3H), 3.50 (s, 4H), 3.15–2.96 (m, 6H), 2.49 (s, 3H), 2.44–2.39 (m, 2H), 2.30 (dq, *J* = 8.2, 4.2 Hz, 1H), 2.05–1.99 (m, 1H), 1.45 (d, *J* = 6.9 Hz, 3H), 0.98 (s, 9H), 0.88–0.82 (m, 6H). ^13^C NMR (151 MHz, Chloroform-*d*) δ 171.85, 171.56, 171.15, 170.12, 168.16, 168.04, 150.47, 148.52, 143.37, 134.18, 134.12, 131.66, 130.91, 130.22, 130.17, 129.61, 129.48, 126.52, 69.98, 58.91, 58.44, 57.81, 57.71, 56.90, 50.51, 49.12, 48.88, 48.07, 43.47, 42.56, 42.36, 38.11, 36.60, 35.64, 26.59, 23.99, 23.87, 23.74, 22.33, 16.18. HRMS (ESI) *m*/*z*: calcd for C_44_H_59_ClN_6_O_8_S_2_, [M + Na], 921.3422; found, 921.3420.

4-(2-((3-(2-chloro-*N*-(1,1-dioxidotetrahydrothiophen-3-yl)acetamido)-2,2-dimethylpropyl)amino)-2-oxoethyl)-*N*-((*S*)-1-((2*S*,4*R*)-4-hydroxy-2-(((*S*)-1-(4-(4-methylthiazol-5-yl)phenyl)ethyl)carbamoyl)pyrrolidin-1-yl)-3,3-dimethyl-1-oxobutan-2-yl)piperazine-1-carboxamide (**PV20**). **PV20** (19% yield, white solid) was synthesized from **19j** according to **General Procedure 3**. ^1^H NMR (600 MHz, Chloroform-*d*) δ 8.66 (s, 1H), 7.49 (d, *J* = 8.0 Hz, 1H), 7.38 (d, *J* = 8.3 Hz, 2H), 7.35 (d, *J* = 8.3 Hz, 2H), 7.28 (t, *J* = 7.0 Hz, 1H), 5.13 (d, *J* = 8.8 Hz, 1H), 5.06 (q, *J* = 7.1 Hz, 1H), 4.71 (t, *J* = 8.0 Hz, 1H), 4.45 (s, 1H), 4.36 (d, *J* = 8.8 Hz, 1H), 4.14 (d, *J* = 11.5 Hz, 1H), 4.05 (t, *J* = 9.0 Hz, 2H), 3.98–3.86 (m, 2H), 3.74–3.60 (m, 2H), 3.54 (dd, *J* = 11.5, 3.6 Hz, 1H), 3.44 (d, *J* = 9.3 Hz, 2H), 3.37–3.31 (m, 3H), 3.23–3.14 (m, 3H), 3.11 (t, *J* = 10.9 Hz, 1H), 3.06 (s, 1H), 3.02 (d, *J* = 14.8 Hz, 1H), 2.51 (d, *J* = 9.1 Hz, 9H), 2.32 (s, 2H), 2.04 (dd, *J* = 13.5, 8.3 Hz, 1H), 1.45 (d, *J* = 6.9 Hz, 3H), 1.05–0.96 (m, 15H). ^13^C NMR (151 MHz, Chloroform-*d*) δ 173.39, 170.33, 169.78, 167.89, 157.97, 150.44, 148.55, 143.26, 131.63, 130.97, 129.64, 126.52, 70.02, 61.46, 59.13, 59.02, 58.37, 57.83, 56.57, 53.17, 50.44, 49.11, 48.93, 47.68, 43.97, 42.09, 38.00, 35.59, 34.92, 26.71, 26.59, 24.00, 22.40, 16.17. HRMS (ESI) *m*/*z*: calcd for C_41_H_61_ClN_8_O_8_S_2_, [M + Na], 915.3640; found, 915.3641.

4-(3-((3-(2-chloro-*N*-(1,1-dioxidotetrahydrothiophen-3-yl)acetamido)-2,2-dimethylpropyl)amino)-3-oxopropyl)-*N*-((*S*)-1-((2*S*,4*R*)-4-hydroxy-2-(((*S*)-1-(4-(4-methylthiazol-5-yl)phenyl)ethyl)carbamoyl)pyrrolidin-1-yl)-3,3-dimethyl-1-oxobutan-2-yl)piperidine-1-carboxamide (**PV21**). **PV21** (16% yield, white solid) was synthesized from **19k** according to **General Procedure 3**. ^1^H NMR (600 MHz, Chloroform-*d*) δ 8.65 (s, 1H), 7.63 (d, *J* = 8.0 Hz, 1H), 7.37–7.33 (m, 4H), 6.30 (t, *J* = 6.5 Hz, 1H), 5.05 (dq, *J* = 14.4, 7.9, 7.2 Hz, 2H), 4.68 (t, *J* = 8.1 Hz, 1H), 4.42 (s, 1H), 4.34 (d, *J* = 8.8 Hz, 1H), 4.09–4.01 (m, 2H), 3.86 (ddd, *J* = 42.9, 23.2, 10.4 Hz, 3H), 3.72–3.58 (m, 2H), 3.54 (dd, *J* = 11.4, 3.5 Hz, 1H), 3.29 (d, *J* = 15.8 Hz, 1H), 3.19 (d, *J* = 15.7 Hz, 1H), 3.16–3.06 (m, 3H), 3.00 (dt, *J* = 12.5, 5.8 Hz, 1H), 2.73–2.66 (m, 3H), 2.49 (s, 5H), 2.36 (dt, *J* = 8.8, 4.5 Hz, 1H), 2.18 (t, *J* = 7.9 Hz, 2H), 2.05–1.97 (m, 1H), 1.68–1.50 (m, 5H), 1.45 (d, *J* = 6.9 Hz, 3H), 1.42–1.40 (m, 1H), 1.22 (s, 1H), 1.01 (s, 9H), 0.95 (d, *J* = 5.3 Hz, 6H). ^13^C NMR (151 MHz, Chloroform-*d*) δ 173.72, 173.40, 170.03, 167.86, 157.75, 150.39, 148.41, 143.35, 131.60, 130.80, 129.52, 126.47, 69.91, 59.01, 58.76, 58.44, 57.62, 56.48, 50.48, 49.16, 48.84, 48.02, 44.26, 42.18, 37.93, 35.95, 35.33, 34.93, 33.40, 31.93, 31.54, 31.44, 26.65, 26.57, 23.90, 22.42, 16.11. HRMS (ESI) *m*/*z*: calcd for C_43_H_64_ClN_7_O_8_S_2_, [M + Na], 928.3844; found, 928.3846.

### 2.3. Protein Expression and Purification

A DNA fragment encoding full-length human PIN1 bearing an N-terminal cleavable His-tag and cloned into a pET28a vector was transformed into *Escherichia coli* BL21(DE3) cells. The transformed cells were grown in LB medium at 37 °C until the optical density at 600 nm (OD600) reached 0.6–0.8. Protein expression was induced with 0.2 mM isopropyl-β-D-thiogalactopyranoside (IPTG) and continued for 20 h at 16 °C. Cells were harvested by centrifugation at 5000 rpm for 15 min at 4 °C. The pellet was resuspended in lysis buffer consisting of phosphate-buffered saline (PBS) supplemented with 20 mM imidazole (pH 7.5) and 1 mM phenylmethylsulfonyl fluoride (PMSF). After sonication, the lysate was centrifuged at 20,000× *g* for 30 min. The supernatant was incubated with Ni-NTA resin (Smart-Lifesciences, Changzhou, China) for 3 h at 4 °C. Bound recombinant proteins were purified using the Ni-NTA system and eluted with a stepwise imidazole gradient (30, 50, 100, 150, 300, and 500 mM) in PBS, pH 7.5. The purified protein fraction was further subjected to gel-filtration chromatography (Cytiva Life Sciences, Wilmington, DE, USA). Fractions containing high-purity protein were pooled, concentrated to 30–40 mg/mL, aliquoted, and stored at −80 °C for subsequent use.

### 2.4. Immunoblot

Cells were seeded in 6-well plates at 70% confluence and then treated with different concentrations of compounds, ensuring a final DMSO concentration of 0.1%. After treatment, cells were placed on ice, washed once with ice-cold PBS, scraped, and centrifuged at 1000× *g* for 5 min to collect the pellet. Cell lysis was performed on ice using RIPA buffer (Beyotime, P0013D, Shanghai, China) with protease and phosphatase inhibitors (Bimake, B14002; Beyotime, P1082). After 10 min of incubation, the lysate was centrifuged at 15,000× *g* for 10 min to collect the supernatant. Protein concentration was determined using a BCA kit (Bioss, C05-02001, Woburn, MA, USA) and adjusted to 2 mg/mL with RIPA buffer. For SDS-PAGE, protein samples were mixed with 5× SDS loading buffer, heated at 95 °C for 5 min, and separated on a 10% polyacrylamide gel (Yeasen, 20325ES62, Shanghai, China) at 100 V for 100 min. Proteins were transferred onto a 0.2 μm nitrocellulose membrane (Pall, 66485, Port Washington, NY, USA) at 250 mA for 120 min. The membrane was blocked with 5% BSA in TBST for 1 h, then incubated overnight at 4 °C with primary antibodies (PIN1: CST, 3722S, Danvers, MA, USA; β-Actin: Santa Cruz, SC-47778, Dallas, TX, USA). After washing, the membrane was incubated with secondary antibodies (antimouse/-rabbit: Santa Cruz, SC-516132/SC-2357) for 2 h at room temperature. The membrane was washed and imaged using the ChemiScope fluorescence system (Clinx, Shanghai, China). For degradation assessment, DC_50_ and D_max_ values were obtained by densitometric analysis of immunoblot bands using ImageJ 1.54n. PIN1 signal intensities were normalized to β-actin and expressed relative to the DMSO-treated control. The resulting degradation–concentration data were fitted to a standard four-parameter logistic model using the equation: $Y=Bottom+\frac{\left(Top-Bottom\right)}{1+10^{(LogDC_{50}-X)\times HillSlope}}$, where *X* represents the compound concentration and *Y* denotes the relative PIN1 protein level. D_max_ was defined as the maximal extent of degradation and was derived from the *Bottom* parameter of the fitted curve.

### 2.5. Quantitative Proteomics

For TMT-based study, **PC2** (2 μM) or DMSO was added to MCF-7 cell culture medium and incubated for 12 h, with three biological replicates per group. Cells were harvested by centrifugation and washed twice with cold PBS. Whole-cell proteins were extracted by adding 300 µL of pre-cooled RIPA lysis buffer to the cell pellet, followed by 10 min of sonication on ice. The lysate was centrifuged, and the supernatant was collected and quantified using a BCA assay. Proteins were reduced with 10 mM TCEP, alkylated with 20 mM IAM, and precipitated overnight with cold acetone. The protein pellet was dried, re-dissolved, and digested with trypsin at 37 °C. The resulting peptides were labeled with TMT reagents, desalted using C18 columns, vacuum-dried, and analyzed by tandem mass spectrometry.

For DIA-based study, MCF-7 cells were treated with **PC2** (2 μM), **PC2-Neg** (2 μM), or DMSO for 12 h, with three biological replicates per group. Cells were collected, lysed with urea buffer containing PMSF, and sonicated on ice. Supernatants were obtained after centrifugation and quantified by BCA assay. Equal protein amounts were reduced with DTT, alkylated with IAM, and digested overnight with trypsin using the SP3 protocol. Peptides were desalted with C18 StageTips, vacuum-dried, and analyzed by DIA mass spectrometry. Protein identification and quantification were performed using Spectronaut with default parameters.

### 2.6. Microscale Thermophoresis Study

Recombinant PIN1 protein (200 nM, His-tag conjugated) was prepared in PBS with 0.05% Tween-20 and 1 mM DTT and labeled with the His-tag labeling dye (RED-tris-NTA MO-L018). **PC2** or sulfopin was serially diluted in PBS, and 10 µL of each concentration was mixed with 10 µL of 200 nM PIN1 protein and incubated at room temperature for 30 min. The mixture (10 µL) was loaded into a capillary, and measurements were performed on the Monolith NT.115 (NanoTemper Technologies, Inc., Munich, Germany) at 25 °C with 60% MST power. Data were analyzed using NanoTemper analysis software.

For ternary complex formation, **PC2** (2 µM) was preincubated with the labeled PIN1 protein for 1 h at room temperature. Recombinant CRBN protein (maximal concentration: 1 µM) was then serially diluted in the same buffer and mixed with an equal volume of the PIN1-**PC2** solution. As a control, an identical titration was performed in the absence of **PC2**. MST measurements were carried out under identical conditions as described above.

### 2.7. Molecular Modeling

The CRBN–thalidomide and PIN1–sulfopin complexes were retrieved from Protein Data Bank (4CI1 and 6VAJ, respectively) and prepared using Protein Preparation Wizard module. The 3D conformations of PROTAC compounds were built and minimized using LigPrep module. All structures were loaded into ICM-Pro and converted to ICM object files. The Monte-Carlo simulation thoroughness was adjusted for more extensive sampling by setting *effort* value to 3. Exposed side-chains within 10 Å from the binding region were sampled by setting *scsRad* value to 10. Docking results were analyzed using ICM-Pro and visualized in PyMOL 2.6.2.

### 2.8. Passive Cell Membrane Permeability

PAMPA assay was conducted to assess the passive membrane permeability of test compounds. Briefly, compounds were first prepared at a concentration of 10 µM in PBS (from 10 mM DMSO stocks). Lecithin was dissolved in dodecane and applied to the filter membrane separating the donor and acceptor compartments. In the donor compartment, 300 µL of the compound solution was added in triplicate, while the acceptor compartment received 5 µL of the lecithin/dodecane mixture followed by 300 µL of PBS (pH 7.4) within 10 min. The assembled PAMPA sandwich was incubated at 25 °C for 16 h to allow passive diffusion. At the end of the incubation, aliquots from both donor and acceptor compartments were collected and mixed with cold methanol containing an internal standard. Samples were centrifuged at 4 °C to remove precipitates, and the supernatants were then analyzed by LC-MS/MS to quantify compound concentrations and calculate permeability.

### 2.9. Cellular Assay

Generally, cells were seeded at a density of 200 cells per well in flat-bottomed 96-well cell culture plates, with each well containing 190 μL of culture medium. Following overnight incubation to allow for cell attachment, **PC2** was prepared in a threefold serial dilution, starting at a maximum concentration of 20 μM. The treated cells were incubated under standard culture conditions (37 °C, 5% CO_2_, and humidified atmosphere) for different time periods (12 h, 24 h, 48 h, 72 h, and 144 h). The antiproliferative effect of the compound was evaluated using the CellTiter-Glo^®^ (Promega Corporation, Madison, WI, USA) luminescent cell viability assay. Luminescence was measured using an EnSight™ multimode plate reader (PerkinElmer, Inc., Waltham, MA, USA). Dose–response curve was generated and fitted to provide GI_50_ values. All experiments were performed with three biological replicates (*n* = 3).

### 2.10. Antitumor Study in Xenograft Mice

All xenograft animal studies were conducted with approval from the Animal Research Ethics Committee of Chongqing Medical University (Approval number: IACUC-CQMU-2025-0458). Ten female nude mice bought from GemPharmatech (Chengdu, China) (BALB/c-Nude, 5 weeks old) were used to establish a human breast cancer xenograft model. One day prior to tumor cell inoculation, each mouse received a subcutaneous injection of 10 μg estradiol. On the following day, MCF-7 cells were mixed with 0.1 mL of matrix gel and injected into the right dorsal flank of the mice. Tumor growth, animal activity, physical condition, and weight were monitored throughout the experiment. Tumor growth was monitored by measuring the two vertical diameters every other day, with volume calculated using the formula 1/2 × *a* × *b*^2^, where *a* and *b* represent the long and short diameters in millimeter, respectively. The mice were randomly divided into treatment and control groups (*n* = 5 per group). When tumors reached 100 mm^3^, the treatment group was administered PC-2 (50 mg/kg, I.P.) and estradiol (10 μg, S.C.) every other day. At the end of the 6-week study, tumors were excised, weighed, and photographed for documentation.

### 2.11. Immunohistochemistry

Tumors from both the control and treatment groups were harvested, fixed in neutral buffered 10% formaldehyde at room temperature for 24 h, and then embedded in paraffin and sectioned. Immunohistochemical staining was performed using the universal two-step kit (mouse/rabbit enhanced polymer detection system). Sections were dewaxed, rehydrated, and treated with 3% hydrogen peroxide for 10 min to block endogenous peroxidase. Antigen retrieval was done in 0.01 mol/L sodium citrate buffer (pH 6.0) via microwave for 15 min. After blocking with 5% BSA for 20 min, sections were incubated overnight at 4 °C with anti-Ki67 antibody (Servicebio, GB121141-50, Wuhan, China), anticleaved-caspase3 antibody (Servicebio, GB11532-50), PIN1 antibody (CST, 3722s), anti-CD68 antibody (Servicebio, GB113109-10), anti-CD168 (Servicebio, GB115709-100) followed by incubation with goat antirabbit IgG at 37 °C for 60 min. DAB was used for color development, and sections were counterstained with hematoxylin, dehydrated, and mounted for microscopy.

### 2.12. Pharmacokinetics

PK evaluation was carried out by Shanghai Medicilon Inc. The in vivo pharmacokinetic experiment of **PC2** was performed in female Sprague-Dawley (SD) rats following intraperitoneal administration. A single dose of **PC2** (50 mg/kg) was administered, and venous blood plasma samples were collected at various time points (0.25, 0.5, 1, 2, 4, 8, and 24 h). For analysis, 30 µL of plasma was mixed with methanol containing the IS to precipitate proteins. The mixture was vortexed and centrifuged at 14,000 rpm for 7 min. The supernatant was collected and analyzed using an LC-MS/MS system to quantify **PC2** concentrations. Samples were assessed on a Sciex 5500 Triple Quad, using an ACQUITY UPLC BEH C18 column (1.7 µm, 2.1 × 50 mm), using abovementioned mobile phases and flow rate, with gradient program adjusted as follows: 10% B (0 min)—90% B (0.6 min)—90% B (1.1 min)—10% B (1.11 min)—10% B (1.4 min), with MS detection in positive ion ESI mode.

## 3. Results and Discussion

### 3.1. Design and Synthesis of PIN1-Targeting PROTACs

**Figure 2:**
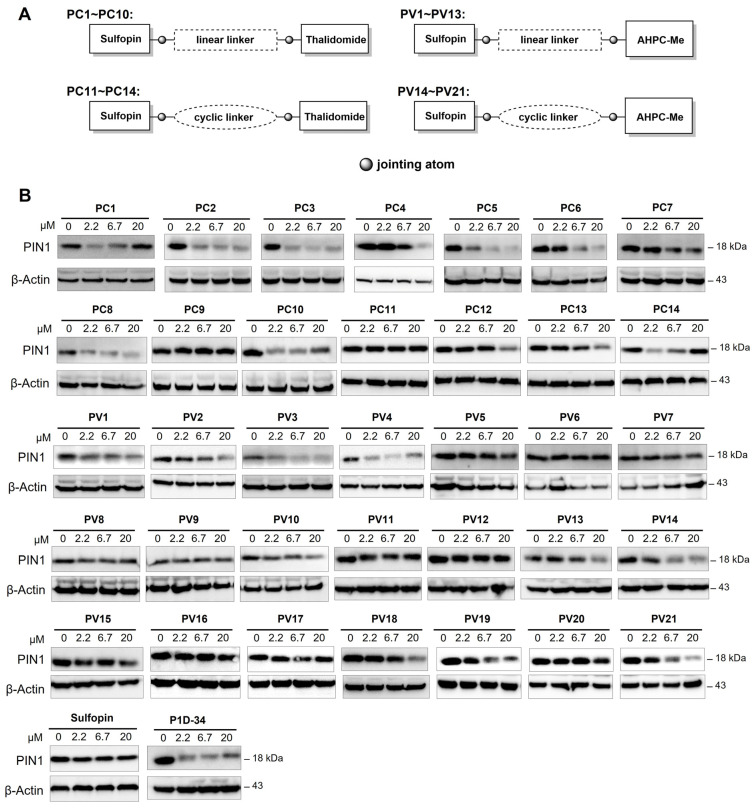
**PC** and **PV** series of PIN1-targeting PROTACs. (**A**) Systematic design of **PC** and **PV** compounds focusing on linker, jointing atom, and E3 ligase recruiter. (**B**) Preliminary degradation test of **PC** and **PV** compounds. MCF-7 cells were treated with DMSO or the given compound at concentrations of 2.2, 6.7, 20 μM for 12 h, and then analyzed by immunoblotting for PIN1 levels.

Given sulfopin’s potent PIN1-inhibition profile and antitumor effect, it was chosen as the PIN1-targeting binder for further development. As CRBN and VHL are among the most extensively characterized and widely utilized E3 ligases in targeted protein degradation, their respective ligands, thalidomide and AHPC-Me, were conjugated to sulfopin to generate two series of PROTACs, namely **PC** (PIN1-CRBN) and **PV** (PIN1-VHL). Accruing evidence underscores the critical role of linker in determining a PROTAC’s efficacy, specificity, and pharmacokinetic properties [26,27]. Accordingly, for both the **PC** and **PV** series, we also systematically evaluated a range of linker structures, including linear and cyclic designs, as well as varied atoms used for linker–ligand connection (referred to as “jointing atoms” in this study), to identify the most optimal compounds (Figure 2A). It is worth noting that Shi’s previous work synthesized a limited number of PIN1 PROTACs, primarily CRBN-recruiting PROTACs with long, linear linkers of at least eight atoms [23]. Instead, this study examined a wider variety of linker and E3 ligase combinations, aiming to provide a more systematic evaluation of PIN1 PROTAC design.

**Scheme 1:**
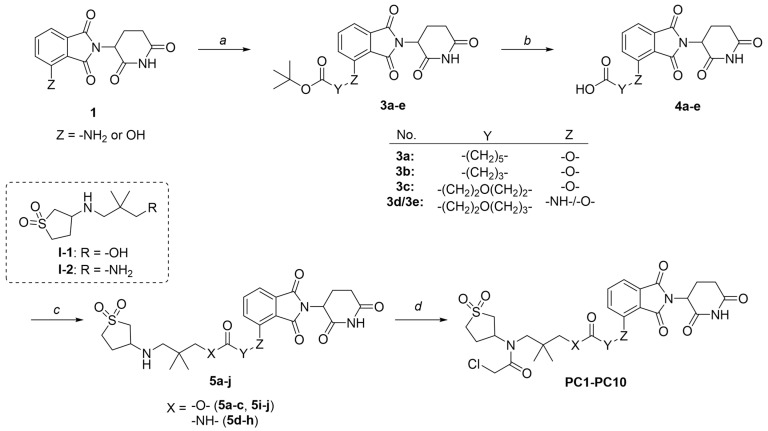
Synthesis of PC1-PC10.

**Scheme 2:**
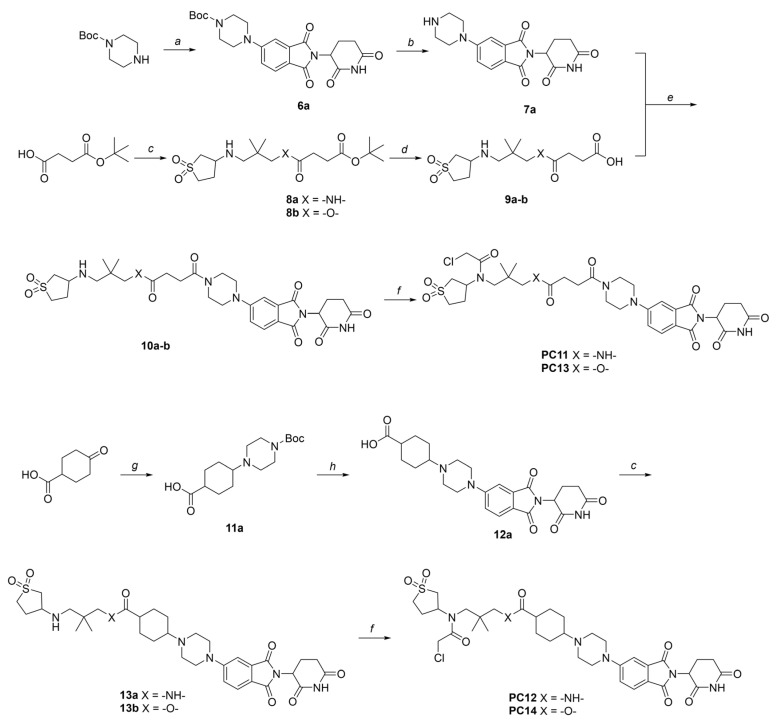
Synthesis of PC11-PC14.

The synthesis of linear-linker-based **PC** compounds (**PC1–PC10**) began with amino- or hydroxyl-substituted thalidomide **1**, which was modified with Boc-protected linkers **2a–d** (see Supplementary Information) to yield compounds **3a–e** (Scheme 1). Following Boc group deprotection, the resulting compounds **4a–e** were coupled with sulfopin intermediates **I-1** or **I-2** (Scheme S1). Finally, an electrophilic chloroacetyl warhead was installed to produce the target compounds. Cyclic-linker-based **PC** compounds (**PC11–PC14**) were prepared using slightly different routes (Scheme 2). 1-Boc-piperazine was attached to thalidomide through an S_N_Ar reaction and subsequently deprotected to yield amine **7a**. Acids **9a–b** were synthesized by coupling Boc-succinic acid with intermediates **I-1** or **I-2**, followed by removal of the *tert*-butyl group. Amide coupling between **7a** and **9a–b**, followed by warhead incorporation, resulted in **PC11** and **PC13**. Alternatively, 4-oxocyclohexanecarboxylic acid was converted to intermediate **11a** via Borch reduction with 1-Boc-piperazine. **PC12** and **PC14** were then synthesized in a similar manner.

**Scheme 3:**
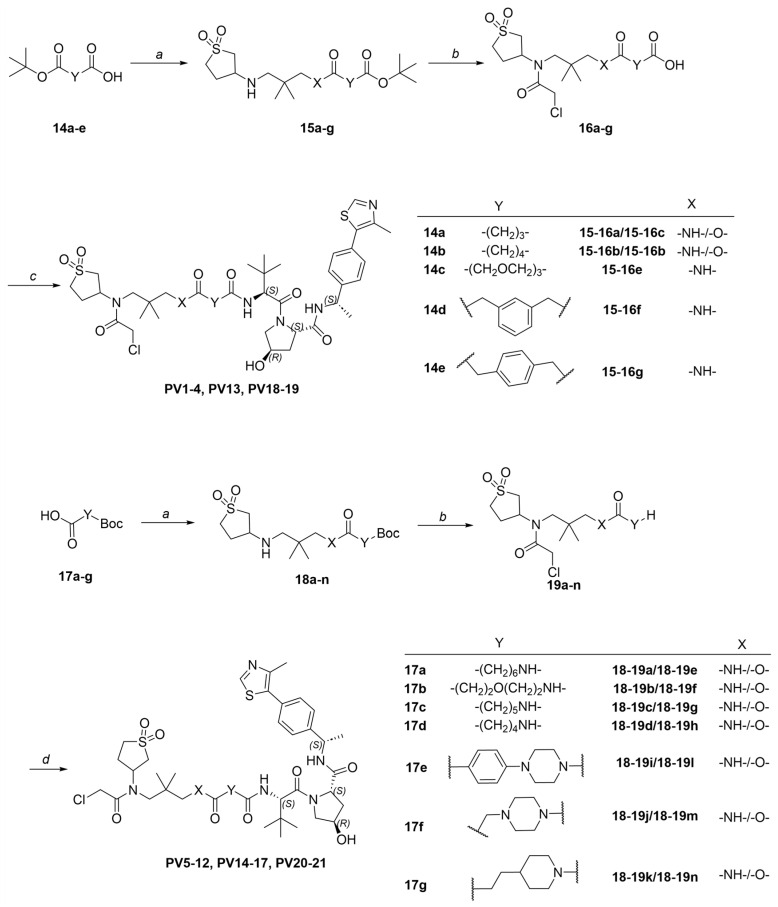
Synthesis of PV1-PV21.

In the **PV** series, two types of connection moieties with AHPC-Me are present: ureido and amide. Unlike the synthetic approach for **PC** compounds, *tert*-butyl-protected intermediates **14a–e** or Boc-protected intermediates **17a–d** were coupled with **I-1** or **I-2**, followed by immediate warhead installation to yield compounds **16a–g** and **19a–h**, respectively (Scheme 3). After deprotection of **16a–g**, AHPC-Me was coupled to the exposed acids via an HATU-promoted reaction, affording the amide compounds **PV1–4**, **PV13**, and **PV18–19**; while for **19a–h**, AHPC-Me was conjugated to their deprotected products using triphosgene, producing the ureido compounds **PV5–12**, **PV14–17**, and **PV20–21**.

Reagents and conditions: (*a*) **2a–d** (1.2 equiv), DIPEA (3.0 equiv), DMF, 70 °C, 20 h; (*b*) TFA/DCM (20% *v*/*v*), 0 °C to RT, 4 h; (*c*) **I-1** (1.0 equiv), DCC (1.5 equiv), DMAP (0.1 equiv), TEA (3.0 equiv), DMF, 0 °C to RT, overnight or **I-2** (1.0 equiv), HATU (1.5 equiv), DIPEA (3.0 equiv), DMF, 0 °C to RT, overnight; (*d*) chloroacetyl chloride (1.5 equiv), DIPEA (3.0 equiv), DCM, 0 °C to RT, 2 h.

Reagents and conditions: (*a*) thalidomide 5-fluoride (1.0 equiv), DIPEA (1.1 equiv), DMSO, 90 °C, 14 h; (*b*) 4M HCl in dioxane (20 equiv), DCM, 0 °C to RT, 2 h; (*c*) **I-1** (1.0 equiv), DCC (1.5 equiv), DMAP (0.1 equiv), TEA (3.0 equiv), DMF, 0 °C to RT, overnight or **I-2** (1.0 equiv), HATU (1.5 equiv), DIPEA (3 equiv), DMF, 0 °C to RT, overnight; (*d*) TFA/DCM (20% *v*/*v*), 0 °C to RT, 4 h; (*e*) HATU (1.5 equiv), DIPEA (3.0 equiv), DMF, 0 °C to RT, overnight; (*f*) chloroacetyl chloride (1.5 equiv), DIPEA (3.0 equiv), DCM, 0 °C to RT, 2 h; (*g*) 1-Boc-piperazine (1.0 equiv), NaBH(OAc)_3_ (1.2 equiv), AcOH/DCE (1/10, *v*/*v*), RT, 2 h; (*h*) (i) TFA (20 equiv), DCM, 0 °C to RT, 3 h; (ii) thalidomide 5-fluoride (1.0 equiv), DIPEA (3.0 equiv), DMSO, 90 °C, 10 h.

Reagents and conditions: (*a*) **I-1** (1.0 equiv), DCC (1.5 equiv), DMAP (0.1 equiv), TEA (3.0 equiv), DMF, 0 °C to RT, overnight; or **I-2** (1.0 equiv), HATU (1.5 equiv), DIPEA (3.0 equiv), DMF, 0 °C to RT, overnight; (*b*) (i) chloroacetyl chloride (1.5 equiv), DIPEA (3.0 equiv), DCM, 0 °C to RT, 2 h; (ii) TFA/DCM (20% *v*/*v*), 0 °C to RT, 4 h; (*c*) (*S*, *R*, *S*)-AHPC-Me·HCl (1.0 equiv), HATU (1.5 equiv), DIPEA (3.0 equiv), DMF, 0 °C to RT, overnight; (*d*) BTC (0.3 equiv), DIPEA (9.0 equiv), (*S*, *R*, *S*)-AHPC-Me·HCl (1.0 equiv), DCM, RT, 2–8 h.

### 3.2. Structure-Activity Relationships of** *PC*** and ***PV*** Series PROTACs

In total, 14 **PC** compounds and 21 **PV** compounds were synthesized and evaluated for their ability to degrade intracellular PIN1 protein using Western blot analyses. Initial screening assessed both series of compounds in two cancer cell lines, MCF-7 (breast cancer) and PATU-8988T (pancreatic adenocarcinoma), at three concentrations, with a maximum concentration of 20 µM (Figure 2B and Figure S1). Sulfopin alone had negligible impact on intracellular PIN1 levels, confirming the specificity of the degradation observed with PROTAC compounds. Notably, some compounds (e.g., **PC1**, **PC10**, **PC14**, **PV4**) displayed characteristic hook effect at high concentration. Overall, both **PC** and **PV** compounds exhibited stronger PIN1-degrading activity in MCF-7 cells than in PATU-8988T cells, suggesting that MCF-7 cells are more sensitive to PIN1-targeted strategies. Consequently, subsequent experiments primarily focused on MCF-7 cells.

To generate robust SAR data, we next conducted concentration-dependent degradation assays in triplicate for all compounds using Western blotting in MCF-7 cells (Table 1 and Table 2, Figure S2). Among linear **PC** compounds, the observed SAR reflects a combined influence of both linker length and the chemical nature of the jointing atoms, with the latter exerting a more decisive impact on activity. Specifically, **PC2** and **PC3**, which employed oxygen as the jointing atom on the sulfopin side (X = O), outperformed corresponding **PC5** and **PC6** which have nitrogen in this position (X = NH). A similar enhancement was observed for **PC10**, which contains two oxygen linkers (X, Z = O) and exhibited markedly improved activity compared with the other long-chain PROTACs **PC7–PC9**. These findings align with Klein et al.’s conclusion that amide-to-ester bioisosteric substitution at the joint region of a PROTAC could enhance potency primarily by reducing hydrogen bond donors and improving membrane permeability [28]. To further validate this, we measured passive permeability using PAMPA (Table S1) and found that **PC1** and **PC2** showed acceptable permeability (−Log P_e_ = 5.62 and 6.33, respectively), whereas **PC4** and **PC5** were substantially less permeable (−Log P_e_ > 9.06 and >9.12, respectively), mirroring their lower cellular activity.

Although the effect of linker length appeared less pronounced, a mild trend was still evident. The shorter linkers (**PC1–PC6**) tended to induce greater degradation than their longer-chain counterparts (**PC7–PC9**). Notably, the control compound **P1D-34**, featuring the longest chain (16-atom), apparently exhibited reduced potency in our study (DC_50_ = 0.85 ± 0.27 μM) versus Shi et al.’s report in AML cells (DC_50_ = 0.18 μM) [23], further suggesting that the activity of the PIN1 PROTAC may be cell-type dependent.

Within the cyclic **PC**-series, **PC14** achieved sub-micromolar potency but only induced partial degradation. The reduced activity may stem from the rigidity of the ring structures, which could impair the plasticity required for optimal interaction between the PIN1 and CRBN interfaces. Despite the limited activity of cyclic compounds, Klein et al.’s amide-to-ester substitution strategy remains practical, with **PC13** and **PC14** outperforming **PC11** and **PC12**, respectively.

**Figure 3:**
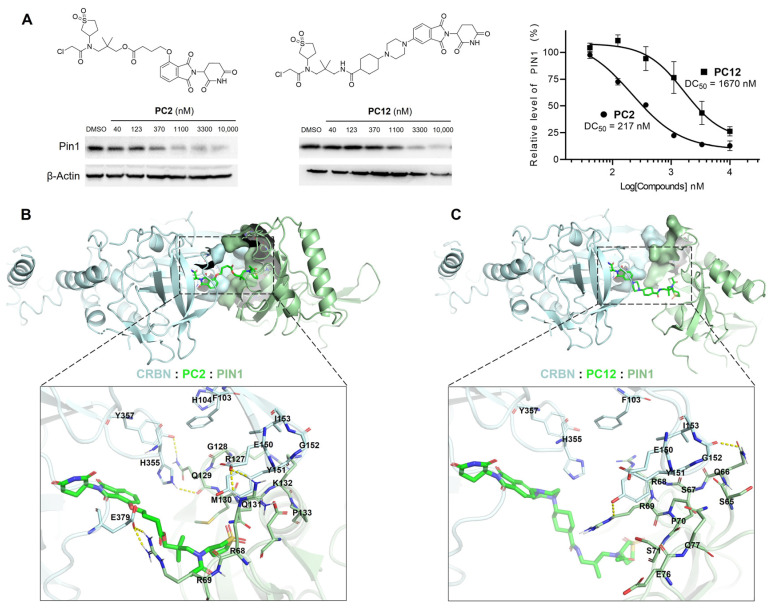
Molecular modeling result. (**A**) Structures and degradative activities of **PC2** (bearing short linear linker) and **PC12** (bearing long cyclic linker). (**B**,**C**) Top-ranked poses of PIN1 and CRBN complexed with **PC2** or **PC12**. In the zoomed-in view, PIN1-CRBN interactions were shown as dashed lines. The 3D structures used for docking were obtained from the Protein Data Bank (PDB ID: 4CI1 for CRBN, and 6VAJ for PIN1).

To gain a deeper insight into the SAR analysis at the molecular level, we performed computational modeling to study the ternary complex formation for **PC2** (short linear linker) and **PC12** (long cyclic linker) (Figure 3A). Using the ICM-PROTAC module, we modeled the PIN1–sulfopin and CRBN–thalidomide complexes onto the PCs to form the ternary complex, followed by Monte Carlo simulations with flexible linkers and surrounding residues to generate and rank configurations by energy. In total, 26 effective poses were generated for **PC2**, with the top-scoring pose exhibiting an energy of −16.97 kcal/mol (Figure 3B), whereas only one effective pose was generated for **PC12**, with an energy of −7.53 kcal/mol (Figure 3C). This result suggests a more productive ternary complex formation induced by **PC2** compared to **PC12**, in line with their difference in potency. The top pose of **PC2** induced a well-defined interaction network between PIN1 and CRBN (Table S2), with an extensive contacting surface area of 2282 Å^2^. In comparison, only limited protein–protein interactions were observed for **PC12**’s pose, resulting in a smaller contacting surface area of 1881 Å^2^. This discrepancy implies that the increased flexibility of and the spatial arrangement of the shorter linker in **PC2** could lead to a more optimal alignment of the binding sites, as opposed to the steric constraints imposed by the longer, cyclic linker of **PC12**.

Given the lack of reported VHL-recruiting PIN1 degraders, we initially aimed to explore the **PV**-series compounds more extensively. However, **PV**s on the whole exhibited inferior degradation activity compared to **PC**s in both MCF-7 and PATU-8988T cell lines (Figure S1), regardless of linker type. Hence, this underperformance likely stems from VHL-related biological factors rather than solely from structural limitations of the PROTACs. As a tumor suppressor, VHL is frequently downregulated in cancers, partly through a PIN1-promoted mechanism [29]. Consistently, Western blot analysis revealed reduced VHL protein levels in MCF-7, MV4-11, MOLM-13, and THP1 cells compared with HEK293T (Figure S3A), which may account for the low efficiency of **PV**s. Despite their relatively low potency, **PV**s follow a similar SAR trend as **PC**s (Table 2): shorter linear linkers and minimal use of secondary amine as jointing atom are generally beneficial to degradative activity. The short-linker-based, ester-linked **PV3** (DC_50_ = 1.50 μM, D_max_ = 73%) and **PV4** (DC_50_ = 2.43 μM, D_max_ = 72%) stand as the most potent compounds in this series. In contrast, **PV5–PV12**, bearing a ureido linkage at the VHL end, were largely inactive, likely due to the presence of an additional hydrogen bond donor. This is further evidenced by PAMPA results (Table S1), where **PV4** (−LogP_e_ = 6.62) exhibited better permeability than **PV8** (−LogP_e_ > 9.29). A long PEG linker (**PV13**) did not bring about any advantage. As for cyclic **PV**s, only **PV15**, **PV18**, **PV19**, and **PV21** showed modest degradation which was insufficient to warrant further investigation.

### 3.3. Characterization of** *PC2*** as the Lead Compound and Its Mechanism of Action

**Figure 4:**
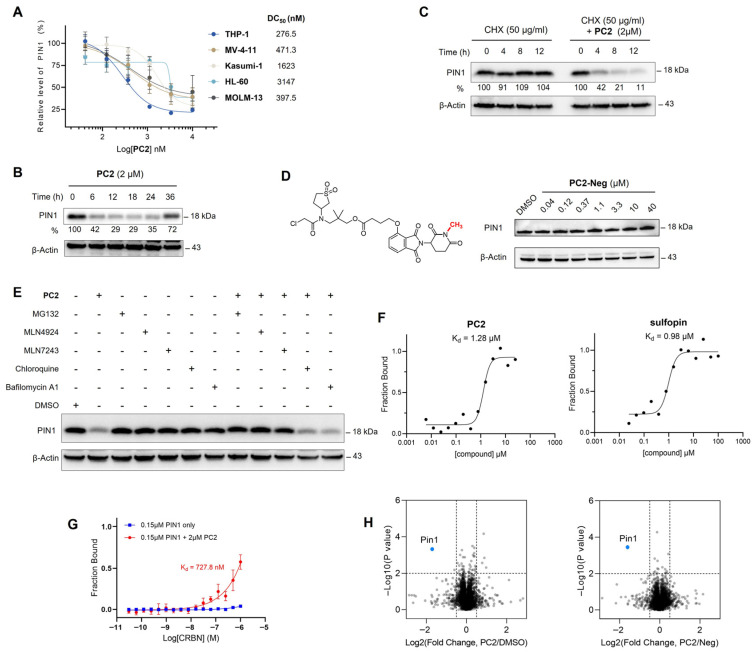
In-depth characterization and mechanistic studies of **PC2**. (**A**) DC_50_ determination of **PC2** in a series of AML cell lines by immunoblot following 12 h treatment with increasing concentrations of the compound (0, 0.04, 0.123, 0.37, 1.1, 3.3, and 10 μM). (**B**) Immunoblot for PIN1 in MCF-7 cells treated with 2 μM of **PC2** for indicated time points. (**C**) Time course of PIN1 degradation by 2 μM of **PC2** in presence of cycloheximide (50 μg/mL). (**D**) Chemical structure and degradative effect of **PC2-Neg** after 12 h treatment. (**E**) Immunoblot for PIN1 in MCF-7 cells pre-treated for 30 min with DMSO, MG132 (10 µM), MLN4924 (10 µM), MLN7243 (0.5 µM), chloroquine (10 µM), or bafilomycin A1 (1 µM), followed by treatment with 2 µM **PC2** or vehicle control. (**F**) MST binding curves of **PC2** and sulfopin to recombinant PIN1 protein (0.1 μM). (**G**) MST titration curves of CRBN to recombinant PIN1 protein in presence or absence of **PC2**. (**H**) DIA-based proteomic profiling of **PC2** and **PC2-Neg** in MCF-7 cells (2 μM of compounds, 12 h). Experiments for (**A**,**B**,**D**,**G**,**H**) were performed in triplicate.

Since foregoing results have demonstrated a promising profile of **PC2**, we further assessed its degradative activity across a series of leukemia cell lines (Figure 4A and Figure S3B), where the control compound **P1D-34** had been initially tested. **PC2** consistently induced degradation of PIN1, particularly in THP-1, MOLM-13, and MV4-11 cells, with corresponding DC_50_ values below 500 nM, suggesting its potential for broader applications.

To characterize the degradation kinetics of **PC2**, we first monitored the levels of PIN1 at various time points following treatment (Figure 4B). Noticeable degradation of PIN1 was observed within 6 h of cellular incubation with **PC2** (2 μM), with the most substantial reduction occurring between 12 and 18 h. Beyond this period, PIN1 levels began to recover, likely due to cellular protein resynthesis and potential drug clearance. By 36 h, a distinct band indicating the re-emergence of PIN1 was observed.

To exclude the influence of protein resynthesis, which could confound the observed degradation kinetics, we incorporated cycloheximide (CHX) into the study (Figure 4C). By inhibiting de novo protein synthesis, CHX allowed us to isolate the effect of **PC2** on pre-existing PIN1. Treatment with CHX alone did not affect intracellular PIN1 levels over 12 h. However, co-treatment with CHX and **PC2** led to more rapid and pronounced degradation, with PIN1 levels reduced by 89% at the 12 h mark. Apparently, **PC2**-mediated degradation primarily impacts the existing pool of PIN1.

To confirm that **PC2**’s activity is CRBN-dependent, we synthesized a CRBN-binding-deficient analog, **PC2-Neg** (Scheme S4). In dose-dependent degradation assay, PIN1 levels remained unchanged following treatment with **PC2-Neg** at concentrations up to 40 μM (Figure 4D), reinforcing the requirement for CRBN engagement in **PC2**-mediated degradation.

To dissect the molecular pathway underlying **PC2**-induced degradation, we utilized a panel of inhibitors targeting key components of the UPS and autophagy–lysosomal pathway (Figure 4E). MG132, a proteasome inhibitor, completely abrogated PIN1 degradation, indicating that proteasomal activity is essential for **PC2** function. Similarly, MLN4924, which inhibits NEDD8-activating enzyme and blocks Cullin-RING ligase activity, and MLN7243, a ubiquitin-activating enzyme inhibitor, both suppressed degradation. In contrast, chloroquine and bafilomycin A1, two inhibitors of lysosomal activity with distinct mechanisms of action, had no effect on **PC2** activity, indicating that **PC2**-mediated degradation is independent of ALP. These findings collectively reinforced **PC2**’s reliance on the UPS.

To systematically evaluate the specificity of **PC2** for PIN1, we first verified its direct binding to recombinant PIN1 protein using microscale thermophoresis (Figure 4F). The binding affinity of **PC2** and sulfopin was found to be comparable, confirming that the incorporation of a short linker and thalidomide did not significantly alter its interaction with PIN1. We next examined ternary complex formation by titrating CRBN with PIN1 in the presence or absence of **PC2** (Figure 4G). While PIN1 alone showed no detectable binding to CRBN, the addition of **PC2** induced stable ternary complex formation with an apparent dissociation constant (*K*_d_) of 727 nM, indicating that **PC2** promotes CRBN association with a PIN1-containing complex, consistent with formation of ternary complex.

To assess **PC2**’s specificity in degrading PIN1 within a cellular environment, we performed both DIA- and TMT-based quantitative proteomics studies (Figure 4H and Figure S4). To our delight, PIN1 was the most significantly and selectively reduced protein upon **PC2** treatment compared with either **PC2-Neg** or DMSO controls, underscoring the exceptional specificity of **PC2** for its target at the protein level. We further conducted RNAseq to examine whether **PC2** treatment would induce widespread changes in the cellular transcriptome. The results showed minimal perturbation, with only 15 genes upregulated and 12 genes downregulated (Figure S5). Overall, these findings support the targeted action of **PC2** on PIN1 with minimal disruption to cellular homeostasis and highlight it as a promising candidate for further antitumor studies.

### 3.4. In Vitro and In Vivo Evaluation of** *PC2***’s Antitumor Efficacy and Pharmacokinetics

As mentioned in the Introduction, two prior studies left a question mark over the therapeutic potential of PIN1-targeting PROTACs due to their differing antiproliferative effects in cancer cells, despite both achieving strong PIN1 degradation. Shi et al. demonstrated that **P1D-34** significantly reduced the survival of multiple AML cell lines and attributed this activity to efficient PIN1 degradation, with a GI_50_/DC_50_ ratio of 12.7-fold in MV4-11 cells. In contrast, Liu et al. reported minimal antiproliferative effects for their lead PROTAC **D4**, with a GI_50_/DC_50_ ratio exceeding 1600-fold in various cancer cell lines including MDA-MB-468, PANC-1, and Huh-7.

**Figure 5:**
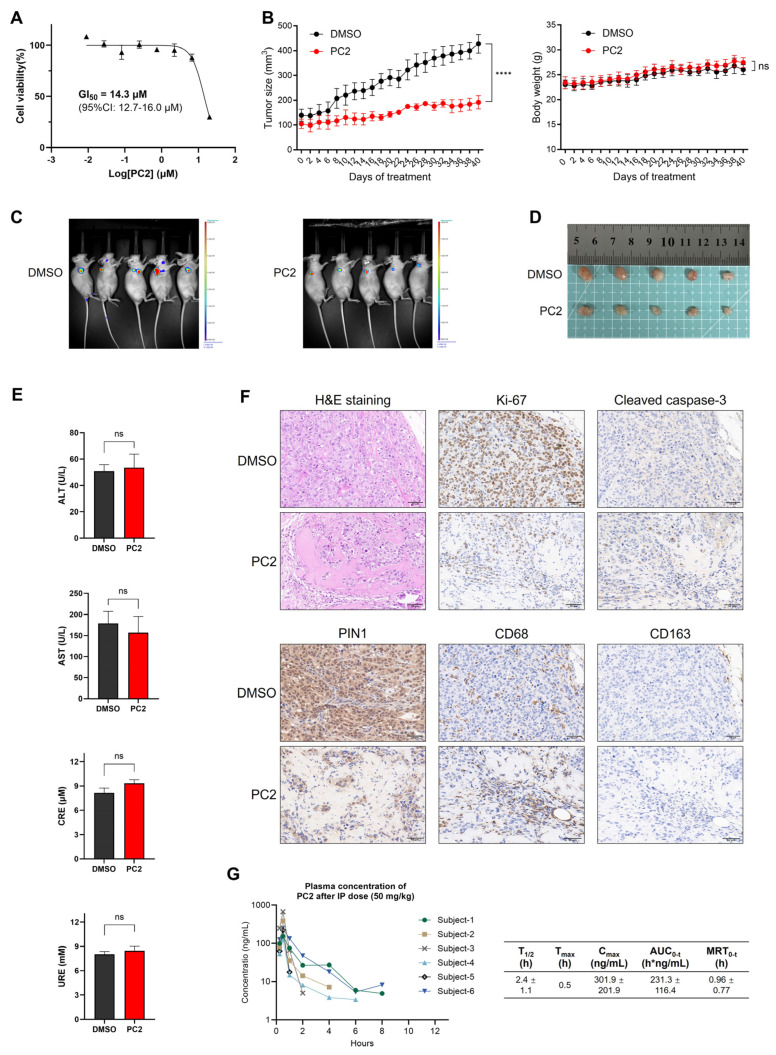
Antitumor effect of **PC2**. (**A**) MCF-7 cell viability after **PC2** treatment for 6 days. (**B**) Tumor volume and body-weight change in female mice administered with DMSO or **PC2** (50 mg/kg, QOD, IP), *n* = 5, SEM indicated. (**** *p* < 0.0001) (**C**) In vivo imaging of EGFP-tagged xenograft tumors. (**D**) Tumor mass excised from xenograft mice postmortem. (**E**) Comparison of clinical markers in mice plasma. (**F**) Histopathological analysis of tumor tissue sections. (**G**) The in vivo pharmacokinetic evaluation of **PC2** (50 mg/kg, IP administration, *n* = 6).

To examine whether **PC2**’s degradative potency translates into antitumor efficacy, we first assessed its antiproliferative activity in MCF-7 cells. **PC2** showed minimal impact on cell viability over the initial 2 days of treatment, with moderate growth inhibition observed after prolonged exposure for 3–6 days (Figure 5A and Figure S6A), yielding a GI_50_ value of 14.3 μM. This effect remained notably weaker than its degradation potency, reflecting a 66-fold discrepancy. The differences in GI_50_/DC_50_ ratio across present and previous studies may suggest varying sensitivity of cell lines to PIN1 degradation. The delayed response likely reflects PIN1’s role as a signaling hub regulating multiple oncogenic and tumor-suppressive pathways, where depletion requires extended time to produce measurable phenotypic effects. In addition, **PC2** displayed moderate antiproliferative activity in THP-1 (GI_50_ = 9.65 μM) and MV4-11 (GI_50_ = 8.98 μM) cells (Figure S6B), consistent with its degradation efficiency in these lines, whereas it showed negligible cytotoxicity in HL-60 cells (GI_50_ > 50 μM) where degradation was also suboptimal. Collectively, these findings suggest that PIN1-specific degradation exerts a limited but on-target effect on cancer cell growth in vitro. It is important to note, though, that such modest cellular effects have been repeatedly observed for PIN1-targeting agents and do not preclude meaningful therapeutic potential. For instance, sulfopin was reported to elicit only limited antiproliferative activity in two-dimensional cancer cell cultures (est. GI_50_~20 μM) even after prolonged exposure, yet it produced substantial antitumor efficacy across multiple animal models including neuroblastoma and pancreatic cancer [6,19].

Consequently, in spite of the mild cellular activity of **PC2**, we moved on to explore its therapeutic potential in vivo, which had not been investigated for **P1D-34** or **D4**. An MCF-7 xenograft model was established in female nude mice, with **PC2** administered intraperitoneally at 50 mg/kg every two days (QOD) for up to six weeks (Figure 5B–D). We were pleased to see a significant suppression of tumor growth in the **PC2**-treated group compared to the control group (DMSO only), with neither noticeable difference in body weight nor overt adverse effect. Interestingly, relative tumor volumes in both groups remained nearly identical during the first six days but began to diverge thereafter (Figure S7), coinciding with the onset of **PC2**’s antiproliferative effect in MCF-7 cells and suggesting a time-dependent on-target response. Additionally, clinical markers sampled from mice plasma including ALT (alanine aminotransferase), AST (aspartate aminotransferase), CRE (creatinine), and URE (urea), remained stable (Figure 5E), suggesting that **PC2** has a favorable safety profile, particularly in terms of liver and kidney function. It is noteworthy that, compared to the benchmark sulfopin (40 mg/kg QD/BID; MW = 282 g/mol), **PC2** demonstrated significant tumor suppression in mouse models at 50 mg/kg QOD despite its higher molecular weight (640 g/mol). This lower molar dose requirement, combined with less frequent administration, indicates superior molar efficacy, a more sustained pharmacodynamic effect, and a potentially improved safety profile for **PC2**.

Next, histopathological studies were performed on tumor samples from both groups. H&E stains revealed increased stromal content and reduced atypia in the **PC2**-treated group compared to the control group (Figure 5F). Tumor tissues were further subjected to staining for Ki-67 to assess proliferation, cleaved caspase-3 to evaluate apoptosis, PIN1 protein levels, and the macrophage markers CD68 as well as CD163. Consistent with the suppressed tumor growth, fewer Ki-67-positive cells and more cleaved-caspase-3-positive cells were observed in the **PC2**-treated group. Importantly, PIN1 staining was markedly reduced in **PC2**-treated mice compared with DMSO controls, demonstrating that **PC2** achieves sufficient intratumoral exposure to engage and degrade its target in vivo. Interestingly, the number of CD68+ macrophages was significantly higher in the **PC2**-treated group, while minimal infiltration of CD163+ macrophages was observed in both groups. This finding implies that **PC2** may be capable of modulating the immune microenvironment to promote antitumor immunity, likely by enhancing macrophage activation or inducing a shift toward M1 polarization. Interestingly, PIN1 inhibition has also been reported to remodel the desmoplastic and immunosuppressive tumor microenvironment by acting on cancer-associated fibroblasts [6]. Although the underlying mechanisms may differ, both their findings and ours represent indirect consequences of PIN1 targeting that converge on TME modulation rather than solely a direct cytotoxic effect. This additional layer of PIN1 biology further underscores that the modest in vitro potency of **PC2** does not necessarily diminish its therapeutic relevance.

Encouraged by these promising results, we proceeded to conduct preliminary in vivo pharmacokinetic (PK) study of **PC2** with intraperitoneal administration (Figure 5G, Table S3) in female mice. The results revealed a modest half-life of 2.4 h for **PC2**, with a maximal concentration (C_max_) of 301.9 ng/mL observed at 0.5 h post-injection. This exposure exceeds its DC_50_ by approximately two-fold and is sufficient to ensure effective PIN1 engagement and degradation in tumors. The mean residence time (MRT) was approximately 1 h. These pharmacokinetic findings rendered **PC2** a promising lead for future development. Next-stage structural optimization will focus on its potential metabolic soft spots (Figure S8), and further studies are warranted to explore its long-term efficacy, tissue distribution, and in vivo mode of actions.

## 4. Conclusions

In this study, we designed and synthesized two series of PROTACs targeting PIN1, a critical regulator in cancer progression. These compounds utilized sulfopin as the PIN1 binder and incorporated ligands for CRBN (**PC** series) and VHL (**PV** series). Through a comprehensive evaluation of linker structures and jointing atoms, we established robust SAR for PIN1-targeting PROTACs: (1) short, linear linkers could enhance degradation efficacy by facilitating favorable ternary complex formation, and (2) fewer secondary amines at the joint likely benefit the potency by improving cell permeability. Notably, the overall lower potency of the **PV** series indicates VHL is not a preferred E3 ligase for targeting PIN1, likely due to reduced VHL activity in cancer cells. However, the established SAR principles remained applicable to the **PV** series.

Our focused SAR study identified **PC2** as a lead compound, outperforming the previously reported PROTAC **P1D-34** (DC_50_ = 0.85 μM) in MCF-7 cells. Mechanistic studies confirmed that **PC2** induces proteasome-dependent PIN1 degradation with high selectivity and minimal off-target effects on the cellular proteome and transcriptome. While **PC2** displayed modest antiproliferative activity in vitro, it showed pronounced efficacy in vivo. In a mouse xenograft model, **PC2** significantly suppressed tumor growth without observable toxicity, which has not yet been demonstrated for existing PIN1 PROTACs. Histopathological analysis suggested that, in addition to degradation of PIN1 in tumor cells, **PC2** might additionally modulate the tumor immune microenvironment to boost its antitumor efficacy. These results may help address ongoing debate regarding the therapeutic relevance of PIN1 degradation, recapitulating the limitations of relying solely on cell viability assays to predict in vivo outcomes. Although the metabolic properties of **PC2** remain to be improved, our pharmacokinetic studies provide valuable insights for subsequent structural optimization. Notably, compared to the covalent inhibitor sulfopin, **PC2** achieved tumor growth suppression at a lower molar dose and with a less frequent dosing schedule. Together, these findings support further development of **PC2** as a promising candidate for PIN1-directed anticancer therapy.

## Figures and Tables

**Table 1:** Structures and degradative activities of **PC** compounds.

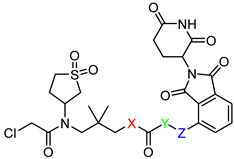			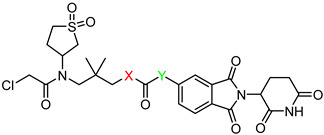		
Structure I			Structure II		
Compound	X	Y	Z	DC_50_ (μM)	D_max_
**P1D-34**	Structure shown in Figure 1			0.85 ± 0.27	87%
**Structure I (PC1–PC10)**					
**PC1**	O	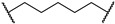	O	1.19 ± 0.36	65%
**PC2**	O	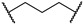	O	0.22 ± 0.06	91%
**PC3**	O	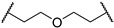	O	0.64 ± 0.11	78%
**PC4**	NH	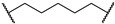	O	1.24 ± 0.25	85%
**PC5**	NH	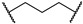	O	1.78 ± 0.75	59%
**PC6**	NH	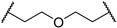	O	3.09 ± 1.17	54%
**PC7**	NH	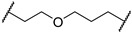	NH	N.A.	40%
**PC8**	NH	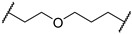	O	N.A.	47%
**PC9**	O	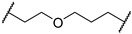	NH	N.A.	11%
**PC10**	O	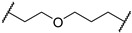	O	0.66 ± 0.21	84%
**Structure II (PC11–PC14)**					
**PC11**	NH	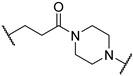	-	N.A.	<10%
**PC12**	NH	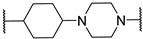	-	1.67 ± 0.81	73%
**PC13**	O	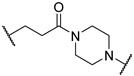	-	N.A.	68%
**PC14**	O	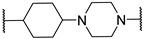	-	0.38 ± 0.18	73%

MCF-7 cells treated with 0, 0.04, 0.123, 0.37, 1.1, 3.3, 10 μM of PCs for 12 h and immunoblotted; N.A., No Activity or DC_50_ > 10 μM; Data were obtained from triplicate experiments and displayed as means + SEM.

**Table 2:** Structures and degradative activities of **PV** compounds.

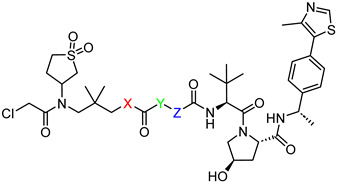					
Compound	X	Y	Z	DC_50_ (μM)	D_max_
**PV1**	NH	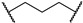	-	N.A.	<10%
**PV2**	NH	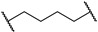	-	N.A.	25%
**PV3**	O	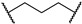	-	1.50 ± 0.27	73%
**PV4**	O	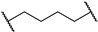	-	2.43 ± 0.99	72%
**PV5**	NH	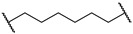	NH	N.A.	<10%
**PV6**	NH	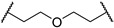	NH	N.A.	<10%
**PV7**	NH	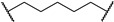	NH	N.A.	30%
**PV8**	NH	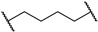	NH	N.A.	39%
**PV9**	O	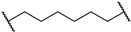	NH	N.A.	<10%
**PV10**	O	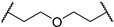	NH	N.A.	47%
**PV11**	O	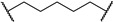	NH	N.A.	29%
**PV12**	O	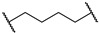	NH	N.A.	<10%
**PV13**	NH	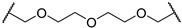	-	N.A.	50%
**PV14**	NH	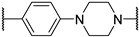	-	N.A.	58%
**PV15**	O	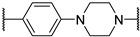	-	1.69 ± 0.84	57%
**PV16**	O	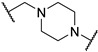	-	N.A.	<10%
**PV17**	O	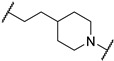	-	N.A.	11%
**PV18**	NH	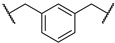	-	1.53 ± 0.55	58%
**PV19**	NH	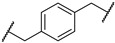	-	1.67 ± 0.53	71%
**PV20**	NH	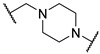	-	N.A.	<10%
**PV21**	NH	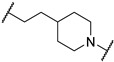	-	2.49 ± 1.08	52%

MCF-7 cells treated with 0, 0.04, 0.123, 0.37, 1.1, 3.3, 10 μM of PVs for 12 h and immunoblotted; N.A., No Activity or DC_50_ > 10 μM; Data were obtained from triplicate experiments and displayed as means + SEM.

## Data Availability

The original contributions presented in this study are included in the article/Supplementary Material (Table S4, Files S1–S4). Further inquiries can be directed to the corresponding authors.

## Supplementary Materials

The following supporting information can be downloaded at: https://www.mdpi.com/article/10.3390/pharmaceutics18030288/s1, Figure S1: Preliminary degradation test of PC and PV compounds in PATU-8988T cells; Figure S2: Concentration-dependent degradation test of PC and PV compounds in MCF-7 cells. (**A**) Repre-sentative immunoblots; (**B**) Concentration-response curves (*n* = 3); Figure S3: Immunoblots for (**A**) VHL protein levels in different cell lines, and (**B**) concentration-dependent experiments of PC2 in a series of AML cell lines (*n* = 3); Figure S4: (**A**) Immunoblots of samples for proteomic study. (**B**) TMT-based proteomic profiling of PC2 (2μM) treatment in MCF-7 cells. (**C**) Violin plot for PIN1 (Uniprot ID: Q13526) in the TMT-based proteomic study; Figure S5: Volcano plot of RNA-seq data; Figure S6: Antiproliferative effect of PC2 (**A**) at different treatment durations in MCF-7 cells; (**B**) at the sixth day in different AML cell lines; Figure S7: Relative tumor volume plot; Figure S8: Predicted sites of metabolism and transformations of PC2 by three computational tools; Table S1: Permeability assay by PAMPA method*^a.^*; Table S2: Interacting residues of PIN1 and CRBN in models of ternary complexes; Table S3: Individual and mean plasma concentration-time data of PC2 after and IP dose (ng/mL); Table S4: RNAseq differential expression data; File S1: 1H and 13C NMR spectra; File S2: Synthetic procedures for intermediates and PC2-Neg; File S3: HPLC traces; File S4: HRMS data.
