# Supplementary material for: Systematic Optimization of Proteolysis-Targeting Chimeras for PIN1 Enables Selective Degradation and Antitumor Activity In Vivo
Source: Pharmaceutics. 2026 Feb 26;18(3):288. doi: 10.3390/pharmaceutics18030288 (PMC13029591; doi:10.3390/pharmaceutics18030288)
Supplement: Supplementary file 1 [file pharmaceutics-18-00288-s001.zip › pharmaceutics-Supplementary figures and tables.pdf]

pharmaceutics

MDPI

Supplementary Materials: Systematic Optimization of Proteolysis-Targeting Chimeras for PIN1 Enables Selective Degradation and Antitumor Activity In Vivo

Yuying Ma, Yang Teng, Jinjin Liu, Yuke Deng, Lingbo Xu, Ruichen Gao, Tingyu Peng, Wei Li, Yue Wei, Linfeng Li, and Zufeng Guo

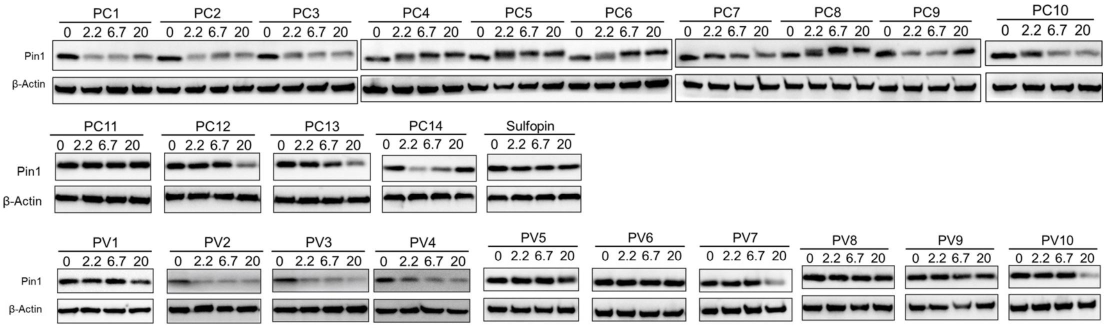

Figure S1. Preliminary degradation test of **PC** and **PV** compounds in PATU-8988T cells.

A

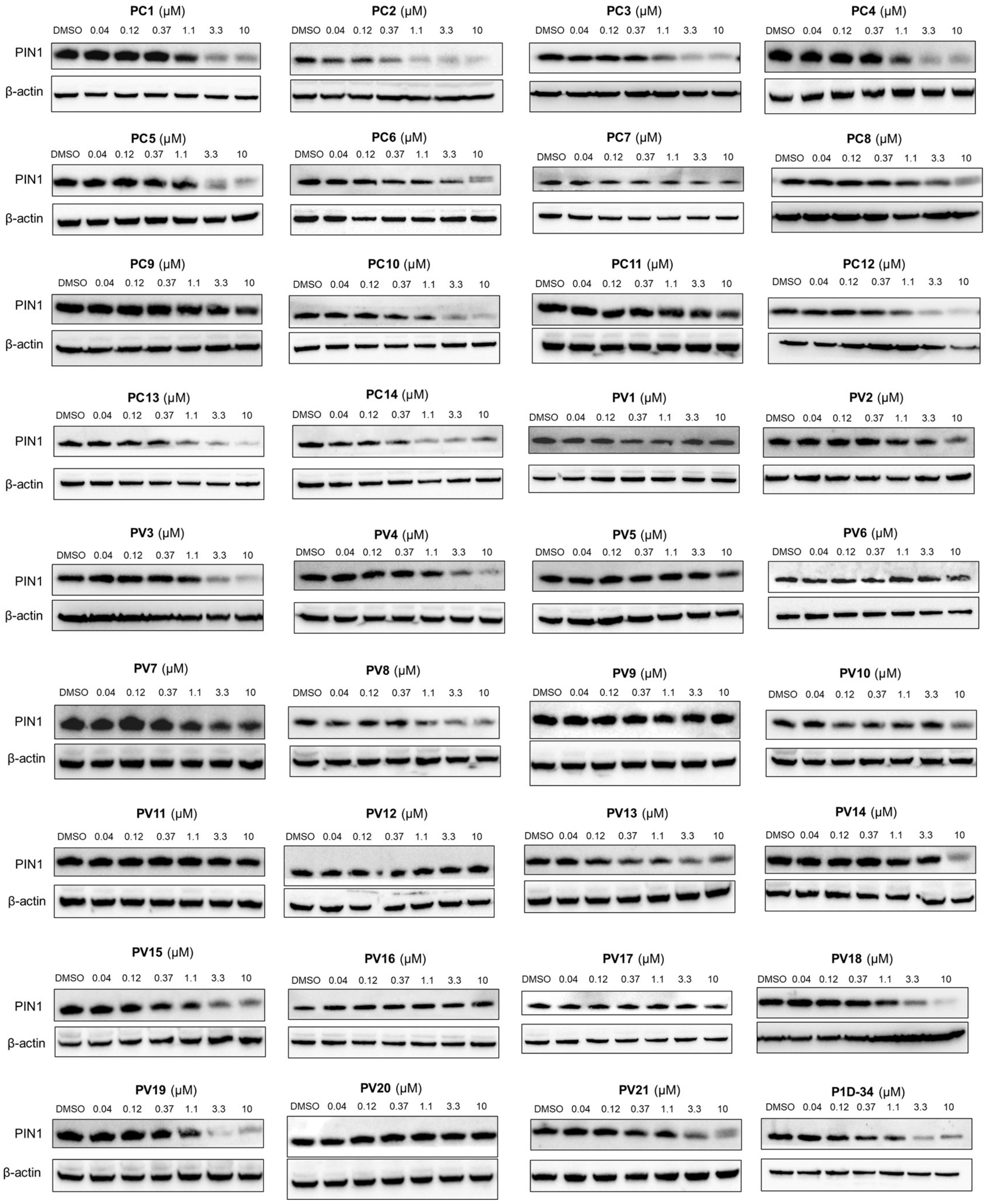
PC1 (µM)
PC2 (µM)
PC3 (µM)
PC4 (µM)
PC5 (µM)
PC6 (µM)
PC7 (µM)
PC8 (µM)
PC9 (µM)
PC10 (µM)
PC11 (µM)
PC12 (µM)
PC13 (µM)
PC14 (µM)
PV1 (µM)
PV2 (µM)
PV3 (µM)
PV4 (µM)
PV5 (µM)
PV6 (µM)
PV7 (µM)
PV8 (µM)
PV9 (µM)
PV10 (µM)
PV11 (µM)
PV12 (µM)
PV13 (µM)
PV14 (µM)
PV15 (µM)
PV16 (µM)
PV17 (µM)
PV18 (µM)
PV19 (µM)
PV20 (µM)
PV21 (µM)
P1D-34 (µM)
DMSO 0.04 0.12 0.37 1.1 3.3 10
PIN1
β-actin

B

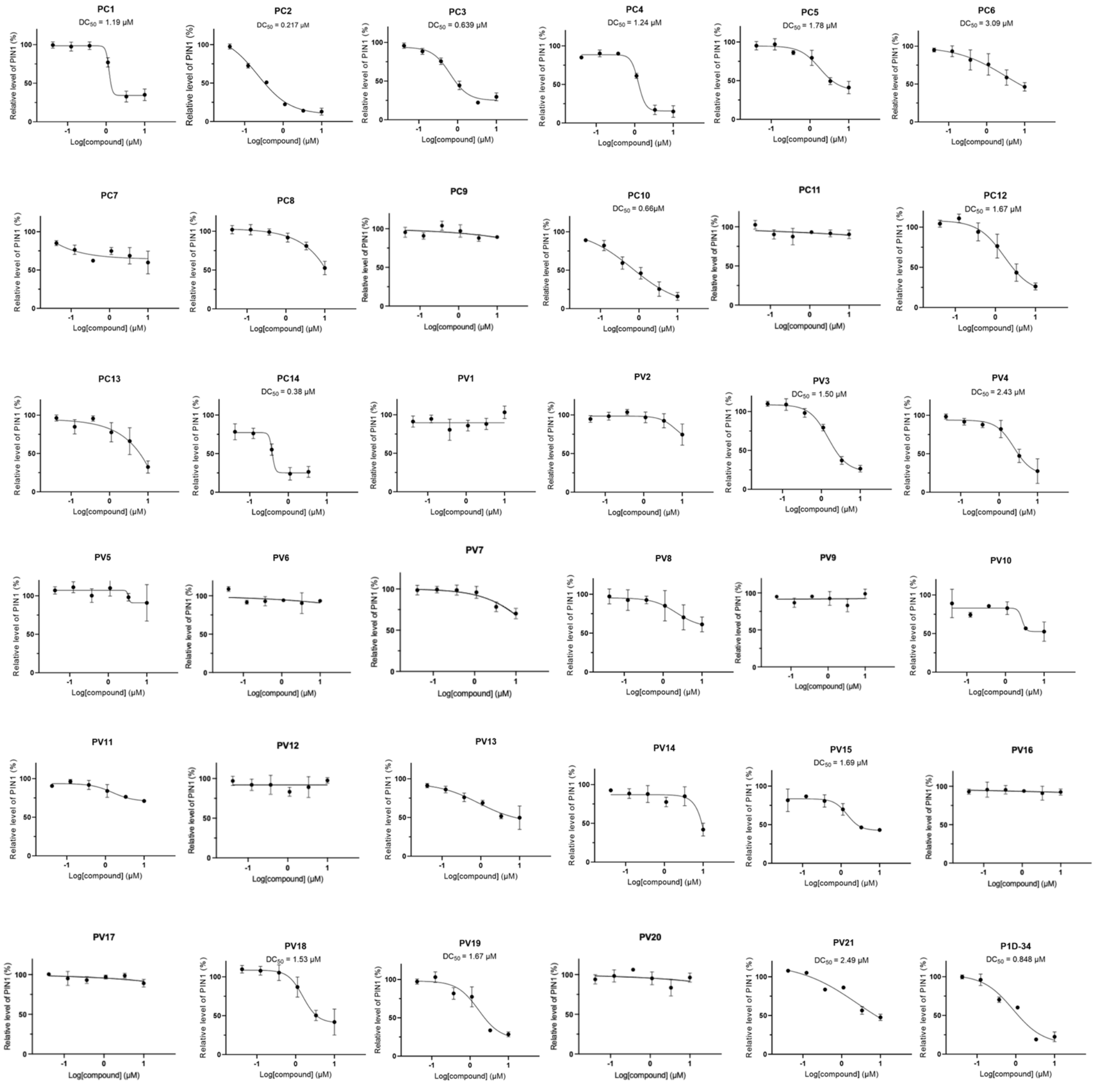

Figure S2. Concentration-dependent degradation test of **PC** and **PV** compounds in MCF-7 cells. (**A**) Representative immunoblots; (**B**) Concentration-response curves (n = 3).

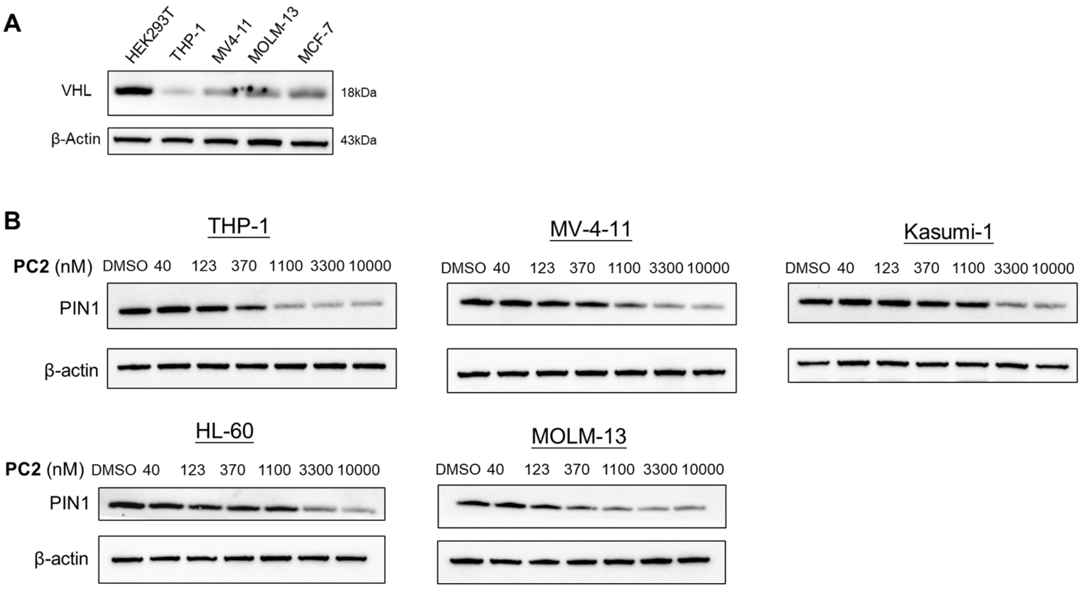

Figure S3. Immunoblots for (**A**) VHL protein levels in different cell lines, and (**B**) concentration-dependent experiments of **PC2** in a series of AML cell lines (n = 3).

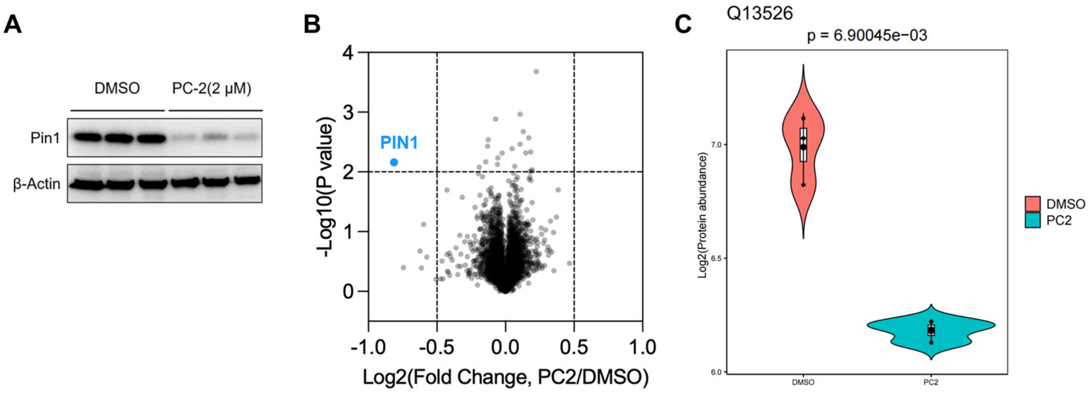

Figure S4. (**A**) Immunoblots of samples for proteomic study. (**B**) TMT-based proteomic profiling of PC2 (2μM) treatment in MCF-7 cells. (**C**) Violin plot for PIN1 (Uniprot ID: Q13526) in the TMT-based proteomic study.

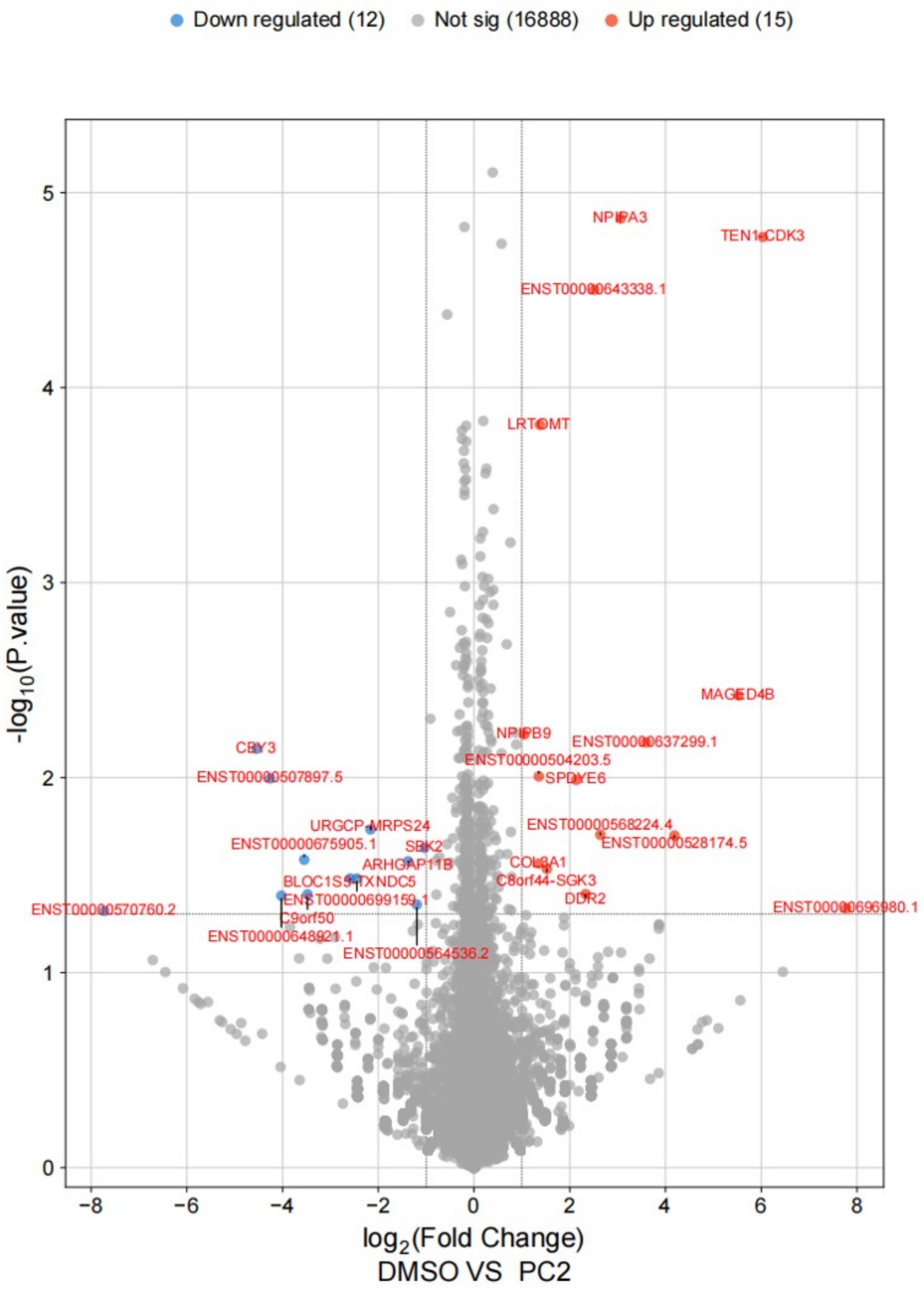

Figure S5. Volcano plot of RNA-seq data.

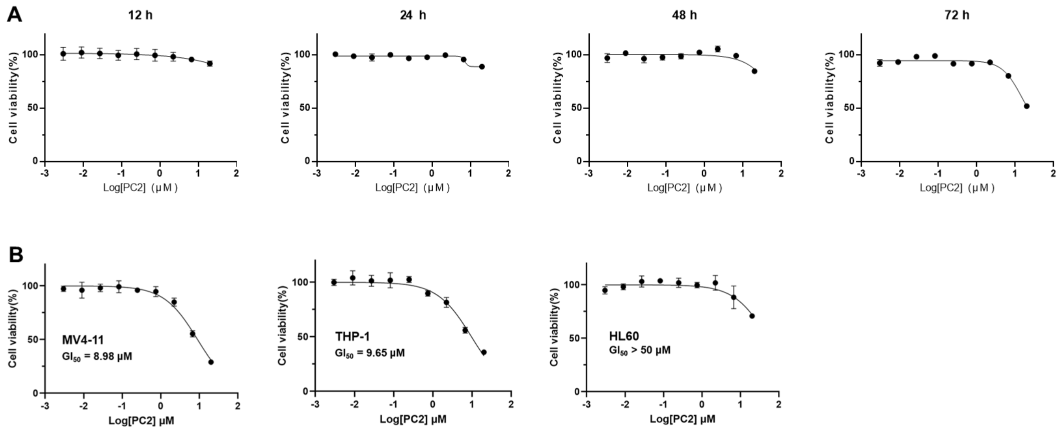

Figure S6. Antiproliferative effect of **PC2** (A) at different treatment durations in MCF-7 cells; (B) at the sixth day in different AML cell lines.

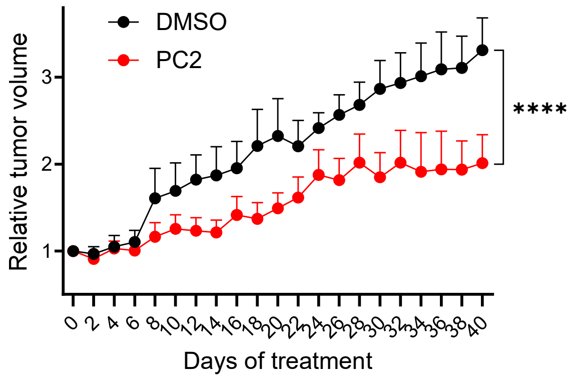

Figure S7. Relative tumor volume plot.

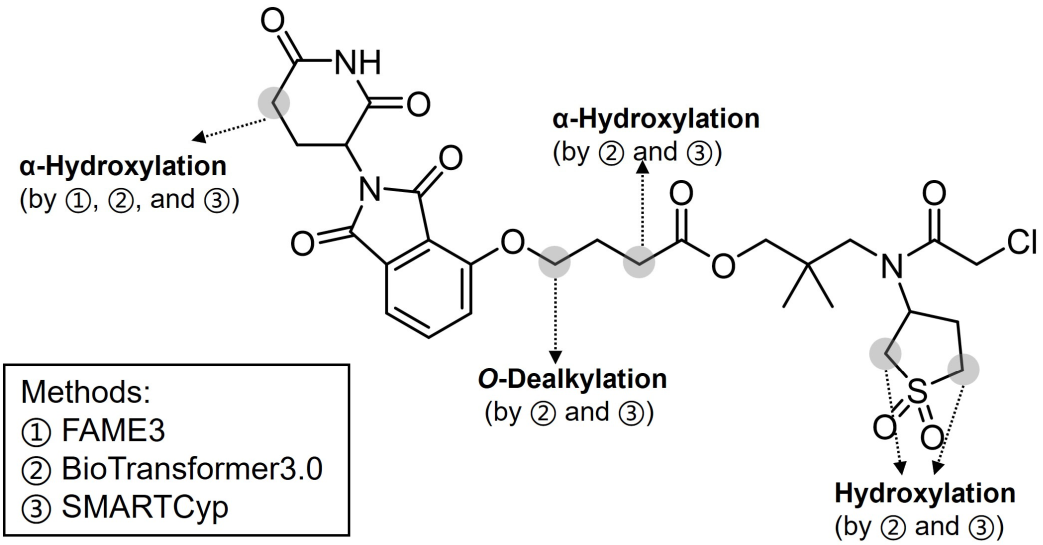

Figure S8. Predicted sites of metabolism and transformations of **PC2** by three computational tools.

Table S1. Permeability assay by PAMPA method.[a]

Compound	Test Conc. (μM)	$P_e \times 10^{-6}$ (cm/s)	-LOG P_e [c]	Recovery (%)
Methotrexate[b]	10	<0.0006	>9.23	<98.67
Testosterone	10	34.288	4.470	90.079
PC1	10	2.428	5.617	92.508
PC2	10	0.473	6.330	96.414
PC4[b]	10	<0.0009	>9.06	<101.92
PC5[b]	10	<0.0008	>9.12	<101.65
PV4	10	0.240	6.620	86.957
PV8[b]	10	<0.0005	>9.29	<95.16
PV12	10	0.101	6.997	93.858

[a] Each compound was evaluated in triplicate.

[b] 50 was used to calculate Pe if CA(t) was BLOD for data analysis.

[c] Passive permeability criterion: <5, high; 5~6, medium; >6, low.

Table S2. Interacting residues of PIN1 and CRBN in models of ternary complexes.

	CRBN	PIN1	Types of Interaction
**PC2**	E379	R69	Salt bridge
	E150; Y151; G152	R68; D153; Q131; K132	Salt bridge; HBond; VdW
	H355; Y357; F103	Q129; G128	HBond; VdW
**PC12**	Y151; G152; I153	R69; P70; S65; Q66	HBond; VdW

Table S3. Individual and mean plasma concentration-time data of **PC2** after and IP dose (ng/mL).

IP Time (h)	Plasma-01	Plasma-02	Plasma-03	Plasma-04	Plasma-05	Plasma-06	Mean IP		SD
0.25	99.74	80.83	249.59	53.06	62.95	126.22	112.065	±	72.298
0.5	153.72	386.12	669.96	127.34	223.84	250.40	301.897	±	201.859
1	75.18	35.43	65.75	14.77	17.72	134.11	57.160	±	45.043
2	26.81	14.20	5.01	8.12	BLQ	47.50	20.328	±	17.332
4	27.29	7.20	BLQ	3.85	BLQ	18.03	14.093	±	10.679
6	5.94	BLQ	BLQ	3.40	BLQ	5.33	4.890	±	1.326
8	4.89	BLQ	BLQ	BLQ	BLQ	8.25	6.570	±	2.376
24	BLQ	BLQ	BLQ	BLQ	BLQ	BLQ	ND	±	ND

ND = Not determined (Parameters not determined due to inadequately defined terminal elimination phase)

BLQ = Below the lower limit of quantitation (LLOQ).
