# Supplementary material for: Systematic Optimization of Proteolysis-Targeting Chimeras for PIN1 Enables Selective Degradation and Antitumor Activity In Vivo
Source: Pharmaceutics. 2026 Feb 26;18(3):288. doi: 10.3390/pharmaceutics18030288 (PMC13029591; doi:10.3390/pharmaceutics18030288)

pharmaceutics

MDPI

Supplementary Materials: Systematic Optimization of Proteolysis-Targeting Chimeras for PIN1 Enables Selective Degradation and Antitumor Activity In Vivo

Yuying Ma, Yang Teng, Jinjin Liu, Yuke Deng, Lingbo Xu, Ruichen Gao, Tingyu Peng, Wei Li, Yue Wei, Linfeng Li, and Zufeng Guo

^{1}H and ^{13}C NMR spectra

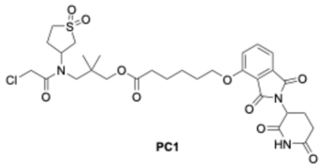

PC1

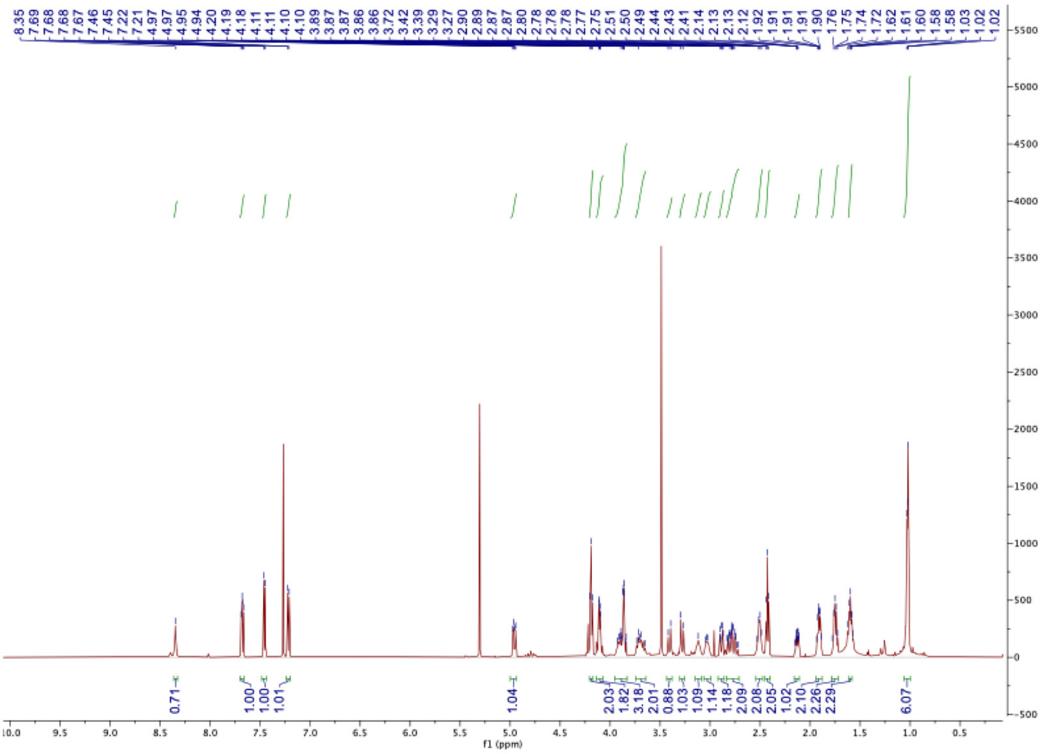

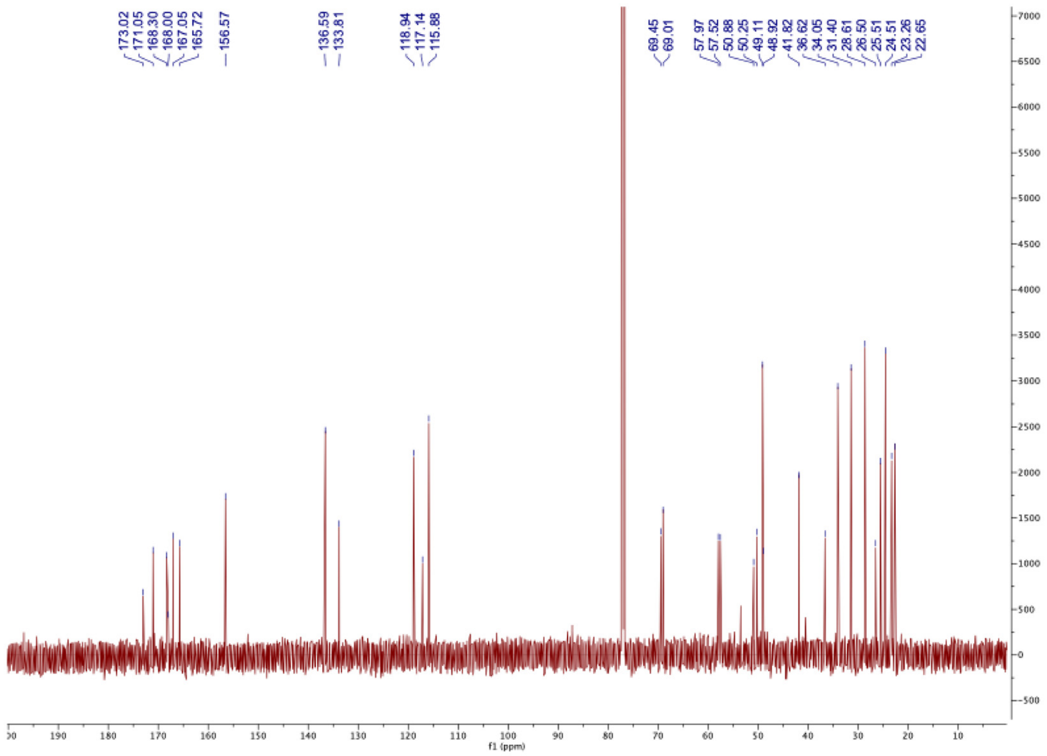
173.02
171.05
168.30
168.00
167.05
165.72
156.57
136.59
133.81
118.94
117.14
115.88
69.45
69.01
57.97
57.52
50.88
50.25
49.11
48.92
41.82
36.62
34.05
31.40
28.61
26.50
25.51
24.51
23.26
22.65
f1 (ppm)

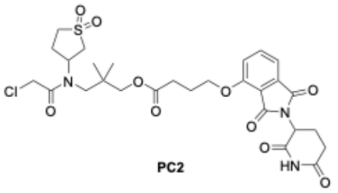
Cl
N
O
S
HN
O

PC2

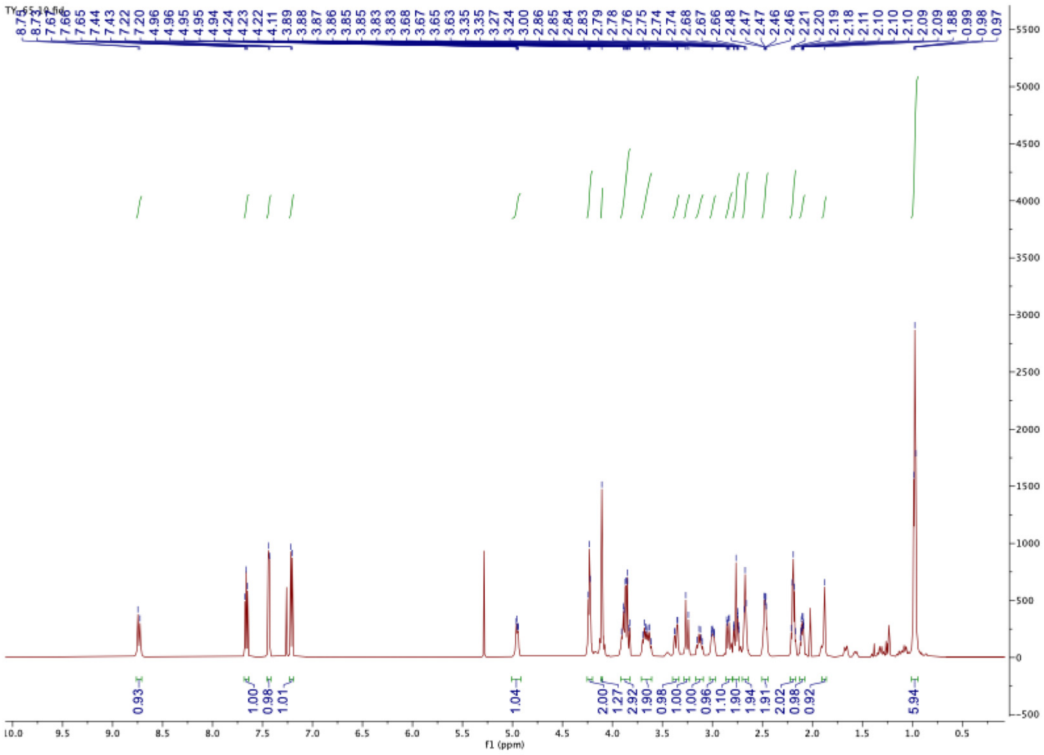
TY-65.10.fid
8.74
7.67
7.66
7.65
7.44
7.43
7.22
7.20
4.96
4.96
4.95
4.95
4.94
4.24
4.23
4.22
4.11
3.89
3.88
3.87
3.86
3.85
3.85
3.83
3.83
3.68
3.67
3.65
3.63
3.35
3.35
3.27
3.24
3.00
2.86
2.85
2.84
2.83
2.79
2.78
2.76
2.75
2.74
2.74
2.68
2.67
2.66
2.48
2.47
2.47
2.46
2.21
2.20
2.19
2.18
2.11
2.10
2.10
2.10
2.09
2.09
1.88
0.99
0.98
0.97
0.93
1.00
0.98
1.01
1.04
2.00
1.27
2.92
1.90
0.98
1.00
1.00
0.96
1.10
1.90
1.94
1.91
2.02
0.98
0.92
5.94
f1 (ppm)

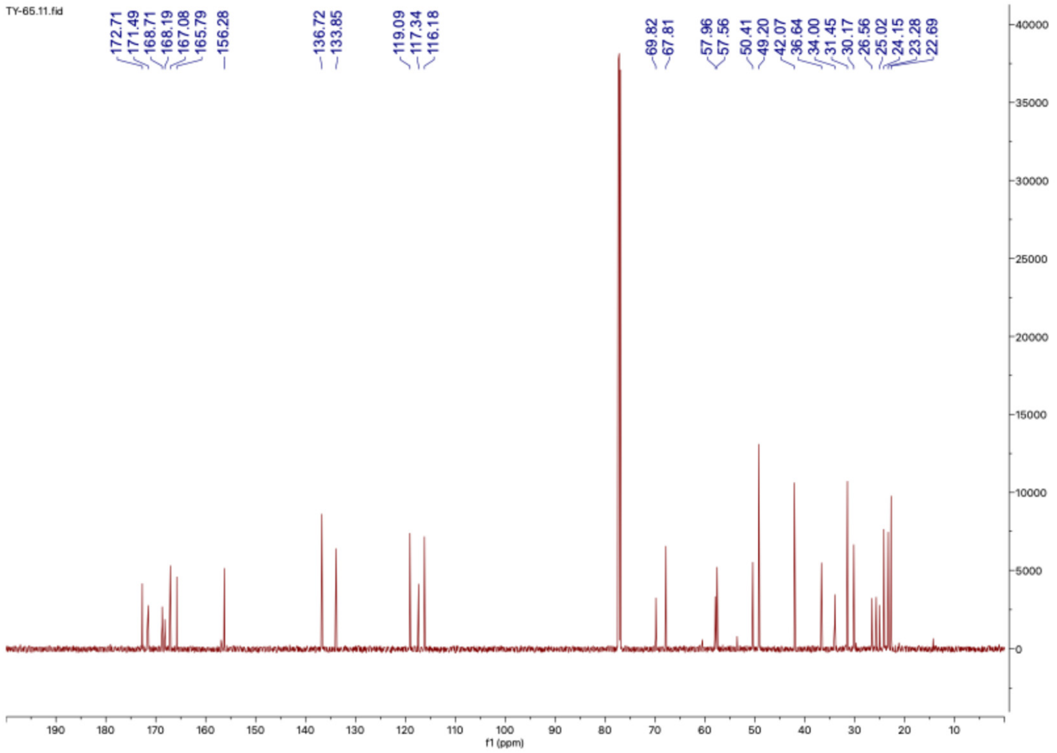
TY-65.11.fid
172.71
171.49
168.71
168.19
167.08
165.79
156.28
136.72
133.85
119.09
117.34
116.18
69.82
67.81
57.96
57.56
50.41
49.20
42.07
36.64
34.00
31.45
30.17
26.56
25.02
24.15
23.28
22.69
f1 (ppm)

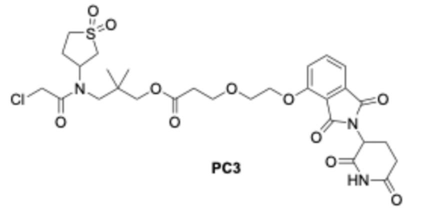

PC3

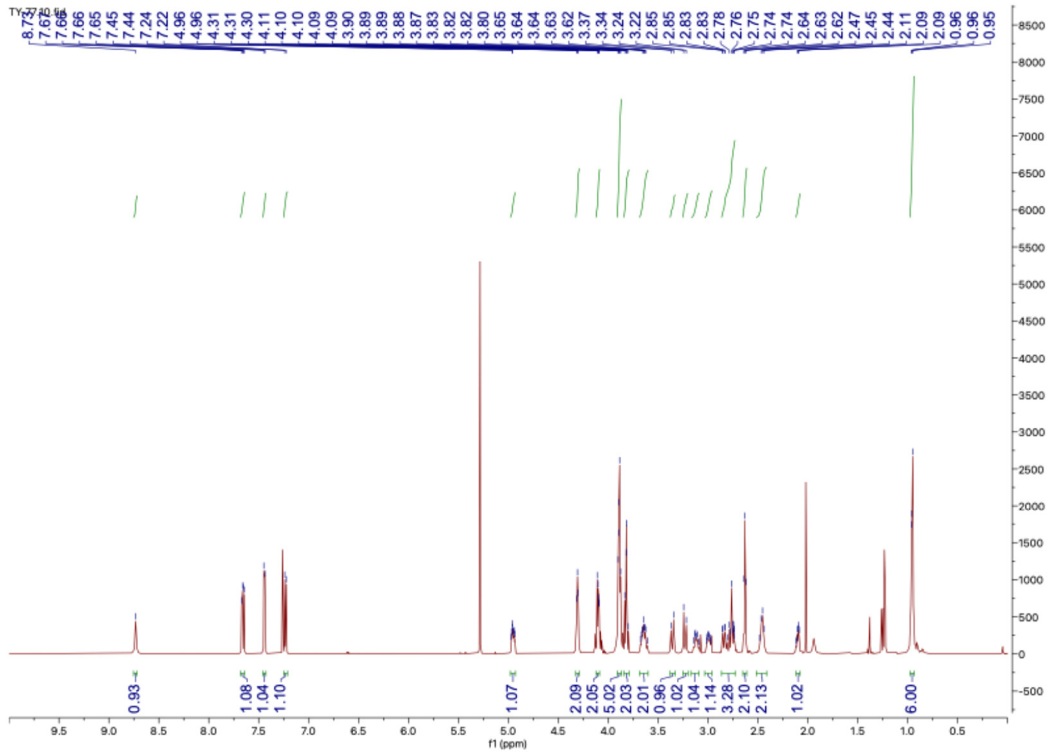
8.73
7.67
7.66
7.66
7.65
7.45
7.44
7.24
7.22
4.96
4.96
4.31
4.30
4.11
4.10
4.09
4.09
3.90
3.89
3.89
3.88
3.87
3.83
3.82
3.82
3.80
3.65
3.64
3.64
3.63
3.62
3.37
3.34
3.24
3.22
2.85
2.85
2.83
2.83
2.78
2.76
2.75
2.74
2.74
2.64
2.63
2.62
2.47
2.45
2.44
2.11
2.09
0.96
0.95
0.93
1.08
1.04
1.10
1.07
2.09
2.05
5.02
2.03
2.01
0.96
1.02
1.04
1.14
3.28
2.10
2.13
1.02
6.00
f1 (ppm)

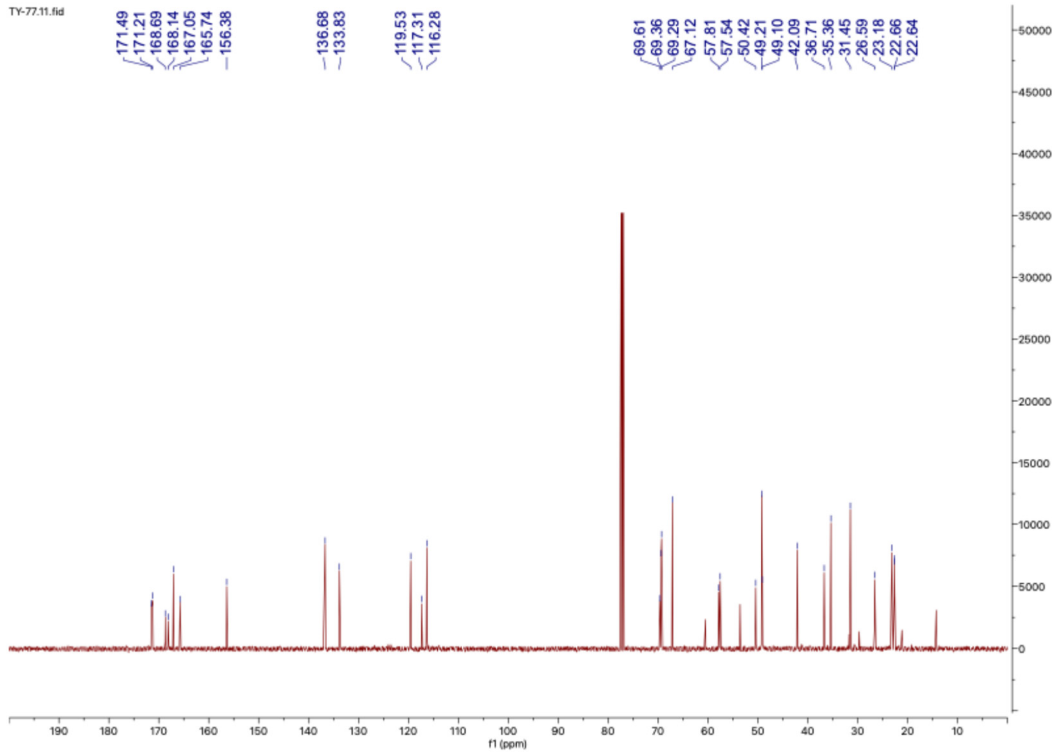
TY-77.11.fid
171.49
171.21
168.69
168.14
167.05
165.74
156.38
136.68
133.83
119.53
117.31
116.28
69.61
69.36
69.29
67.12
57.81
57.54
50.42
49.21
49.10
42.09
36.71
35.36
31.45
26.59
23.18
22.66
22.64
f1 (ppm)

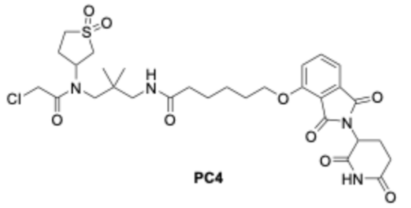

Cl
O
N
S
O
O
H
N
O
O
O
N
O
O
O
HN
O

PC4

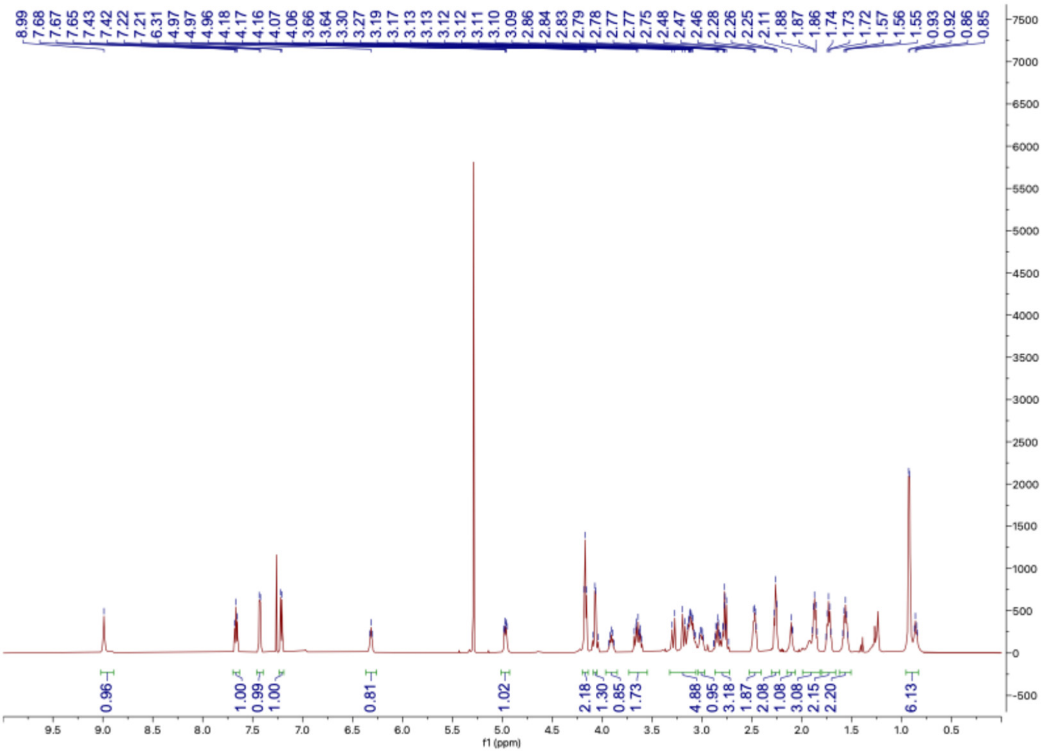

8.99 7.68 7.67 7.65 7.43 7.42 7.22 7.21 6.31 4.97 4.97 4.96 4.18 4.17 4.16 4.07 4.06 3.66 3.64 3.30 3.27 3.19 3.17 3.13 3.13 3.12 3.12 3.11 3.10 3.09 2.86 2.84 2.83 2.79 2.78 2.77 2.77 2.75 2.48 2.47 2.46 2.28 2.26 2.25 2.11 1.88 1.87 1.86 1.74 1.73 1.72 1.57 1.56 1.55 0.93 0.92 0.86 0.85
0.96 1.00 0.99 1.00 0.81 1.02 2.18 1.30 0.85 1.73 4.88 0.95 3.18 1.87 2.08 1.08 3.08 2.15 2.20 6.13
f1 (ppm)

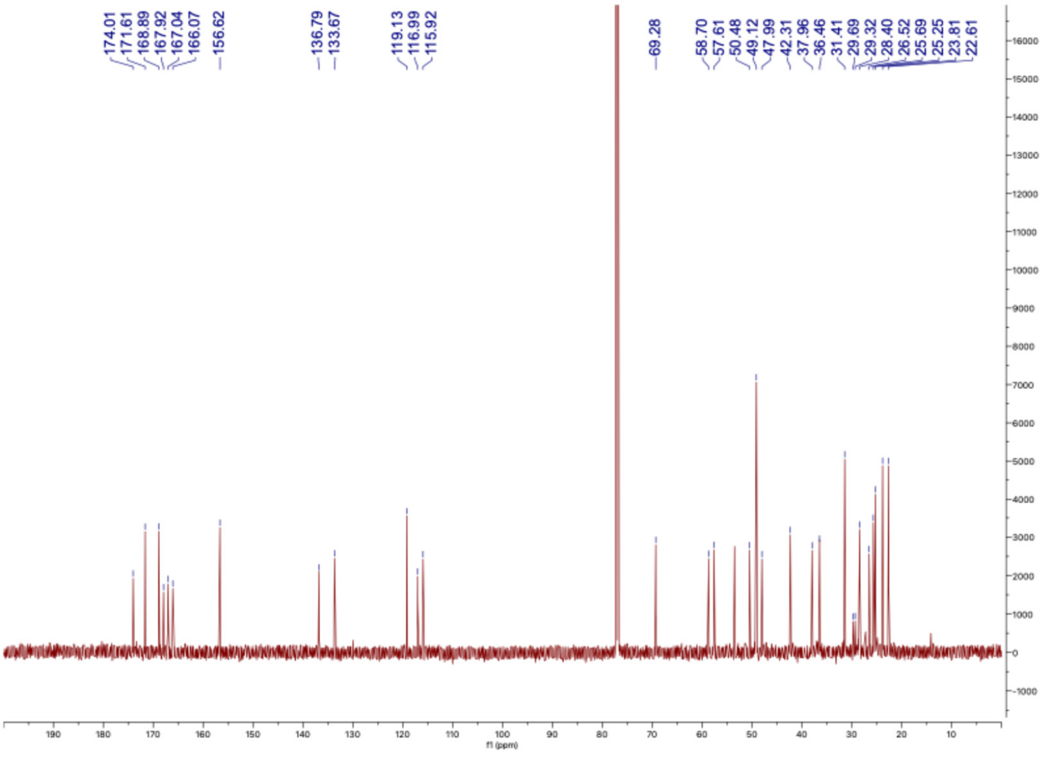

174.01 171.61 168.89 167.92 167.04 166.07 156.62 136.79 133.67 119.13 116.99 115.92 69.28 58.70 57.61 50.48 49.12 47.99 42.31 37.96 36.46 31.41 29.69 29.32 28.40 26.52 25.69 25.25 23.81 22.61
f1 (ppm)

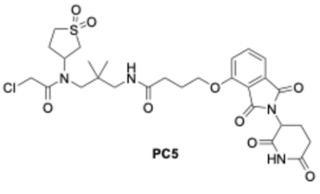
Cl
N
O
S
O
O
H
N
O
O
O
N
O
O
HN
O

PC5

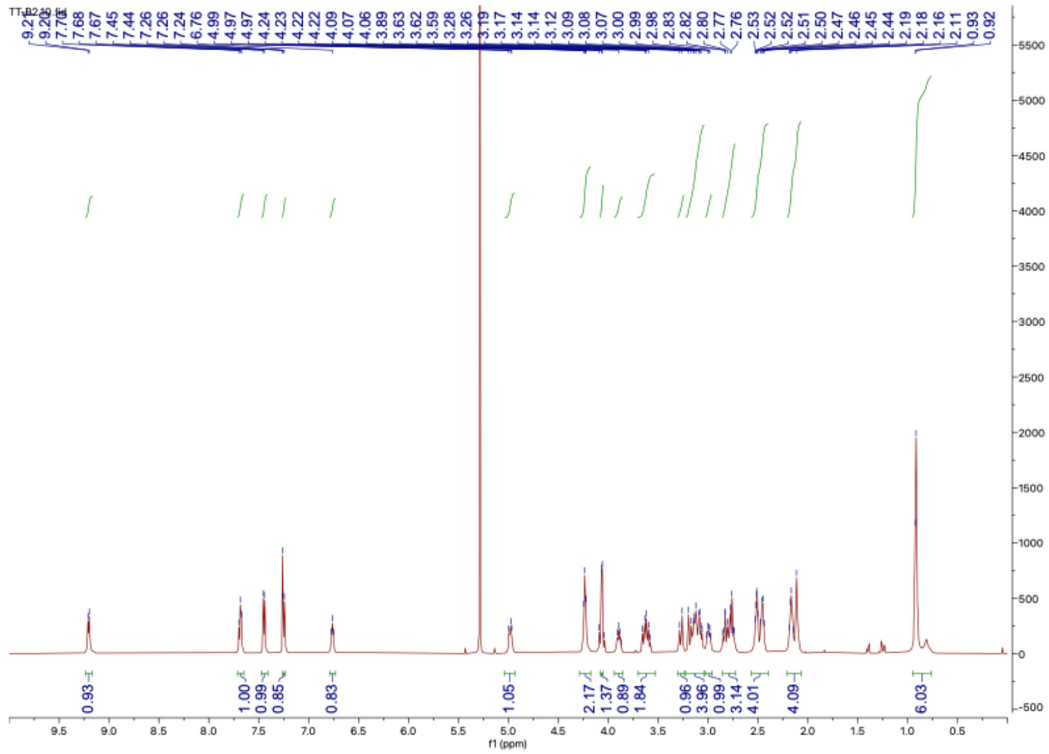
TT-B2.10.fid
9.21
9.20
7.70
7.68
7.67
7.45
7.44
7.26
7.26
7.24
6.76
4.99
4.97
4.97
4.24
4.23
4.22
4.22
4.09
4.07
4.06
3.89
3.63
3.62
3.59
3.28
3.26
3.19
3.17
3.14
3.14
3.12
3.09
3.08
3.07
3.00
2.99
2.98
2.83
2.82
2.80
2.77
2.76
2.53
2.52
2.52
2.51
2.50
2.47
2.46
2.45
2.44
2.19
2.18
2.16
2.11
0.93
0.92
0.93
1.00
0.99
0.85
0.83
1.05
2.17
1.37
0.89
1.84
0.96
3.96
0.99
3.14
4.01
4.09
6.03
f1 (ppm)

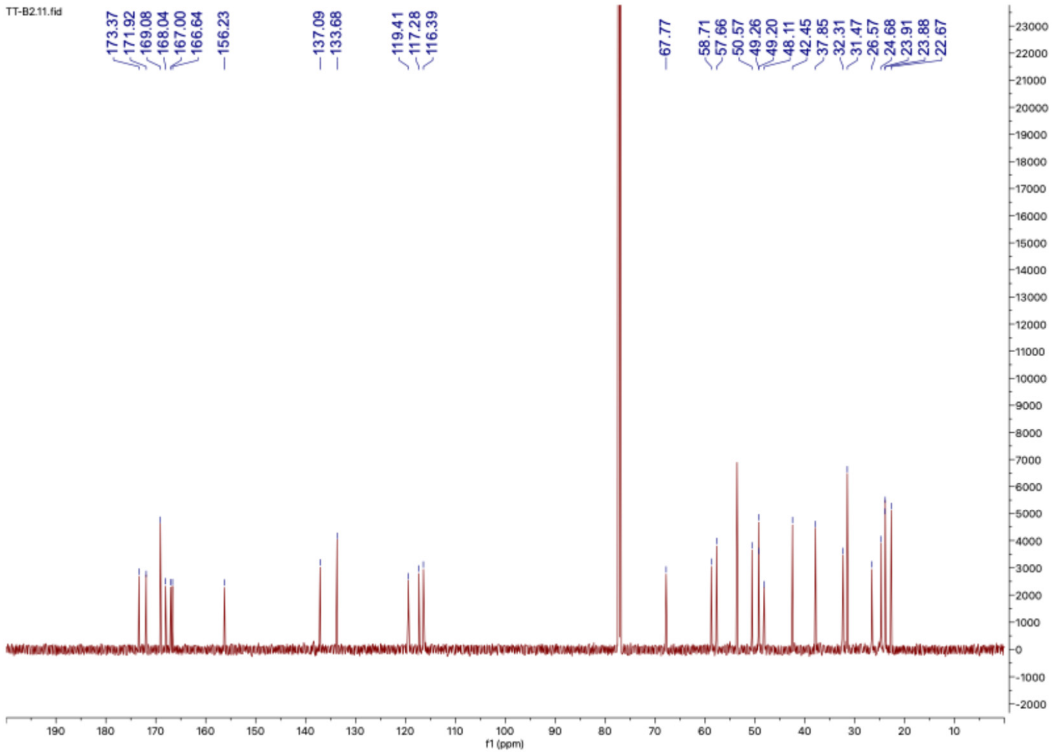
TT-B2.11.fid
173.37
171.92
169.08
168.04
167.00
166.64
156.23
137.09
133.68
119.41
117.28
116.39
67.77
58.71
57.66
50.57
49.26
49.20
48.11
42.45
37.85
32.31
31.47
26.57
24.68
23.91
23.88
22.67
f1 (ppm)

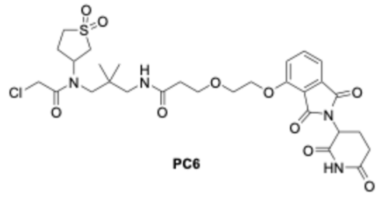
Cl
N
H
N
O
O
O
O
S
O
O
N
O
O
HN
O
O

PC6

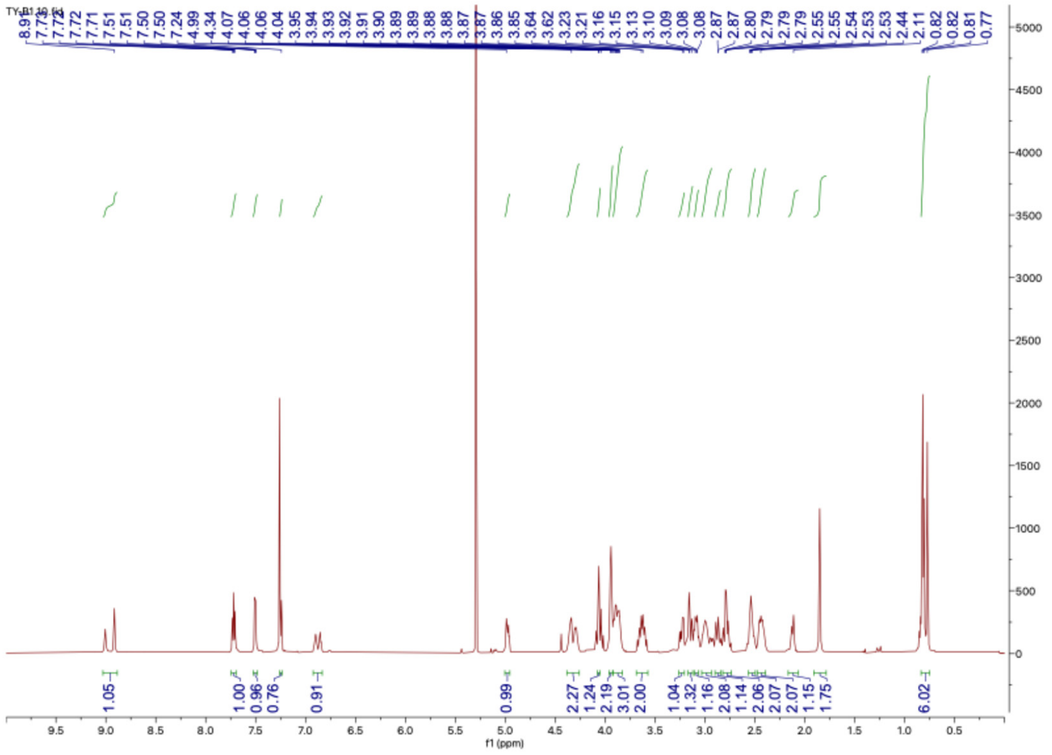
TY-B1.10.fid
7.72
7.71
7.51
7.51
7.50
7.50
7.24
4.99
4.34
4.07
4.06
4.06
4.04
3.95
3.94
3.93
3.92
3.91
3.90
3.89
3.89
3.88
3.88
3.87
3.87
3.86
3.85
3.64
3.62
3.23
3.21
3.16
3.15
3.13
3.10
3.09
3.08
3.08
2.87
2.87
2.80
2.79
2.79
2.79
2.55
2.55
2.54
2.53
2.53
2.44
2.11
0.82
0.82
0.81
0.77
1.05
1.00
0.96
0.76
0.91
0.99
2.27
1.24
2.19
3.01
2.00
1.04
1.32
1.16
2.08
1.14
2.06
2.07
2.07
1.15
1.75
6.02
f1 (ppm)

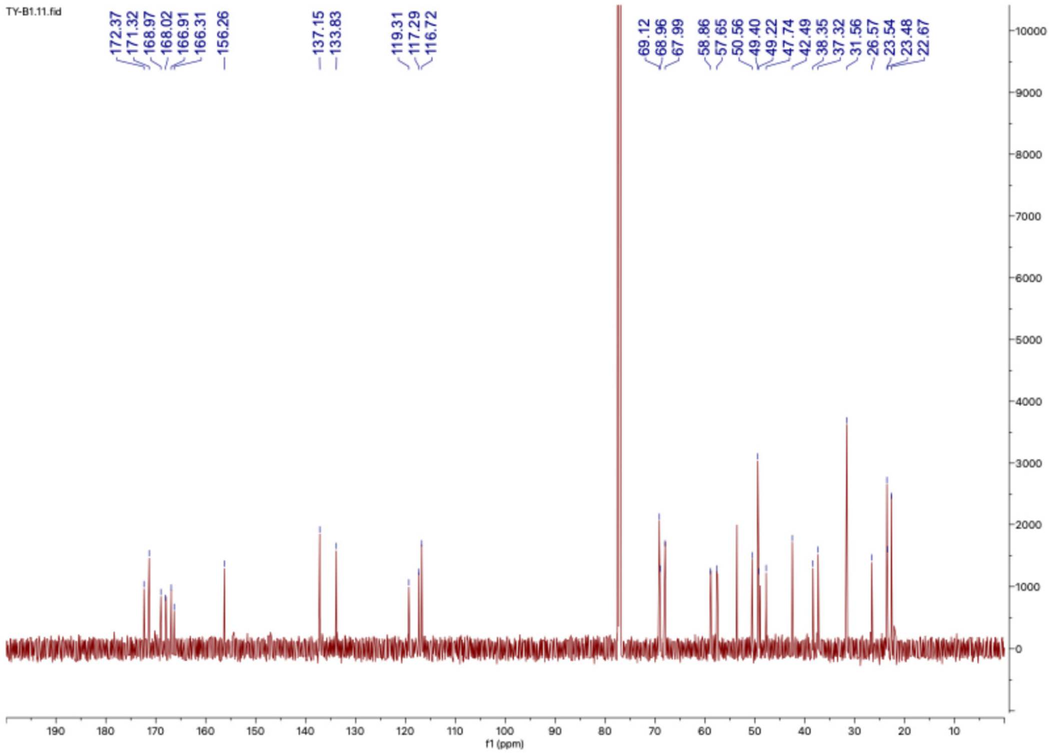
TY-B1.11.fid
172.37
171.32
168.97
168.02
166.91
166.31
156.26
137.15
133.83
119.31
117.29
116.72
69.12
68.96
67.99
58.86
57.65
50.56
49.40
49.22
47.74
42.49
38.35
37.32
31.56
26.57
23.54
23.48
22.67
f1 (ppm)

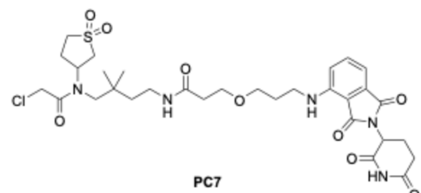
Cl
N
N
H
O
N
H
HN

PC7

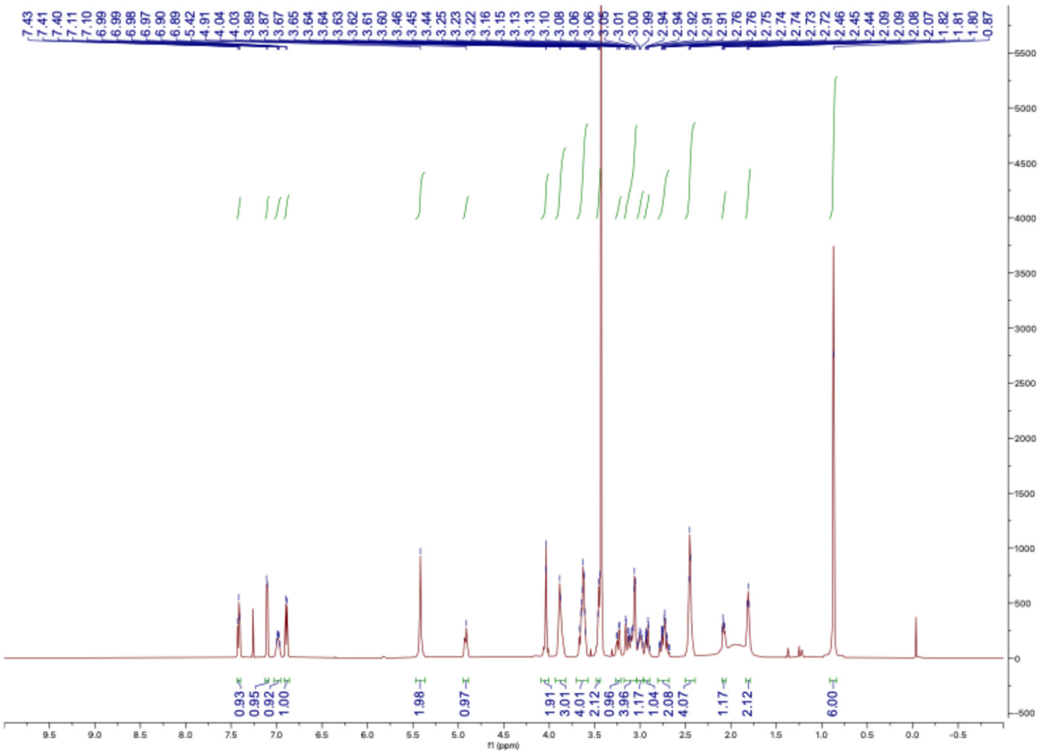
7.43
7.41
7.40
7.11
7.10
6.99
6.99
6.98
6.97
6.90
6.89
5.42
4.91
4.04
4.03
3.89
3.87
3.67
3.65
3.64
3.64
3.63
3.62
3.61
3.60
3.46
3.45
3.44
3.25
3.23
3.22
3.16
3.15
3.13
3.13
3.10
3.08
3.06
3.06
3.06
3.05
3.01
3.00
2.99
2.94
2.94
2.92
2.91
2.91
2.76
2.76
2.75
2.74
2.74
2.73
2.72
2.46
2.45
2.44
2.09
2.09
2.08
2.07
1.82
1.81
1.80
0.87
0.93
0.95
0.92
1.00
1.98
0.97
1.91
3.01
4.01
2.12
0.96
3.96
1.17
1.04
2.08
4.07
1.17
2.12
6.00
f1 (ppm)

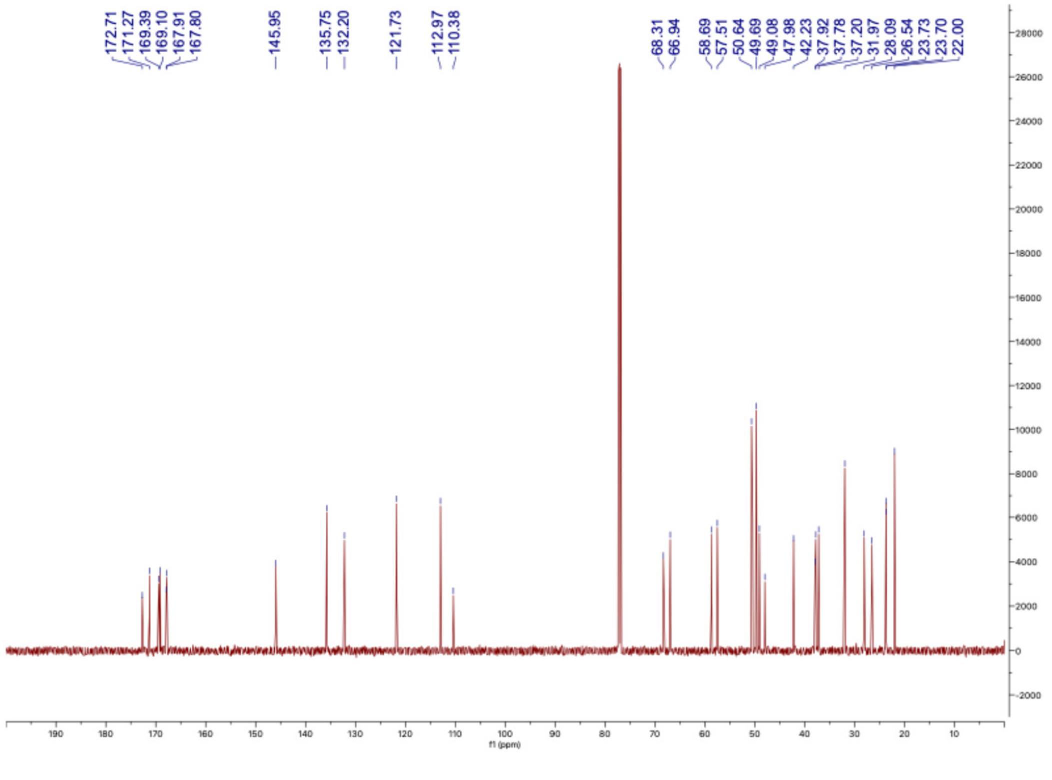
172.71
171.27
169.39
169.10
167.91
167.80
145.95
135.75
132.20
121.73
112.97
110.38
68.31
66.94
58.69
57.51
50.64
49.69
49.08
47.98
42.23
37.92
37.78
37.20
31.97
28.09
26.54
23.73
23.70
22.00
f1 (ppm)

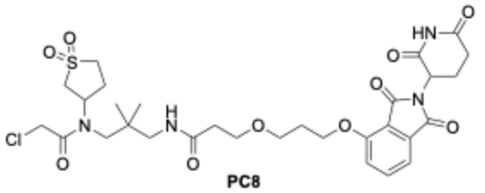

O
O
S
Cl
N
O
H
N
O
O
O
HN
O
O
O
N
O

PC8

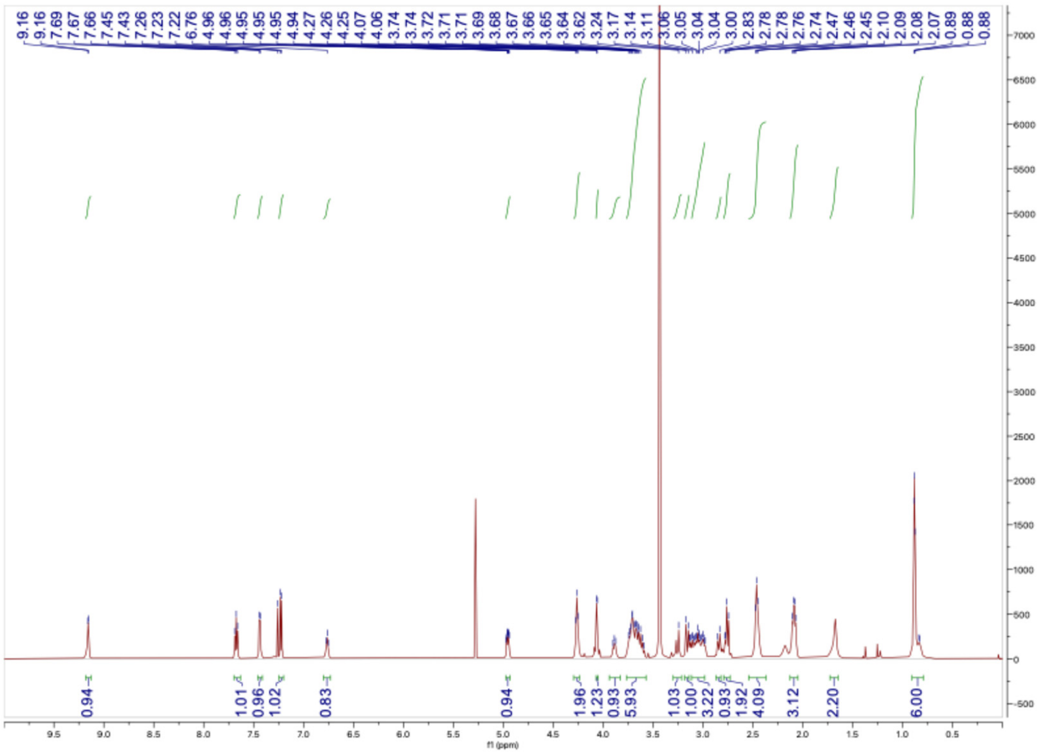

9.16 9.16 7.69 7.67 7.66 7.45 7.43 7.26 7.23 7.22 6.76 4.96 4.95 4.95 4.95 4.94 4.27 4.26 4.25 4.07 4.06 3.74 3.74 3.72 3.71 3.69 3.68 3.67 3.66 3.65 3.64 3.62 3.24 3.17 3.14 3.11 3.06 3.05 3.04 3.04 3.00 2.83 2.78 2.76 2.74 2.47 2.46 2.45 2.10 2.09 2.08 2.07 0.89 0.88 0.88
0.94 1.01 0.96 1.02 0.83 0.94 1.96 1.23 0.93 5.93 1.03 1.00 3.22 0.93 1.92 4.09 3.12 2.20 6.00
f1 (ppm)

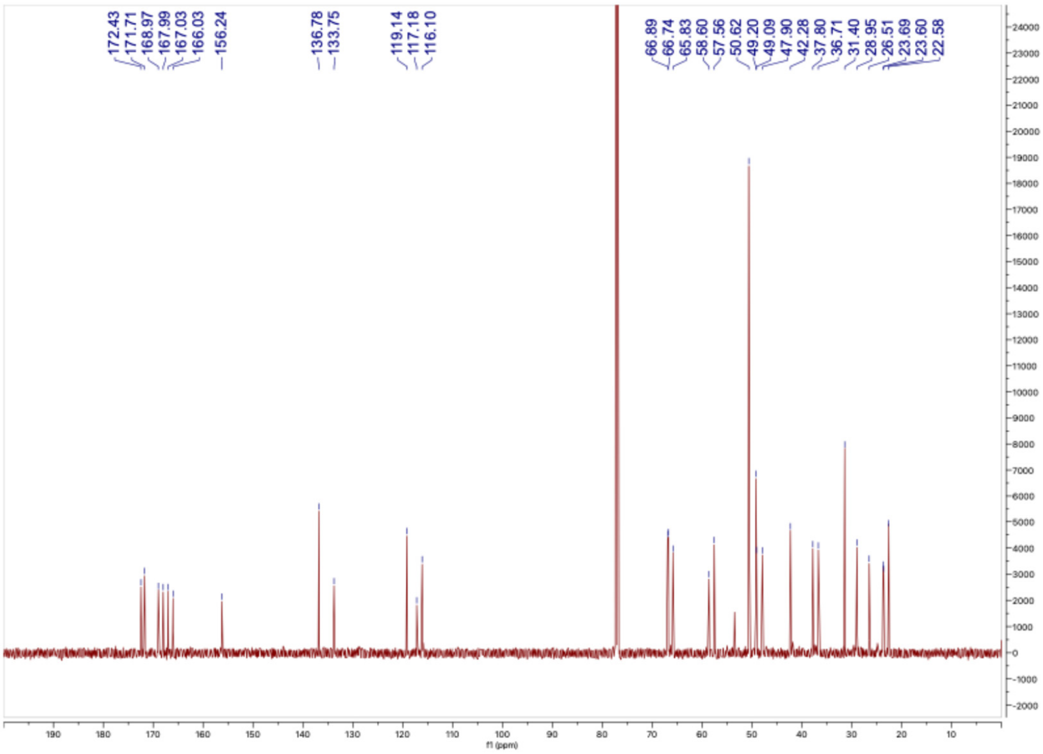

172.43 171.71 168.97 167.99 167.03 166.03 156.24 136.78 133.75 119.14 117.18 116.10 66.89 66.74 65.83 58.60 57.56 50.62 49.20 49.09 47.90 42.28 37.80 36.71 31.40 28.95 26.51 23.69 23.60 22.58
f1 (ppm)

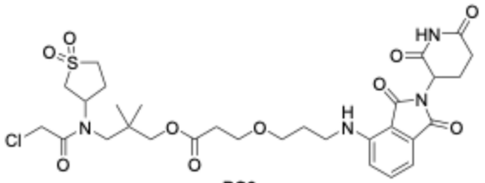
Cl
N
O
S
H
HN

PC9

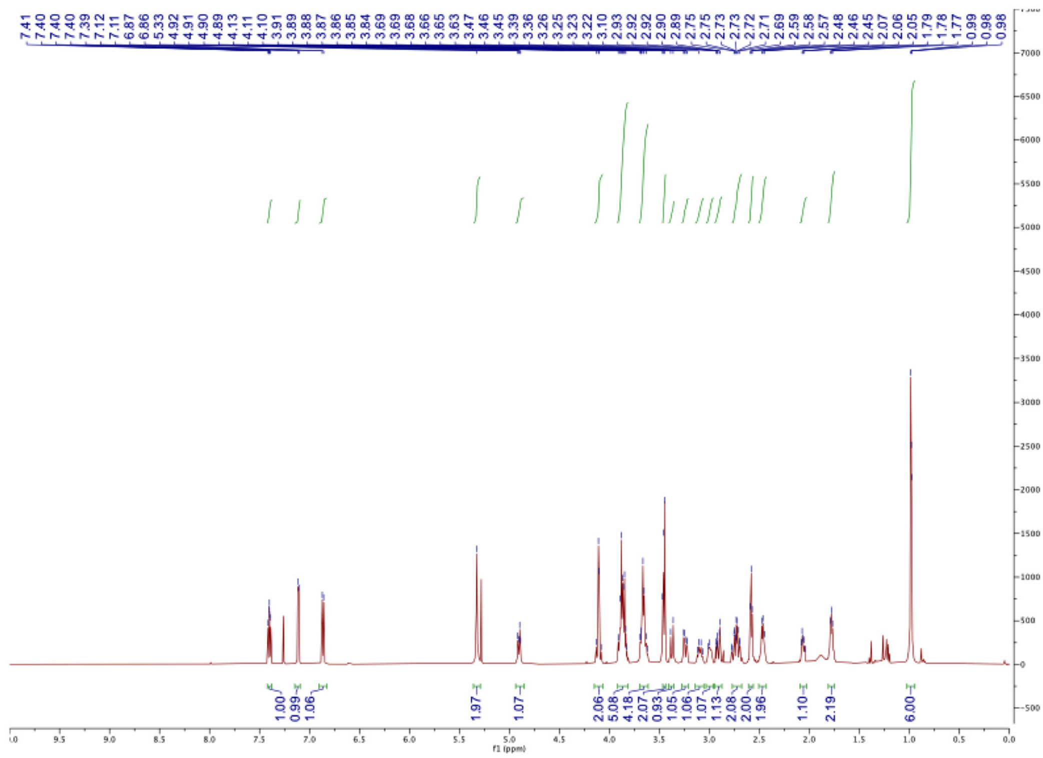

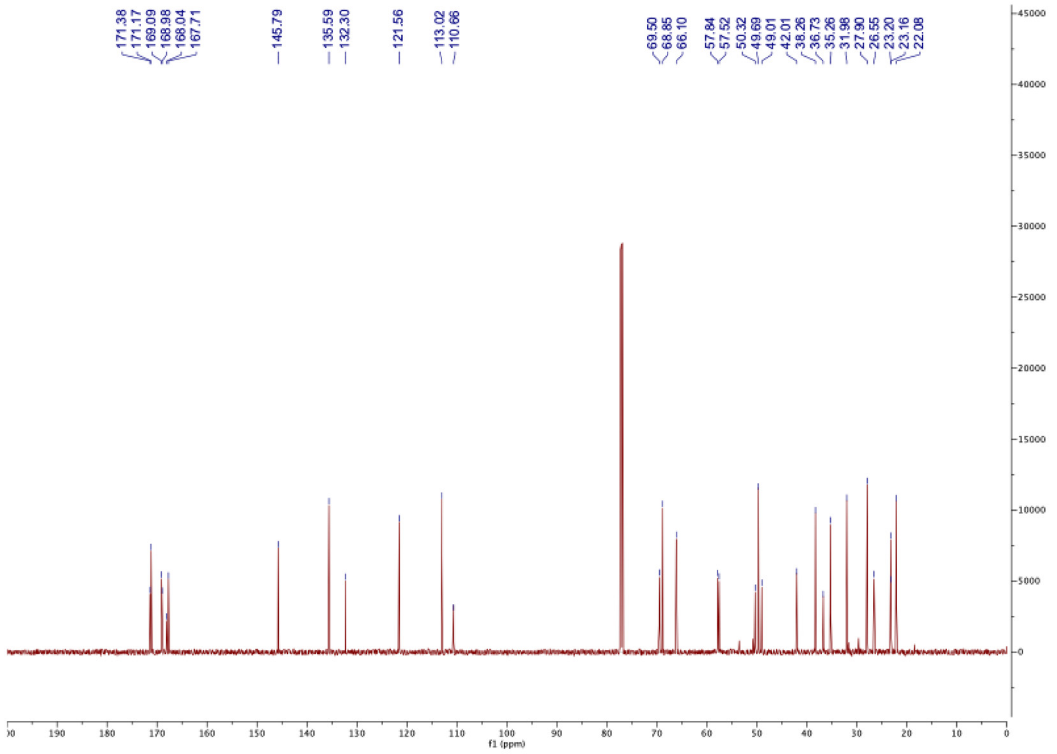
171.38
171.17
169.09
168.98
168.04
167.71
145.79
135.59
132.30
121.56
113.02
110.66
69.50
68.85
66.10
57.84
57.52
50.32
49.69
49.01
42.01
38.26
36.73
35.26
31.98
27.90
26.55
23.20
23.16
22.08
f1 (ppm)

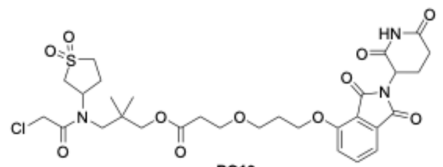
O
O
S
Cl
N
O
O
O
O
O
HN
O
O
N
O
O

PC10

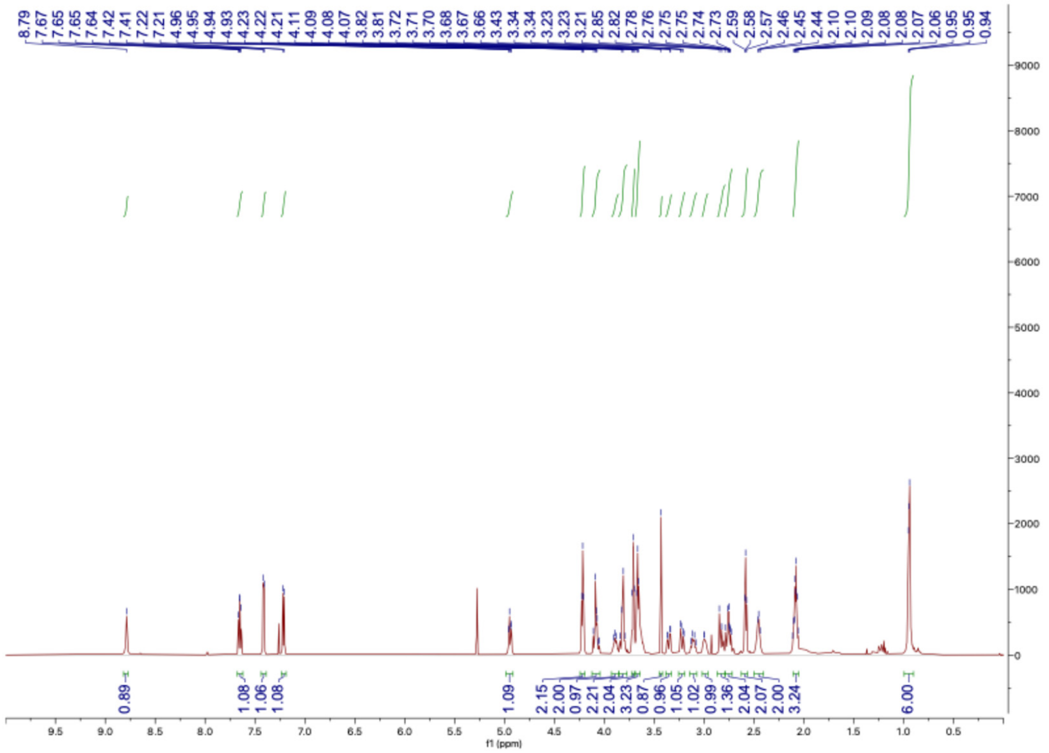
8.79 7.67 7.65 7.64 7.42 7.41 7.22 7.21 4.96 4.95 4.94 4.93 4.23 4.22 4.21 4.11 4.09 4.08 4.07 3.82 3.81 3.72 3.71 3.70 3.68 3.67 3.66 3.43 3.34 3.34 3.23 3.23 3.21 2.85 2.82 2.78 2.76 2.75 2.75 2.74 2.73 2.59 2.58 2.57 2.46 2.45 2.44 2.10 2.09 2.08 2.08 2.07 2.06 0.95 0.94
0.89 1.08 1.06 1.08 1.09 2.15 2.00 0.97 2.21 2.04 3.23 0.87 0.96 1.05 1.02 0.99 1.36 2.04 2.07 2.00 3.24 6.00
f1 (ppm)

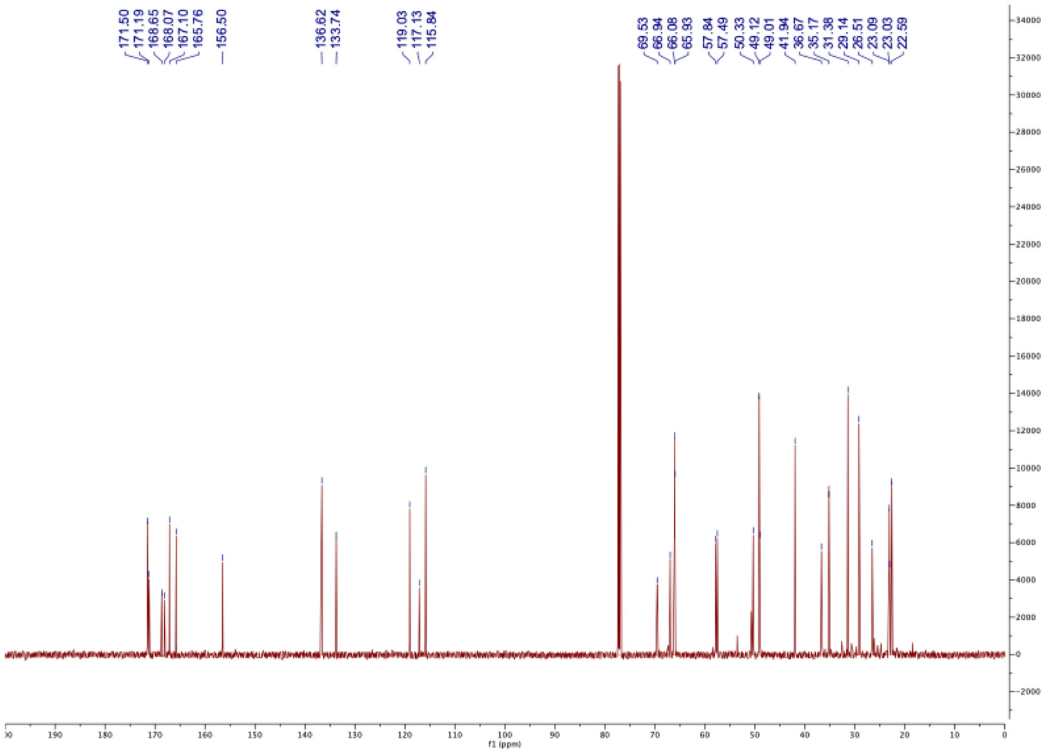
171.50 171.19 168.65 168.07 167.10 165.76 156.50 136.62 133.74 119.03 117.13 115.84 69.53 66.94 66.08 65.93 57.84 57.49 50.33 49.12 49.01 41.94 36.67 35.17 31.38 29.14 26.51 23.09 23.03 22.59
f1 (ppm)

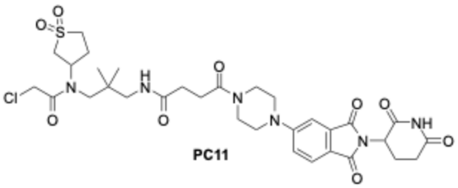

PC11

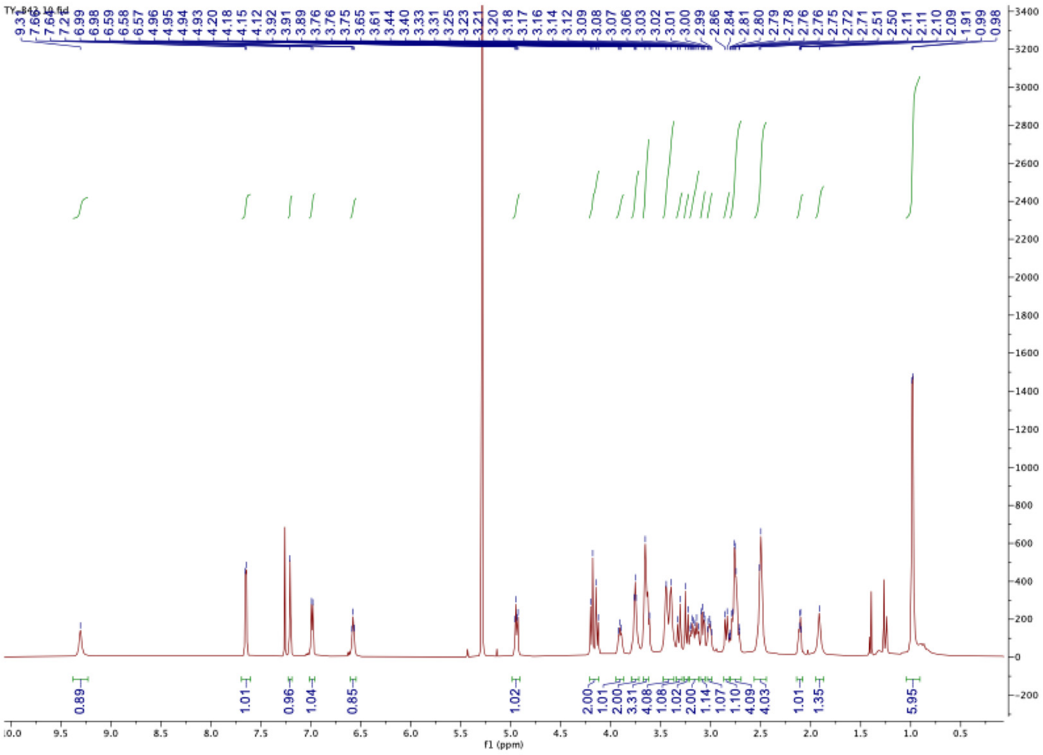
6.99
6.98
6.59
6.58
6.57
4.96
4.95
4.94
4.93
4.20
4.18
4.15
4.12
3.92
3.91
3.89
3.76
3.76
3.75
3.65
3.61
3.44
3.40
3.33
3.31
3.25
3.23
3.21
3.20
3.18
3.17
3.16
3.14
3.12
3.09
3.08
3.07
3.06
3.03
3.02
3.01
3.00
2.99
2.86
2.84
2.81
2.80
2.79
2.78
2.76
2.76
2.75
2.72
2.71
2.51
2.50
2.11
2.11
2.10
2.09
1.91
0.99
0.98
0.89
1.01
0.96
1.04
0.85
1.02
2.00
1.01
2.00
3.31
4.08
1.08
1.02
2.00
1.14
1.07
1.10
4.09
4.03
1.01
1.35
5.95
f1 (ppm)

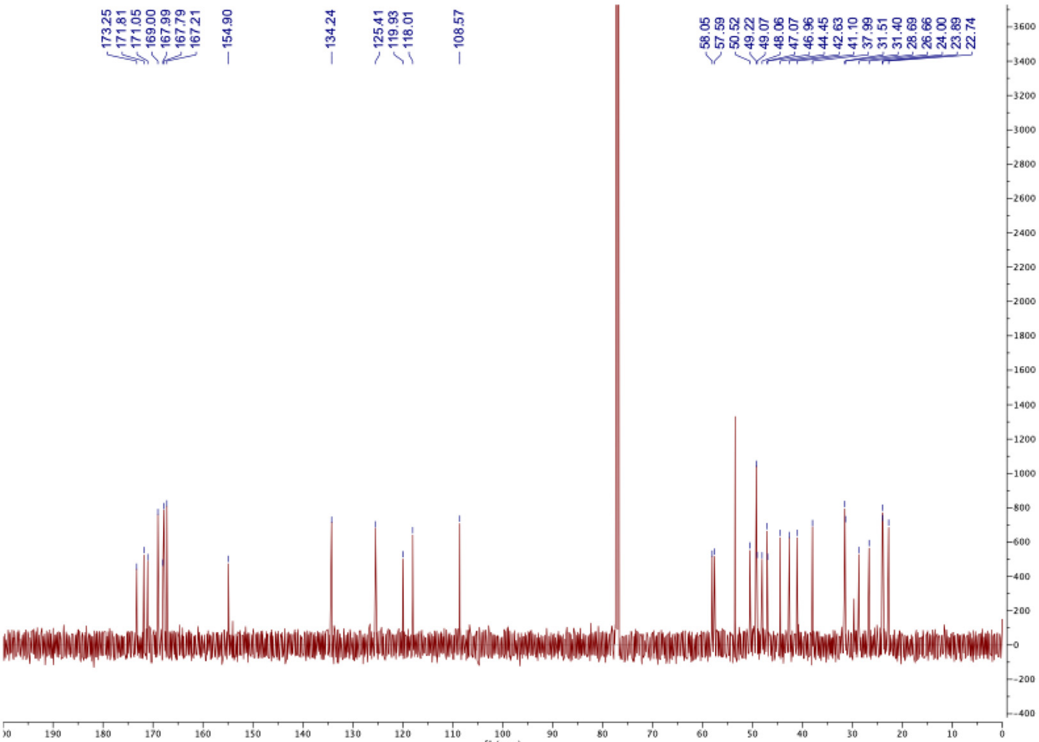
173.25
171.81
171.05
169.00
167.99
167.79
167.21
154.90
134.24
125.41
119.93
118.01
108.57
58.05
57.59
50.52
49.22
49.07
48.06
47.07
46.96
44.45
42.63
41.10
37.99
31.51
31.40
28.69
26.66
24.00
23.89
22.74
f1 (ppm)

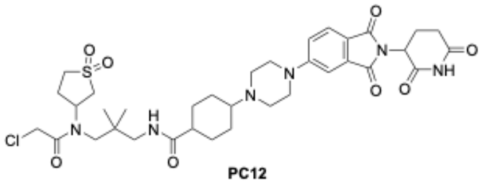

PC12

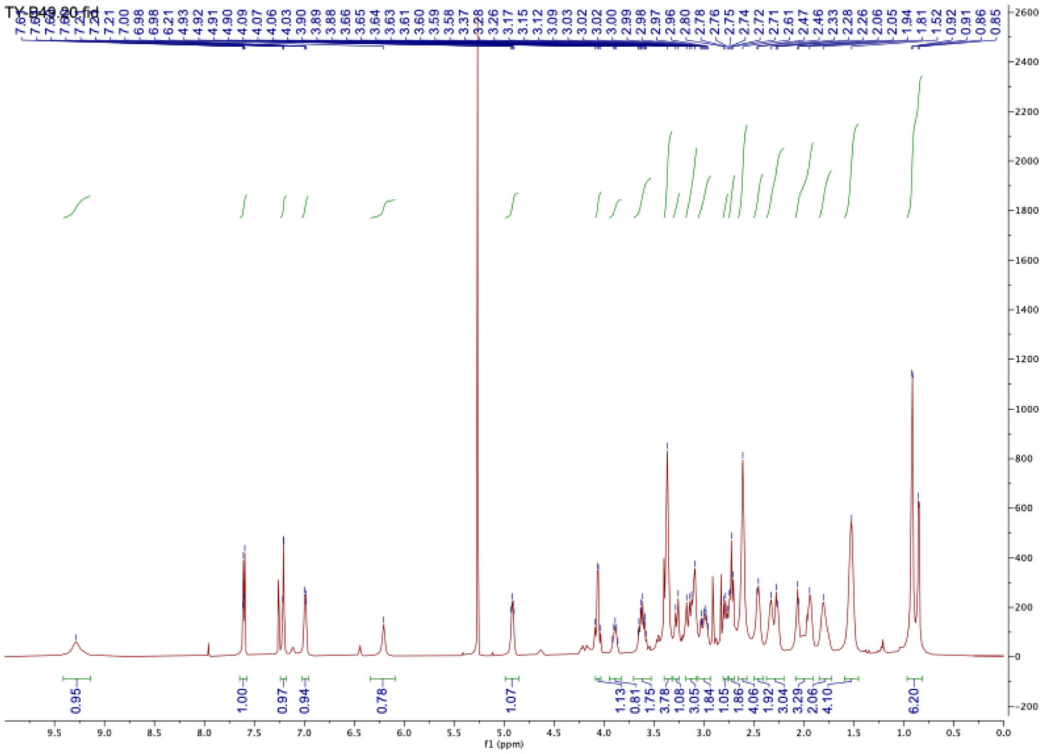
TY-B49.20.fid
7.21
7.00
6.98
6.98
6.21
4.93
4.92
4.91
4.90
4.09
4.07
4.06
4.03
3.90
3.89
3.88
3.66
3.65
3.64
3.63
3.61
3.60
3.59
3.58
3.37
3.28
3.26
3.17
3.15
3.12
3.09
3.03
3.02
3.02
3.00
2.99
2.98
2.97
2.96
2.80
2.78
2.76
2.75
2.74
2.72
2.71
2.61
2.47
2.46
2.33
2.28
2.26
2.06
2.05
1.94
1.81
1.52
0.92
0.91
0.86
0.85
0.95
1.00
0.97
0.94
0.78
1.07
1.13
0.81
1.75
3.78
1.08
3.05
1.84
1.05
1.86
4.06
1.92
3.04
3.29
2.06
4.10
6.20
f1 (ppm)

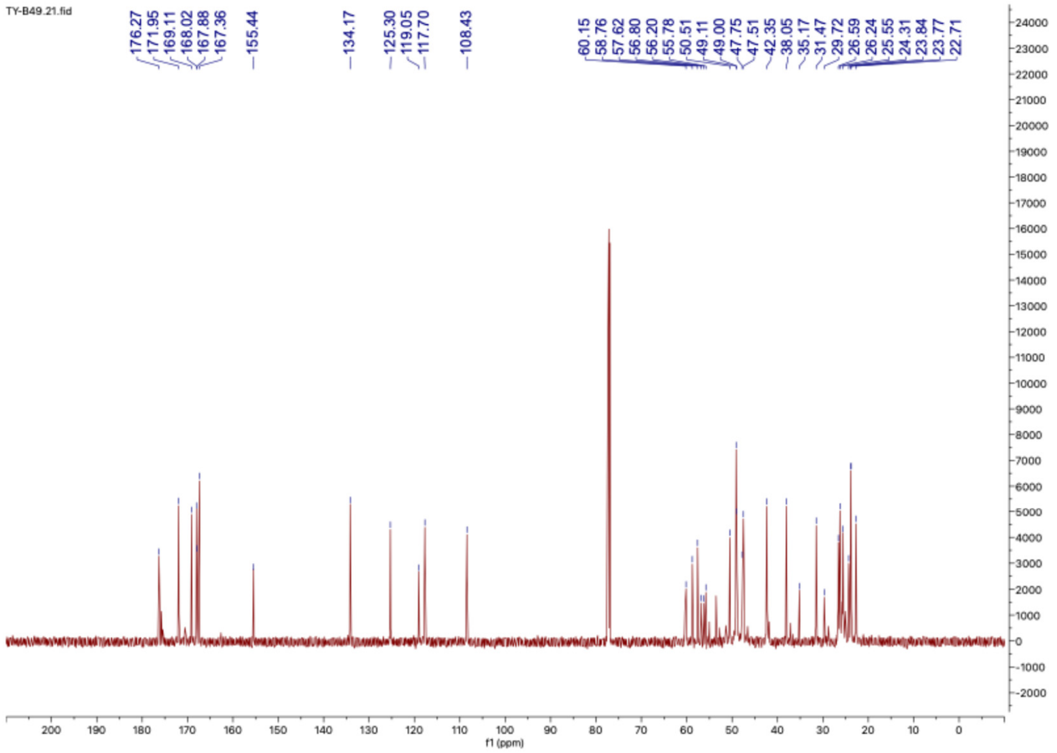
TY-B49.21.fid
176.27
171.95
169.11
168.02
167.88
167.36
155.44
134.17
125.30
119.05
117.70
108.43
60.15
58.76
57.62
56.80
56.20
55.78
50.51
49.11
49.00
47.75
47.51
42.35
38.05
35.17
31.47
29.72
26.59
26.24
25.55
24.31
23.84
23.77
22.71
f1 (ppm)

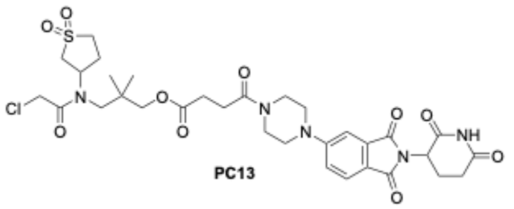

PC13

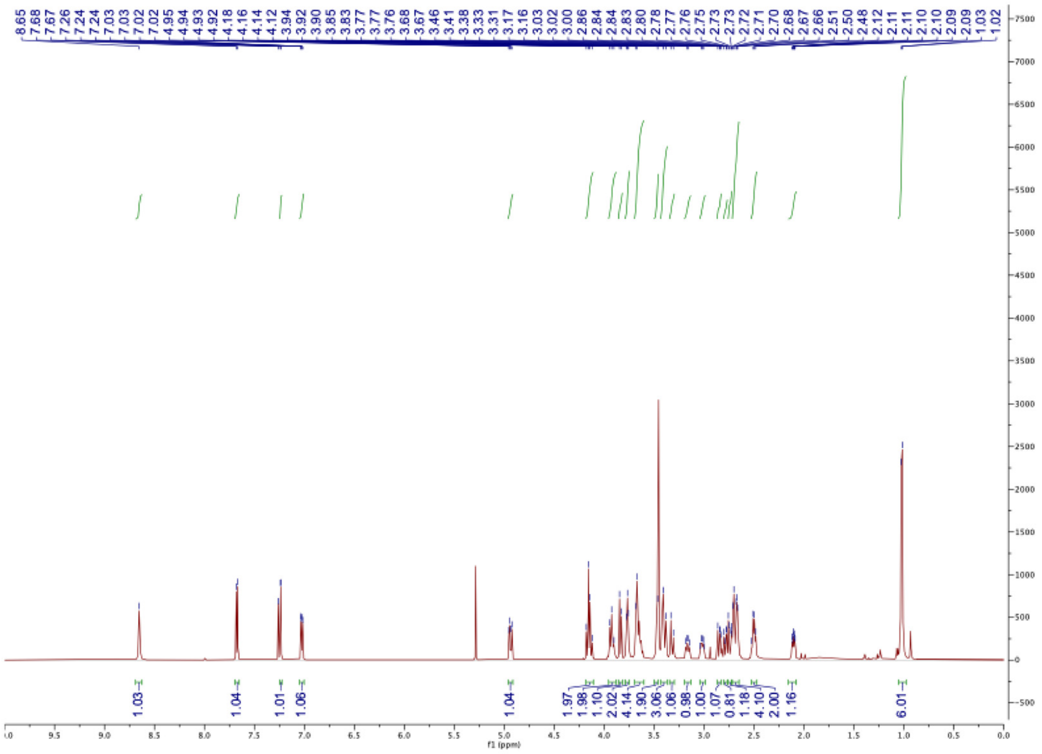
8.65
7.68
7.67
7.26
7.24
7.24
7.03
7.03
7.02
7.02
4.95
4.94
4.93
4.92
4.18
4.16
4.14
4.12
3.94
3.92
3.90
3.85
3.83
3.77
3.76
3.68
3.67
3.46
3.41
3.38
3.33
3.31
3.17
3.16
3.03
3.02
3.00
2.86
2.84
2.84
2.83
2.80
2.78
2.77
2.76
2.75
2.73
2.73
2.72
2.71
2.70
2.68
2.67
2.66
2.51
2.50
2.48
2.12
2.11
2.11
2.10
2.10
2.09
1.03
1.02
1.03
1.04
1.01
1.06
1.04
1.97
1.98
1.10
2.02
4.14
1.90
3.06
1.06
0.98
1.00
1.07
0.81
1.18
4.10
2.00
1.16
6.01
f1 (ppm)

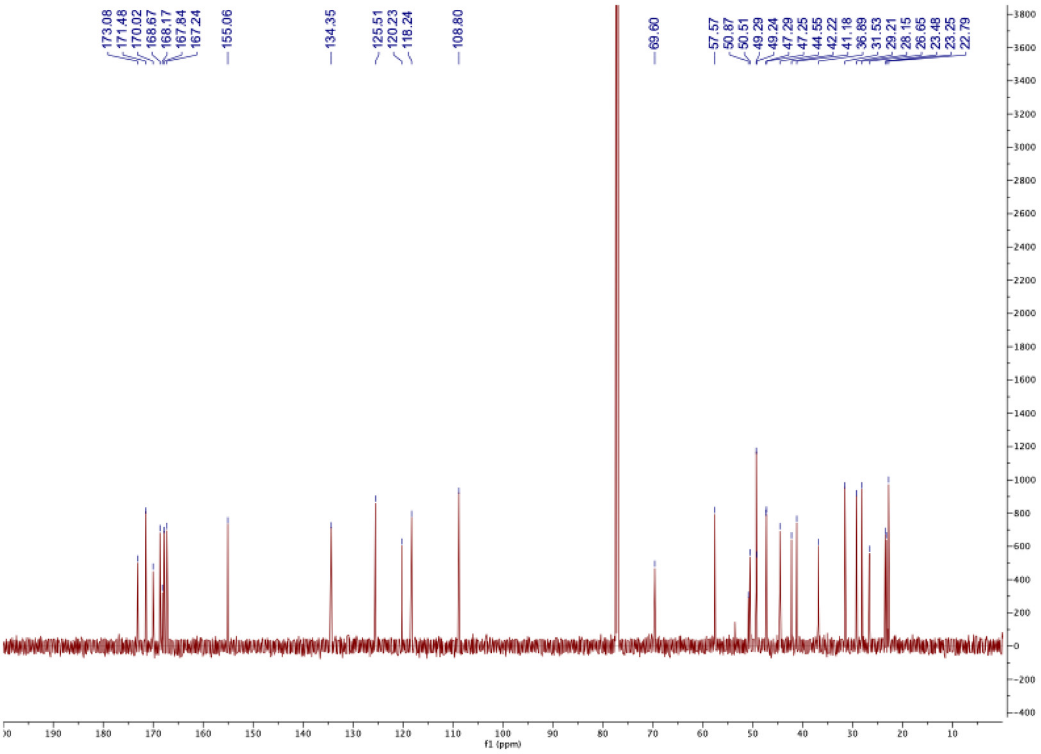
173.08
171.48
170.02
168.67
168.17
167.84
167.24
155.06
134.35
125.51
120.23
118.24
108.80
69.60
57.57
50.87
50.51
49.29
49.24
47.29
47.25
44.55
42.22
41.18
36.89
31.53
29.21
28.15
26.65
23.48
23.25
22.79
f1 (ppm)

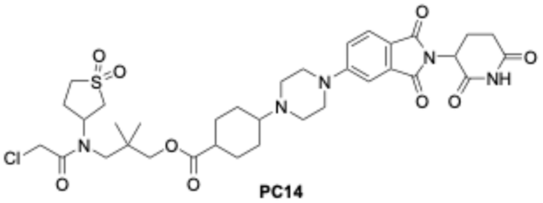

PC14

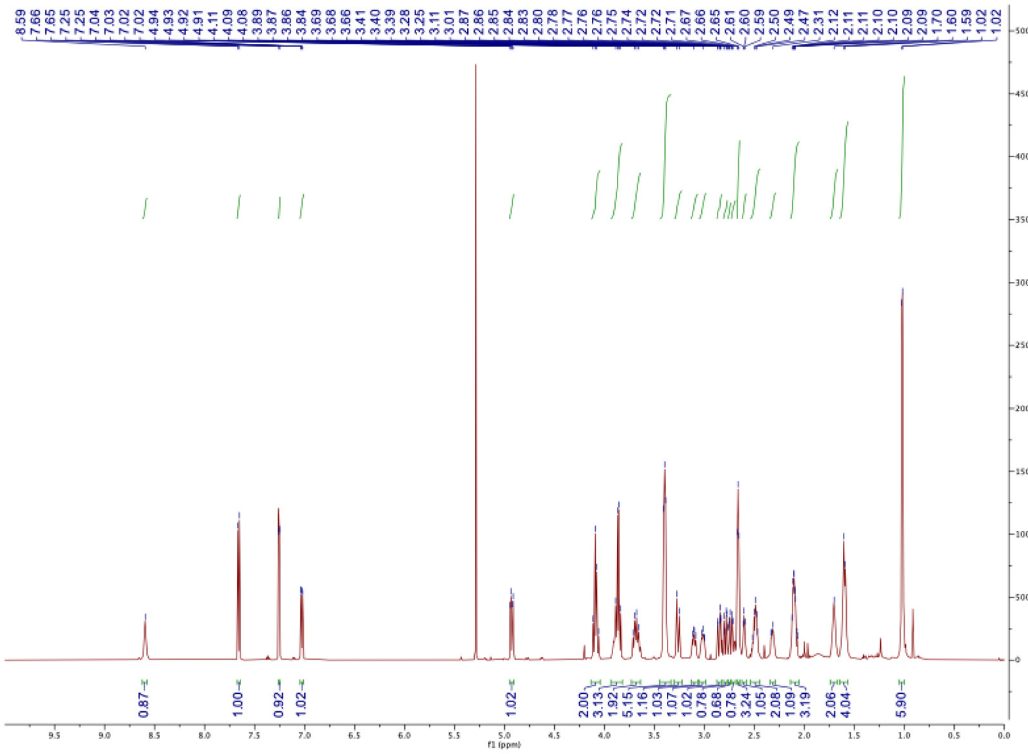

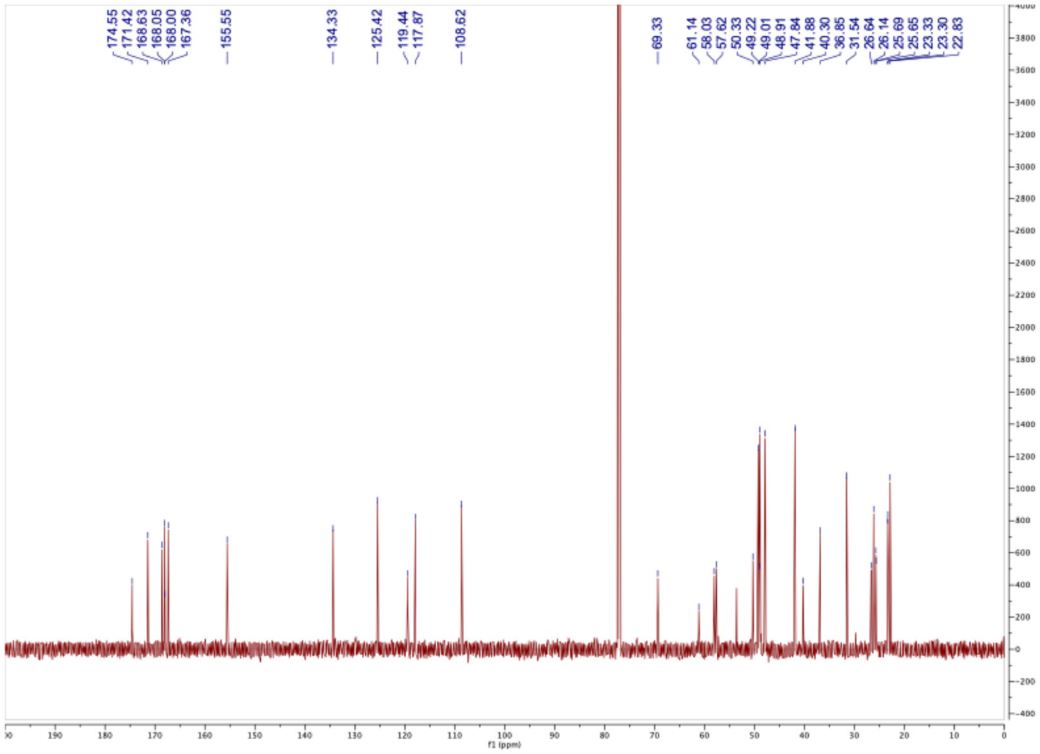

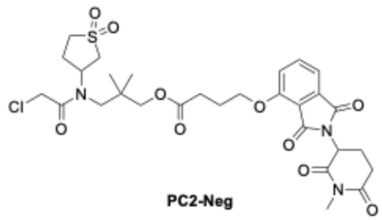

PC2-Neg

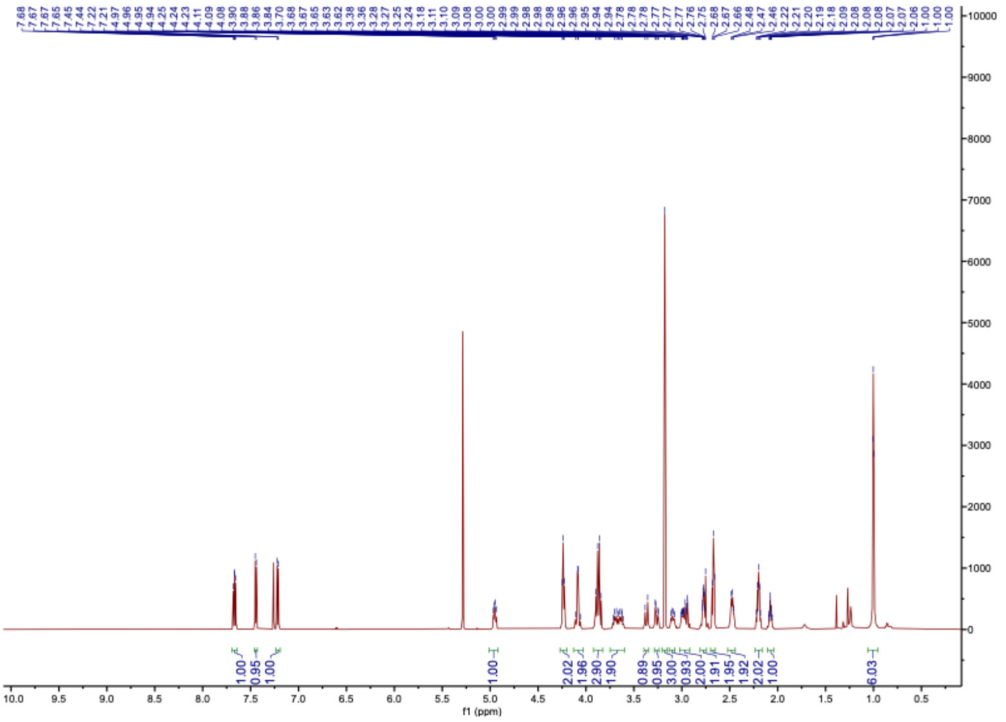
7.68 7.67 7.65 7.45 7.44 7.22 7.21 4.97 4.96 4.95 4.94 4.25 4.24 4.23 4.11 4.09 4.08 3.90 3.88 3.86 3.84 3.70 3.68 3.67 3.65 3.63 3.62 3.38 3.36 3.28 3.27 3.25 3.24 3.18 3.11 3.10 3.09 3.08 3.00 2.99 2.99 2.98 2.98 2.98 2.98 2.96 2.96 2.95 2.94 2.94 2.94 2.78 2.78 2.78 2.77 2.77 2.77 2.76 2.75 2.68 2.67 2.66 2.48 2.47 2.46 2.22 2.21 2.20 2.19 2.18 2.09 2.08 2.08 2.08 2.08 2.07 2.06 1.00 1.00
1.00 0.95 1.00 1.00 2.02 1.96 2.90 1.90 0.89 0.95 3.00 0.93 2.00 1.91 1.95 1.92 2.02 1.00 6.03
f1 (ppm)

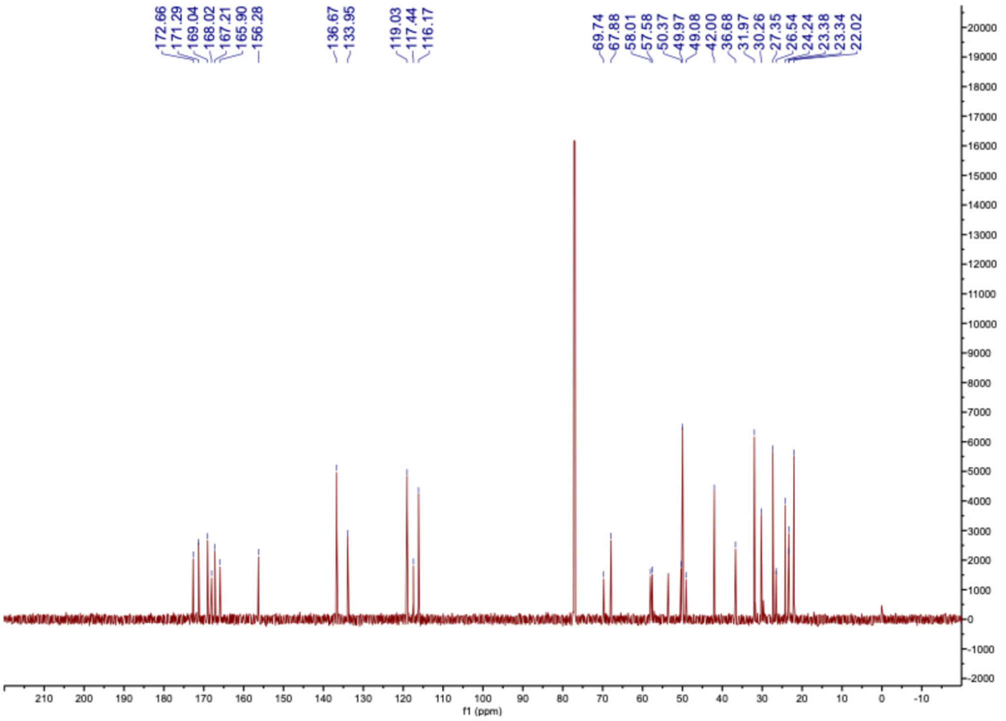
172.66 171.29 169.04 168.02 167.21 165.90 156.28
136.67 133.95
119.03 117.44 116.17
69.74 67.88 58.01 57.58 50.37 49.97 49.08 42.00 36.68 31.97 30.26 27.35 26.54 24.24 23.38 23.34 22.02
f1 (ppm)

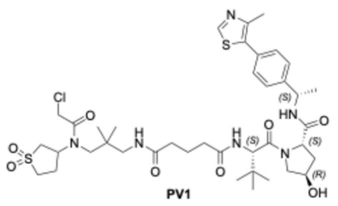

Cl
O
N
O
S
O
H
N
O
O
H
N
(S)
O
N
(S)
HN
O
(S)
(R)
OH
N
S

PV1

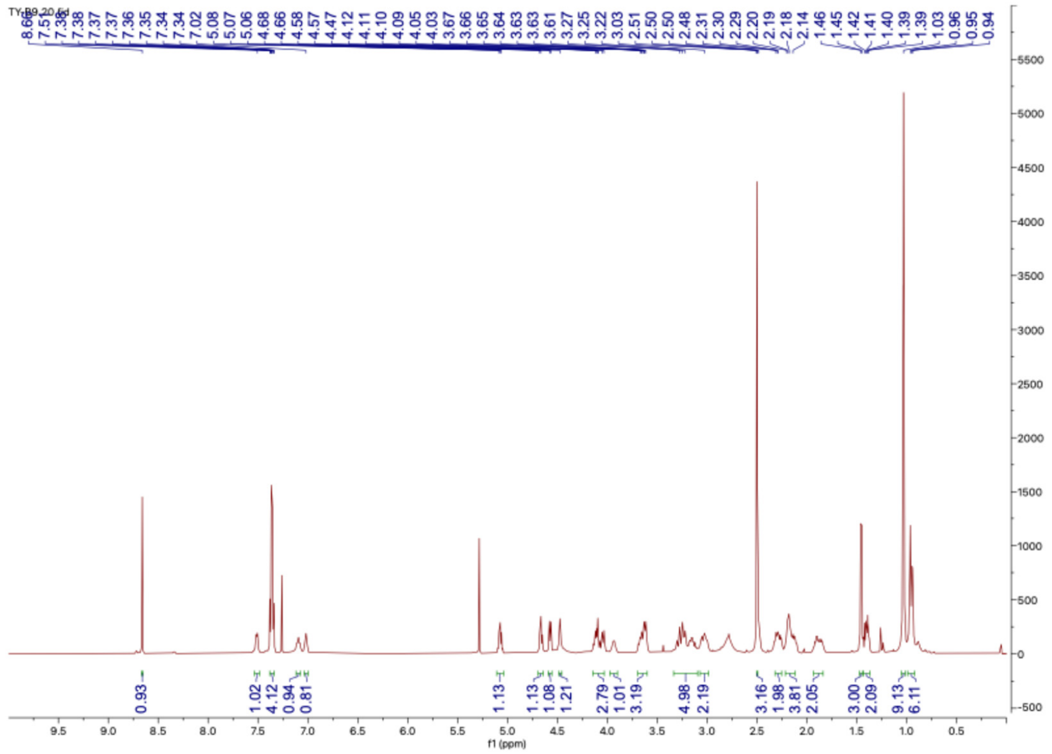

TY-B9.20.fid
8.66
7.51
7.38
7.38
7.37
7.37
7.36
7.35
7.34
7.34
7.02
5.08
5.07
5.06
4.68
4.66
4.58
4.57
4.47
4.12
4.11
4.10
4.09
4.05
4.03
3.67
3.66
3.65
3.64
3.63
3.63
3.61
3.27
3.25
3.22
3.03
3.03
2.51
2.50
2.50
2.48
2.31
2.30
2.29
2.20
2.19
2.18
2.14
1.46
1.45
1.42
1.41
1.40
1.39
1.39
1.03
0.96
0.95
0.94
0.93
1.02
4.12
0.94
0.81
1.13
1.13
1.08
1.21
2.79
1.01
3.19
4.98
2.19
3.16
1.98
3.81
2.05
3.00
2.09
9.13
6.11
f1 (ppm)

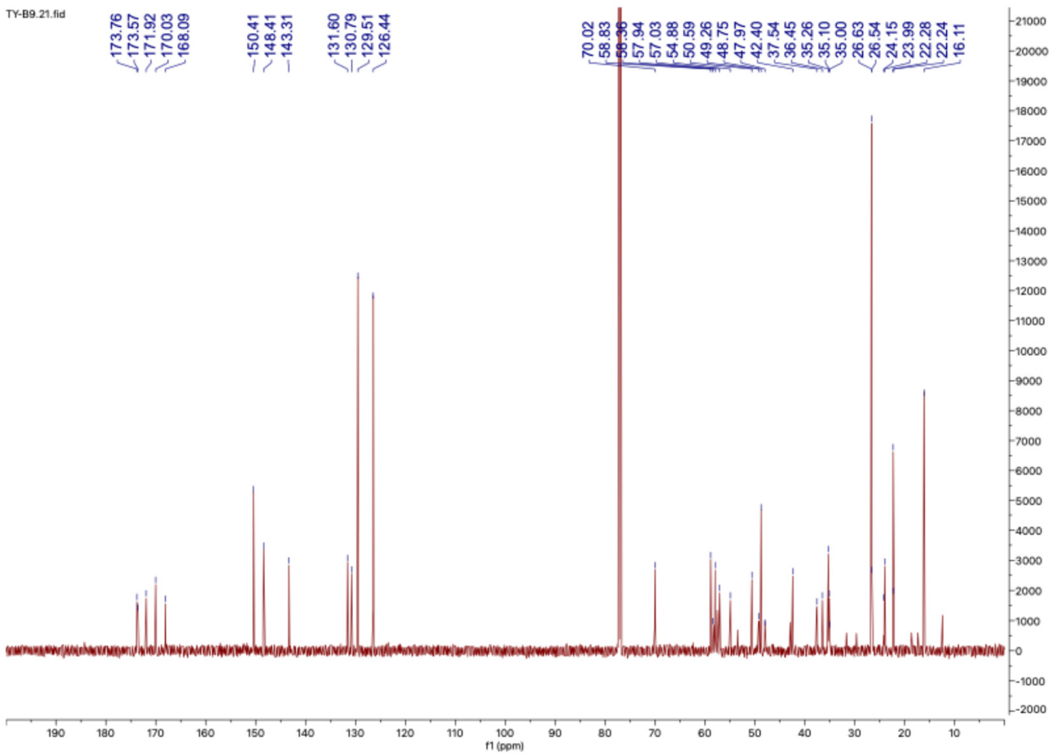

TY-B9.21.fid
173.76
173.57
171.92
170.03
168.09
150.41
148.41
143.31
131.60
130.79
129.51
126.44
70.02
58.83
58.36
57.94
57.03
54.88
50.59
49.26
48.75
47.97
42.40
37.54
36.45
35.26
35.10
35.00
26.63
26.54
24.15
23.99
22.28
22.24
16.11
f1 (ppm)

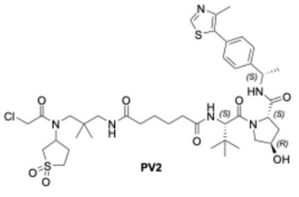

PV2

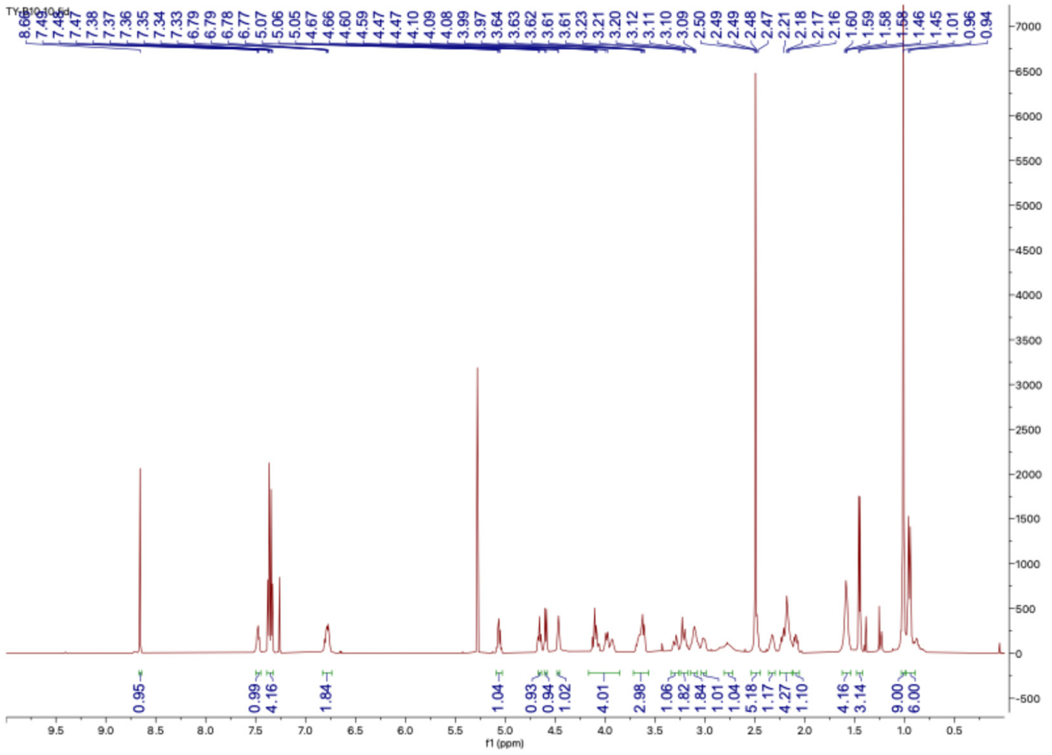

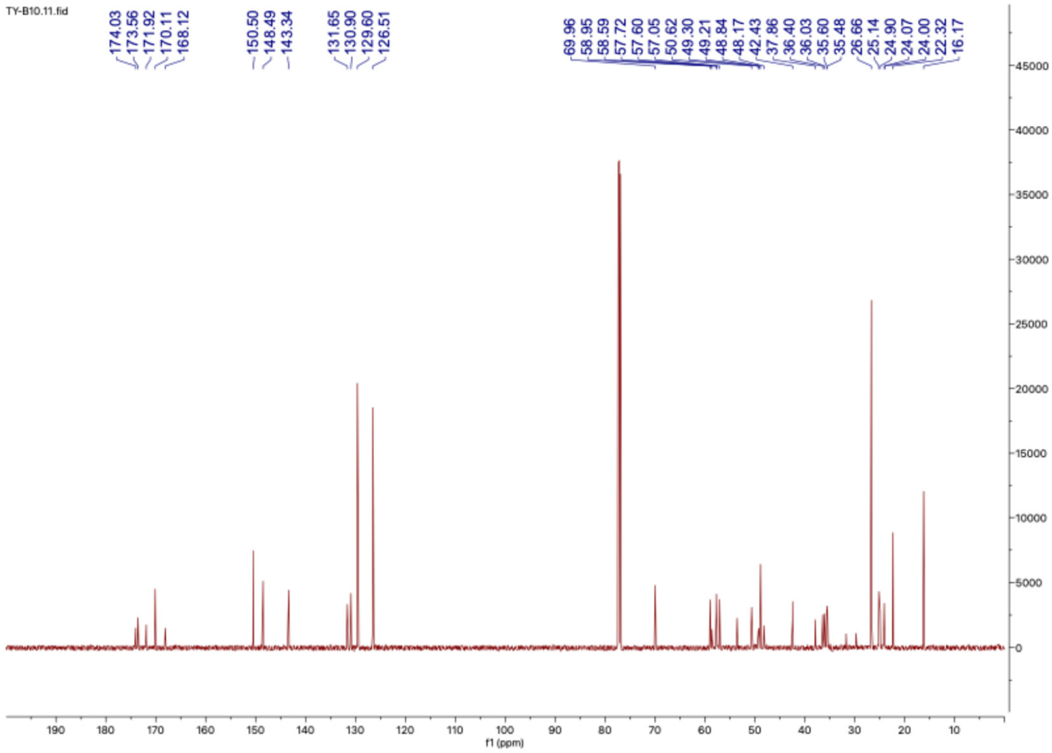

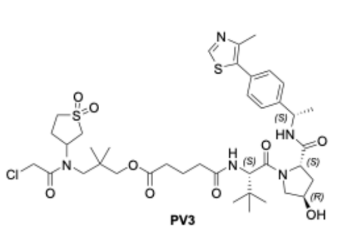
Cl
N
O
S
O
O
H
N
HO
(S)
(R)
OH
HN
S
N

PV3

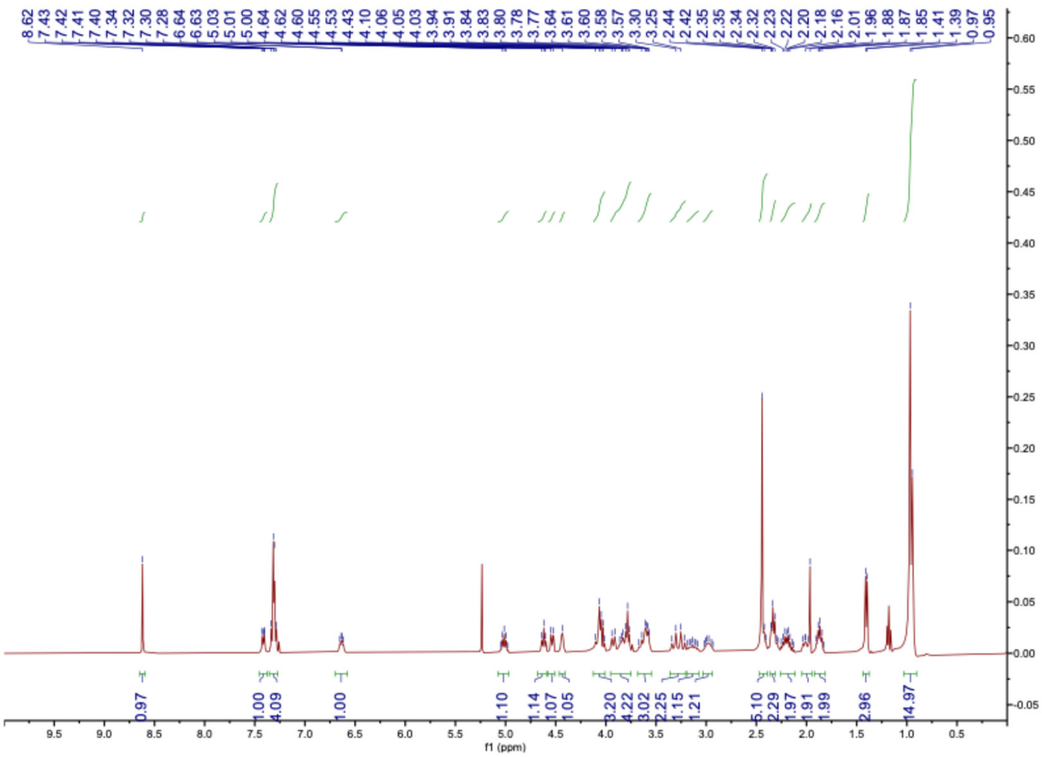
8.62
7.43
7.42
7.41
7.40
7.34
7.32
7.30
7.28
6.64
6.63
5.03
5.01
5.00
4.64
4.62
4.60
4.55
4.53
4.43
4.10
4.06
4.05
4.03
3.94
3.91
3.84
3.83
3.80
3.78
3.77
3.64
3.61
3.60
3.58
3.57
3.30
3.25
2.44
2.42
2.35
2.35
2.34
2.32
2.23
2.22
2.20
2.18
2.16
2.01
1.96
1.88
1.87
1.85
1.41
1.39
0.97
0.95
0.97
1.00
4.09
1.00
1.10
1.14
1.07
1.05
3.20
4.22
3.02
2.25
1.15
1.21
5.10
2.29
1.97
1.91
1.99
2.96
14.97
f1 (ppm)

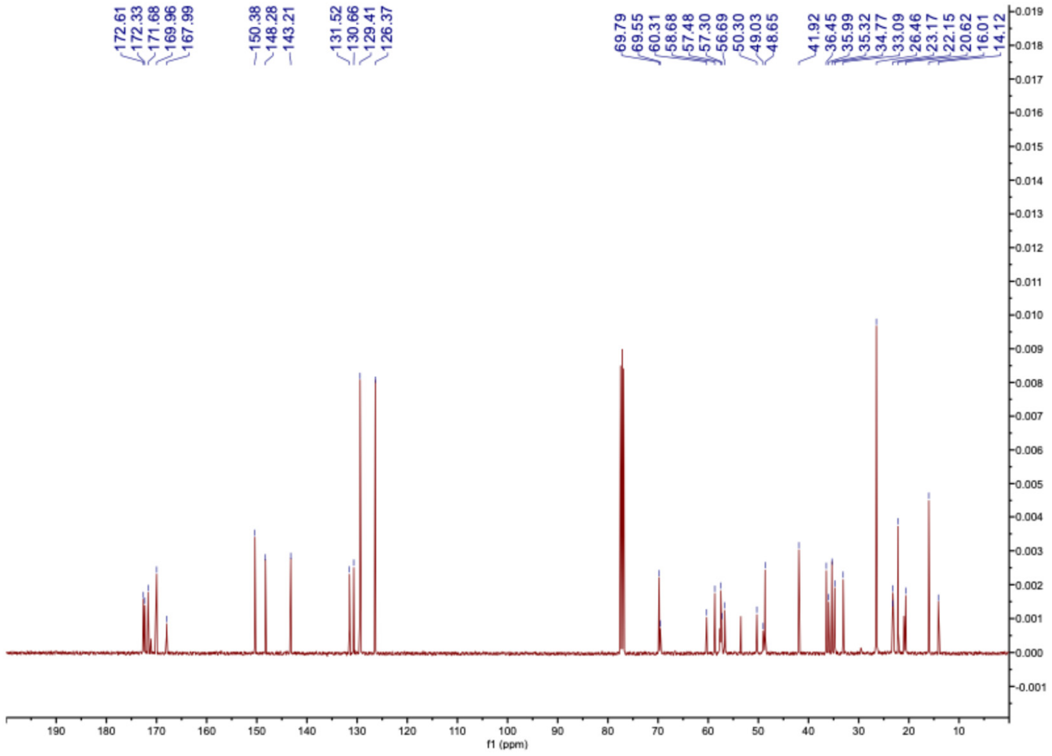
172.61
172.33
171.68
169.96
167.99
150.38
148.28
143.21
131.52
130.66
129.41
126.37
69.79
69.55
60.31
58.68
57.48
57.30
56.69
50.30
49.03
48.65
41.92
36.45
35.99
35.32
34.77
33.09
26.46
23.17
22.15
20.62
16.01
14.12
f1 (ppm)

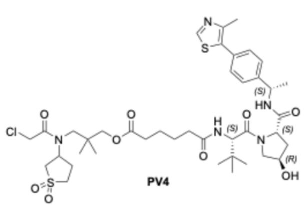

PV4

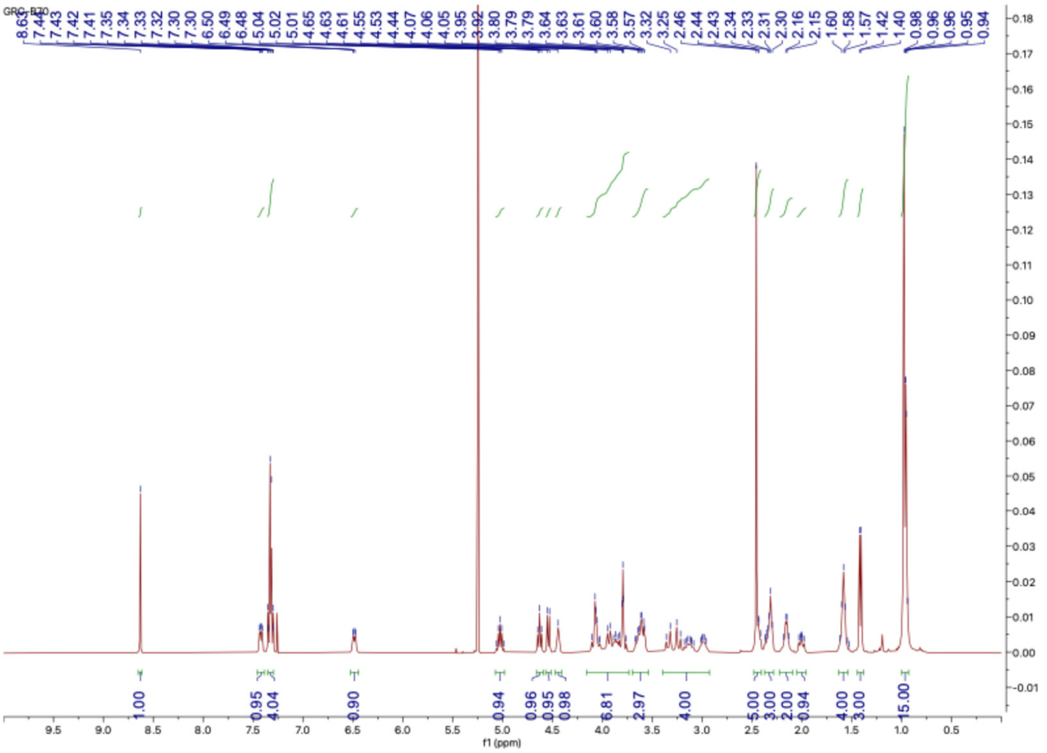

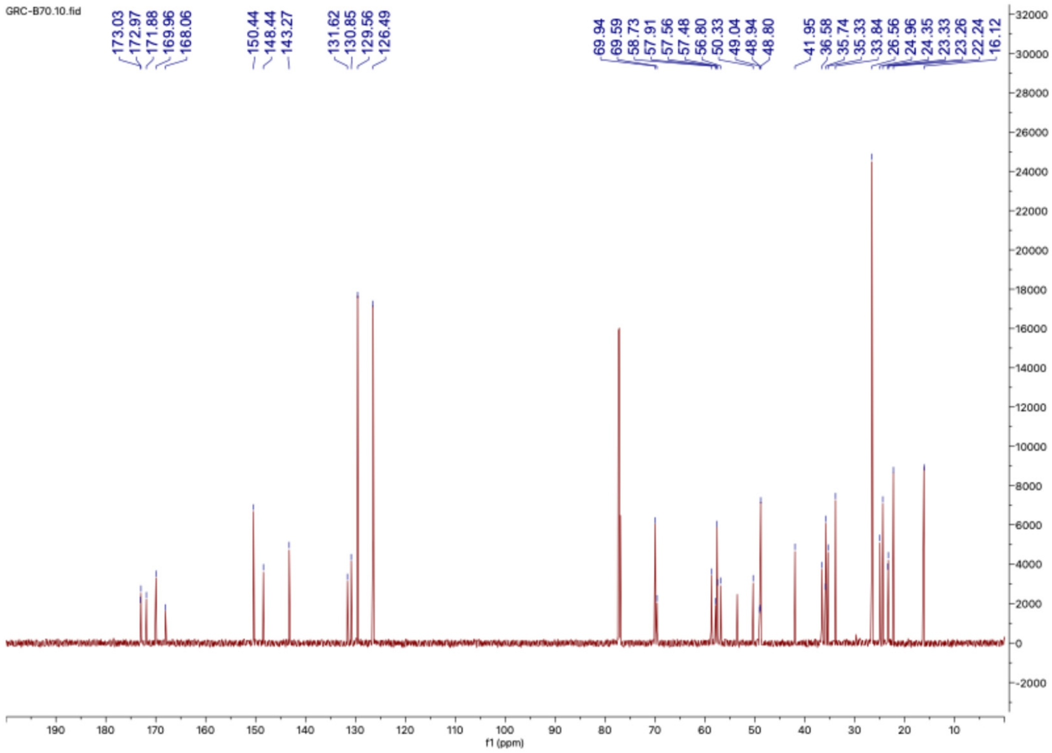

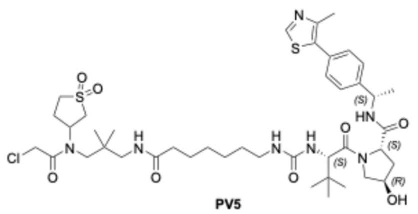

PV5

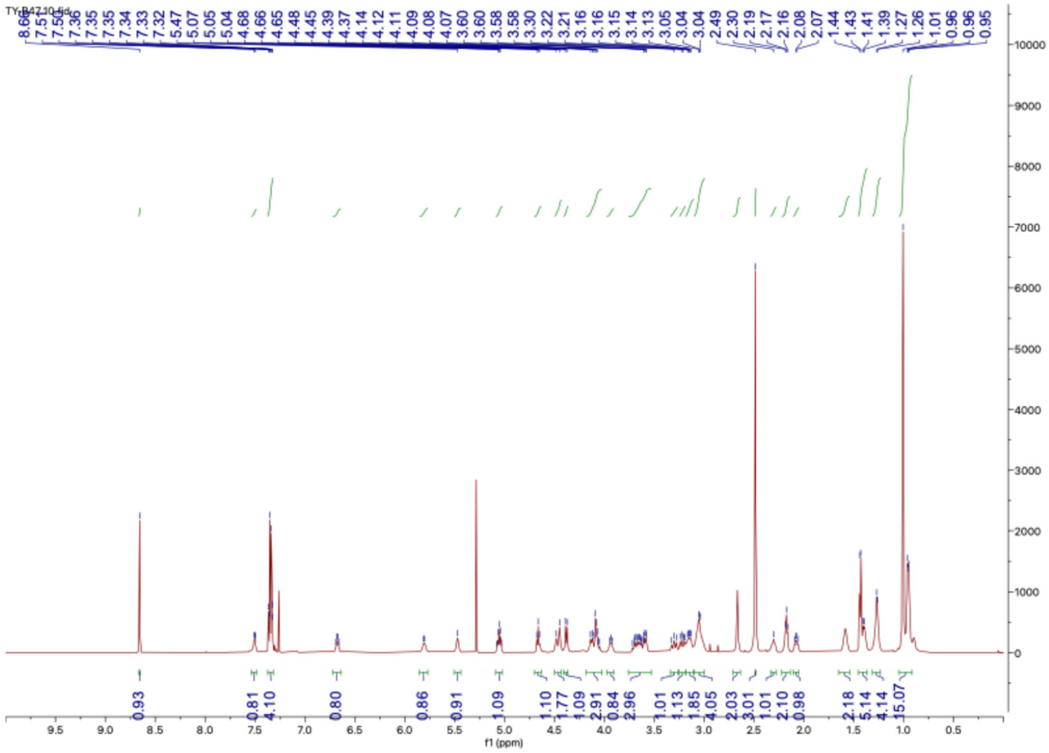

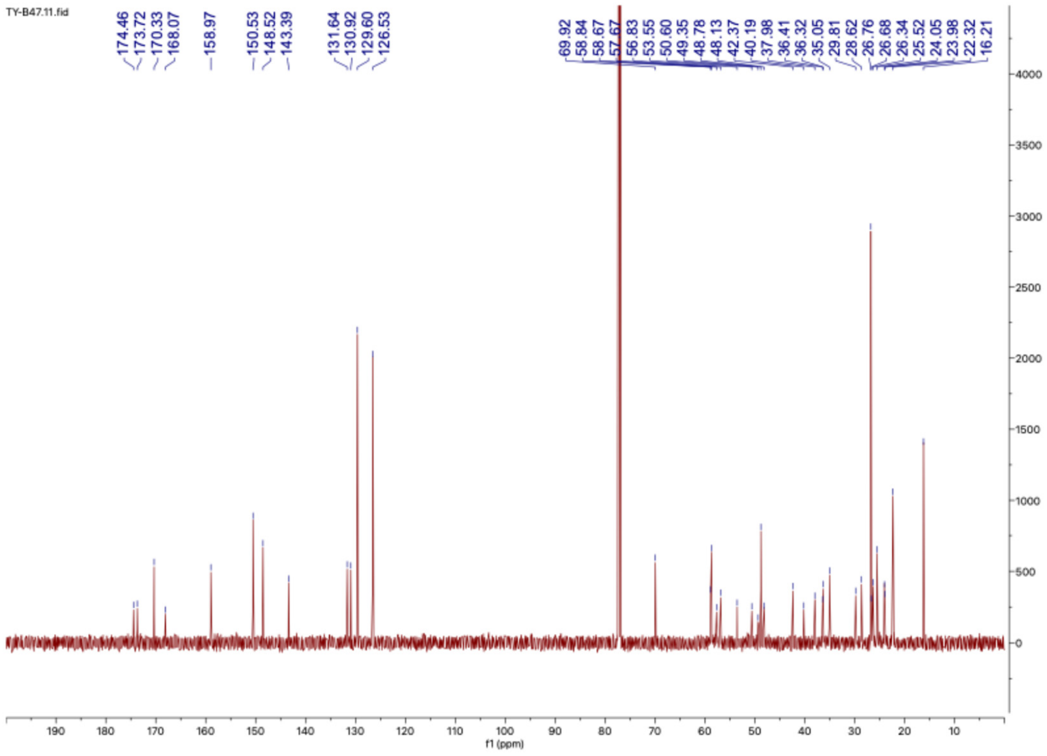

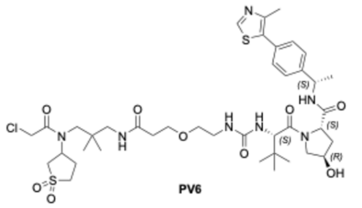

PV6

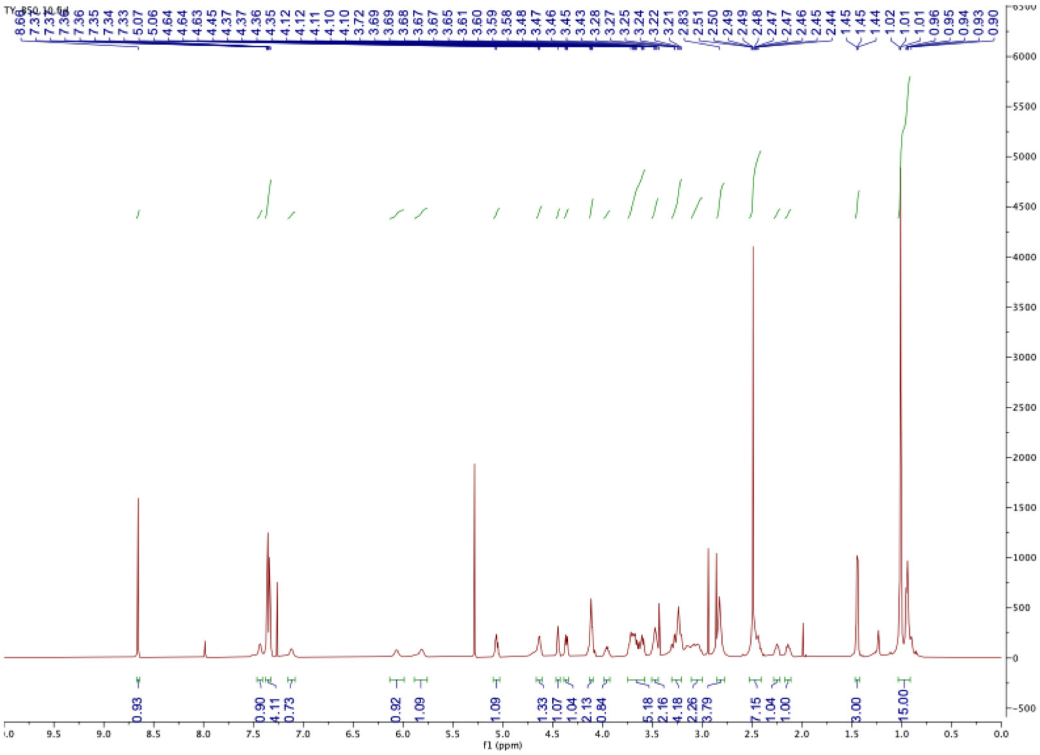
7.36
7.35
7.34
7.33
5.07
5.06
4.64
4.64
4.63
4.45
4.37
4.37
4.36
4.35
4.12
4.12
4.11
4.10
3.72
3.69
3.69
3.68
3.67
3.67
3.65
3.61
3.60
3.59
3.58
3.48
3.47
3.46
3.45
3.43
3.28
3.27
3.25
3.24
3.22
3.21
2.83
2.51
2.50
2.49
2.49
2.48
2.47
2.47
2.46
2.45
2.44
1.45
1.45
1.44
1.02
1.01
1.01
0.96
0.95
0.94
0.93
0.90
0.93
0.90
4.11
0.73
0.92
1.09
1.09
1.33
1.07
1.04
2.13
0.84
5.18
2.16
4.18
2.26
3.79
7.15
1.04
1.00
3.00
15.00
f1 (ppm)

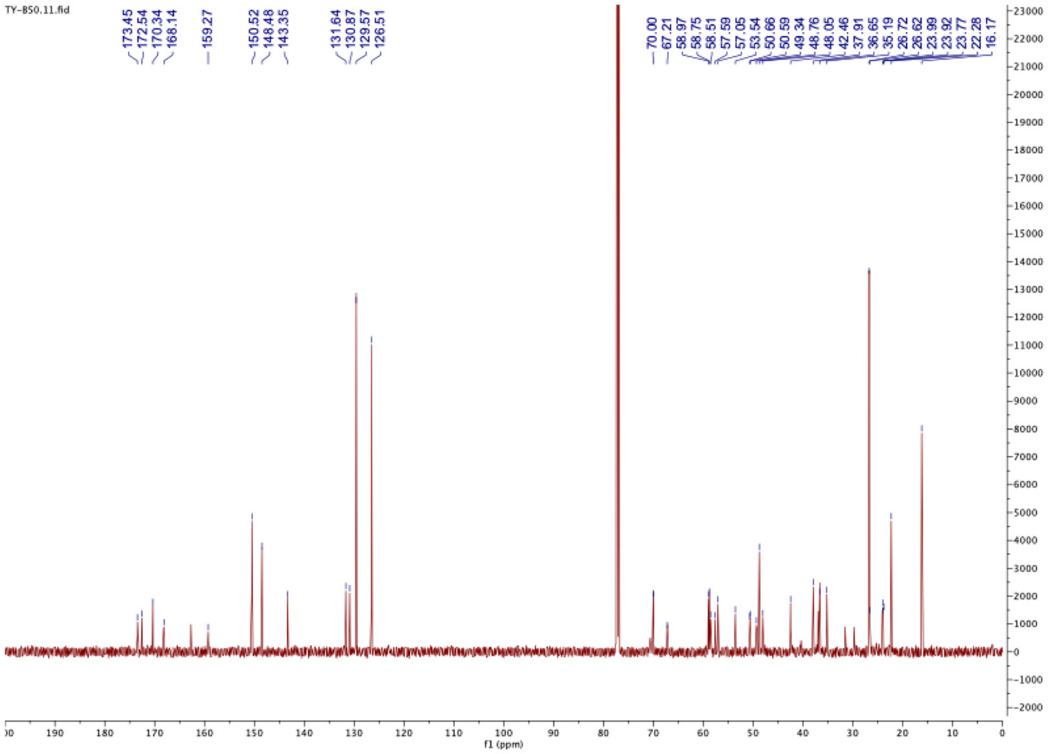
TY-B50.11.fid
173.45
172.54
170.34
168.14
159.27
150.52
148.48
143.35
131.64
130.87
129.57
126.51
70.00
67.21
58.97
58.75
58.51
57.59
57.05
53.54
50.66
50.59
49.34
48.76
48.05
42.46
37.91
36.65
35.19
26.72
26.62
23.99
23.92
23.77
22.28
16.17
f1 (ppm)

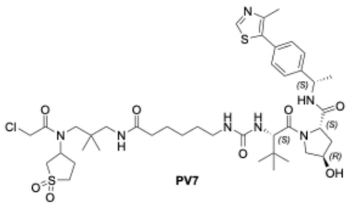

PV7

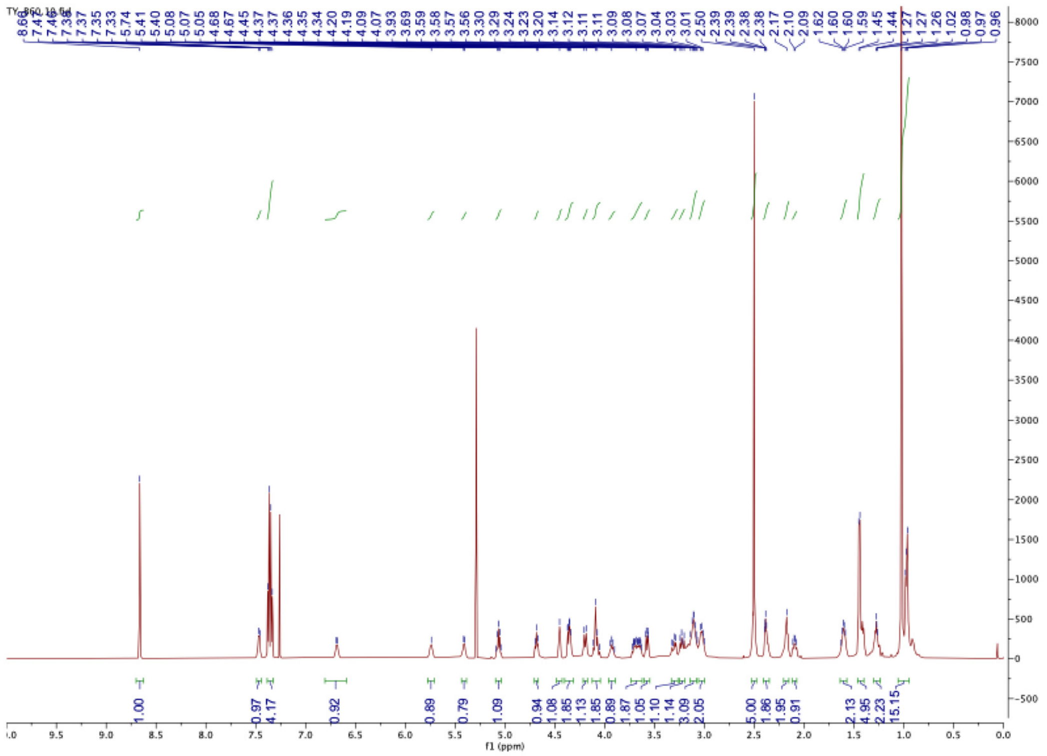

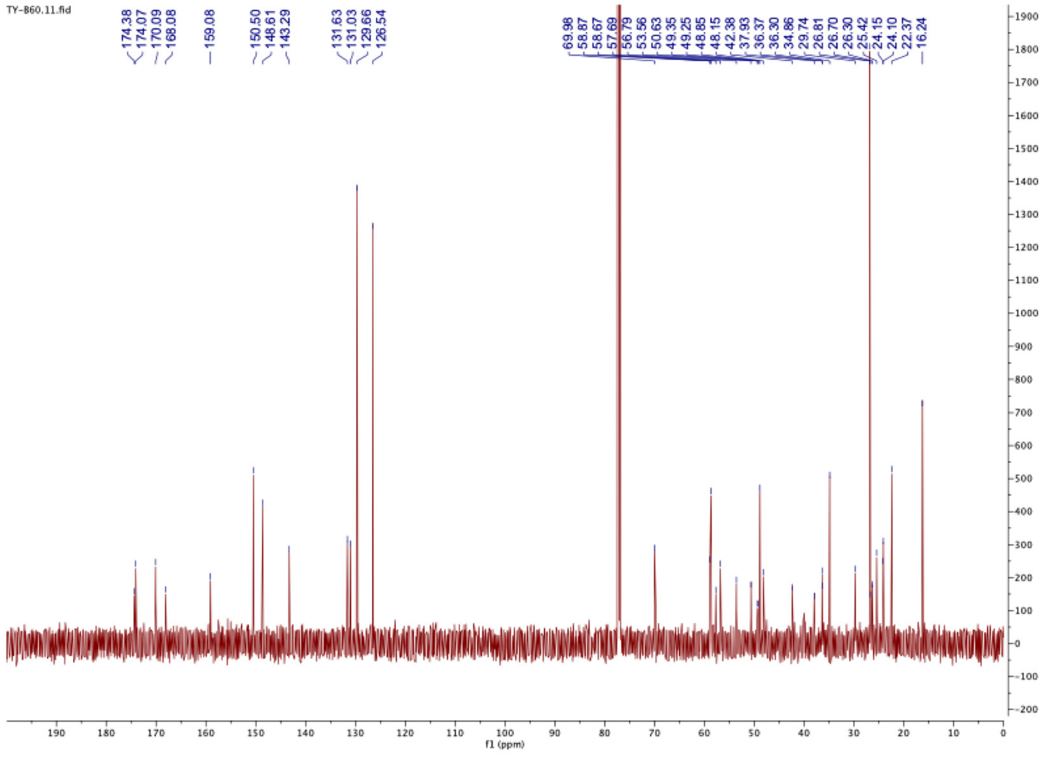

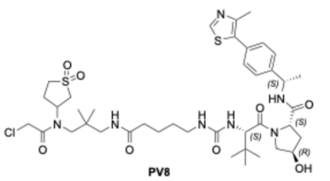

Cl
O
N
S
O
O
H
N
O
H
N
H
N
O
O
(S)
N
(S)
HN
O
(S)
(R)
OH
N
S

PV8

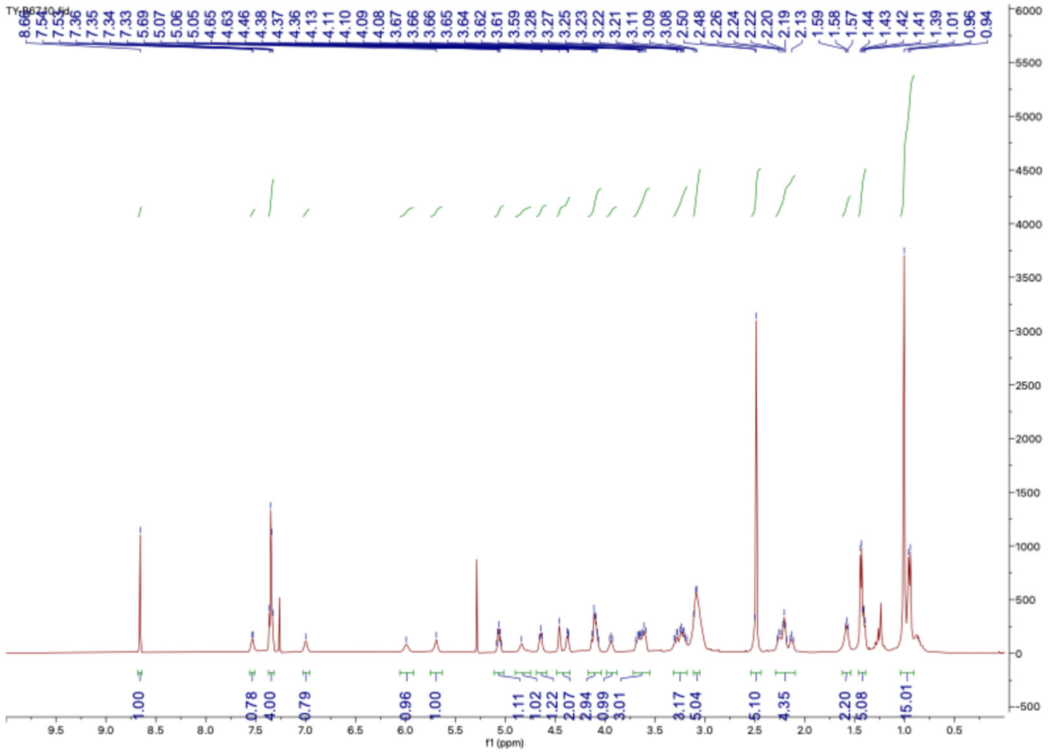

7.36
7.35
7.34
7.33
5.69
5.07
5.06
5.05
4.65
4.63
4.46
4.38
4.37
4.36
4.13
4.11
4.10
4.09
4.08
3.67
3.66
3.66
3.65
3.64
3.62
3.61
3.59
3.28
3.27
3.25
3.23
3.22
3.21
3.11
3.09
3.08
2.50
2.48
2.26
2.24
2.22
2.20
2.19
2.13
1.59
1.58
1.57
1.44
1.43
1.42
1.41
1.39
1.01
0.96
0.94
1.00
0.78
4.00
0.79
0.96
1.00
1.11
1.02
1.22
2.07
2.94
0.99
3.01
3.17
5.04
5.10
4.35
2.20
5.08
15.01
f1 (ppm)

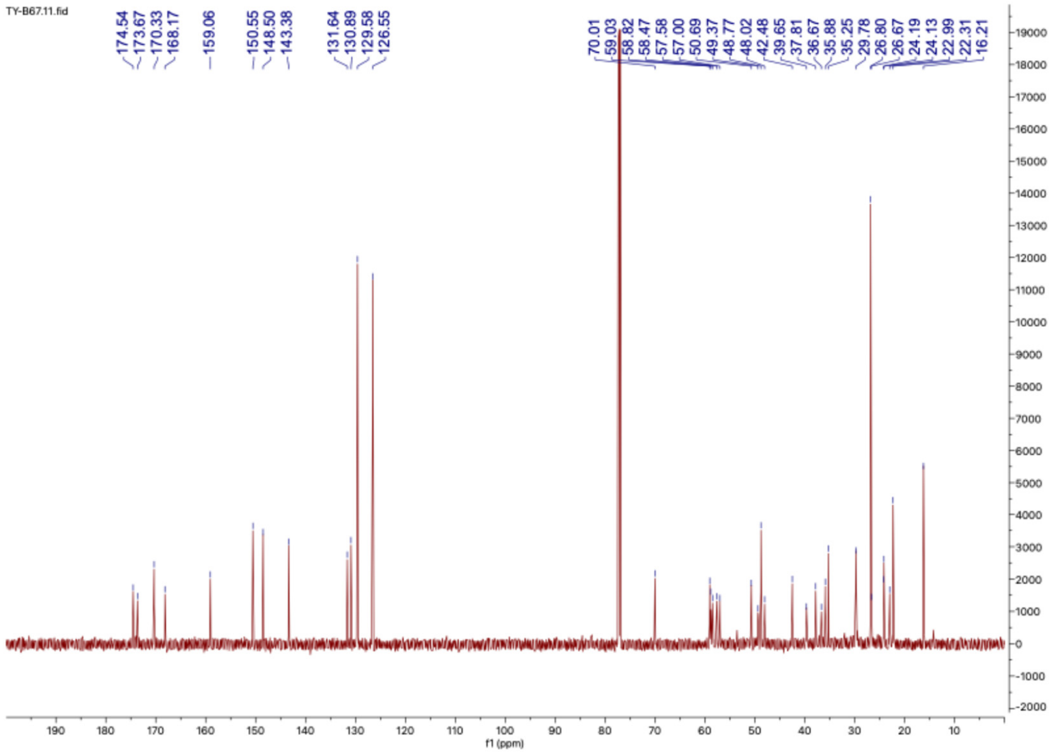

TY-B67.11.fid
174.54
173.67
170.33
168.17
159.06
150.55
148.50
143.38
131.64
130.89
129.58
126.55
70.01
59.03
58.82
58.47
57.58
57.00
50.69
49.37
48.77
48.02
42.48
39.65
37.81
36.67
35.88
35.25
29.78
26.80
26.67
24.19
24.13
22.99
22.31
16.21
f1 (ppm)

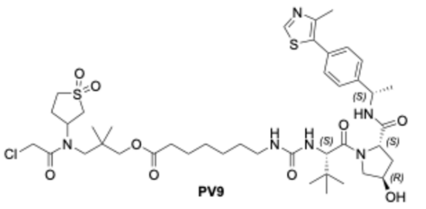

PV9

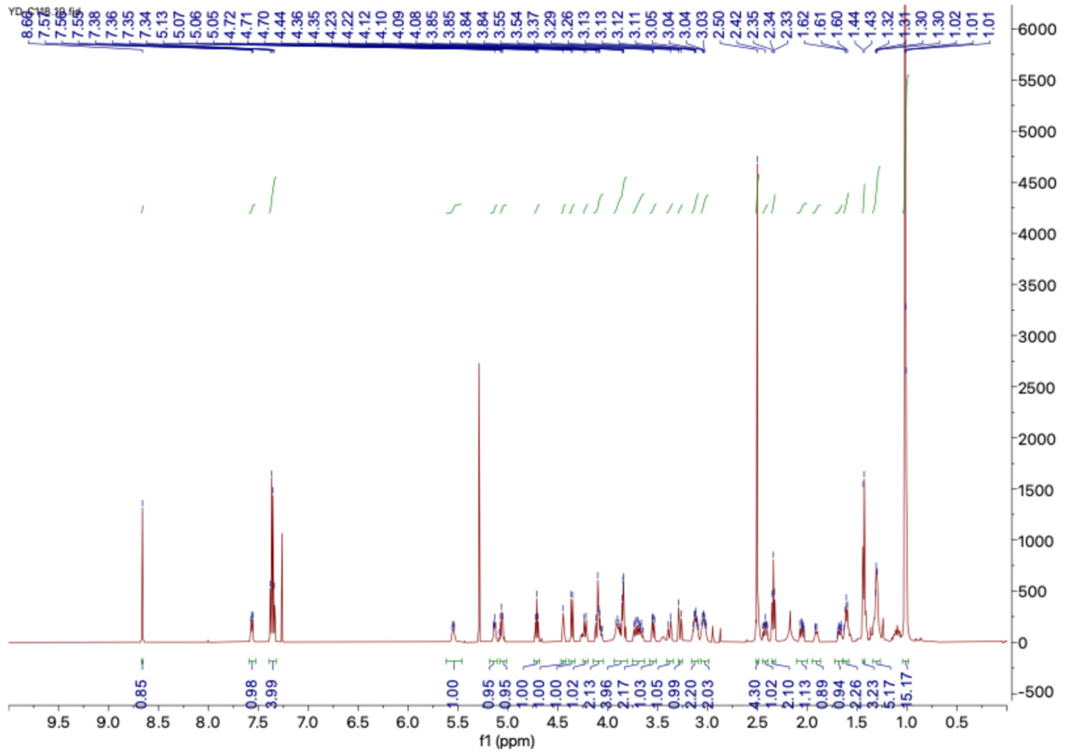

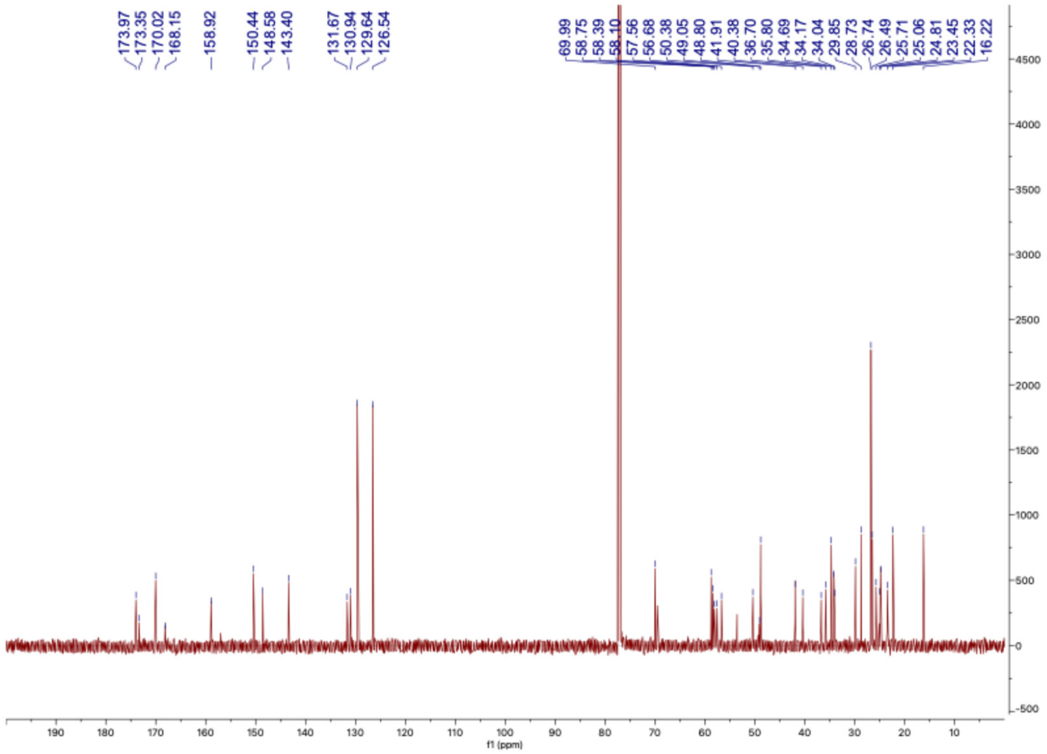

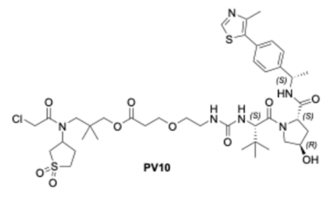
Cl
O
N
O
O
O
O
H
N
N
H
(S)
O
N
(S)
O
HN
(S)
N
S
(R)
OH
S
O
O

PV10

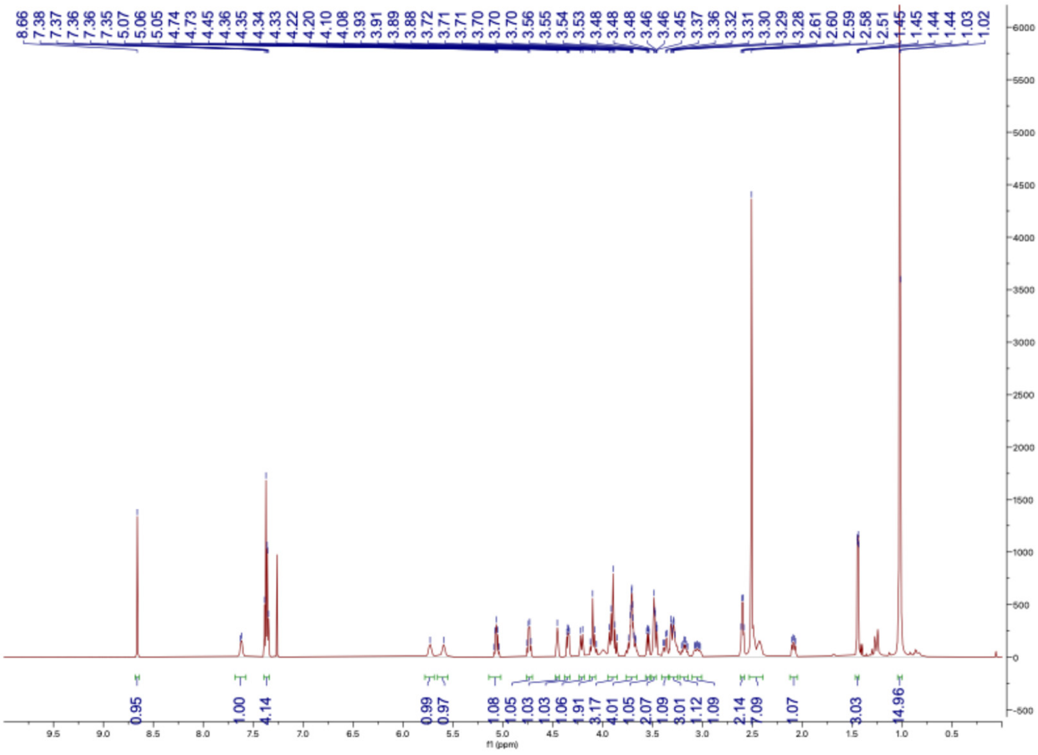
8.66 7.38 7.37 7.36 7.36 7.35 5.07 5.06 5.05 4.74 4.73 4.45 4.36 4.35 4.34 4.33 4.22 4.20 4.10 4.08 3.93 3.91 3.89 3.88 3.72 3.71 3.71 3.70 3.70 3.56 3.55 3.54 3.53 3.48 3.48 3.46 3.46 3.45 3.37 3.36 3.32 3.31 3.30 3.29 3.28 2.61 2.60 2.59 2.58 2.51 1.45 1.45 1.44 1.44 1.03 1.02
0.95 1.00 4.14 0.99 0.97 1.08 1.05 1.03 1.03 1.06 1.91 3.17 4.01 1.05 2.07 1.09 3.01 1.12 1.09 2.14 7.09 1.07 3.03 14.96
f1 (ppm)

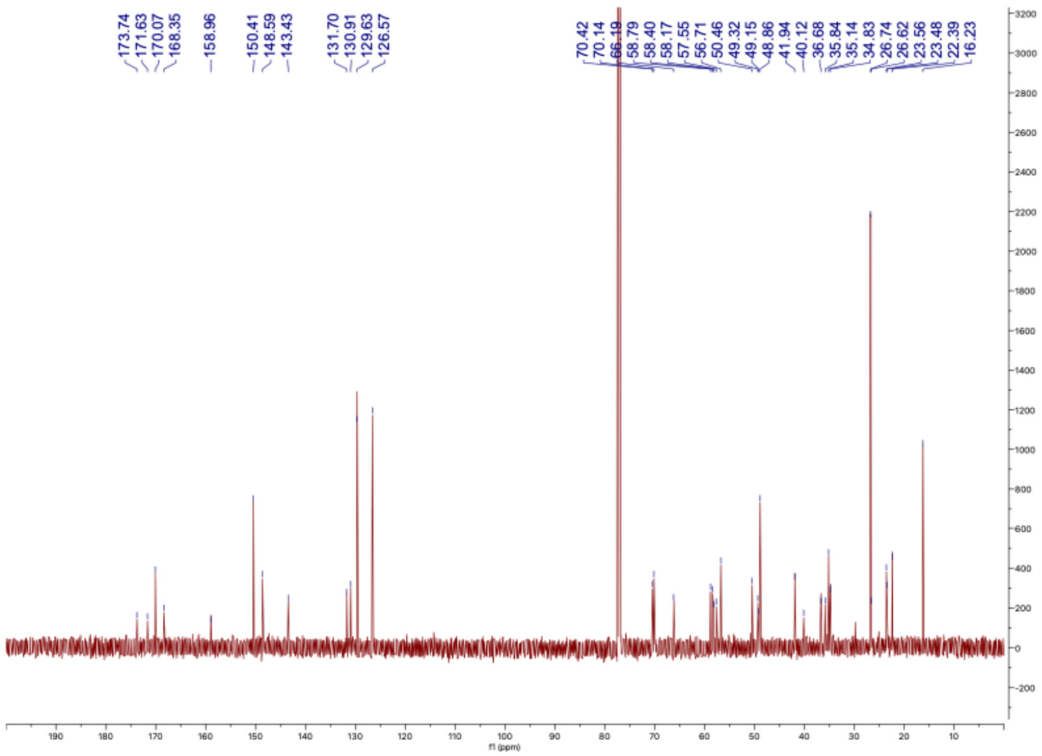
173.74 171.63 170.07 168.35 158.96 150.41 148.59 143.43 131.70 130.91 129.63 126.57
70.42 70.14 66.19 58.79 58.40 58.17 57.55 56.71 50.46 49.32 49.15 48.86 41.94 40.12 36.68 35.84 35.14 34.83 26.74 26.62 23.56 23.48 22.39 16.23
f1 (ppm)

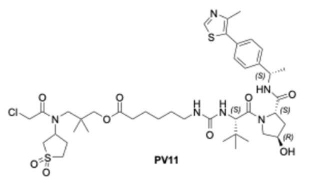
Cl
N
O
S
HN
OH
(S)
(R)

PV11

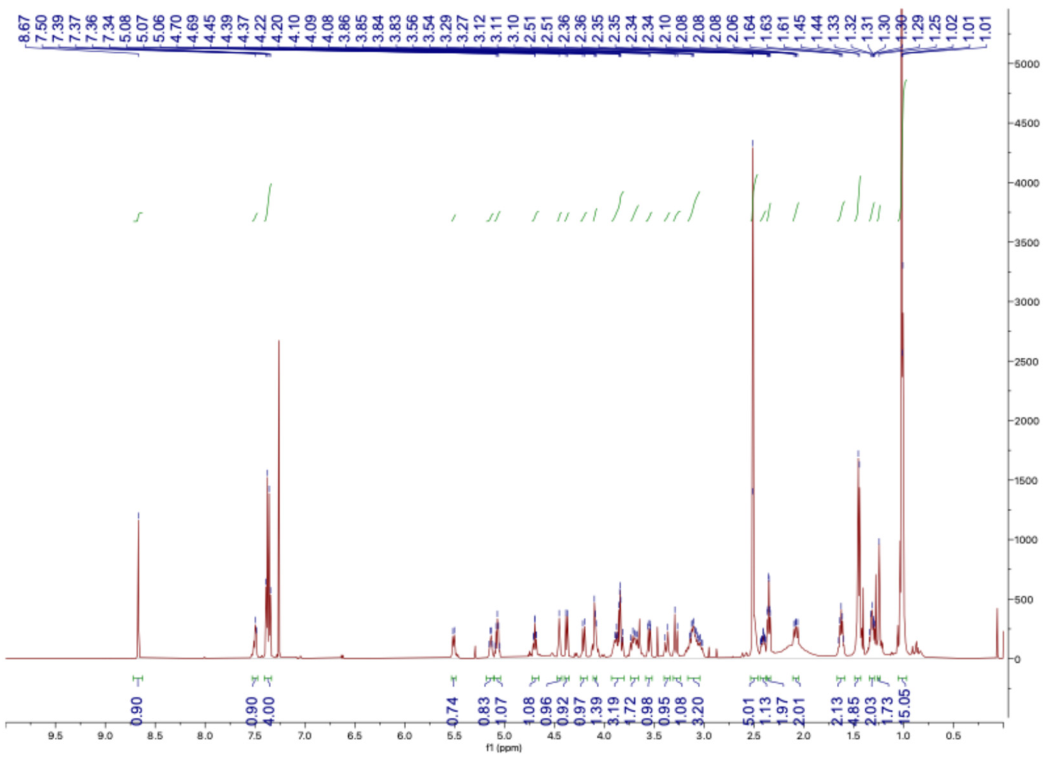
8.67
7.50
7.39
7.37
7.36
7.34
5.08
5.07
5.06
4.70
4.69
4.45
4.39
4.37
4.22
4.20
4.10
4.09
4.08
3.86
3.85
3.84
3.83
3.56
3.54
3.29
3.27
3.12
3.11
3.10
2.51
2.51
2.36
2.36
2.35
2.34
2.34
2.10
2.08
2.08
2.08
2.06
1.64
1.63
1.61
1.45
1.44
1.33
1.32
1.31
1.30
1.30
1.29
1.25
1.02
1.01
1.01
0.90
0.90
4.00
0.74
0.83
1.07
1.08
0.96
0.92
0.97
1.39
3.19
1.72
0.98
0.95
1.08
3.20
5.01
1.13
1.97
2.01
2.13
4.85
2.03
1.73
15.05
f1 (ppm)

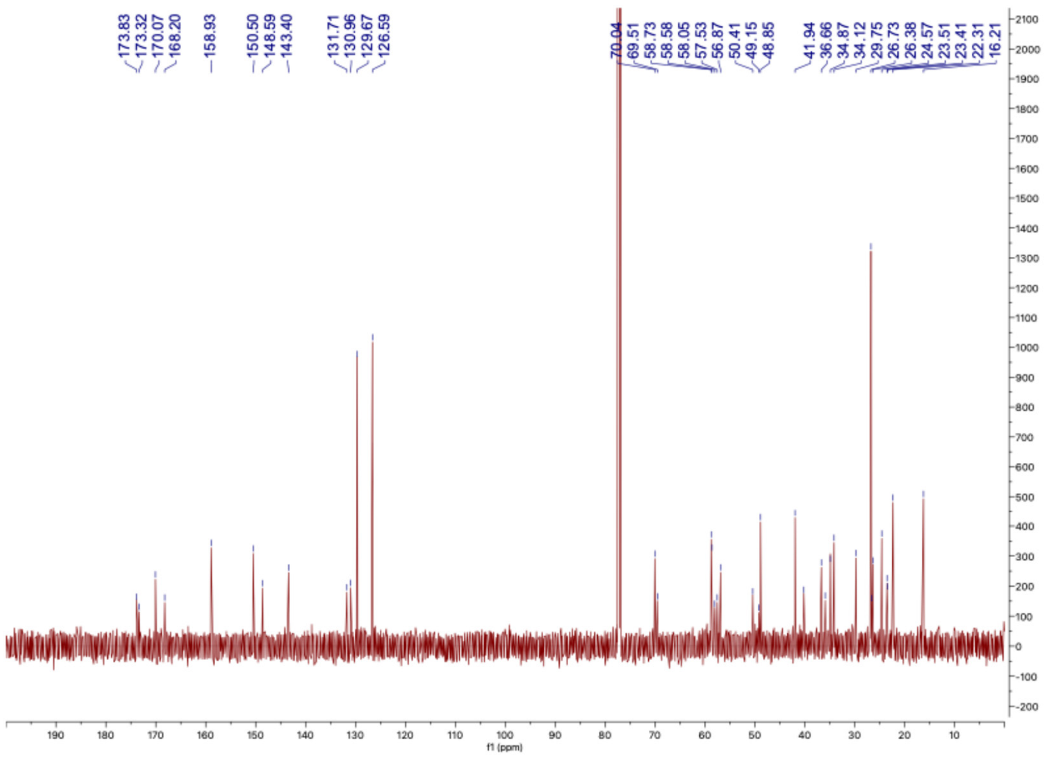
173.83
173.32
170.07
168.20
158.93
150.50
148.59
143.40
131.71
130.96
129.67
126.59
70.04
69.51
58.73
58.58
58.05
57.53
56.87
50.41
49.15
48.85
41.94
36.66
34.87
34.12
29.75
26.73
26.38
24.57
23.51
23.41
22.31
16.21
f1 (ppm)

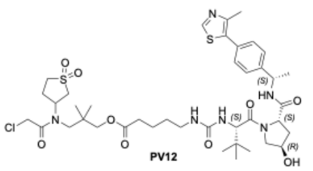

N
S
O
S=O
Cl
N
O
O
O
H
N
N
H
(S)
N
O
(S)
HN
O
(S)
(R)
OH

PV12

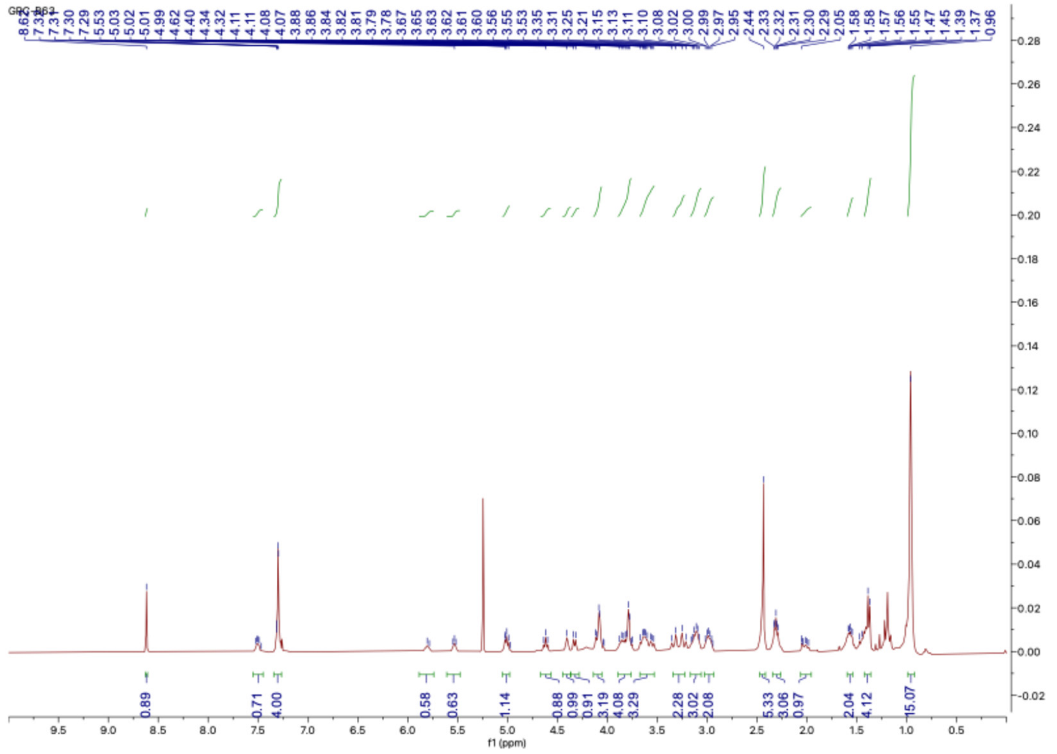

8.62
7.31
7.30
7.29
5.53
5.03
5.02
5.01
4.99
4.62
4.40
4.34
4.32
4.11
4.11
4.08
4.07
3.88
3.86
3.84
3.82
3.81
3.79
3.78
3.67
3.65
3.63
3.62
3.61
3.60
3.56
3.55
3.53
3.35
3.31
3.25
3.21
3.15
3.13
3.11
3.10
3.08
3.02
3.00
2.99
2.97
2.95
2.44
2.33
2.32
2.31
2.30
2.29
2.05
1.58
1.58
1.57
1.56
1.55
1.47
1.45
1.39
1.37
0.96
0.89
0.71
4.00
0.58
0.63
1.14
0.88
0.99
0.91
3.19
4.08
3.29
2.28
3.02
2.08
5.33
3.06
0.97
2.04
4.12
15.07
f1 (ppm)

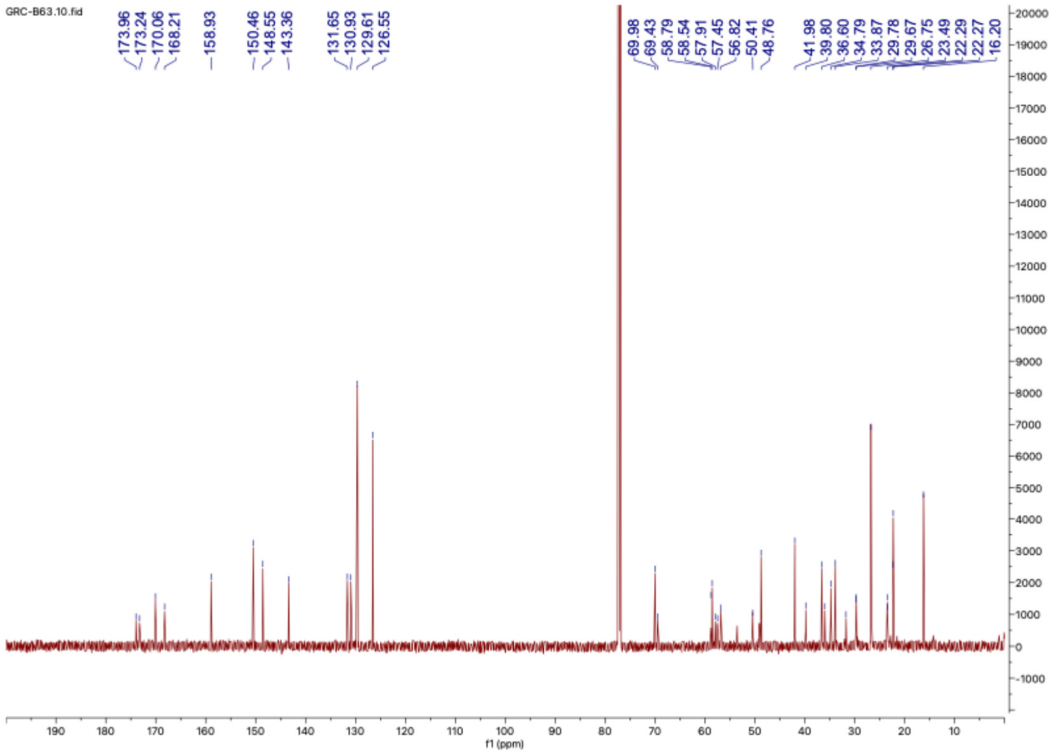

GRC-B63.10.fid
173.96
173.24
170.06
168.21
158.93
150.46
148.55
143.36
131.65
130.93
129.61
126.55
69.98
69.43
58.79
58.54
57.91
57.45
56.82
50.41
48.76
41.98
39.80
36.60
34.79
33.87
29.78
29.67
26.75
23.49
22.29
22.27
16.20
f1 (ppm)

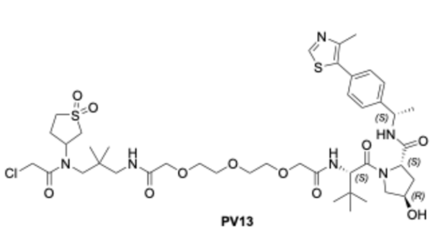

PV13

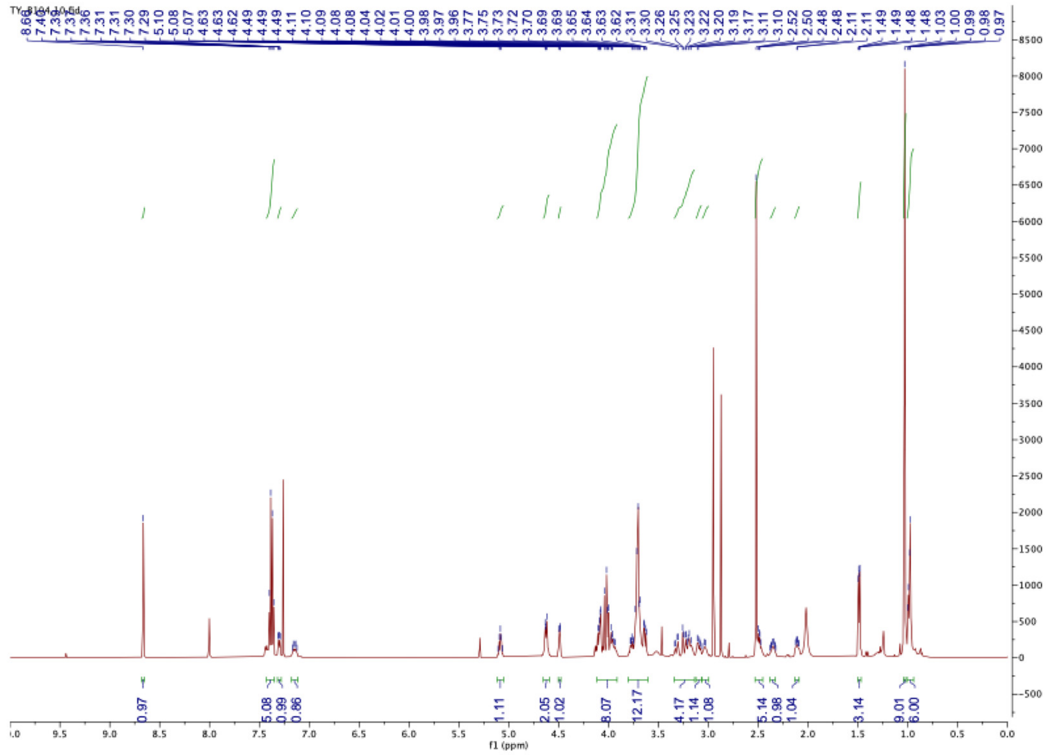

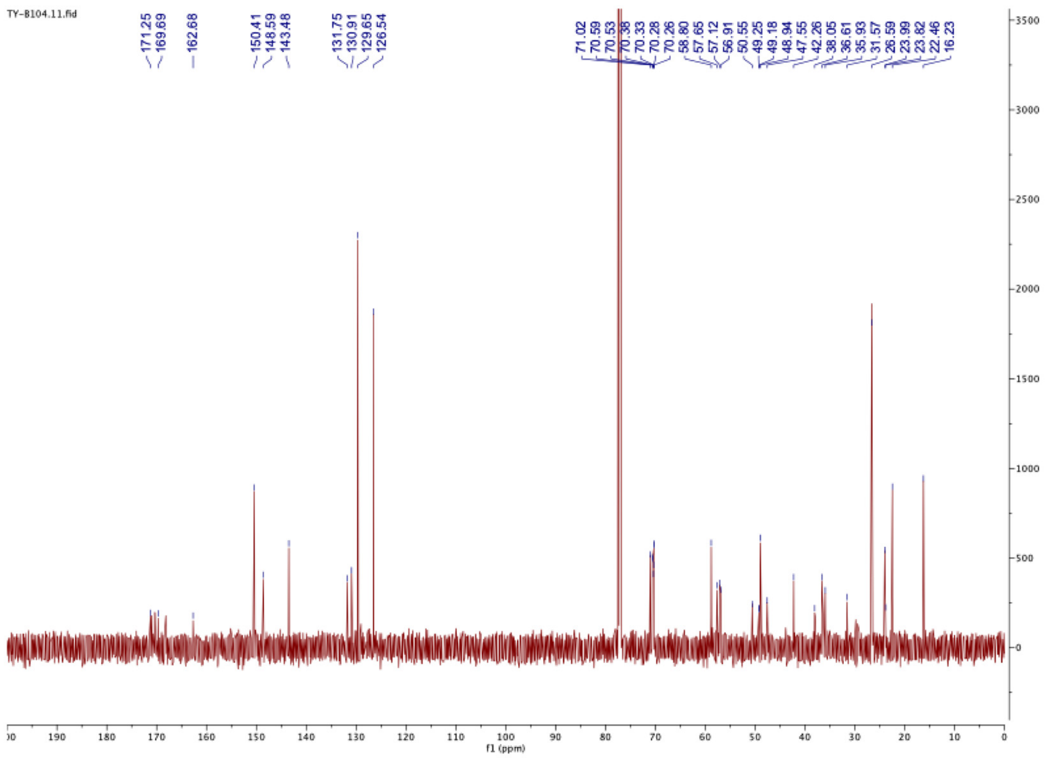

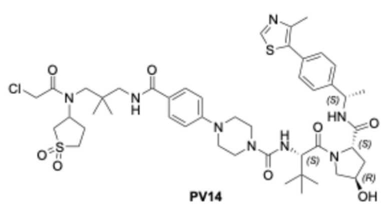

PV14

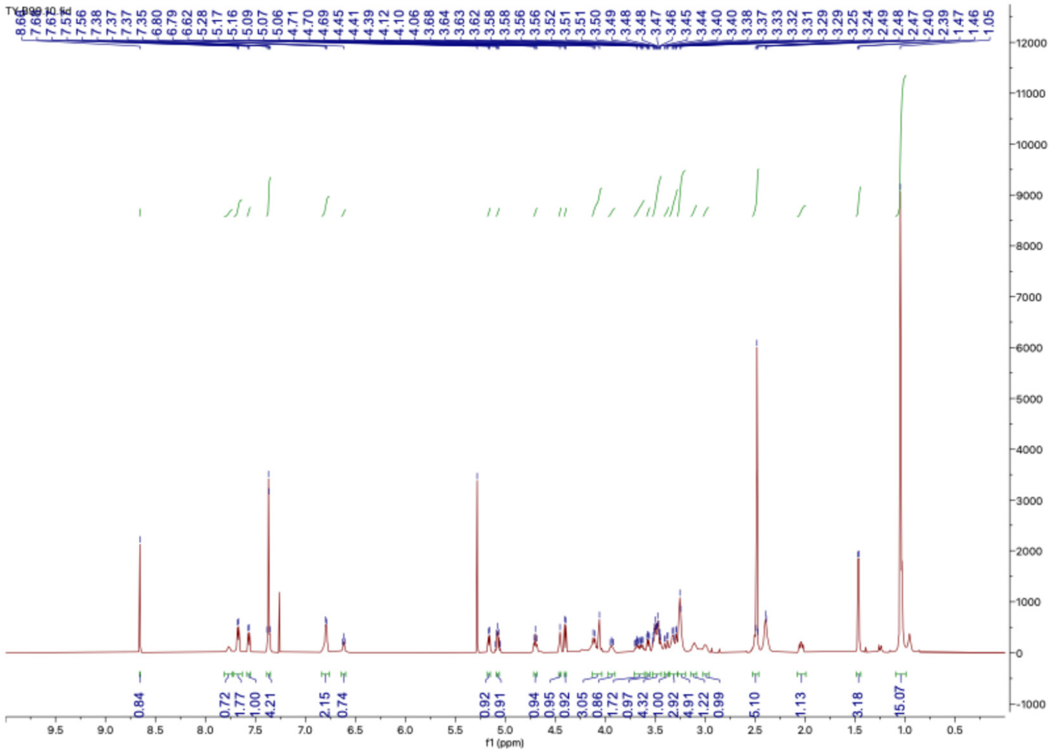

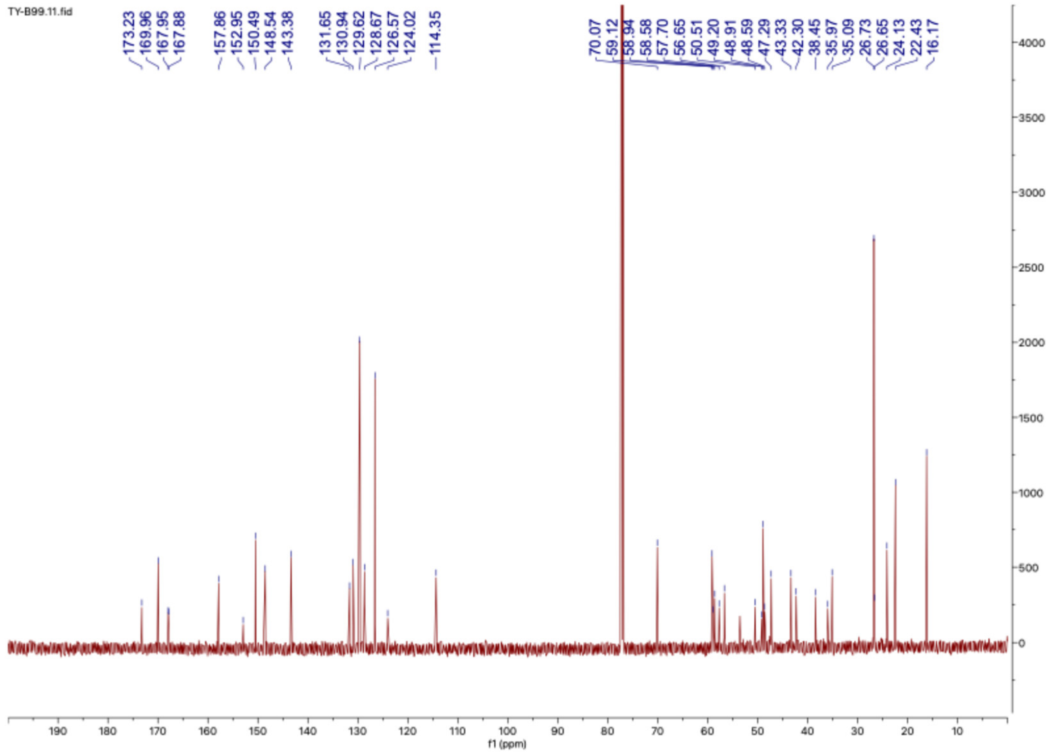

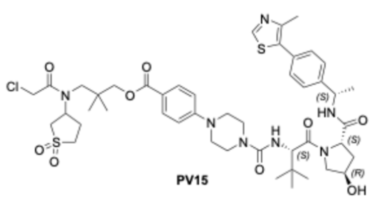
PV15

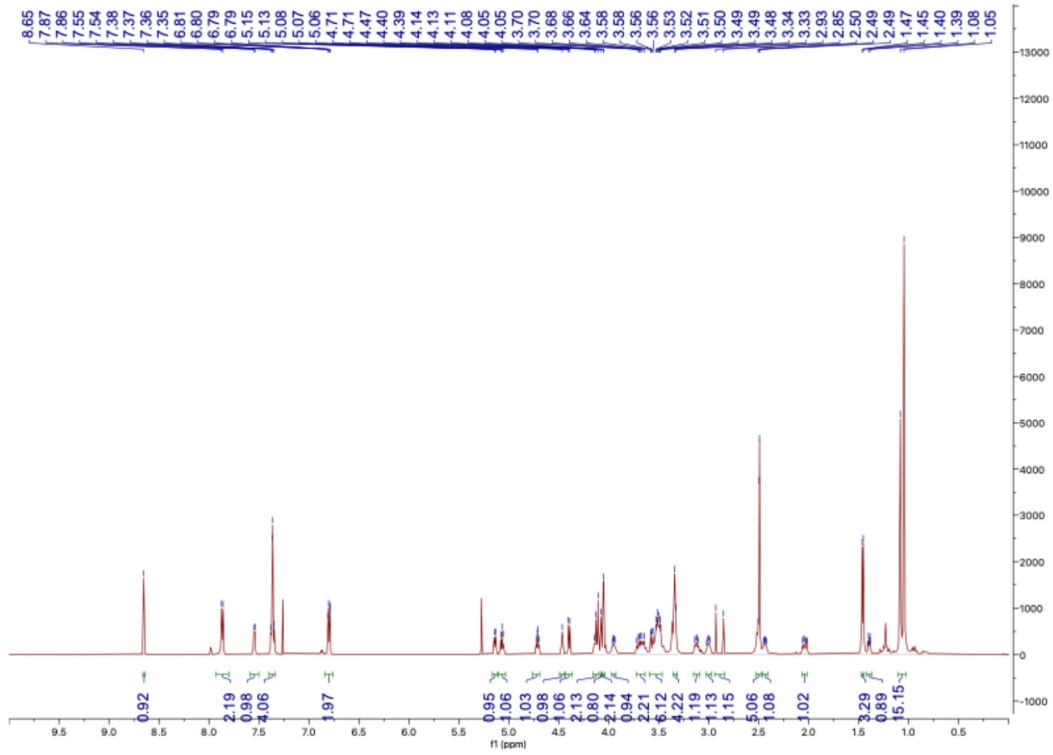
8.65 7.87 7.86 7.55 7.54 7.38 7.37 7.36 7.35 6.81 6.80 6.79 6.79 5.15 5.13 5.08 5.07 5.06 4.71 4.71 4.47 4.40 4.39 4.14 4.13 4.11 4.08 4.05 4.05 3.70 3.70 3.68 3.66 3.64 3.58 3.58 3.56 3.56 3.53 3.52 3.51 3.50 3.49 3.49 3.48 3.34 3.33 2.93 2.85 2.50 2.49 2.49 1.47 1.45 1.40 1.39 1.08 1.05
0.92 2.19 0.98 4.06 1.97 0.95 1.06 1.03 0.98 1.06 2.13 0.80 2.14 0.94 2.21 6.12 4.22 1.19 1.13 1.15 5.06 1.08 1.02 3.29 0.89 15.15
f1 (ppm)

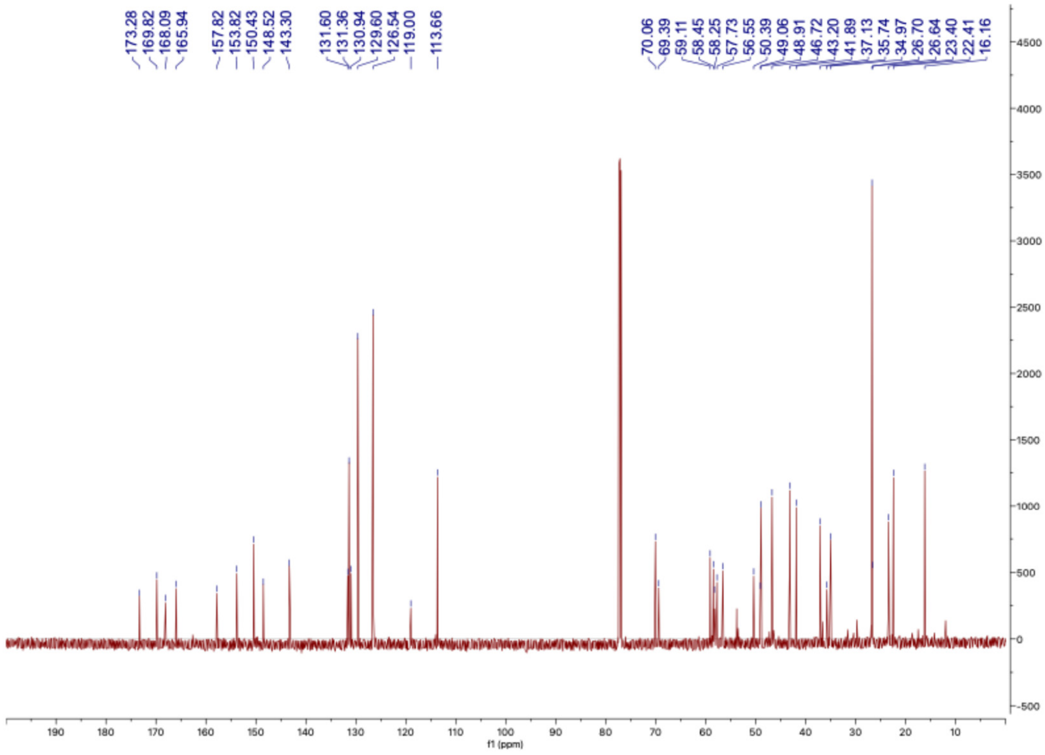
173.28 169.82 168.09 165.94 157.82 153.82 150.43 148.52 143.30 131.60 131.36 130.94 129.60 126.54 119.00 113.66
70.06 69.39 59.11 58.45 58.25 57.73 56.55 50.39 49.06 48.91 46.72 43.20 41.89 37.13 35.74 34.97 26.70 26.64 23.40 22.41 16.16
f1 (ppm)

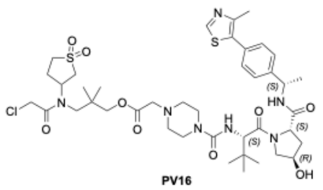

PV16

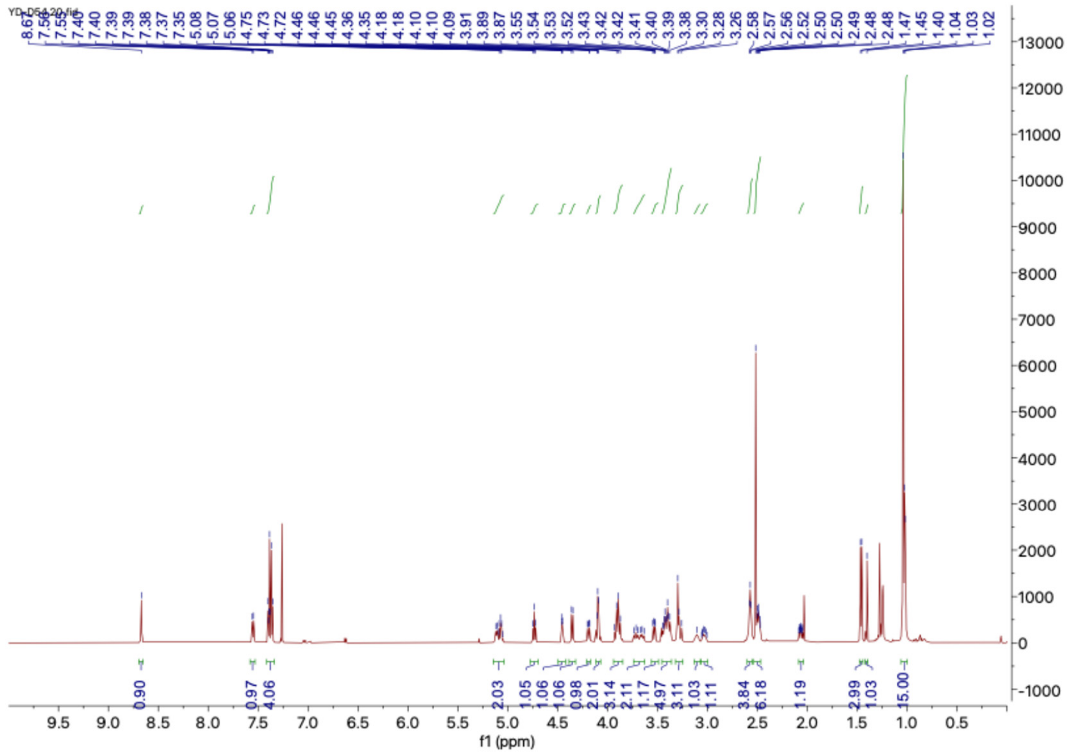
7.56
7.55
7.40
7.39
7.39
7.38
7.37
7.35
5.08
5.07
5.06
4.75
4.73
4.72
4.46
4.46
4.45
4.36
4.35
4.18
4.18
4.10
4.09
3.91
3.89
3.87
3.55
3.54
3.53
3.52
3.43
3.42
3.42
3.41
3.40
3.39
3.38
3.30
3.28
3.26
2.58
2.57
2.56
2.52
2.50
2.50
2.49
2.48
2.48
1.47
1.45
1.40
1.04
1.03
1.02
0.90
0.97
4.06
2.03
1.05
1.06
0.98
2.01
3.14
2.11
1.17
4.97
3.11
1.03
1.11
3.84
6.18
1.19
2.99
1.03
15.00
f1 (ppm)

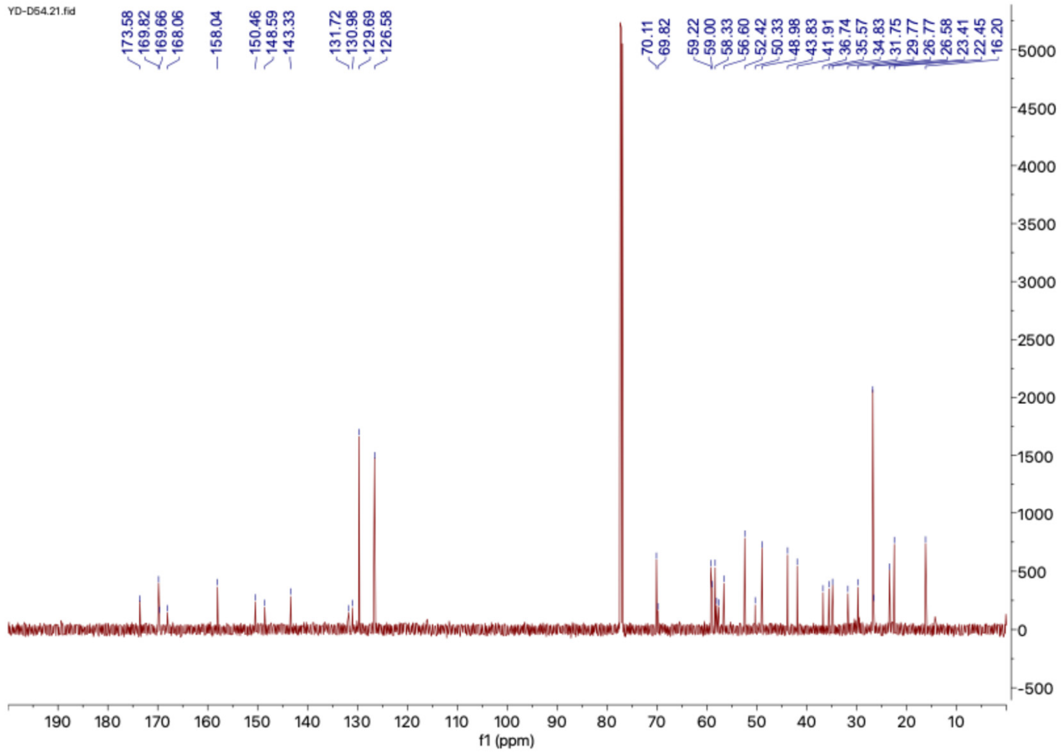
YD-D54.21.fid
173.58
169.82
169.66
168.06
158.04
150.46
148.59
143.33
131.72
130.98
129.69
126.58
70.11
69.82
59.22
59.00
58.33
56.60
52.42
50.33
48.98
43.83
41.91
36.74
35.57
34.83
31.75
29.77
26.58
23.41
22.45
16.20
f1 (ppm)

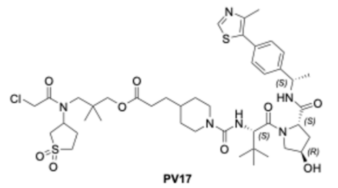
Cl
N
O
S
N
HN
(S)
(R)
OH

PV17

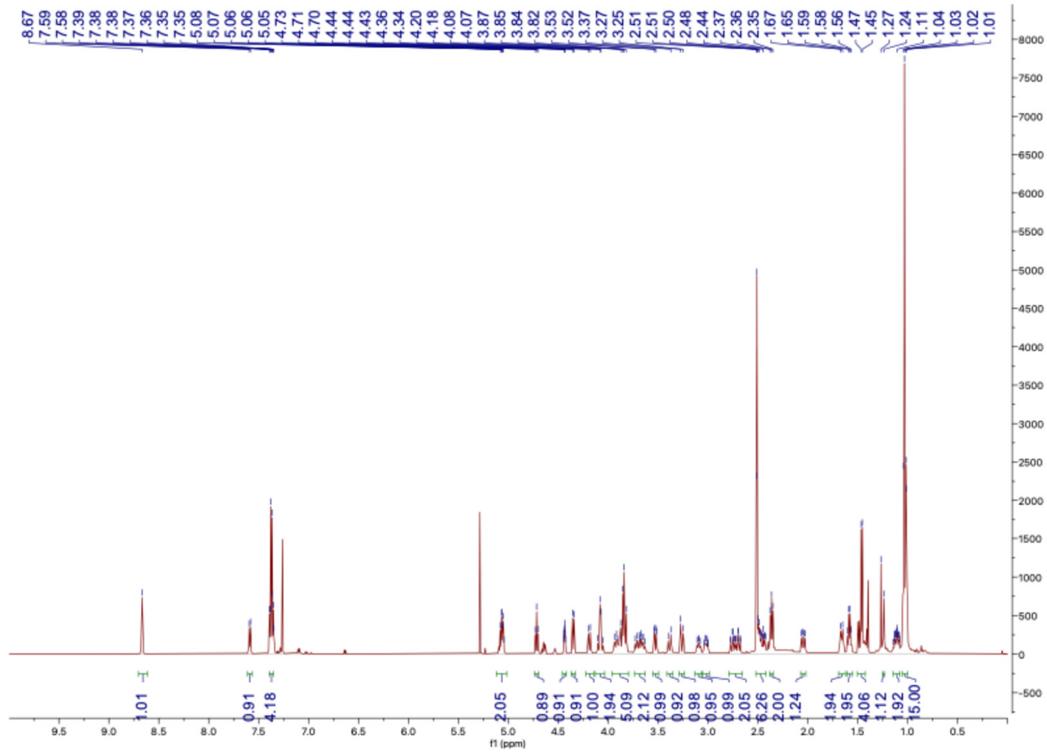
8.67 7.59 7.58 7.39 7.38 7.38 7.37 7.36 7.35 7.35 5.08 5.07 5.06 5.06 5.05 4.73 4.71 4.70 4.44 4.44 4.43 4.36 4.34 4.20 4.18 4.08 4.07 3.87 3.85 3.84 3.82 3.53 3.52 3.37 3.27 3.25 2.51 2.51 2.50 2.48 2.44 2.37 2.36 2.35 1.67 1.65 1.59 1.58 1.56 1.47 1.45 1.27 1.24 1.11 1.04 1.03 1.02 1.01
1.01 0.91 4.18 2.05 0.89 0.91 0.91 1.00 1.94 5.09 2.12 0.99 0.99 0.92 0.98 0.99 0.95 2.05 6.26 2.00 1.24 1.94 1.95 4.06 1.12 1.92 15.00
f1 (ppm)

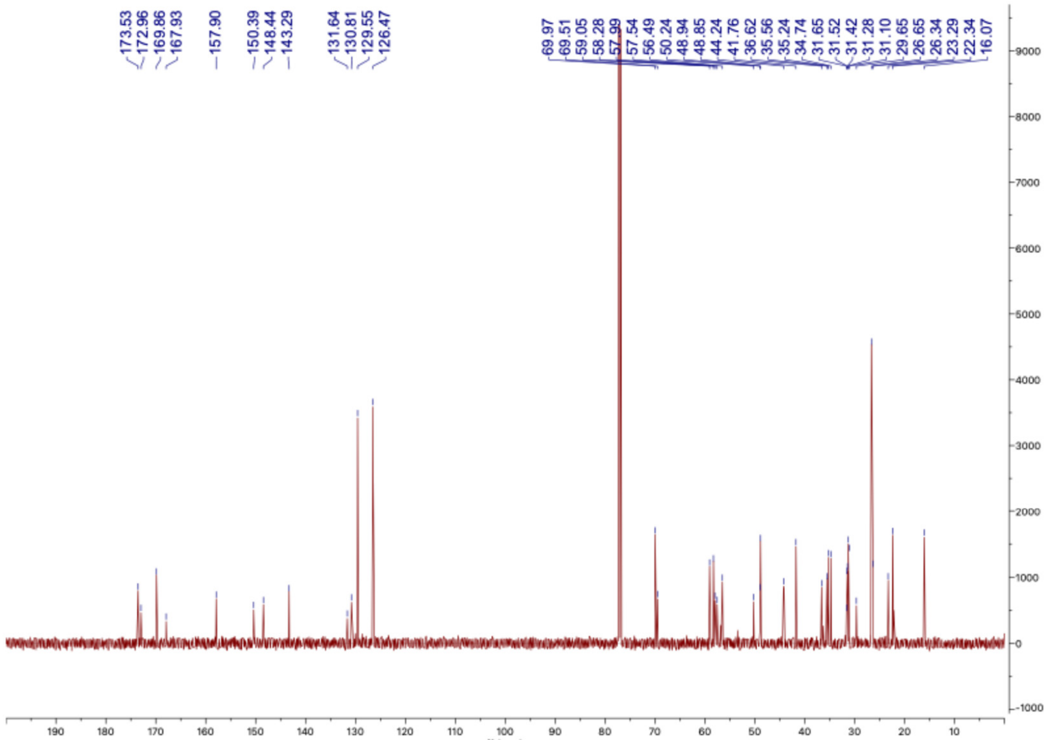
173.53 172.96 169.86 167.93 157.90 150.39 148.44 143.29 131.64 130.81 129.55 126.47
69.97 69.51 59.05 58.28 57.99 57.54 56.49 50.24 48.94 48.85 44.24 41.76 36.62 35.56 35.24 34.74 31.65 31.52 31.42 31.28 31.10 29.65 26.65 26.34 23.29 22.34 16.07
f1 (ppm)

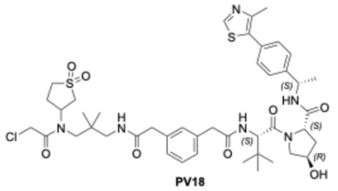

PV18

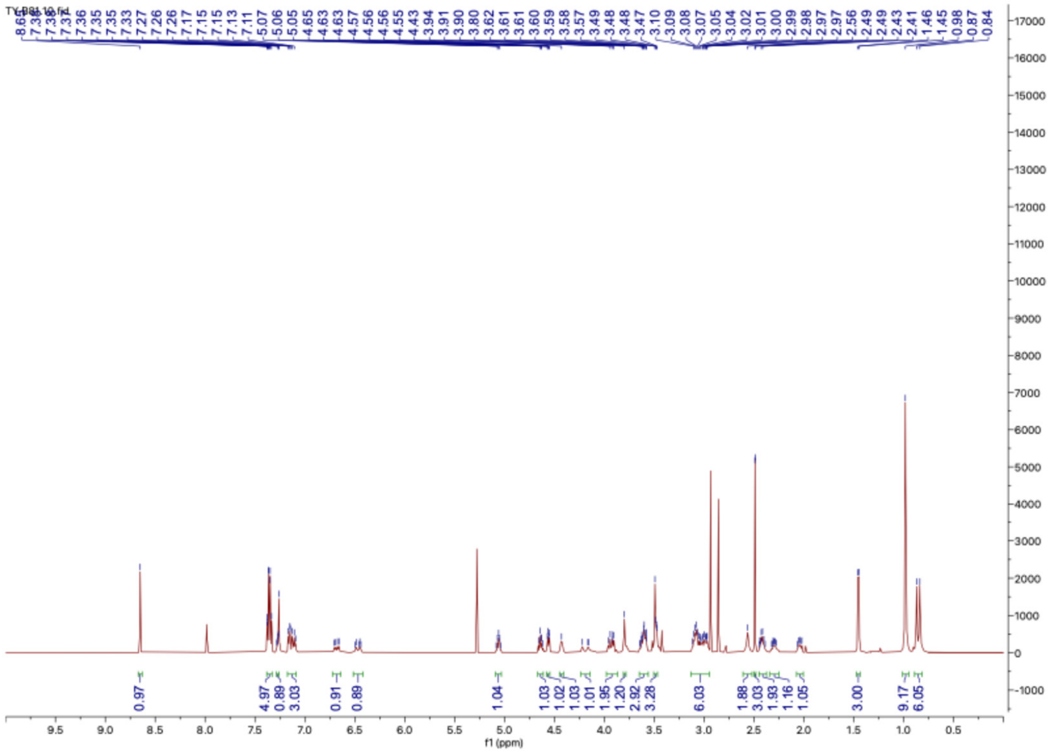
f1 (ppm)

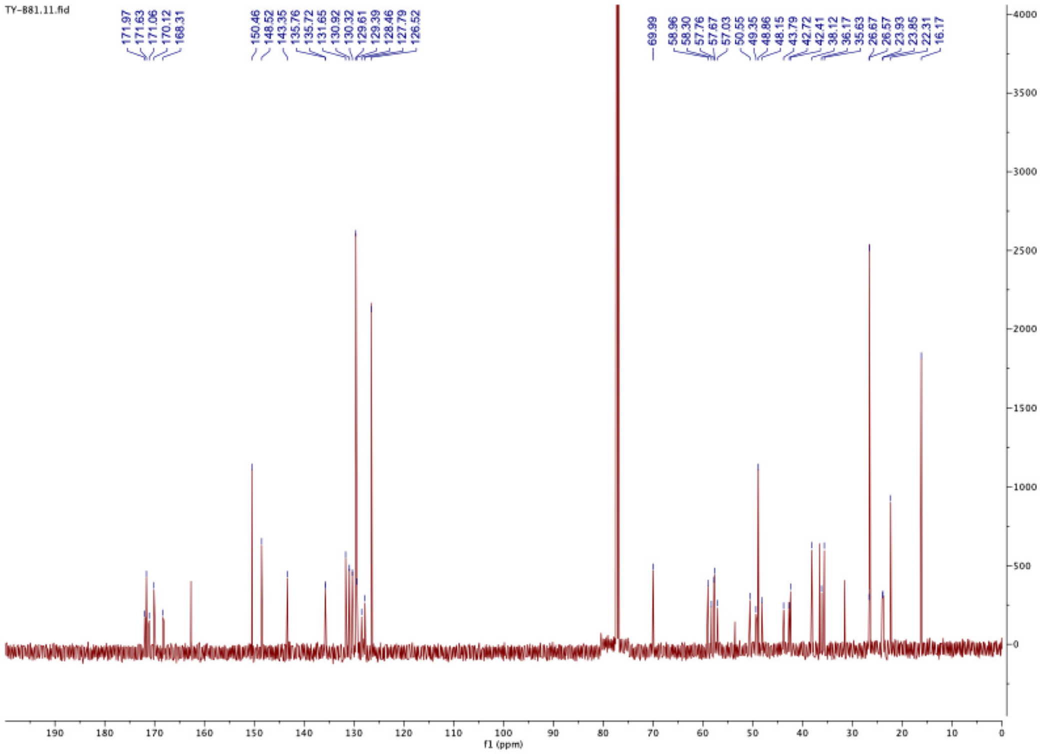
TY-B81.11.fid
171.97
171.63
171.06
170.12
168.31
150.46
148.52
143.35
135.76
135.72
131.65
130.92
130.32
129.61
129.39
128.46
127.79
126.52
69.99
58.96
58.30
57.76
57.67
57.03
50.55
49.35
48.86
48.15
43.79
42.72
42.41
38.12
36.17
35.63
26.67
26.57
23.93
23.85
22.31
16.17
f1 (ppm)

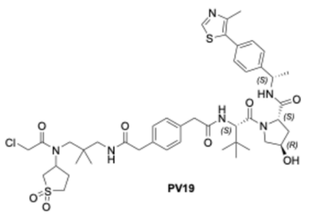

PV19

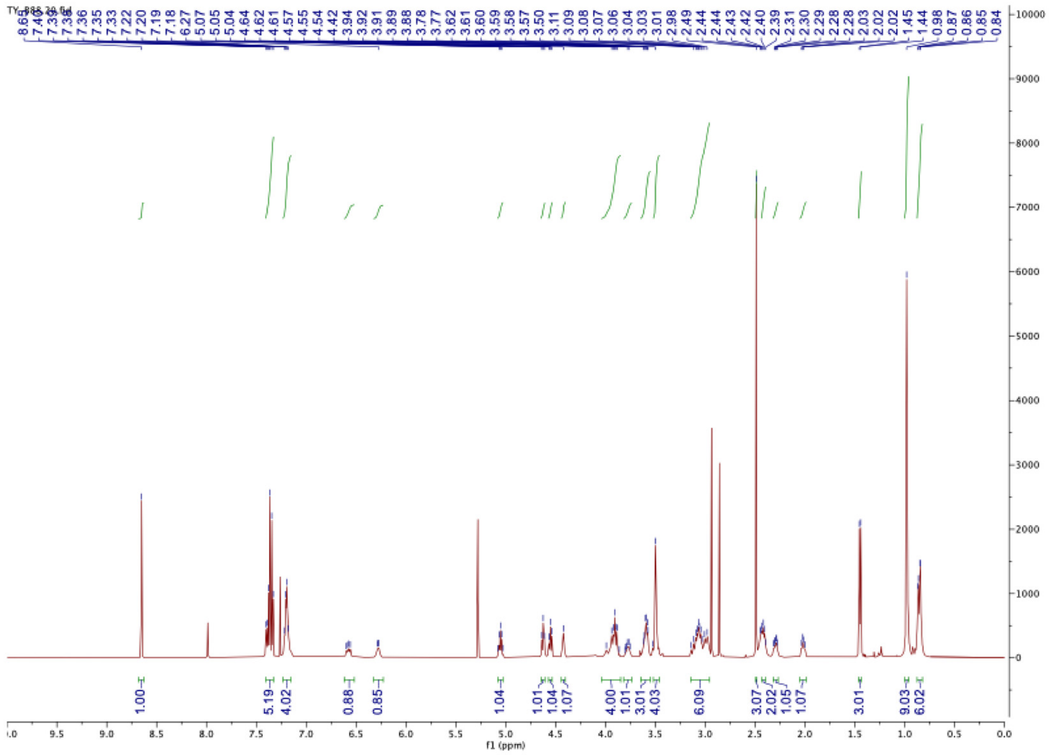

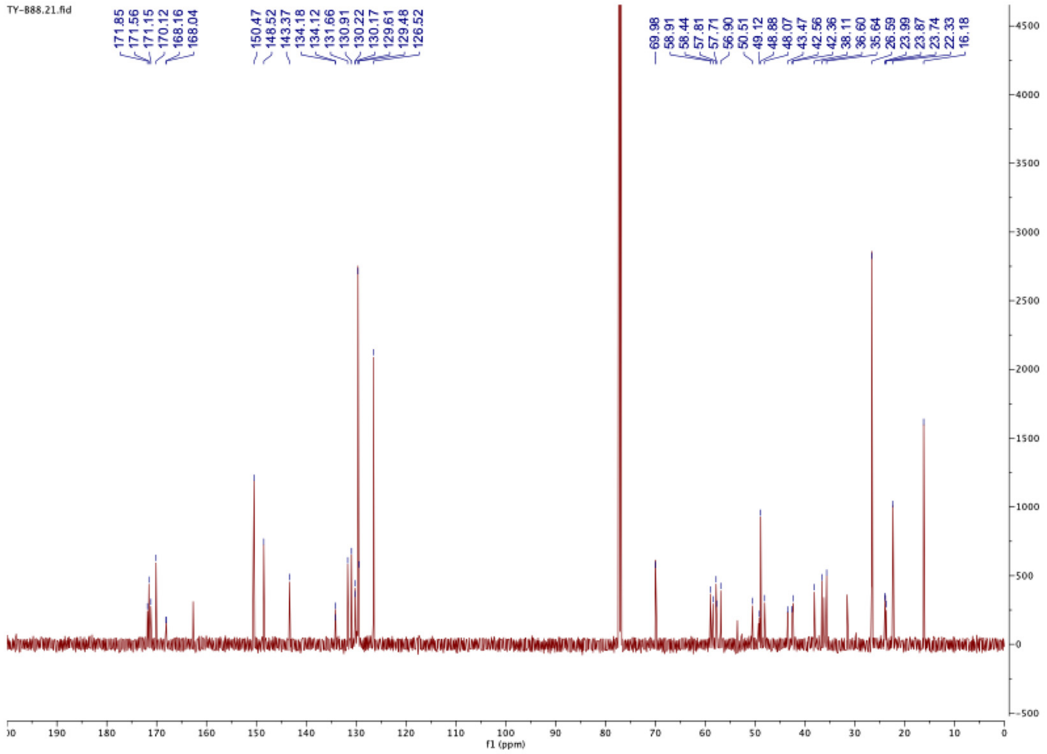

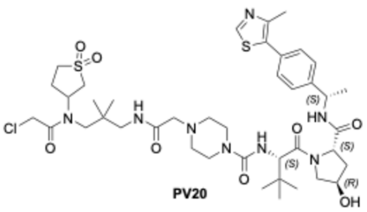

PV20

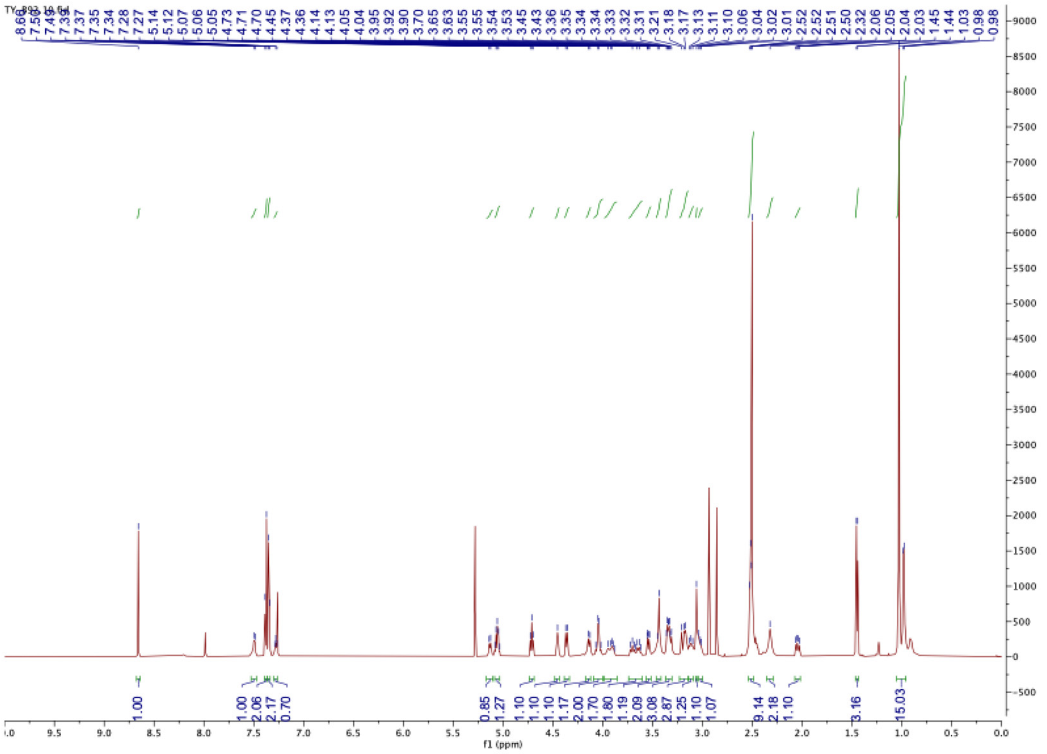
8.66
7.50
7.49
7.38
7.37
7.35
7.34
7.28
7.27
5.14
5.12
5.07
5.06
5.05
4.73
4.71
4.70
4.45
4.37
4.36
4.14
4.13
4.05
4.04
3.95
3.92
3.90
3.70
3.65
3.63
3.55
3.55
3.54
3.53
3.45
3.43
3.36
3.35
3.34
3.34
3.33
3.32
3.31
3.21
3.18
3.17
3.13
3.11
3.10
3.06
3.04
3.02
3.01
2.52
2.52
2.51
2.50
2.32
2.06
2.05
2.04
2.03
1.45
1.44
1.03
0.98
0.98
f1 (ppm)

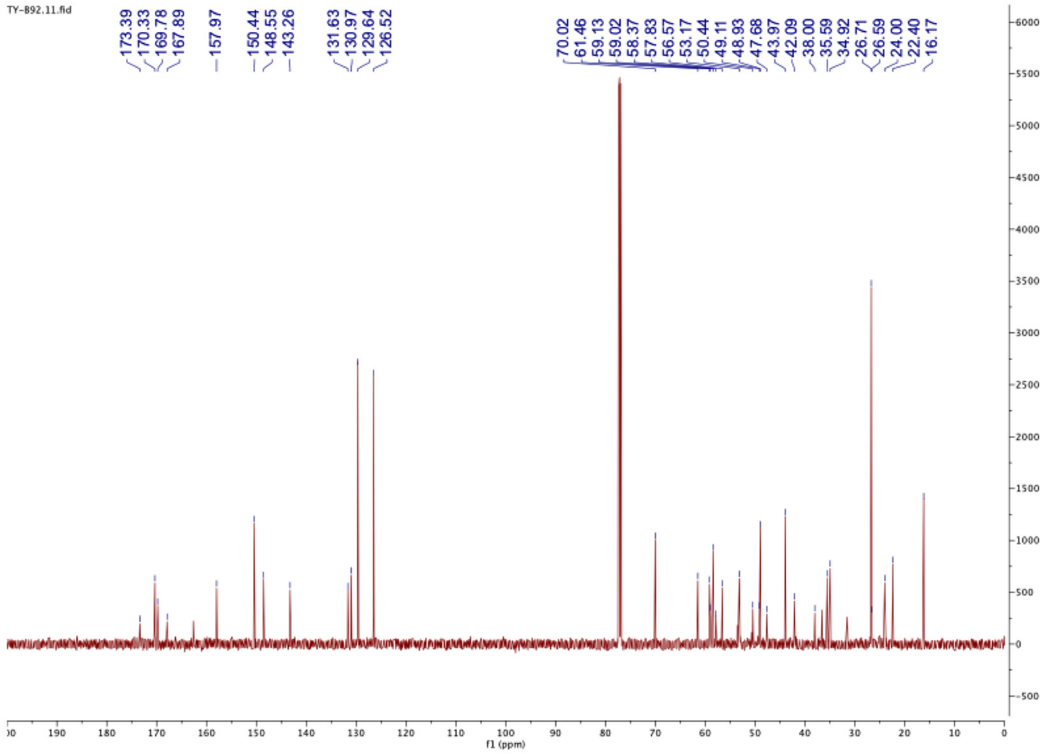
TY-B92.11.fid
173.39
170.33
169.78
167.89
157.97
150.44
148.55
143.26
131.63
130.97
129.64
126.52
70.02
61.46
59.13
59.02
58.37
57.83
56.57
53.17
50.44
49.11
48.93
47.68
43.97
42.09
38.00
35.59
34.92
26.71
26.59
24.00
22.40
16.17
f1 (ppm)

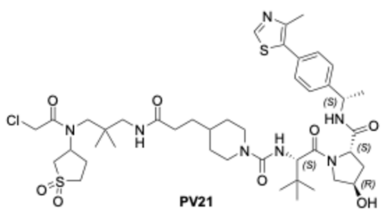

PV21

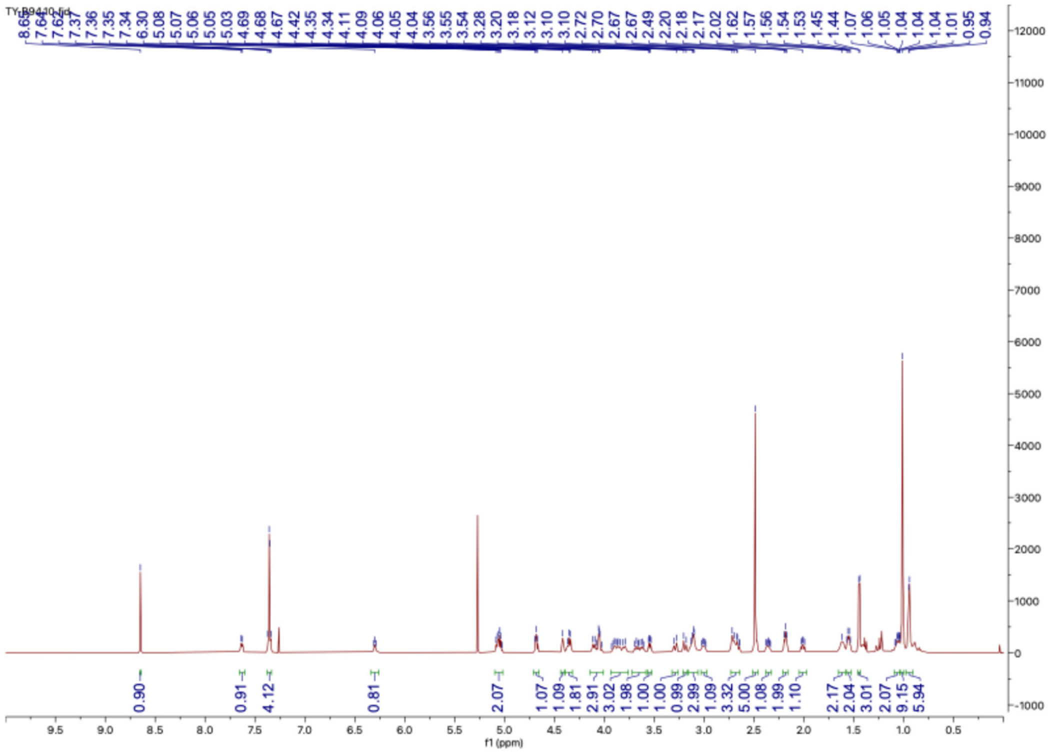

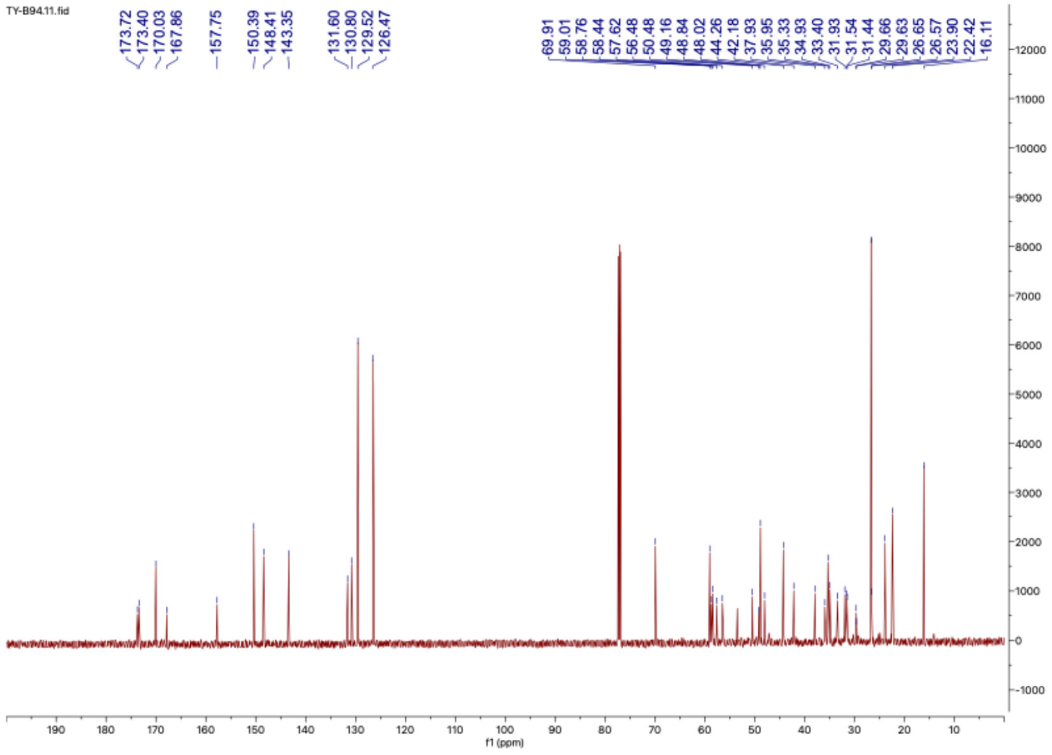

Supplement: Supplementary file 1 [file pharmaceutics-18-00288-s001.zip › pharmaceutics-Supplementary File S1-1H and 13C NMR spectra.pdf]
