# Supplementary material for: Systematic Optimization of Proteolysis-Targeting Chimeras for PIN1 Enables Selective Degradation and Antitumor Activity In Vivo
Source: Pharmaceutics. 2026 Feb 26;18(3):288. doi: 10.3390/pharmaceutics18030288 (PMC13029591; doi:10.3390/pharmaceutics18030288)
Supplement: Supplementary file 1 [file pharmaceutics-18-00288-s001.zip › pharmaceutics-Supplementary File S2-Synthetic procedures for intermediates and PC2-Neg..pdf]

pharmaceutics

MDPI

Supplementary Materials: Systematic Optimization of Proteolysis-Targeting Chimeras for PIN1 Enables Selective Degradation and Antitumor Activity In Vivo

Yuying Ma, Yang Teng, Jinjin Liu, Yuke Deng, Lingbo Xu, Ruichen Gao, Tingyu Peng, Wei Li, Yue Wei, Linfeng Li, and Zufeng Guo

Synthetic procedures for intermediates and PC2-Neg.

Scheme S1. Synthesis of Key Intermediate I-1 and I-2.

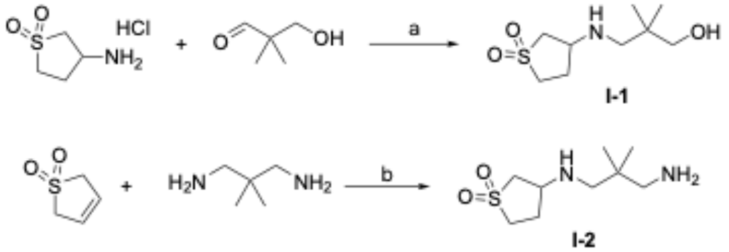

Reagents and conditions: (a) TEA (0.9 equiv), acetic acid (0.2 equiv), STAB (2.1 equiv), DMF, RT, overnight; (b) Cs_2CO_3 (10 mol%), 100 °C, 8 h.

3-((3-hydroxy-2,2-dimethylpropyl)amino)tetrahydrothiophene 1,1-dioxide (**I-1**). 3-Aminotetrahydrothiophene 1,1-dioxide hydrochloride (3.0 g, 17.4 mmol, 1.0 equiv) and triethylamine (TEA) (2.19 mL, 15.7 mmol, 0.9 equiv) were dissolved in anhydrous DMF (15 mL) and stirred at room temperature (RT) for 1 h. To the mixture, 3-hydroxy-2,2-dimethylpropanal (1.1 equiv) and acetic acid (0.2 equiv) were added, and the reaction was stirred for an additional hour at RT. Sodium triacetoxyborohydride (STAB) (7.74 g, 36.54 mmol, 2.1 equiv) was then added in one portion, and the mixture was stirred overnight at RT. After solvent evaporation, the residue was dissolved in saturated aqueous $NaHCO_3$, and the aqueous phase was extracted with DCM (2×). The organic layers were combined, dried over Na_2SO_4 and concentrated. The residue was purified by column chromatography (DCM: MeOH= 30: 1, v/v) to afford the pure

compound **I-1** as a white solid (Yield: 78%). ^{1}H NMR (600 MHz, Methanol-*d*$_4$) δ 3.59 (qd, *J* = 7.2, 5.5 Hz, 1H), 3.42 – 3.34 (m, 3H), 3.30 – 3.25 (m, 1H), 3.08 (dddd, *J* = 13.2, 8.5, 7.6, 0.8 Hz, 1H), 2.98 (dd, *J* = 13.3, 6.9 Hz, 1H), 2.57 (q, *J* = 11.7 Hz, 2H), 2.49 – 2.43 (m, 1H), 2.12 (dq, *J* = 13.5, 8.1 Hz, 1H), 0.91 (d, *J* = 8.3 Hz, 6H). ^{13}C NMR (151 MHz, Methanol- *d*$_4$) δ 71.27, 56.91, 56.87, 56.84, 51.61, 36.44, 29.60, 23.13, 23.04. LC-MS (m/z): positive mode 222.1 $[M + H]^+$.

3-((3-amino-2,2-dimethylpropyl)amino)tetrahydrothiophene 1,1-dioxide (**I-2**). To a dry Schlenk tube containing a magnetic stir bar, 2,5-dihydrothiophene 1,1-dioxide (5.0 g, 42.3 mmol, 1.0 equiv), 2,2-dimethylpropane-1,3-diamine (8.6 g, 84.6 mmol, 2.0 equiv), and Cs_2CO_3 (10 mol%) were added sequentially. The tube was tightly sealed, and the reaction mixture was vigorously stirred in a pre-warmed 100 °C oil bath for 8 hours. After the reaction was completed, remove the excess amine by rotary evaporation, then the residue was purified by column chromatography (DCM: MeOH = 15: 1, v/v) to afford the pure compound **I-2** as a colorless oil (Yield: 68%). ^{1}H NMR (600 MHz, Chloroform-*d*) δ 3.55 (ddd, *J* = 11.9, 6.5, 5.3 Hz, 1H), 3.35 – 3.29 (m, 1H), 3.29 – 3.24 (m, 1H), 3.10 – 3.01 (m, 1H), 2.96 (ddt, *J* = 13.3, 5.7, 1.0 Hz, 1H), 2.62 – 2.53 (m, 2H), 2.45 (d, *J* = 1.9 Hz, 2H), 2.43 – 2.37 (m, 1H), 2.12 (dddd, *J* = 14.7, 13.7, 7.2, 1.0 Hz, 1H), 0.89 (d, *J* = 4.9 Hz, 6H). LC-MS (m/z): positive mode 221.1 $[M + H]^+$.

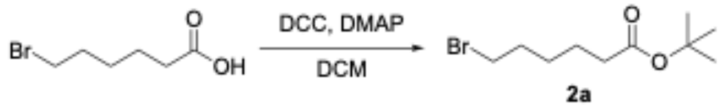

Tert-butyl 6-bromohexanoate (**2a**). To a solution of 6-bromohexanoic acid (3.0 g, 15.5 mmol, 1.0 equiv) in anhydrous DCM, tert-butanol (7.5 mL, 77.7 mmol, 5.0 equiv),

DCC (4.1 g, 20.1 mmol, 1.3 equiv), and DMAP (195 mg, 1.6 mmol, 0.1 equiv) were added at 0° C. The reaction mixture was allowed to warm to RT and stirred for 60 hours. After completion, the mixture was washed sequentially with 1.0 M HCl, aqueous $NaHCO_3$, and brine, then dried over Na_2SO_4. The organic layer was concentrated under reduced pressure, and the residue was purified by column chromatography (PE: EA = 40: 1, v/v), affording the pure compound **2a** as a white solid (Yield: 82%). ^{1}H NMR (600 MHz, Chloroform-*d*) δ 3.74 (t, *J* = 5.6 Hz, 2H), 3.67 (t, *J* = 6.2 Hz, 2H), 3.63 (t, *J* = 5.7 Hz, 2H), 2.48 (t, *J* = 6.2 Hz, 2H), 1.82 – 1.79 (m, 2H), 1.44 (s, 9H). LC-MS (m/z): positive mode 251.1 $[M + H]^+$.

Tert-butyl 6-((2-(2,6-dioxopiperidin-3-yl)-1,3-dioxoisoindolin-4-yl)oxy)hexanoate (**3a**). To a solution of thalidomide-4-OH (1.0 g, 3.6 mmol, 1.0 equiv) in anhydrous DMF was added **2a** (1.1 g, 4.3 mmol, 1.2 equiv) and DIPEA (1.9 mL, 10.8 mmol, 3.0 equiv). The reaction mixture was stirred at 70 °C for 20 h. After completion of the reaction (monitored by TLC), the mixture was concentrated in vacuo. The residue was extracted with DCM, washed with H_2O, brine and dried over Na_2SO_4. The DCM layer was concentrated, then the residue was purified by column chromatography (DCM: MeOH = 15: 1, v/v) to afford the pure compound **3a** as a white solid (Yield: 48%). ^{1}H NMR (600 MHz, DMSO-*d*$_6$) δ 11.11 (s, 1H), 7.80 (dd, *J* = 8.5, 7.3 Hz, 1H), 7.51 (d, *J* = 8.5 Hz, 1H), 7.44 (d, *J* = 7.2 Hz, 1H), 5.07 (dd, *J* = 12.9, 5.4 Hz, 1H), 4.19 (t, *J* = 6.4 Hz, 2H), 2.88 – 2.83 (m, 1H), 2.58 (dt, *J* = 17.9, 3.3 Hz, 1H), 2.52 (dd, *J* = 3.9, 2.0 Hz, 1H), 2.21 (t, *J* = 7.3 Hz, 2H), 2.07 – 1.96 (m, 1H), 1.76 (p, *J* = 6.7 Hz, 2H), 1.56 (p, *J* = 7.4 Hz, 2H), 1.45 (tt, *J* = 9.0, 5.9 Hz, 2H), 1.38 (s, 9H). LC-MS (m/z): positive mode 445.2 $[M + H]^+$.

6-((2-(2,6-dioxopiperidin-3-yl)-1,3-dioxoisoindolin-4-yl)oxy)hexanoic acid (**4a**). To a solution of **3a** (500 mg, 1.1 mmol, 1.0 equiv) in anhydrous DCM (5 mL) at 0 °C. TFA (20% v/v, 1.0 mL) was added at 0 °C. Then it was allowed to warmed to RT and stirred for 4 h. After the reaction was completed, removed the solvent in vacuo. The obtained residue was directly used in the next step without further purification.

Tert-butyl 4-((2-(2,6-dioxopiperidin-3-yl)-1,3-dioxoisoindolin-4-yl)oxy)butanoate (**3b**). Compound **3b** (Yield: 81%, yellow solid) was synthesized following the same procedure as for **3a**, using commercially available *tert*-butyl 4-bromobutanoate (**2b**). ^{1}H NMR (600 MHz, Chloroform-*d*) δ 8.49 (s, 1H), 7.65 (dd, *J* = 8.5, 7.3 Hz, 1H), 7.43 (d, *J* = 7.3 Hz, 1H), 7.22 (d, *J* = 8.5 Hz, 1H), 4.95 (dd, *J* = 12.2, 5.4 Hz, 1H), 4.21 (t, *J* = 6.3 Hz, 2H), 2.89 – 2.67 (m, 3H), 2.48 (t, *J* = 7.2 Hz, 2H), 2.17 – 2.06 (m, 3H), 1.42 (s, 9H). ^{13}C NMR (151 MHz, Chloroform-*d*) δ 172.49, 171.36, 168.40, 167.16, 165.76, 156.55, 136.63, 133.88, 119.16, 117.29, 116.00, 80.67, 68.39, 49.19, 31.53, 31.46, 28.19, 24.45, 22.70. LC-MS (m/z): positive mode 417.2 $[M + H]^{+}$.

4-((2-(2,6-dioxopiperidin-3-yl)-1,3-dioxoisoindolin-4-yl)oxy)butanoic acid (**4b**). **4b** was prepared with the same synthetic method as **4a**, directly used in the next step without further purification.

Scheme S2. Synthesis of 2c

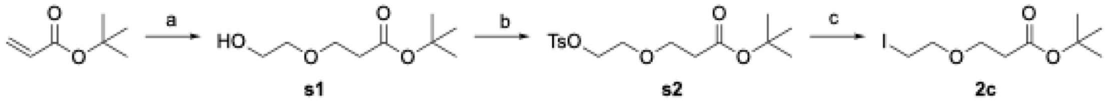

Reagents and conditions: (a) ethane-1,2-diol (5.0 equiv), Triton B (0.037 equiv), ACN, RT, 40 h; (b) *p*-TsCl (1.3 equiv), TEA (1.2 equiv), DMAP (0.25 equiv), DCM, RT, overnight; (c) NaI (2.0 equiv), acetone, 60 °C, 3h.

Tert-butyl 3-(2-hydroxyethoxy)propanoate (**s1**). To a solution of *tert*-butyl acrylate (4 g, 31.2 mmol, 1.0 equiv) in ACN (30 mL), ethane-1,2-diol (8.7 mL, 156.0 mmol, 5.0 equiv) and Triton B (190 mg, 1.2 mmol, 0.037 equiv) were added. The reaction mixture was stirred at RT for 40 h. After completion, remove the solvent in vacuo. The residue was dissolved in EA (3X), and washed with H_2O and brine, then dried over Na_2SO_4. The organic layer was concentrated under reduced pressure, and the residue was purified by column chromatography (PE: EA = 1: 1, v/v), affording the pure compound **s1** as a colorless oil (Yield: 28%). ^{1}H NMR (600 MHz, Chloroform-*d*) δ 3.69 (q, *J* = 5.5 Hz, 4H), 3.56 – 3.51 (m, 2H), 2.47 (t, *J* = 6.2 Hz, 2H), 1.98 (s, 1H), 1.42 (s, 9H). LC-MS (m/z): positive mode 191.1 $[M + H]^+$.

Tert-butyl 3-(2-(tosyloxy)ethoxy)propanoate (**s2**). To a solution of **s1** (2 g, 10.5 mmol, 1.0 equiv) in anhydrous DCM (15 mL) at 0 °C, TEA (1.8 mL, 12.6 mmol, 1.2 equiv) and a catalytic amount DMAP (0.3 g, 2.6 mmol, 0.25 equiv) were added. A solution of *p*-TsCl (2.6 g, 13.6 mmol, 1.3 equiv) in anhydrous DCM was then added dropwise. The reaction mixture was allowed to warm to RT and stirred overnight. After completion, the DCM layer was washed sequentially with H_2O and brine, then dried over Na_2SO_4. The DCM layer was concentrated under reduced pressure, and the residue was purified by column chromatography (PE: EA = 1: 1, v/v), affording the pure compound **s2** as a colorless oil (Yield: 92%). ^{1}H NMR (600 MHz, Chloroform-*d*) δ 7.79 (d, *J* = 8.3 Hz, 2H), 7.34 (dt, *J* = 8.0, 0.7 Hz, 2H), 4.15 – 4.11 (m, 2H), 3.65 – 3.61 (m, 4H), 2.44 (s, 3H), 2.41 (t, *J* = 6.4 Hz, 2H), 1.43 (s, 9H). LC-MS (m/z): positive mode 345.1 $[M + H]^+$.

Tert-butyl 3-(2-iodoethoxy)propanoate (**2c**). To a solution of **s2** (2 g, 5.8 mmol, 1.0 equiv) in acetone (15 mL), NaI (1.7 g, 11.6 mmol, 2.0 equiv) was added. The reaction mixture was stirred at 60 °C for 3 h. Upon completion, the reaction was cooled to RT, filtered, and solvent from the filtrate was removed in vacuo to yield crude **2c**, which was used directly in the next step.

Tert-butyl 3-(2-((2-(2,6-dioxopiperidin-3-yl)-1,3-dioxoisoindolin-4-yl)oxy)ethoxy)propanoate (**3c**). To a solution of **2c** (1.7 g, 5.8 mmol, 1.0 equiv) in NMP (30 mL), thalidomide-4-OH (3.2 g, 11.6 mmol, 2.0 equiv) and DIPEA (3.0 mL, 17.4 mmol, 3.0 equiv) were added. The reaction mixture was stirred at 110 °C for 24 h. Upon completion, the reaction mixture was extracted with DCM and washed sequentially with H_2O, 1M HCl and brine, then dried over Na_2SO_4. The DCM layer was concentrated under reduced pressure, and the residue was purified by column chromatography (DCM: MeOH = 25: 1, v/v), affording the pure compound **3c** as a white solid (Yield: 90%). ^{1}H NMR (600 MHz, Chloroform-*d*) δ 8.21 (s, 1H), 7.66 (dd, *J* = 8.5, 7.3 Hz, 1H), 7.46 (dd, *J* = 7.3, 0.7 Hz, 1H), 7.27 (dd, *J* = 8.5, 0.7 Hz, 1H), 4.95 (dd, *J* = 12.5, 5.4 Hz, 1H), 4.33 (dd, *J* = 5.7, 4.0 Hz, 2H), 3.92 – 3.88 (m, 2H), 3.82 (t, *J* = 6.4 Hz, 2H), 2.92 – 2.70 (m, 3H), 2.50 (t, *J* = 6.4 Hz, 2H), 2.14 – 2.09 (m, 1H), 1.42 (s, 9H). LC-MS (m/z): positive mode 447.2 $[M + H]^+$.

3-(2-((2-(2,6-dioxopiperidin-3-yl)-1,3-dioxoisoindolin-4-yl)oxy)ethoxy)propanoic acid (**4c**). **4c** was prepared with the same synthetic method as **4a**, directly used in the next step without further purification.

Scheme S3. Synthesis of 2d

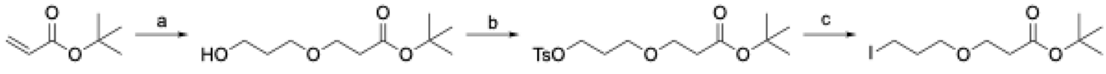

Reagents and conditions: (a) propane-1,3-diol (5.0 equiv), Triton B (0.037 equiv), ACN, RT, 40 h; (b) *p*-TsCl (1.3 equiv), TEA (1.2 equiv), DMAP (0.25 equiv), DCM, RT, overnight; (c) NaI (2.0 equiv), acetone, 60 °C, 3h.

Tert-butyl 3-(3-hydroxypropoxy)propanoate (**s3**). **s3** (Yield: 25%, white solid) was prepared with the same synthetic method as **s1**. ^{1}H NMR (600 MHz, Chloroform-*d*) δ 3.75 (t, *J* = 5.6 Hz, 2H), 3.68 (t, *J* = 6.2 Hz, 2H), 3.64 (t, *J* = 5.7 Hz, 2H), 2.49 (t, *J* = 6.2 Hz, 2H), 1.81 (p, *J* = 5.6 Hz, 2H), 1.46 (s, 9H). LC-MS (m/z): positive mode 205.1 [M + H]$^+$.

Tert-butyl 3-(3-(tosyloxy)propoxy)propanoate (**s4**). **s4** (Yield: 30%, white solid) was prepared with the same synthetic method as **s2**. ^{1}H NMR (600 MHz, Chloroform-*d*) δ 7.76 – 7.65 (m, 2H), 7.44 (d, *J* = 8.0, 2H), 4.16 (t, *J* = 7.2 Hz, 2H), 3.55 – 3.50 (m, 4H), 2.60 (t, *J* = 6.2 Hz, 2H), 2.39 (s, 3H), 2.10 – 2.00 (m, 2H), 1.40 (s, 9H). LC-MS (m/z): positive mode 359.2 [M + H]$^+$.

Tert-butyl 3-(3-iodopropoxy)propanoate (**2d**). **2d** was prepared with the same synthetic method as **2c**, directly used in the next step without further purification.

Tert-butyl 3-(3-((2-(2,6-dioxopiperidin-3-yl)-1,3-dioxoisoindolin-4-yl)amino)propoxy)propanoate (**3d**). **3d** (Yield: 25%, white solid) was prepared with the same synthetic method as **3c**. ^{1}H NMR (600 MHz, Chloroform-*d*) δ 7.42 (dd, *J* = 8.3, 7.1 Hz, 1H), 7.15 (dd, *J* = 7.1, 0.7 Hz, 1H), 6.87 (dd, *J* = 8.4, 0.7 Hz, 1H), 4.94 – 4.88 (m, 1H), 3.96 – 3.87 (m, 2H), 3.65 – 3.61 (m, 2H), 3.47 (t, *J* = 6.3 Hz, 2H), 3.00 – 2.90 (m, 1H), 2.78 – 2.72 (m, 2H), 2.51 – 2.45 (m, 3H), 2.11 – 2.06 (m, 1H), 1.86 – 1.79 (m, 3H), 1.44 (s, 9H). LC-MS (m/z): positive mode 460.2 [M + H]$^+$.

3-(3-((2-(2,6-dioxopiperidin-3-yl)-1,3-dioxoisoindolin-4-yl)amino)propoxy)propanoic acid (**4d**). **4d** was synthesized using the same method as **4a** and was used directly in the next step without further purification.

3-(3-((2-(2,6-dioxopiperidin-3-yl)-1,3-dioxoisoindolin-4-yl)oxy)propoxy)propanoic acid (**4e**). **4e** was synthesized using the same method as **4a** and was used directly in the next step without further purification.

Tert-butyl 4-(2-(2,6-dioxopiperidin-3-yl)-1,3-dioxoisoindolin-5-yl)piperazine-1-carboxylate (**6a**). To a solution of thalidomide 5-fluoride (1.0 equiv) in anhydrous DMSO (8 mL) was added 1-boc-piperazine (1.0 equiv) and DIPEA (1.1 equiv). The reaction mixture was heated to 90 °C and stirred for 14 h. After completion, the reaction mixture was extracted with EA and washed sequentially with H_2O and brine, then dried over Na_2SO_4. The EA layer was concentrated under reduced pressure, and the residue was purified by column chromatography (PE: EA = 2: 1, v/v), affording the pure compound **6a** as a green solid (Yield: 95%). ^{1}H NMR (600 MHz, Chloroform-*d*) δ 9.19 (s, 1H), 7.66 (d, *J* = 8.5 Hz, 1H), 7.26 (d, *J* = 2.5 Hz, 1H), 7.03 (dd, *J* = 8.5, 2.4 Hz, 1H), 5.04 – 4.91 (m, 1H), 3.50 (dt, *J* = 113.9, 5.2 Hz, 8H), 2.80 (ddd, *J* = 32.0, 14.7, 6.7 Hz, 3H), 2.14 – 2.06 (m, 1H), 1.48 (s, 9H). LC-MS (m/z): positive mode 443.2 $[M + H]^+$.

2-(2,6-dioxopiperidin-3-yl)-5-(piperazin-1-yl)isoindoline-1,3-dione (**7a**). To a solution of **6a** (580 mg, 1.3 mmol, 1.0 equiv) in anhydrous DCM was added 4M HCl in dioxane (6.5 mL, 26 mmol, 20 equiv) at 0 °C. Then it was allowed to warmed to RT and stirred for 2 h. After the reaction was completed, removed the solvent in vacuo. The obtained residue was directly used in the next step without further purification.

Tert-butyl 4-((3-((1,1-dioxidotetrahydrothiophen-3-yl)amino)-2,2-dimethylpropyl)amino)-4-oxobutanoate (**8a**). To a solution of **I-2** (500 mg, 2.3 mmol, 1.0 equiv) in anhydrous DMF (5 mL) were added 4-(*tert*-butoxy)-4-oxobutanoic acid (392 mg, 2.3 mmol, 1.0 equiv), HATU (1.3 g, 3.4 mmol, 1.5 equiv) and DIPEA (1.3 mL, 6.8 mmol, 3.0 equiv) at 0 °C. The reaction mixture was allowed to warm to RT and stirred overnight. After completion, the reaction mixture was extracted with DCM and washed sequentially with H_2O, 1M HCl, aqueous $NaHCO_3$ and brine, then dried over Na_2SO_4. The DCM layer was concentrated under reduced pressure, and the residue was purified by column chromatography (DCM: MeOH = 25: 1, v/v), affording the pure compound **8a** as a brown oil (Yield: 85%). ^{1}H NMR (600 MHz, Chloroform-*d*) δ 6.58 (t, *J* = 6.5 Hz, 1H), 3.47 (p, *J* = 5.7 Hz, 1H), 3.27 (dt, *J* = 13.0, 7.8 Hz, 1H), 3.14 (ddd, *J* = 28.5, 13.5, 6.5 Hz, 2H), 3.04 – 2.97 (m, 2H), 2.96 – 2.93 (m, 1H), 2.52 (t, *J* = 6.4 Hz, 2H), 2.41 (t, *J* = 6.9 Hz, 3H), 2.33 – 2.27 (m, 2H), 2.12 – 2.06 (m, 1H), 1.39 (s, 9H), 0.85 (d, *J* = 1.7 Hz, 6H). LC-MS (m/z): positive mode 377.2 $[M + H]^+$.

4-((3-((1,1-dioxidotetrahydrothiophen-3-yl)amino)-2,2-dimethylpropyl)amino)-4-oxobutanoic acid (**9a**). To a solution of **8a** (700 mg, 2.2 mmol, 1.0 equiv) in anhydrous DCM (8 mL) at 0 °C. TFA (20% v/v, 1.6 mL) was added at 0 °C. Then it was allowed to warmed to RT and stirred for 4 h. After the reaction was completed, removed the solvent in vacuo. The obtained residue was directly used in the next step without further purification.

4-(4-(*tert*-butoxycarbonyl)piperazin-1-yl)cyclohexane-1-carboxylic acid (**11a**). To a solution of 4-oxocyclohexane-1-carboxylic acid (1.2 g, 8.5 mmol, 1.0 equiv) in a

mixture of acetic acid and 1, 2-dichloroethane (1:10, v/v), 1-Boc-piperazine (1.6 g, 8.5 mmol, 1.0 equiv) was added. The mixture was stirred for 10 minutes, after which $NaBH(OAc)_3$ (2.2 g, 10.2 mmol, 1.2 equiv) was added. The reaction was then stirred at RT for 2 h. After completion, remove the solvent in vacuo and the obtained residue was purified by column chromatography (DCM: MeOH = 10: 1, v/v), affording the pure compound **11a** (Yield: 34%). ^{1}H NMR (400 MHz, Chloroform-*d*) δ 3.67 – 3.42 (m, 4H), 2.93 (t, *J* = 5.2 Hz, 4H), 2.70 – 2.65 (m, 1H), 2.18 (p, *J* = 6.6 Hz, 1H), 1.94 (m, 2H), 1.80 – 1.62 (m, 4H), 1.50 – 1.48 (m, 2H) 1.46 (s, 9H). LC-MS (m/z): negative mode 357.2 $[M + FA - H]^-$.

4-(4-(2-(2,6-dioxopiperidin-3-yl)-1,3-dioxoisoindolin-5-yl)piperazin-1-yl)cyclohexane-1-carboxylic acid (**12a**). To a solution of **11a** (940 mg, 1.3 mmol, 1.0 equiv) in anhydrous DCM was added TFA (2.0 mL, 26 mmol, 20 equiv) at 0 °C. Then it was allowed to warmed to RT and stirred for 3 h. After the reaction was completed, removed the solvent in vacuo. The obtained residue was directly used in the next step without further purification. To a solution of thalidomide 5-fluoride (1.0 equiv) in anhydrous DMSO (8 mL) was added obtained residue before (1.0 equiv) and DIPEA (3 equiv). The reaction mixture was heated to 90 °C and stirred for 10 h. After completion, the reaction mixture was extracted with EA and washed sequentially with H_2O and brine, then dried over Na_2SO_4. The EA layer was concentrated under reduced pressure, and the residue was purified by column chromatography (PE: EA = 2: 1, v/v), affording the pure compound **12a** as a green solid (Yield: 75%). ^{1}H NMR (400 MHz, DMSO-D_6) δ 11.10 (s, 1H), 7.67 (d, *J* = 8.5 Hz, 1H), 7.31 (d, *J* = 2.2 Hz, 1H),

7.23 (dd, *J* = 8.7, 2.3 Hz, 1H), 7.16 – 6.94 (m, 1H), 5.07 (dd, *J* = 12.9, 5.4 Hz, 1H), 3.40 (d, *J* = 4.7 Hz, 3H), 2.95 – 2.82 (m, 1H), 2.59 – 2.54 (m, 4H), 2.37 – 2.17 (m, 2H), 2.06 – 1.96 (m, 3H), 1.84 (s, 3H), 1.54 (s, 4H), 1.41 (s, 2H). LC-MS (m/z): negative mode 513.2 $[M + FA - H]^-$.

Tert-butyl (3-((1,1-dioxidotetrahydrothiophen-3-yl)amino)-2,2-dimethylpropyl) succinate (**8b**). To solution of **I-1** (828 mg, 3.7 mmol, 1.0 equiv) and 4-(*tert*-butoxy)-4-oxobutanoic acid (783 mg, 4.5 mmol, 1.2 equiv) in anhydrous DCM (10 mL) were added DCC (1.1 g, 5.6 mmol, 1.5 equiv), DMAP (45 mg, 0.4 mmol, 0.1 equiv) and TEA (1.6 mL, 11.1 mmol, 3.0 equiv). After stirred overnight at RT, the reaction mixture was washed with 1.0 M HCl, $NaHCO_3$ (aq.), brine and dried over Na_2SO_4. The DCM layer was concentrated, then the residue was purified by column chromatography (100% EA) to afford the pure compound **8b** as a white solid (Yield: 25%). ^1H NMR (600 MHz, Chloroform-*d*) δ 3.91 – 3.85 (m, 2H), 3.53 – 3.48 (m, 1H), 3.26 (dt, *J* = 14.8, 7.1 Hz, 2H), 3.02 (dt, *J* = 13.1, 7.7 Hz, 1H), 2.88 – 2.82 (m, 1H), 2.58 – 2.50 (m, 4H), 2.43 – 2.33 (m, 3H), 2.04 (dd, *J* = 13.6, 8.0 Hz, 1H), 1.43 (s, 9H), 0.90 (s, 6H). LC-MS (m/z): positive mode 378.2 $[M + H]^+$.

4-(3-((1,1-dioxidotetrahydrothiophen-3-yl)amino)-2,2-dimethylpropoxy)-4-oxo-butanoic acid (**9b**). **9b** was prepared with the same synthetic method as **9a**, directly used in the next step without further purification.

Tert-butyl 5-((3-((1,1-dioxidotetrahydrothiophen-3-yl)amino)-2,2-dimethylpropyl)amino)-5-oxopentanoate (**15a**). To a solution of **I-2** (500 mg, 2.3 mmol, 1.0 equiv) in anhydrous DMF (5 mL) were added 5-(*tert*-butoxy)-5-oxopentanoic acid (**14a**, 432

mg, 2.3 mmol, 1.0 equiv), HATU (1.3 g, 3.4 mmol, 1.5 equiv) and DIPEA (1.3 mL, 6.8 mmol, 3.0 equiv) at 0 °C. The reaction mixture was allowed to warm to RT and stirred overnight. After completion, the reaction mixture was extracted with DCM and washed sequentially with H_2O, 1M HCl, aqueous $NaHCO_3$ and brine, then dried over Na_2SO_4. The DCM layer was concentrated under reduced pressure, and the residue was purified by column chromatography (DCM: MeOH = 25: 1, v/v), affording the pure compound **15a** as a yellow oil (Yield: 85%). ^{1}H NMR (600 MHz, Chloroform-*d*) δ 6.78 (t, *J* = 6.4 Hz, 1H), 3.35 (p, *J* = 5.7 Hz, 1H), 3.13 (dt, *J* = 13.0, 7.8 Hz, 1H), 3.05 (dd, *J* = 13.3, 6.2 Hz, 1H), 2.98 (dd, *J* = 13.7, 6.8 Hz, 1H), 2.87 (ddt, *J* = 19.7, 13.4, 6.2 Hz, 2H), 2.81 (d, *J* = 5.3 Hz, 1H), 2.79 (d, *J* = 5.2 Hz, 1H), 2.22 (dtd, *J* = 12.8, 7.8, 4.8 Hz, 1H), 2.16 (s, 2H), 2.07 (dt, *J* = 10.5, 7.4 Hz, 4H), 2.01 – 1.94 (m, 1H), 1.72 (p, *J* = 7.6 Hz, 2H), 1.26 (s, 9H), 0.73 (d, *J* = 2.5 Hz, 6H). ^{13}C NMR (151 MHz, Chloroform-*d*) δ 172.69, 172.26, 79.94, 56.65, 55.63, 55.50, 50.23, 47.03, 35.17, 34.87, 34.44, 29.38, 27.82, 24.04, 23.99, 21.14. LC-MS (m/z): positive mode 391.2 $[M + H]^+$.

5-((3-(2-chloro-*N*-(1,1-dioxidotetrahydrothiophen-3-yl)acetamido)-2,2-dimethylpropyl)amino)-5-oxopentanoic acid (**16a**). To a solution of 1**5a** (470 mg, 1.2 mmol, 1.0 equiv) in anhydrous DCM (10 mL) was added DIPEA (0.6 mL, 3.6 mmol, 3.0 equiv). 2-chloroacetyl chloride (0.2 mL, 2.4 mmol, 2.0 equiv) in anhydrous DCM (1 mL) was added dropwise at 0 °C. Then it was allowed to warmed to RT and stirred for 2 h. After the reaction was completed, the DCM layer was washed with 1.0 M HCl, aqueous $NaHCO_3$, brine and dried over Na_2SO_4. The DCM layer was concentrated, then the residue was purified by column chromatography (DCM: MeOH = 20: 1, v/v) to

afford the pure compound as a yellow solid (Yield: 71%). The obtained solid was dissolved in anhydrous DCM (4 mL) at 0 °C. TFA (20% v/v, 0.8 mL) was added at 0 °C. Then it was allowed to warmed to RT and stirred for 4 h. After the reaction was completed, removed the solvent in vacuo. The obtained **16a** was directly used in the next step without further purification.

Tert-butyl 6-((3-((1,1-dioxidotetrahydrothiophen-3-yl)amino)-2,2-dimethylpropyl)amino)-6-oxohexanoate (**15b**). **15b** (65% yield, white solid) was prepared with the same synthetic method as **15a** using commercially available 6-(*tert*-butoxy)-6-oxohexanoic acid (**14b**). ^{1}H NMR (600 MHz, Chloroform-*d*) δ 6.51 (t, *J* = 6.0 Hz, 1H), 3.90 – 3.78 (m, 1H), 3.55 (dt, *J* = 13.0, 7.8 Hz, 1H), 3.27 – 3.15 (m, 2H), 3.01 (dd, *J* = 11.3, 6.0 Hz, 1H), 2.95 (dd, *J* = 14.1, 5.9 Hz, 1H), 2.87 (dd, *J* = 14.2, 4.7, 2H), 2.42 (dd, *J* = 12.1, 5.8, 1H), 2.23 (s, 2H), 2.05 (dt, *J* = 8.5, 5.4 Hz, 4H), 1.96 – 1.88 (m, 1H), 1.75 – 1.57 (m, 4H), 1.36 (s, 9H), 0.93 (d, *J* = 6.5 Hz, 6H). LC-MS (m/z): positive mode 405.2 $[M + H]^+$.

6-((3-(2-chloro-*N*-(1,1-dioxidotetrahydrothiophen-3-yl)acetamido)-2,2-dimethylpropyl)amino)-6-oxohexanoic acid (**16b**). **16b** was prepared with the same synthetic method as **16a**. The obtained **16b** was directly used in the next step without further purification.

Tert-butyl (3-((1,1-dioxidotetrahydrothiophen-3-yl)amino)-2,2-dimethylpropyl) glutarate (**15c**). To solution of **I-1** (290 mg, 1.3 mmol, 1.0 equiv) and 5-(*tert*-butoxy)-5-oxopentanoic acid (**14a**, 293 mg, 1.6 mmol, 1.2 equiv) in anhydrous DCM (10 mL) were added DCC (405 mg, 1.9 mmol, 1.5 equiv), DMAP (14 mg, 0.1 mmol, 0.1 equiv) and TEA (0.6 mL, 4 mmol, 3.0 equiv). After stirred overnight at RT, the reaction mixture

was washed with 1.0 M HCl, aqueous $NaHCO_3$, brine and dried over Na_2SO_4. The DCM layer was concentrated, then the residue was purified by column chromatography (PE: EA = 3: 7, v/v) to afford the pure compound **15c** as a white solid (Yield: 27%). ^{1}H NMR (400 MHz, Chloroform-*d*) δ 3.82 (d, *J* = 1.2 Hz, 2H), 3.47 (qd, *J* = 6.6, 5.1 Hz, 1H), 3.27 – 3.18 (m, 2H), 2.98 (dtd, *J* = 13.1, 7.7, 0.8 Hz, 1H), 2.81 (ddt, *J* = 13.3, 6.3, 0.9 Hz, 1H), 2.38 – 2.29 (m, 5H), 2.22 (t, *J* = 7.3 Hz, 2H), 2.01 (dtd, *J* = 13.6, 7.3, 0.8 Hz, 1H), 1.90 – 1.81 (m, 2H), 1.39 (s, 9H), 0.86 (s, 6H). LC-MS (m/z): positive mode 392.2 $[M + H]^+$.

5-(3-(2-chloro-*N*-(1,1-dioxidotetrahydrothiophen-3-yl)acetamido)-2,2-dimethylpropoxy)-5-oxopentanoic acid (**16c**). **16c** was prepared with the same synthetic method as **16a**. The obtained **16c** was directly used in the next step without further purification.

Tert-butyl (3-((1,1-dioxidotetrahydrothiophen-3-yl)amino)-2,2-dimethylpropyl) adipate (**15d**). **15d** (58% yield, colorless oil) was prepared with the same synthetic method as **15c**. ^{1}H NMR (400 MHz, Chloroform-*d*) δ 4.04 (q, *J* = 7.2 Hz, 2H), 3.85 (s, 2H), 3.65 (t, *J* = 6.2 Hz, 2H), 3.44 (dt, *J* = 8.5, 6.1 Hz, 3H), 3.26 – 3.16 (m, 4H), 3.01 – 2.91 (m, 1H), 2.83 – 2.76 (m, 1H), 2.52 (t, *J* = 6.2 Hz, 2H), 2.33 – 2.27 (m, 1H), 2.04 – 1.97 (m, 1H), 1.36 (s, 9H), 0.85 (s, 6H). LC-MS (m/z): positive mode 406.2 $[M + H]^+$.

6-(3-(2-chloro-*N*-(1,1-dioxidotetrahydrothiophen-3-yl)acetamido)-2,2-dimethylpropoxy)-6-oxohexanoic acid (**16d**). **16d** was prepared with the same synthetic method as **16a**. The obtained **16d** was directly used in the next step without further purification.

Tert-butyl (7-((3-((1,1-dioxidotetrahydrothiophen-3-yl)amino)-2,2-dimethylpropyl)amino)-7-oxoheptyl)carbamate (**18a**). **18a** (Yield: 74%, colorless oil) was prepared with the same synthetic method as **15a** using commercially available 7-((*tert*-butoxycarbonyl)amino)heptanoic acid (**17a**). ^{1}H NMR (600 MHz, Chloroform-*d*) δ 6.49 (t, *J* = 6.3 Hz, 1H), 4.62 (t, *J* = 6.1 Hz, 1H), 3.52 (p, *J* = 5.7 Hz, 1H), 3.29 (dt, *J* = 13.0, 7.8 Hz, 1H), 3.21 (dd, *J* = 13.3, 6.2 Hz, 1H), 3.17 – 3.13 (m, 1H), 3.08 – 2.93 (m, 6H), 2.39 (dtd, *J* = 12.9, 7.8, 5.0 Hz, 1H), 2.15 (dt, *J* = 19.2, 7.1 Hz, 5H), 1.62 – 1.57 (m, 2H), 1.44 (t, *J* = 6.4 Hz, 2H), 1.41 (s, 9H), 1.32 – 1.29 (m, 4H), 0.89 (d, *J* = 2.2 Hz, 6H). LC-MS (m/z): positive mode 448.3 $[M + H]^+$.

7-amino-*N*-(3-(2-chloro-*N*-(1,1-dioxidotetrahydrothiophen-3-yl)acetamido)-2,2-dimethylpropyl)heptanamide (**19a**). **19a** (brown solid) was prepared with the same synthetic method as **16a**. The obtained **19a** was directly used in the next step without further purification.

Tert-butyl (2-(3-((3-((1,1-dioxidotetrahydrothiophen-3-yl)amino)-2,2-dimethylpropyl)amino)-3-oxopropoxy)ethyl)carbamate (**18b**). **18b** (Yield: 66%, yellow solid) was prepared with the same synthetic method as **15a** using commercially available 3-(2-((*tert*-butoxycarbonyl)amino)ethoxy)propanoic acid (**17b**). ^{1}H NMR (600 MHz, Chloroform-*d*) δ 6.70 (t, *J* = 6.6 Hz, 1H), 4.97 (s, 1H), 3.69 (t, *J* = 5.8 Hz, 2H), 3.50 (q, *J* = 5.3 Hz, 3H), 3.32 – 3.25 (m, 3H), 3.18 (ddd, *J* = 15.9, 13.5, 6.5 Hz, 2H), 3.04 (dt, *J* = 13.6, 6.6 Hz, 2H), 2.94 (dd, *J* = 13.4, 5.4 Hz, 1H), 2.43 (td, *J* = 5.6, 2.9 Hz, 2H), 2.40 – 2.34 (m, 1H), 2.33 – 2.27 (m, 2H), 2.11 (dq, *J* = 13.6, 6.7 Hz, 1H), 1.92 (s, 1H), 1.41 (s, 9H), 0.89 (d, *J* = 1.3 Hz, 6H). LC-MS (m/z): positive mode 436.2 $[M + H]^+$.

3-(2-aminoethoxy)-*N*-(3-(2-chloro-*N*-(1,1-dioxidotetrahydrothiophen-3-yl)acetamido)-2,2-dimethylpropyl)propanamide (**19b**). **19b** (Yield: 63%, brown solid) was prepared with the same synthetic method as **16a**. The obtained **19b** was directly used in the next step without further purification.

Tert-butyl (6-((3-((1,1-dioxidotetrahydrothiophen-3-yl)amino)-2,2-dimethylpropyl)amino)-6-oxohexyl)carbamate (**18c**). **18c** (Yield: 98%, yellow solid) was prepared with the same synthetic method as **15a** using commercially available 6-((*tert*-butoxycarbonyl)amino)hexanoic acid (**17c**). ^{1}H NMR (600 MHz, Chloroform-*d*) 6.51 (t, *J* = 6.5 Hz, 1H), 4.63 (s, 1H), 3.70 (hept, *J* = 6.7 Hz, 1H), 3.53 (p, *J* = 5.5 Hz, 1H), 3.29 (dt, *J* = 13.0, 8.0 Hz, 1H), 3.22 – 3.14 (m, 3H), 3.09 – 3.03 (m, 3H), 3.00 (dd, *J* = 13.8, 5.8 Hz, 1H), 2.98 – 2.94 (m, 1H), 2.39 (dtd, *J* = 13.0, 7.9, 4.8 Hz, 1H), 2.33 (s, 2H), 2.18 (dd, *J* = 8.1, 6.8 Hz, 2H), 1.64 – 1.60 (m, 2H), 1.47 (t, *J* = 7.4 Hz, 2H), 1.42 – 1.41 (m, 9H), 1.34 – 1.30 (m, 2H), 0.89 (d, *J* = 3.4 Hz, 6H). LC-MS (m/z): positive mode 434.3 $[M + H]^+$.

6-amino-*N*-(3-(2-chloro-*N*-(1,1-dioxidotetrahydrothiophen-3-yl)acetamido)-2,2-dimethylpropyl)hexanamide (**19c**). **19c** (Yield: 87%, brown solid) was prepared with the same synthetic method as **16a**. The obtained **19c** was directly used in the next step without further purification.

Tert-butyl (5-((3-((1,1-dioxidotetrahydrothiophen-3-yl)amino)-2,2-dimethylpropyl)amino)-5-oxopentyl)carbamate (**18d**). **18d** (Yield: 99%, white solid) was prepared with the same synthetic method as **15a** using commercially available 5-((*tert*-butoxycarbonyl)amino)pentanoic acid (**17d**). ^{1}H NMR (600 MHz, Chloroform-*d*) δ 6.60 (s, 1H), 4.73 (d, *J* = 6.4 Hz, 1H), 3.50 (p, *J* = 5.4 Hz, 1H), 3.28 (dt, *J* = 13.0, 8.0 Hz,

1H), 3.18 (dt, *J* = 12.2, 6.0 Hz, 2H), 3.14 – 3.02 (m, 3H), 2.98 (ddd, *J* = 13.4, 5.3, 3.4 Hz, 2H), 2.36 (dtd, *J* = 13.0, 8.0, 4.7 Hz, 1H), 2.32 – 2.29 (m, 2H), 2.20 (td, *J* = 7.4, 2.1 Hz, 2H), 2.16 – 2.11 (m, 1H), 1.63 (pd, *J* = 7.2, 4.5 Hz, 2H), 1.48 (p, *J* = 7.2 Hz, 2H), 1.40 (s, 9H), 0.88 (d, *J* = 1.5 Hz, 6H). LC-MS (m/z): positive mode 420.3 $[M + H]^+$.

5-amino-*N*-(3-(2-chloro-*N*-(1,1-dioxidotetrahydrothiophen-3-yl)acetamido)-2,2-dimethylpropyl)pentanamide (**19d**). **19d** (Yield: 61%, brown solid) was prepared with the same synthetic method as **16a**. The obtained **19d** was directly used in the next step without further purification.

3-((1,1-dioxidotetrahydrothiophen-3-yl)amino)-2,2-dimethylpropyl 7-((*tert*-butoxycarbonyl)amino)heptanoate (**18e**). **18e** (Yield: 98%, white solid) was prepared with the same synthetic method as **15c** using commercially available 7-((*tert*-butoxycarbonyl)amino)heptanoic acid (**17a**). ^{1}H NMR (400 MHz, Chloroform-*d*) δ 4.77 (t, *J* = 5.9 Hz, 1H), 3.71 (s, 2H), 3.46 – 3.34 (m, 1H), 3.13 (ddd, *J* = 13.7, 9.4, 6.9 Hz, 2H), 2.99 – 2.84 (m, 3H), 2.73 (dd, *J* = 13.2, 6.3 Hz, 1H), 2.29 – 2.20 (m, 3H), 2.16 (t, *J* = 7.5 Hz, 2H), 1.97 – 1.89 (m, 1H), 1.46 (qt, *J* = 9.3, 5.0 Hz, 2H), 1.27 (s, 12H), 1.21 – 1.14 (m, 4H), 0.76 (s, 6H). LC-MS (m/z): positive mode 449.3 $[M + H]^+$.

3-(2-chloro-*N*-(1,1-dioxidotetrahydrothiophen-3-yl)acetamido)-2,2-dimethylpropyl 7-aminoheptanoate (**19e**). **19e** (Yield: 39%, colorless oil) was prepared with the same synthetic method as **16a**. The obtained **19e** was directly used in the next step without further purification.

3-((1,1-dioxidotetrahydrothiophen-3-yl)amino)-2,2-dimethylpropyl 3-(2-((*tert*-butoxycarbonyl)amino)ethoxy)propanoate (**18f**). **18f** (Yield: 50%, white solid) was

prepared with the same synthetic method as **15c** using commercially available 3-(2-((*tert*-butoxycarbonyl)amino)ethoxy)propanoic acid (**17b**). ^{1}H NMR (400 MHz, Chloroform-*d*) δ 3.88 (d, *J* = 1.6 Hz, 2H), 3.56 – 3.50 (m, 1H), 3.28 (dt, *J* = 14.0, 7.1 Hz, 2H), 3.09 – 3.00 (m, 1H), 2.87 (dd, *J* = 13.2, 6.2 Hz, 1H), 2.46 – 2.32 (m, 5H), 2.24 (t, *J* = 7.0 Hz, 2H), 2.12 – 2.05 (m, 1H), 1.64 (tdt, *J* = 7.7, 6.5, 4.4 Hz, 4H), 1.44 (s, 9H), 0.92 (s, 6H). LC-MS (m/z): positive mode 437.2 $[M + H]^{+}$.

3-(2-chloro-*N*-(1,1-dioxidotetrahydrothiophen-3-yl)acetamido)-2,2-dimethylpropyl 3-(2-aminoethoxy)propanoate (**19f**). **19f** (Yield: 65%, colorless oil) was prepared with the same synthetic method as **16a**. The obtained **19f** was directly used in the next step without further purification.

3-((1,1-dioxidotetrahydrothiophen-3-yl)amino)-2,2-dimethylpropyl 6-((*tert*-butoxycarbonyl)amino)hexanoate (**18g**). **18g** (Yield: 31%, white solid) was prepared with the same synthetic method as **15c** using commercially available 6-((*tert*-butoxycarbonyl)amino)hexanoic acid (**17c**). ^{1}H NMR (400 MHz, Chloroform-*d*) δ 3.88 (s, 2H), 3.57 – 3.47 (m, 1H), 3.34 – 3.23 (m, 2H), 3.14 – 2.99 (m, 3H), 2.86 (dd, *J* = 13.3, 6.2 Hz, 1H), 2.45 – 2.37 (m, 3H), 2.33 (t, *J* = 7.5 Hz, 2H), 2.12 – 2.05 (m, 1H), 1.68 – 1.61 (m, 2H), 1.50 (dt, *J* = 14.3, 7.7 Hz, 3H), 1.44 (s, 9H), 1.40 – 1.35 (m, 2H), 1.34 (s, 1H), 0.92 (d, *J* = 2.1 Hz, 6H). LC-MS (m/z): positive mode 435.3 $[M + H]^{+}$.

3-(2-chloro-*N*-(1,1-dioxidotetrahydrothiophen-3-yl)acetamido)-2,2-dimethylpropyl 6-aminohexanoate (**19g**). **19g** (Yield: 57%, colorless oil) was prepared with the same synthetic method as **16a**. The obtained **19g** was directly used in the next step without further purification.

3-((1,1-dioxidotetrahydrothiophen-3-yl)amino)-2,2-dimethylpropyl 5-((*tert*-butoxycarbonyl)amino)pentanoate (**18h**). **18h** (Yield: 30%, white solid) was prepared with the same synthetic method as **15c** using commercially available 5-((*tert*-butoxycarbonyl)amino)pentanoic acid (**17d**). ^{1}H NMR (400 MHz, Chloroform-*d*) δ 4.70 (s, 1H), 3.88 (s, 2H), 3.54 (dd, *J* = 12.3, 6.1 Hz, 1H), 3.34 – 3.25 (m, 2H), 3.18 – 3.00 (m, 3H), 2.89 – 2.82 (m, 1H), 2.46 – 2.30 (m, 5H), 2.08 (d, *J* = 6.9 Hz, 1H), 1.69 – 1.63 (m, 2H), 1.56 – 1.49 (m, 2H), 1.44 (s, 9H), 0.92 (s, 6H). LC-MS (m/z): positive mode 421.2 $[M + H]^+$.

3-(2-chloro-*N*-(1,1-dioxidotetrahydrothiophen-3-yl)acetamido)-2,2-dimethylpropyl 5-aminopentanoate (**19h**). **19h** (Yield: 76%, colorless oil) was prepared with the same synthetic method as **16a**. The obtained **19h** was directly used in the next step without further purification.

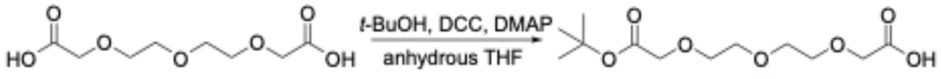

13,13-dimethyl-11-oxo-3,6,9,12-tetraoxatetradecanoic acid (**14c**). To solution of 2,2'-((oxybis(ethane-2,1-diyl))bis(oxy))diacetic acid (400 mg, 1.8 mmol, 1.0 equiv) in anhydrous THF (8 mL), were added *t*-BuOH (1.3 g, 18 mmol, 10.0 equiv), DCC (0.5 g, 2.1 mmol, 1.2 equiv) and DMAP (11 mg, 0.09 mmol, 0.05 equiv). After stirred 24 h at RT, filtered, and solvent from the filtrate was removed in vacuo. Then the residue was dissolved in DCM, and washed with H_2O and brine, then dried over Na_2SO_4. The organic layer was concentrated under reduced pressure, and the residue was directly used in the next step without further purification (white solid, Yield: 96%).

Tert-butyl 15-((1,1-dioxidotetrahydrothiophen-3-yl)amino)-14,14-dimethyl-11-oxo-3,6,9-trioxa-12-azapentadecanoate (**15e**). **15e** (Yield: 33%, colorless oil) was prepared with the same synthetic method as **15a**. ^{1}H NMR (600 MHz, Chloroform-*d*) δ 7.38 (t, *J* = 6.6 Hz, 1H), 4.00 (s, 2H), 3.99 (s, 2H), 3.68 (dd, *J* = 4.4, 1.1 Hz, 9H), 3.48 (td, *J* = 6.6, 5.2 Hz, 1H), 3.32 – 3.27 (m, 1H), 3.23 (dd, *J* = 13.2, 6.6 Hz, 1H), 3.13 (d, *J* = 6.5 Hz, 2H), 3.02 (dt, *J* = 13.1, 7.4 Hz, 1H), 2.90 (dd, *J* = 13.3, 6.1 Hz, 1H), 2.41 – 2.36 (m, 1H), 2.33 – 2.28 (m, 2H), 2.10 (dt, *J* = 13.8, 7.1 Hz, 1H), 1.46 (s, 9H), 0.91 (d, *J* = 1.9 Hz, 6H). LC-MS (m/z): positive mode 481.3 $[M + H]^+$.

18-chloro-16-(1,1-dioxidotetrahydrothiophen-3-yl)-14,14-dimethyl-11,17-dioxo-3,6,9,12-tetraoxa-16-azaoctadecanoic acid (**16e**). **16e** (Yield: 52%, colorless oil) was prepared with the same synthetic method as **16a**. The obtained **16e** was directly used in the next step without further purification.

Tert-butyl 4-(4-((3-((1,1-dioxidotetrahydrothiophen-3-yl)amino)-2,2-dimethylpropyl)carbamoyl)phenyl)piperazine-1-carboxylate (**18i**). **18i** (Yield: 64%, white solid) was prepared with the same synthetic method as **15a** using commercially available 4-(4-(*tert*-butoxycarbonyl)piperazin-1-yl)benzoic acid (**17e**). ^{1}H NMR (600 MHz, Chloroform-*d*) δ 7.69 (d, *J* = 8.9 Hz, 2H), 7.06 (t, *J* = 6.2 Hz, 1H), 6.88 – 6.84 (m, 2H), 3.56 – 3.52 (m, 4H), 3.52 – 3.47 (m, 1H), 3.34 – 3.20 (m, 8H), 3.02 (dt, *J* = 12.9, 7.5 Hz, 1H), 2.91 (dd, *J* = 13.3, 5.9 Hz, 1H), 2.43 – 2.36 (m, 3H), 2.12 – 2.06 (m, 1H), 1.46 (s, 9H), 0.94 (d, *J* = 2.1 Hz, 6H). LC-MS (m/z): positive mode 509.3 $[M + H]^+$.

N-(3-(2-chloro-*N*-(1,1-dioxidotetrahydrothiophen-3-yl)acetamido)-2,2-dimethylpropyl)-4-(piperazin-1-yl)benzamide (**19i**). **19i** (Yield: 68%, colorless oil) was

prepared with the same synthetic method as **16a**. The obtained **19i** was directly used in the next step without further purification.

Tert-butyl 4-(4-((3-((1,1-dioxidotetrahydrothiophen-3-yl)amino)-2,2-dimethylpropoxy)carbonyl)phenyl)piperazine-1-carboxylate (**18l**). **18l** (Yield: 21%, white solid) was prepared with the same synthetic method as **15c** using commercially available 4-(4-(*tert*-butoxycarbonyl)piperazin-1-yl)benzoic acid (**17e**). ^{1}H NMR (600 MHz, Chloroform-*d*) δ 7.91 (d, *J* = 8.9 Hz, 2H), 6.87 (d, *J* = 9.0 Hz, 2H), 4.07 (d, *J* = 3.7 Hz, 2H), 3.58 (t, *J* = 5.3 Hz, 4H), 3.54 – 3.50 (m, 1H), 3.33 – 3.24 (m, 6H), 3.01 (dt, *J* = 13.2, 7.7 Hz, 1H), 2.83 (dd, *J* = 13.1, 6.5 Hz, 1H), 2.52 – 2.43 (m, 2H), 2.38 (ddd, *J* = 18.8, 11.7, 5.0 Hz, 1H), 2.08 – 1.99 (m, 2H), 1.48 (s, 9H), 1.00 (d, *J* = 4.0 Hz, 6H). LC-MS (m/z): positive mode 510.3 $[M + H]^+$.

3-(2-chloro-*N*-(1,1-dioxidotetrahydrothiophen-3-yl)acetamido)-2,2-dimethylpropyl 4-(piperazin-1-yl)benzoate (**19l**). **19l** (Yield: 86%, colorless oil) was prepared with the same synthetic method as **16a**. The obtained **19l** was directly used in the next step without further purification.

Tert-butyl 4-(2-(3-((1,1-dioxidotetrahydrothiophen-3-yl)amino)-2,2-dimethylpropoxy)-2-oxoethyl)piperazine-1-carboxylate (**18m**). **18m** (Yield: 38%, white solid) was prepared with the same synthetic method as **15c** using commercially available 2-(4-(*tert*-butoxycarbonyl)piperazin-1-yl)acetic acid (**17f**). ^{1}H NMR (600 MHz, Chloroform-*d*) δ 3.93 (s, 2H), 3.54 (ddd, *J* = 24.3, 12.1, 6.2 Hz, 2H), 3.28 (dt, *J* = 12.8, 6.4 Hz, 3H), 3.23 (d, *J* = 3.2 Hz, 2H), 3.11 – 2.99 (m, 2H), 2.96 – 2.92 (m, 1H), 2.86 (dd, *J* =

13.3, 6.1 Hz, 1H), 2.56 (d, *J* = 4.4 Hz, 2H), 2.48 – 2.36 (m, 4H), 2.13 – 2.01 (m, 3H), 1.45 (s, 9H), 0.91 (d, *J* = 1.3 Hz, 6H). LC-MS (m/z): positive mode 448.2 $[M + H]^+$.

3-(2-chloro-*N*-(1,1-dioxidotetrahydrothiophen-3-yl)acetamido)-2,2-dimethylpropyl 2-(piperazin-1-yl)acetate (**19m**). **19m** (Yield: 59%, colorless oil) was prepared with the same synthetic method as **16a**. The obtained **19m** was directly used in the next step without further purification.

Tert-butyl 4-(3-(3-((1,1-dioxidotetrahydrothiophen-3-yl)amino)-2,2-dimethylpropoxy)-3-oxopropyl)piperidine-1-carboxylate (**18n**). **18n** (Yield: 32%, white solid) was prepared with the same synthetic method as **15c** using commercially available 3-(4-(*tert*-butoxycarbonyl)piperazin-1-yl)propanoic acid (**17g**). ^{1}H NMR (600 MHz, Chloroform-*d*) δ 4.20 – 3.98 (m, 2H), 3.87 (s, 2H), 3.54 – 3.49 (m, 1H), 3.26 (ddt, *J* = 14.0, 7.2, 3.8 Hz, 2H), 3.03 (dt, *J* = 13.1, 7.7 Hz, 1H), 2.84 (dd, *J* = 13.2, 6.2 Hz, 1H), 2.65 (s, 2H), 2.42 – 2.37 (m, 3H), 2.36 – 2.31 (m, 2H), 2.07 – 2.01 (m, 1H), 1.66 (s, 2H), 1.57 (q, *J* = 7.5 Hz, 2H), 1.44 (s, 10H), 1.09 (qd, *J* = 12.3, 4.6 Hz, 3H), 0.90 (d, *J* = 1.0 Hz, 6H). LC-MS (m/z): positive mode 461.3 $[M + H]^+$.

3-(2-chloro-*N*-(1,1-dioxidotetrahydrothiophen-3-yl)acetamido)-2,2-dimethylpropyl 3-(piperidin-4-yl)propanoate (**19n**). **19n** (Yield: 89%, colorless oil) was prepared with the same synthetic method as **16a**. The obtained **19n** was directly used in the next step without further purification.

2-(3-(2-(*tert*-butoxy)-2-oxoethyl)phenyl)acetic acid (**14d**). **14d** (Yield: 57%, white solid) was prepared with the same synthetic method as **14c**, using commercially available 2,2'-(1,3-phenylene)diacetic acid. ^{1}H NMR (600 MHz, Chloroform-*d*) δ 7.12 – 7.07

(m, 2H), 6.99 – 6.90 (m, 2H), 3.61 (q, *J* = 4.5 Hz, 2H), 3.54 – 3.50 (m, 1H), 3.43 (t, *J* = 6.2 Hz, 1H), 1.35 (d, *J* = 4.9 Hz, 9H). LC-MS (m/z): negative mode 295.1 [M + FA - H]$^-$.

Tert-butyl 2-(3-(2-((3-((1,1-dioxidotetrahydrothiophen-3-yl)amino)-2,2-dimethylpropyl)amino)-2-oxoethyl)phenyl)acetate (**15f**). **15f** (Yield: 91%, brown oil) was prepared with the same synthetic method as **15a**. ^{1}H NMR (600 MHz, Chloroform-*d*) δ 7.31 (t, *J* = 7.7 Hz, 1H), 7.18 – 7.14 (m, 3H), 6.50 (t, *J* = 6.2 Hz, 1H), 3.55 (d, *J* = 19.1 Hz, 4H), 3.30 (qd, *J* = 6.7, 5.1 Hz, 1H), 3.15 (dtd, *J* = 13.7, 7.2, 6.5, 5.2 Hz, 2H), 3.04 (td, *J* = 13.8, 6.2 Hz, 2H), 2.95 (dt, *J* = 13.1, 7.7 Hz, 1H), 2.79 (s, 1H), 2.64 (dd, *J* = 13.2, 6.6 Hz, 1H), 2.25 – 2.17 (m, 3H), 1.85 (dt, *J* = 13.5, 7.6 Hz, 1H), 1.43 (s, 9H), 0.80 (d, *J* = 8.9 Hz, 6H). LC-MS (m/z): positive mode 453.2 [M + H]$^+$.

2-(3-(2-((3-(2-chloro-*N*-(1,1-dioxidotetrahydrothiophen-3-yl)acetamido)-2,2-dimethylpropyl)amino)-2-oxoethyl)phenyl)acetic acid (**16f**). **16f** (Yield: 91%, brown oil) was prepared with the same synthetic method as **16a**. The obtained **16f** was directly used in the next step without further purification.

2-(4-(2-(*tert*-butoxy)-2-oxoethyl)phenyl)acetic acid (**14e**). **14e** (Yield: 25%, white solid) was prepared with the same synthetic method as **14c**, using commercially available 2,2'-(1,4-phenylene)diacetic acid. ^{1}H NMR (600 MHz, Chloroform-*d*) δ 7.22 – 6.96 (m, 4H), 3.78 – 3.36 (m, 4H), 1.43 (dq, *J* = 8.2, 4.7 Hz, 9H). LC-MS (m/z): negative mode 295.1 [M + FA - H]$^-$.

Tert-butyl 2-(4-(2-((3-((1,1-dioxidotetrahydrothiophen-3-yl)amino)-2,2-dimethylpropyl)amino)-2-oxoethyl)phenyl)acetate (**15g**). **15g** (Yield: 55%, white solid) was prepared with the same synthetic method as **15a.** ^{1}H NMR (600 MHz, Chloroform-*d*)

δ 7.23 (d, *J* = 2.3 Hz, 4H), 6.24 (d, *J* = 6.6 Hz, 1H), 3.53 (d, *J* = 15.1 Hz, 4H), 3.39 – 3.34 (m, 1H), 3.21 (dt, *J* = 14.8, 5.9 Hz, 1H), 3.11 (td, *J* = 14.0, 6.6 Hz, 3H), 3.00 (ddd, *J* = 13.1, 6.8, 2.8 Hz, 2H), 2.72 (dd, *J* = 13.3, 6.0 Hz, 1H), 2.32 – 2.25 (m, 1H), 2.20 (d, *J* = 8.2 Hz, 2H), 1.95 (dq, *J* = 14.4, 7.4 Hz, 1H), 1.43 (s, 9H), 0.80 (d, *J* = 5.0 Hz, 6H). LC-MS (m/z): positive mode 453.2 $[M + H]^+$.

2-(4-(2-((3-(2-chloro-*N*-(1,1-dioxidotetrahydrothiophen-3-yl)acetamido)-2,2-dimethylpropyl)amino)-2-oxoethyl)phenyl)acetic acid (**16g**). **16g** (Yield: 70%, brown oil) was prepared with the same synthetic method as **16a.** The obtained **16g** was directly used in the next step without further purification.

Tert-butyl 4-(2-((3-((1,1-dioxidotetrahydrothiophen-3-yl)amino)-2,2-dimethylpropyl)amino)-2-oxoethyl)piperazine-1-carboxylate (**18j**). **18j** (Yield: 99%, yellow oil) was prepared with the same synthetic method as **15a** using commercially available 2-(4-(*tert*-butoxycarbonyl)piperazin-1-yl)acetic acid (**17f**). ^{1}H NMR (600 MHz, Chloroform-*d*) δ 7.36 (t, *J* = 6.8 Hz, 1H), 3.50 – 3.46 (m, 1H), 3.44 (d, *J* = 5.2 Hz, 4H), 3.29 (dt, *J* = 13.0, 7.4 Hz, 1H), 3.23 (dd, *J* = 13.2, 6.5 Hz, 1H), 3.14 (dd, *J* = 13.8, 6.9 Hz, 1H), 3.08 (dd, *J* = 13.8, 6.7 Hz, 1H), 3.04 – 2.99 (m, 3H), 2.87 (dd, *J* = 13.2, 6.1 Hz, 1H), 2.79 (s, 1H), 2.48 (t, *J* = 5.0 Hz, 4H), 2.41 – 2.35 (m, 1H), 2.26 (q, *J* = 11.7 Hz, 2H), 2.07 (dq, *J* = 14.4, 7.4 Hz, 1H), 1.45 (s, 9H), 0.90 (s, 6H). LC-MS (m/z): positive mode 447.3 $[M + H]^+$.

2-chloro-*N*-(2,2-dimethyl-3-(2-(piperazin-1-yl)acetamido)propyl)-*N*-(1,1-dioxidotetrahydrothiophen-3-yl)acetamide (**19j**). **19j** (Yield: 43%, brown solid) was prepared with the same synthetic method as **16a**. The obtained **19j** was directly used in the next step without further purification. LC-MS (m/z): positive mode 423.2 $[M + H]^+$.

Tert-butyl 4-(3-((3-((1,1-dioxidotetrahydrothiophen-3-yl)amino)-2,2-dimethylpropyl)amino)-3-oxopropyl)piperidine-1-carboxylate (**18k**). **18k** (Yield: 85%, colorless oil) was prepared with the same synthetic method as **15a** using commercially available 3-(4-(*tert*-butoxycarbonyl)piperazin-1-yl)propanoic acid (**17g**). ^{1}H NMR (600 MHz, Chloroform-*d*) δ 6.36 (t, *J* = 6.4 Hz, 1H), 4.13 (s, 1H), 3.59 (p, *J* = 5.3 Hz, 1H), 3.53 (q, *J* = 7.0 Hz, 1H), 3.36 (dt, *J* = 13.0, 8.1 Hz, 1H), 3.26 (td, *J* = 13.1, 6.5 Hz, 2H), 3.13 (ddd, *J* = 13.3, 7.9, 5.8 Hz, 1H), 3.07 (dd, *J* = 13.8, 5.8 Hz, 1H), 3.02 (dd, *J* = 13.3, 4.8 Hz, 1H), 2.70 (d, *J* = 13.9 Hz, 2H), 2.45 (dtd, *J* = 13.1, 8.1, 4.7 Hz, 1H), 2.41 – 2.35 (m, 2H), 2.27 (t, *J* = 7.8 Hz, 2H), 2.20 (dq, *J* = 13.4, 6.7 Hz, 1H), 1.74 – 1.68 (m, 3H), 1.63 (q, *J* = 7.5 Hz, 2H), 1.50 (s, 9H), 1.48 – 1.43 (m, 1H), 1.19 – 1.11 (m, 2H), 0.96 (d, *J* = 2.3 Hz, 6H). LC-MS (m/z): positive mode 460.3 $[M + H]^{+}$.

N-(3-(2-chloro-*N*-(1,1-dioxidotetrahydrothiophen-3-yl)acetamido)-2,2-dimethylpropyl)-3-(piperidin-4-yl)propanamide (**19k**). **19k** (Yield: 63%, brown solid) was prepared with the same synthetic method as **16a**. The obtained **19k** was directly used in the next step without further purification. LC-MS (m/z): positive mode 463.2 $[M + H]^{+}$.

Scheme S4. Synthesis of PC2-Neg.

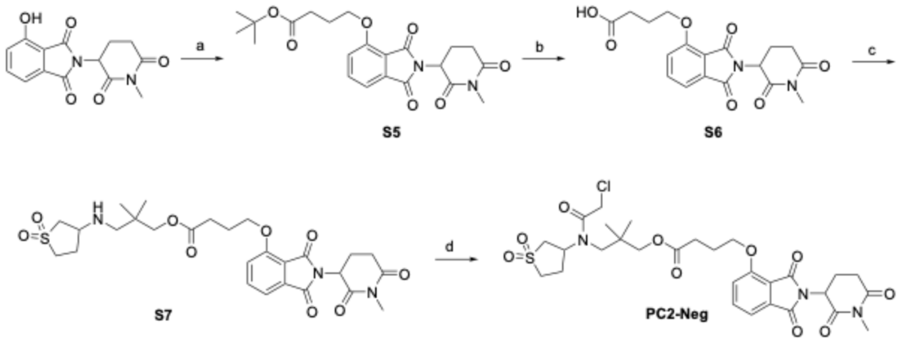

Reagents and conditions: (a) **2b**, DIPEA, DMF, 70 °C; (b) TFA, anhydrous DCM; (c) **I-1**, DCC, DMAP, TEA, DMF; (d) chloroacetyl chloride, DIPEA, DCM.

Tert-butyl 4-((2-(1-methyl-2,6-dioxopiperidin-3-yl)-1,3-dioxoisoindolin-4-yl)oxy)butanoate (**S5**). Compound **S5** (Yield: 65%, yellow solid) was synthesized following the same procedure as for **3a**, using commercially available 4-hydroxy-2-(1-methyl-2,6-dioxopiperidin-3-yl)isoindoline-1,3-dione and **2b**. ^{1}H NMR (600 MHz, Chloroform-*d*) δ 7.66 (dd, *J* = 8.5, 7.3 Hz, 1H), 7.44 (d, *J* = 7.3 Hz, 1H), 7.22 (d, *J* = 8.5 Hz, 1H), 4.96 (dd, *J* = 12.2, 5.2 Hz, 1H), 4.25 – 4.20 (m, 2H), 3.17 (s, 3H), 2.79 – 2.66 (m, 3H), 2.52 (t, *J* = 7.2 Hz, 2H), 2.13 – 2.00 (m, 3H), 1.46 (s, 9H). LC-MS (m/z): positive mode 431.2 $[M + H]^{+}$.

4-((2-(1-methyl-2,6-dioxopiperidin-3-yl)-1,3-dioxoisoindolin-4-yl)oxy)butanoic acid (**S6**). Compound **S6** was prepared with the same synthetic method as **4a**, directly used in the next step without further purification.

3-((1,1-dioxidotetrahydrothiophen-3-yl)amino)-2,2-dimethylpropyl 4-((2-(1-methyl-2,6-dioxopiperidin-3-yl)-1,3-dioxoisoindolin-4-yl)oxy)butanoate. (**S7**). Compound **S7** (Yield: 54%, white solid) was prepared with the same synthetic method as **5a**. ^{1}H NMR (600 MHz, Chloroform-*d*) δ 7.67 (dd, *J* = 8.5, 7.3 Hz, 1H), 7.45 (d, *J* = 7.3 Hz, 1H), 7.22 (d, *J* = 8.5 Hz, 1H), 4.97 (dt, *J* = 12.4, 5.2 Hz, 1H), 4.27 – 4.20 (m, 2H), 3.94 – 3.86 (m, 2H), 3.50 (p, *J* = 6.4 Hz, 1H), 3.31 – 3.22 (m, 2H), 3.19 (s, 3H), 3.06 – 2.91 (m, 2H), 2.84 (dd, *J* = 13.3, 6.2 Hz, 1H), 2.81 – 2.75 (m, 2H), 2.63 (td, *J* = 7.2, 3.3 Hz, 2H), 2.43 – 2.33 (m, 3H), 2.23 – 2.15 (m, 2H), 2.12 – 1.99 (m, 2H), 1.27 – 1.22 (m, 1H), 0.90 (d, *J* = 1.4 Hz, 6H). ^{13}C NMR (151 MHz, Chloroform-*d*) δ 173.14, 171.38, 169.05, 167.28, 165.92, 156.39, 136.66, 134.00, 119.02, 117.45, 116.10, 70.31, 68.08, 57.15, 55.81, 54.84, 50.68,

49.99, 35.06, 31.99, 30.34, 29.66, 27.35, 24.39, 23.06, 23.01, 22.06. LC-MS (m/z): positive mode 578.2 $[M + H]^+$.
