# Supplementary material for: Systematic Optimization of Proteolysis-Targeting Chimeras for PIN1 Enables Selective Degradation and Antitumor Activity In Vivo
Source: Pharmaceutics. 2026 Feb 26;18(3):288. doi: 10.3390/pharmaceutics18030288 (PMC13029591; doi:10.3390/pharmaceutics18030288)
Supplement: Supplementary file 1 [file pharmaceutics-18-00288-s001.zip › pharmaceutics-Supplementary File S3-HPLC traces.pdf]

pharmaceutics

Supplementary Materials: Systematic Optimization of Proteolysis-Targeting Chimeras for PIN1 Enables Selective Degradation and Antitumor Activity In Vivo

Yuying Ma, Yang Teng, Jinjin Liu, Yuke Deng, Lingbo Xu, Ruichen Gao, Tingyu Peng, Wei Li, Yue Wei, Linfeng Li, and Zufeng Guo

HPLC traces

PC1

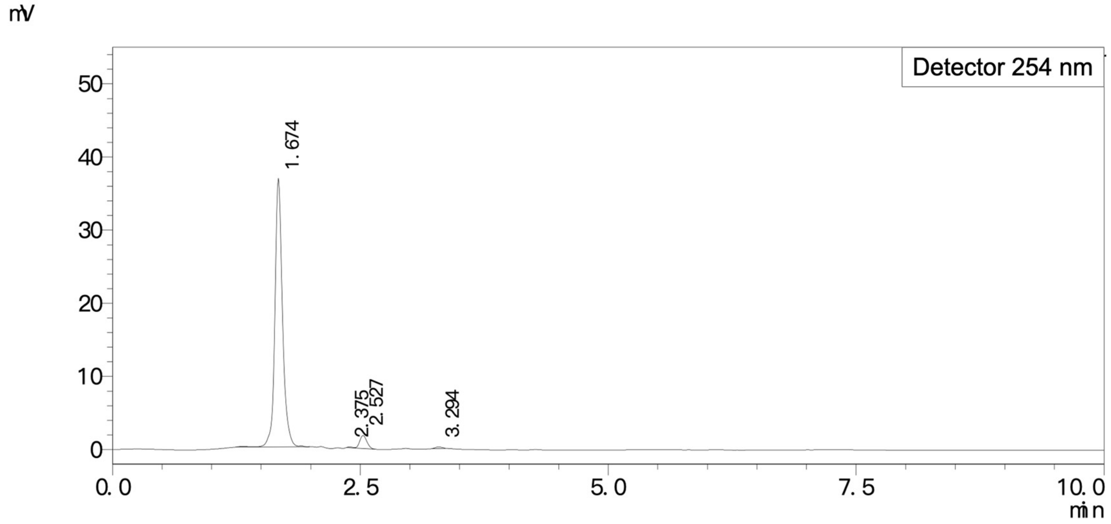

PC2

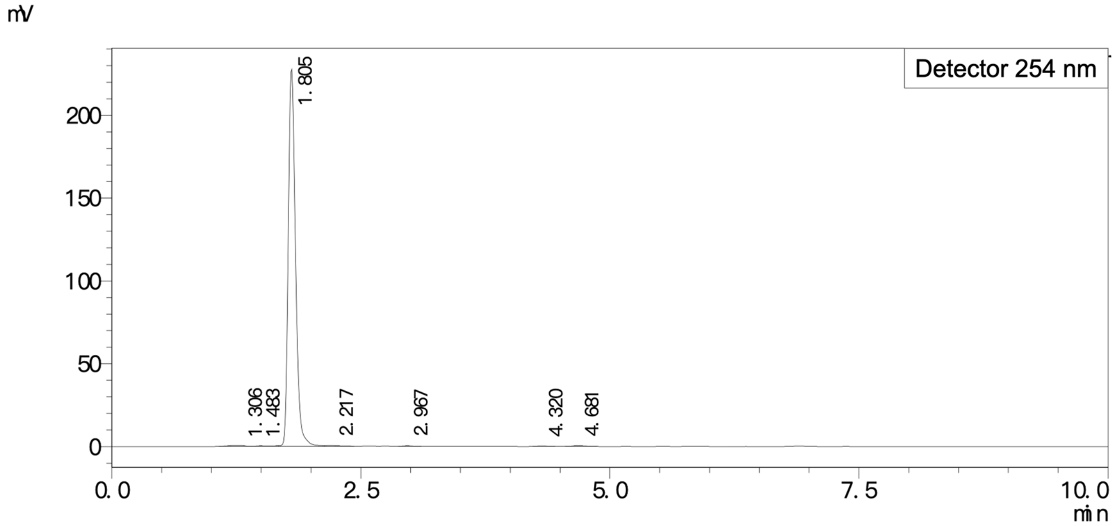

PC3

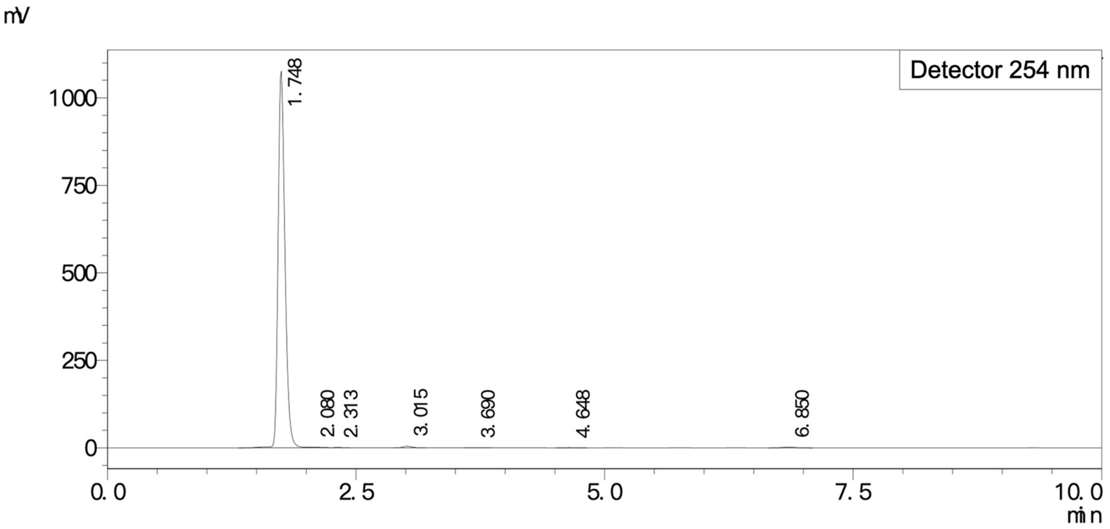
mV
Detector 254 nm
1. 748
2. 080
2. 313
3. 015
3. 690
4. 648
6. 850
1000
750
500
250
0
0. 0
2. 5
5. 0
7. 5
10. 0
min

PC4

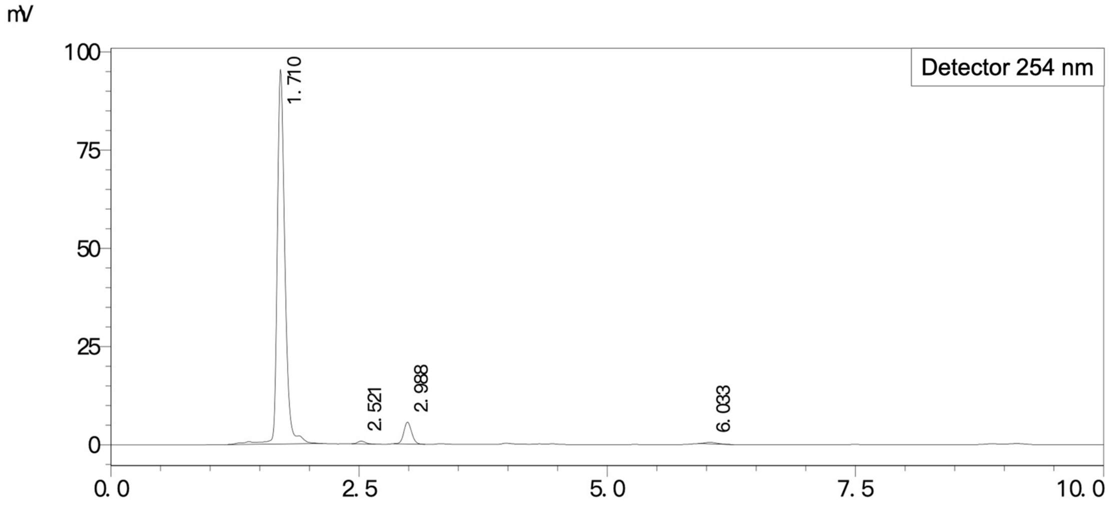
mV
Detector 254 nm
1. 710
2. 521
2. 988
6. 033
100
75
50
25
0
0. 0
2. 5
5. 0
7. 5
10. 0

PC5

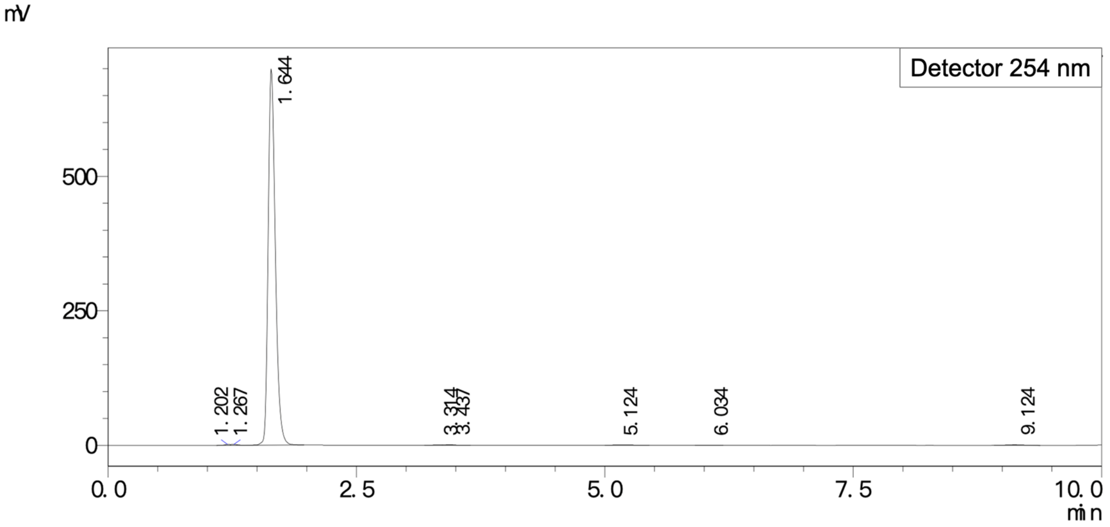
mV
Detector 254 nm
1. 644
1. 202
1. 267
3. 314
3. 437
5. 124
6. 034
9. 124
500
250
0
0. 0
2. 5
5. 0
7. 5
10. 0
min

PC6

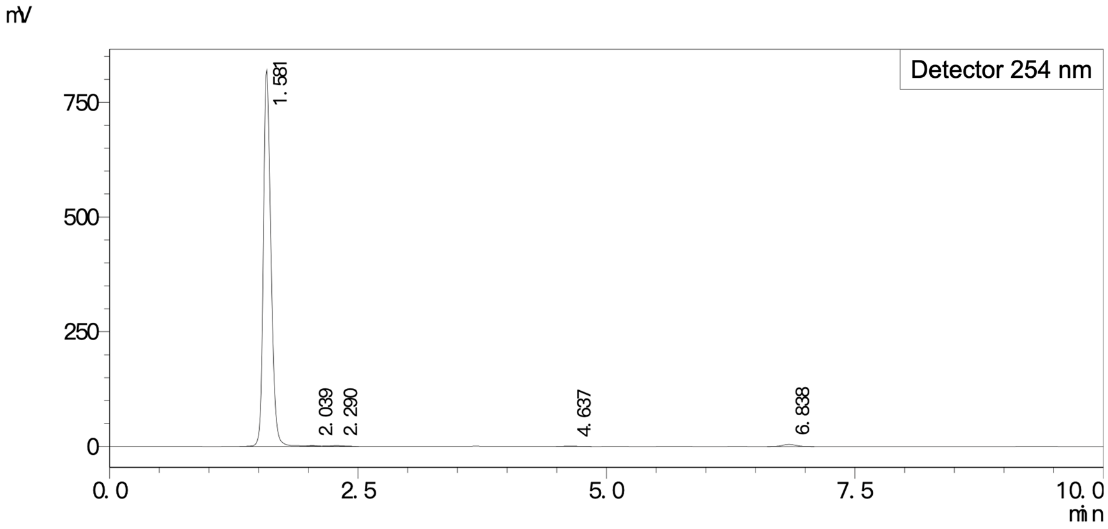
mV
Detector 254 nm
1.581
2.039
2.290
4.637
6.838
750
500
250
0
0.0
2.5
5.0
7.5
10.0
min

PC7

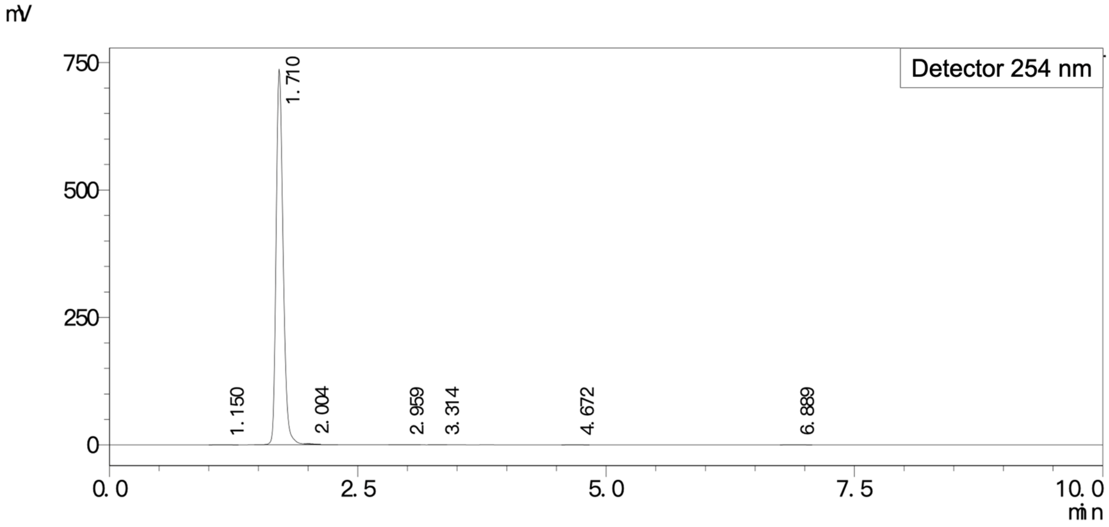
mV
Detector 254 nm
1.150
1.710
2.004
2.959
3.314
4.672
6.889
750
500
250
0
0.0
2.5
5.0
7.5
10.0
min

PC8

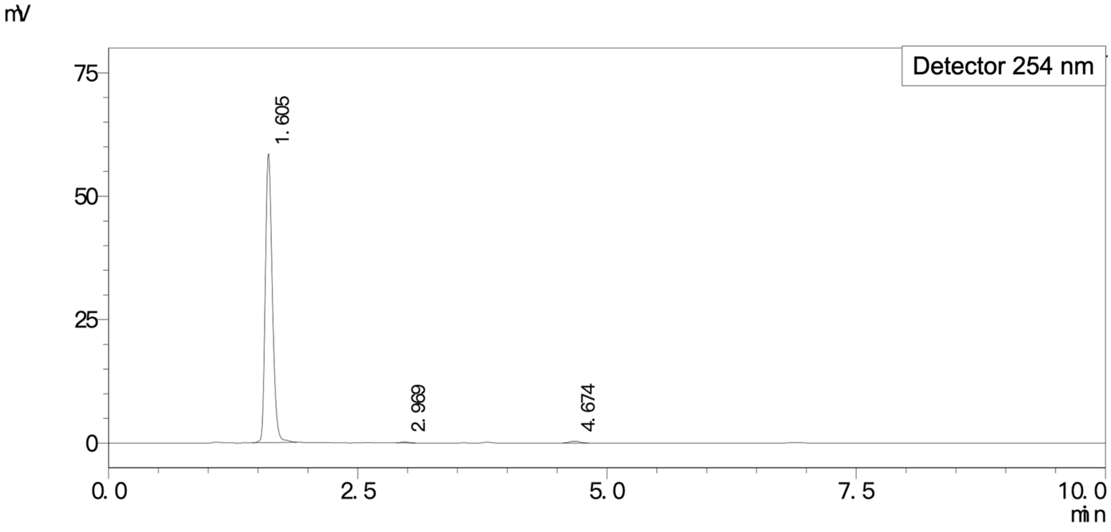
mV
Detector 254 nm
1.605
2.969
4.674
75
50
25
0
0.0
2.5
5.0
7.5
10.0
min

PC9

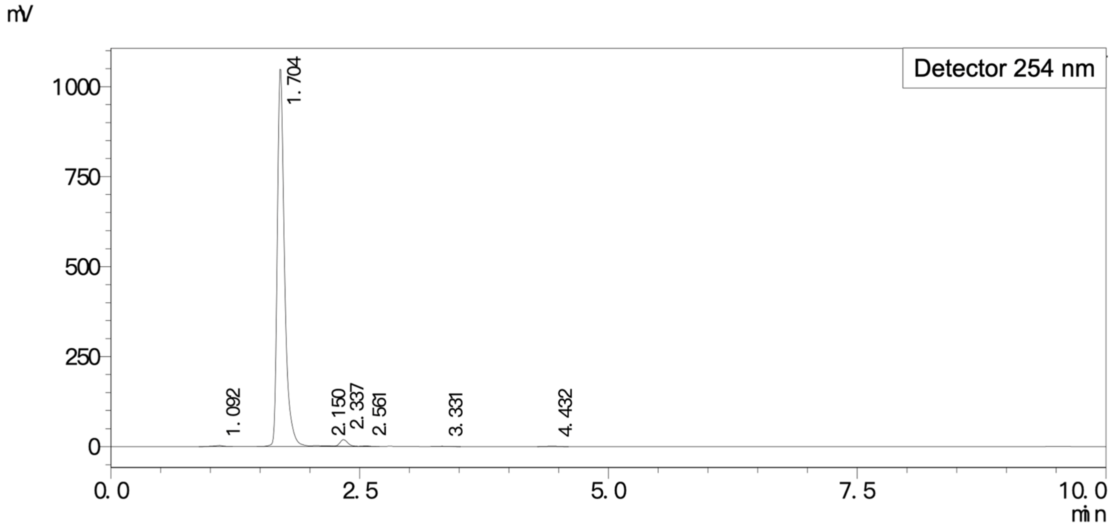

PC10

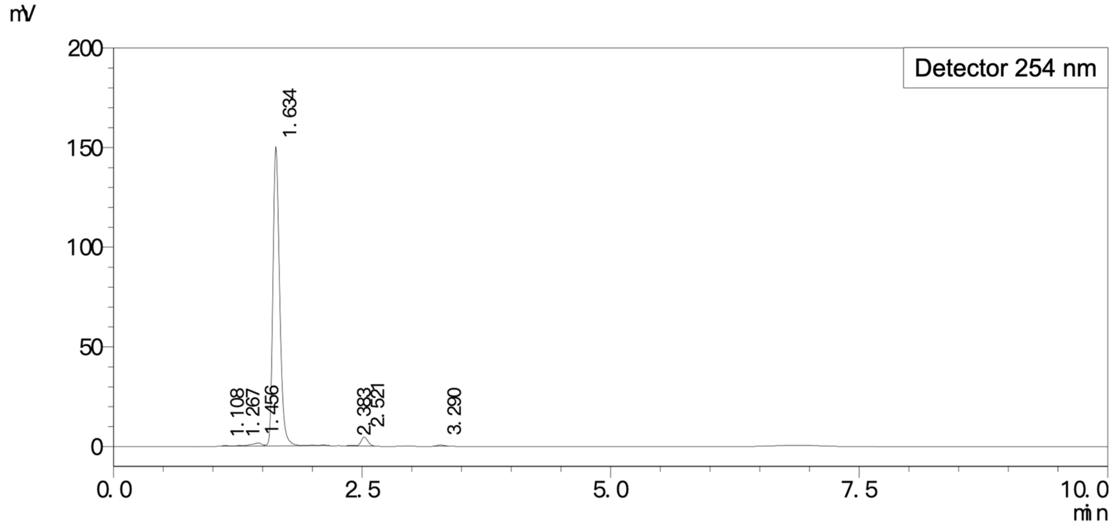

PC11

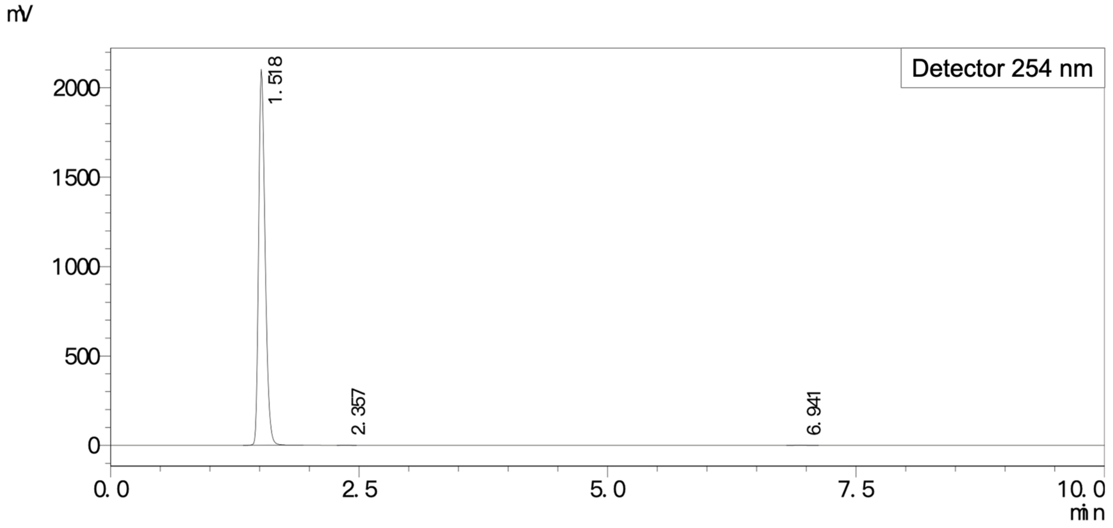

PC12

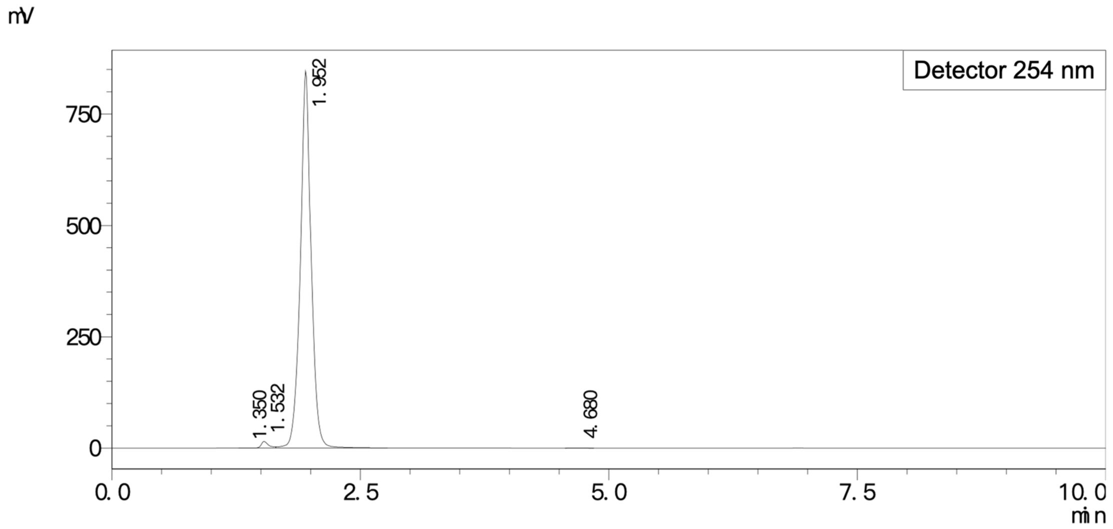

PC13

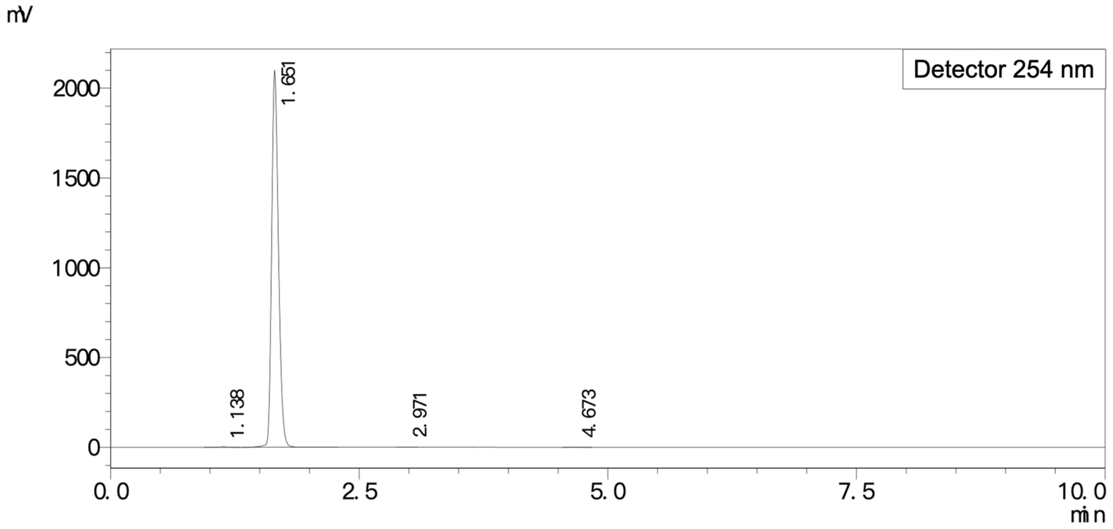

PC14

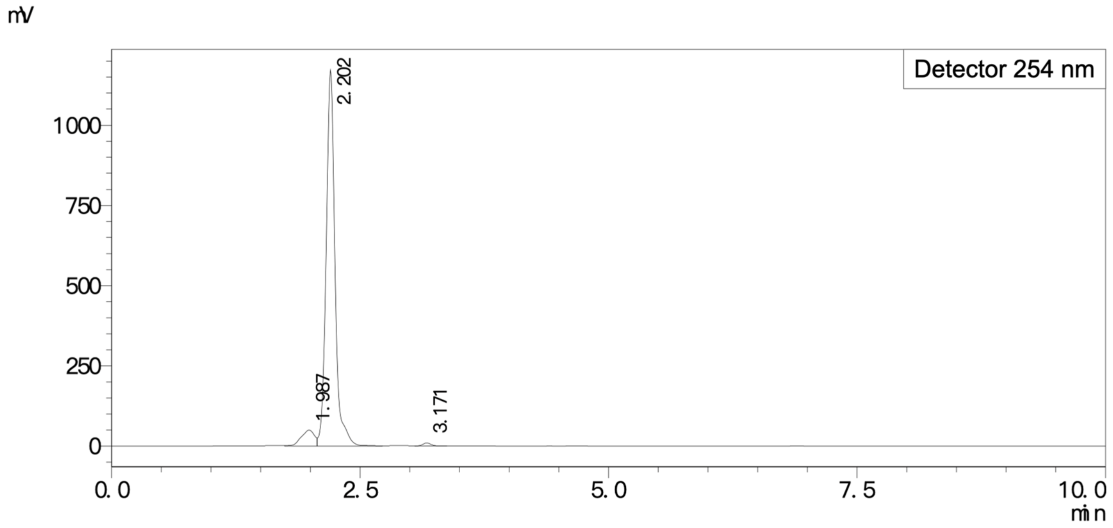

PV1

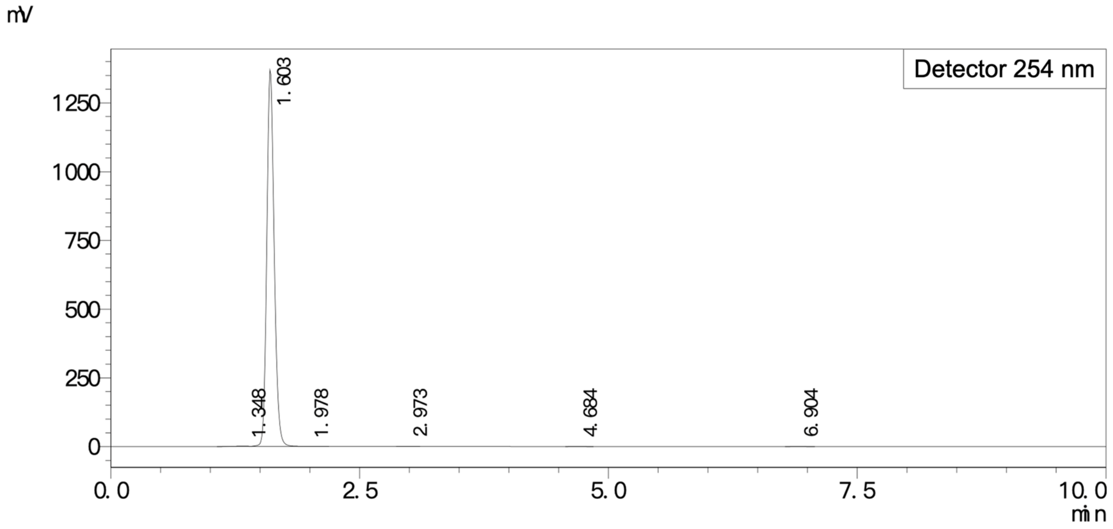
mV
Detector 254 nm
1250
1000
750
500
250
0
1.603
1.348
1.978
2.973
4.684
6.904
0.0
2.5
5.0
7.5
10.0
min

PV2

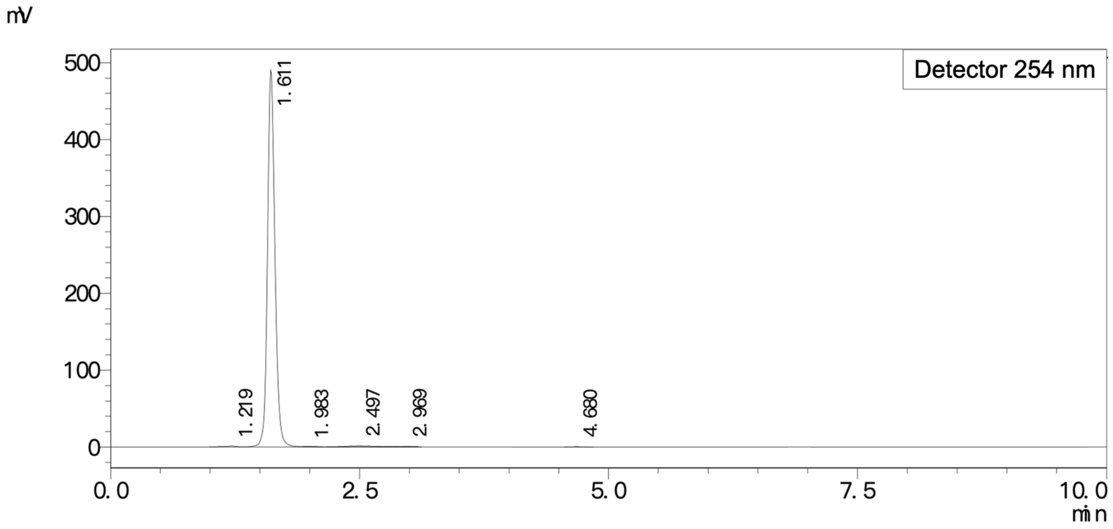
mV
Detector 254 nm
500
400
300
200
100
0
1.611
1.219
1.983
2.497
2.969
4.680
0.0
2.5
5.0
7.5
10.0
min

PV3

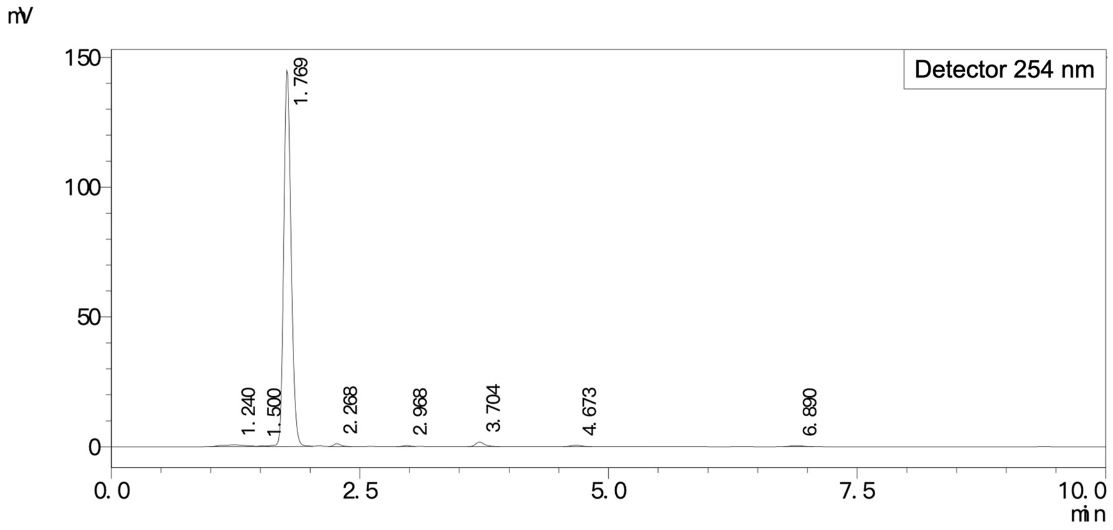
mV
Detector 254 nm
150
100
50
0
1.769
1.240
1.500
2.268
2.968
3.704
4.673
6.890
0.0
2.5
5.0
7.5
10.0
min

PV4

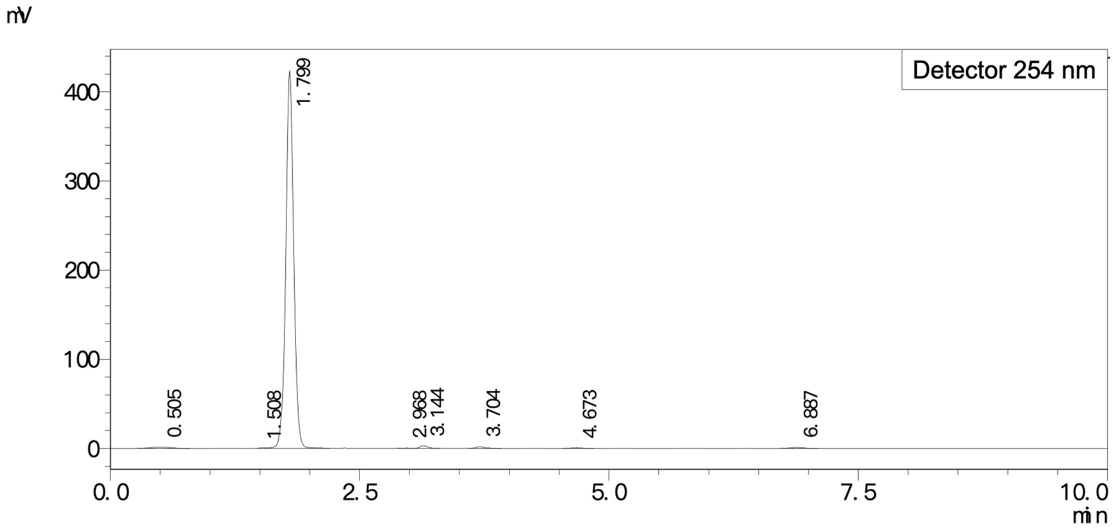
mV
Detector 254 nm
400
300
200
100
0
1.799
0.505
1.508
2.968
3.144
3.704
4.673
6.887
0.0
2.5
5.0
7.5
10.0
min

PV5

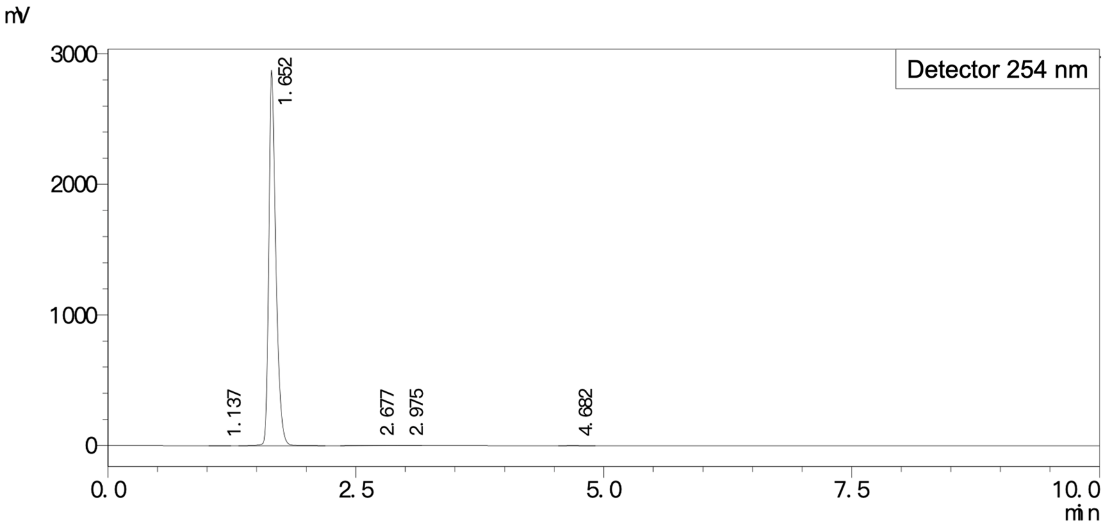
mV
Detector 254 nm
3000
2000
1000
0
1.652
1.137
2.677
2.975
4.682
0.0
2.5
5.0
7.5
10.0
min

PV6

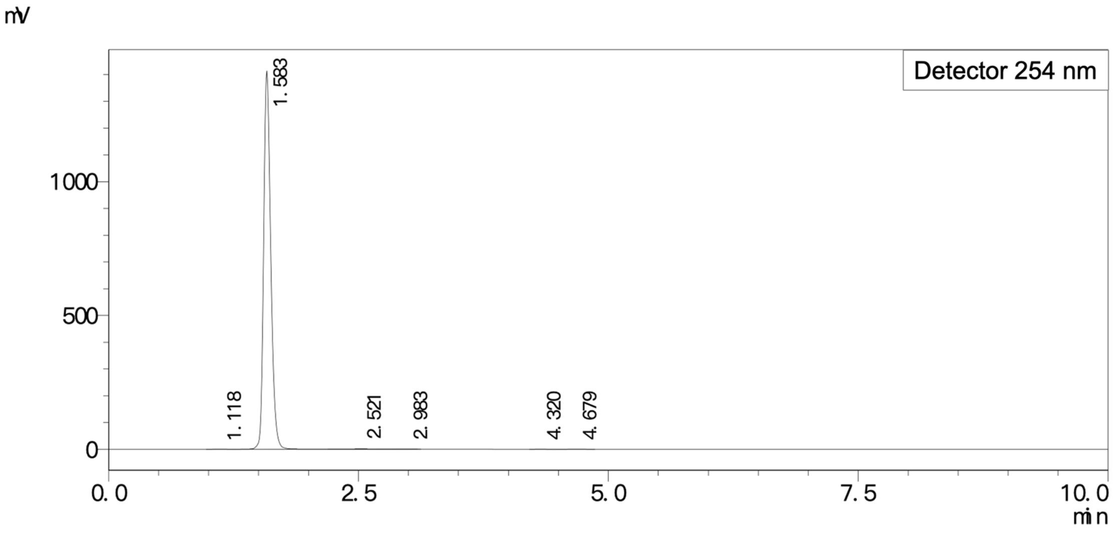
mV
Detector 254 nm
1000
500
0
1.583
1.118
2.521
2.983
4.320
4.679
0.0
2.5
5.0
7.5
10.0
min

PV7

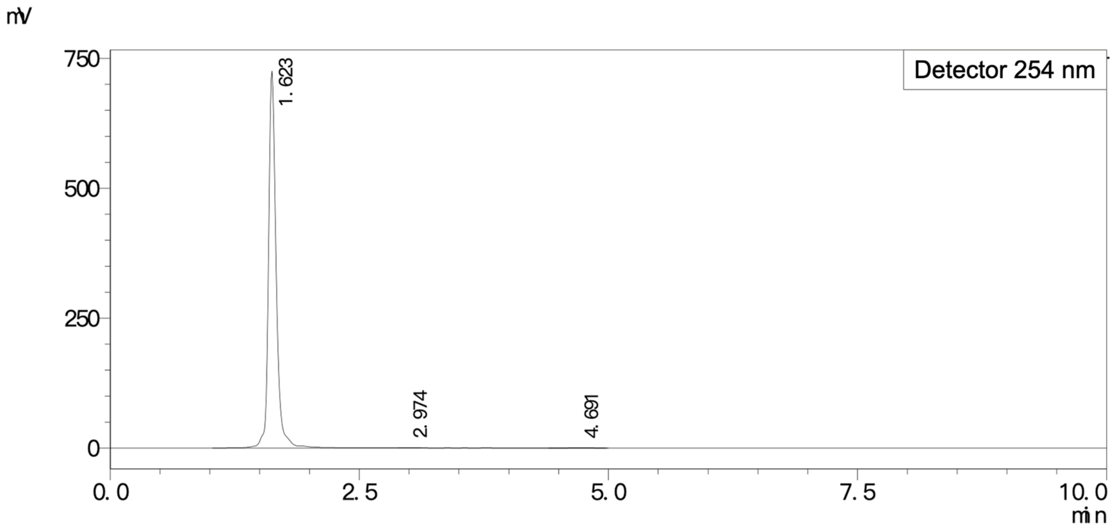
mV
Detector 254 nm
1. 623
2. 974
4. 691
750
500
250
0
0. 0
2. 5
5. 0
7. 5
10. 0
min

PV8

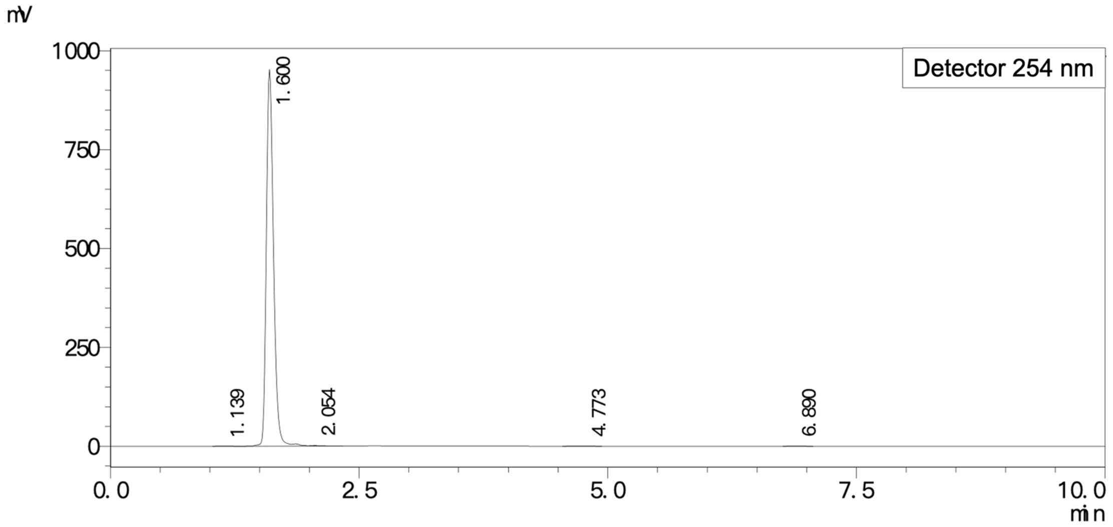
mV
Detector 254 nm
1. 600
1. 139
2. 054
4. 773
6. 890
1000
750
500
250
0
0. 0
2. 5
5. 0
7. 5
10. 0
min

PV9

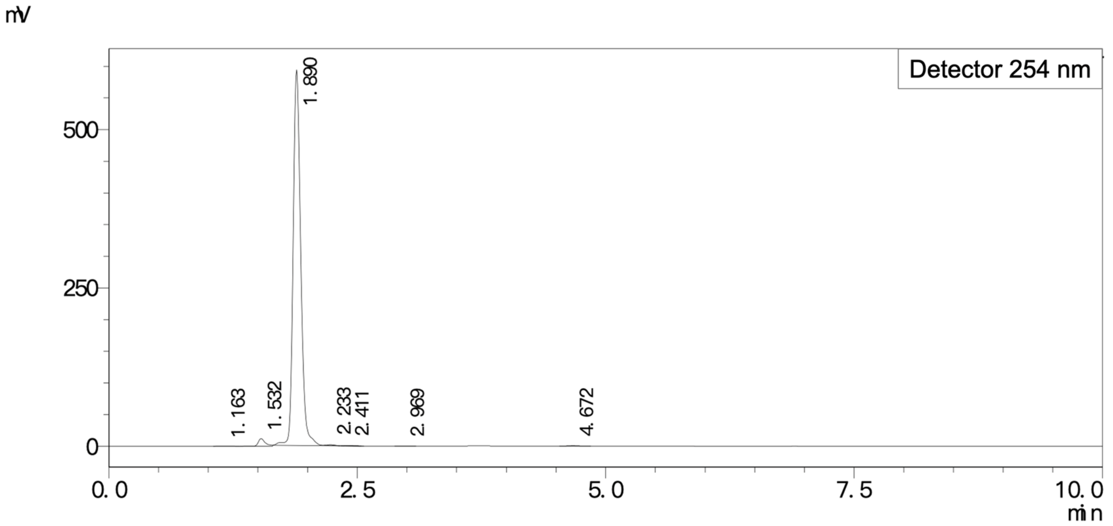
mV
Detector 254 nm
1. 890
1. 163
1. 532
2. 233
2. 411
2. 969
4. 672
500
250
0
0. 0
2. 5
5. 0
7. 5
10. 0
min

PV10

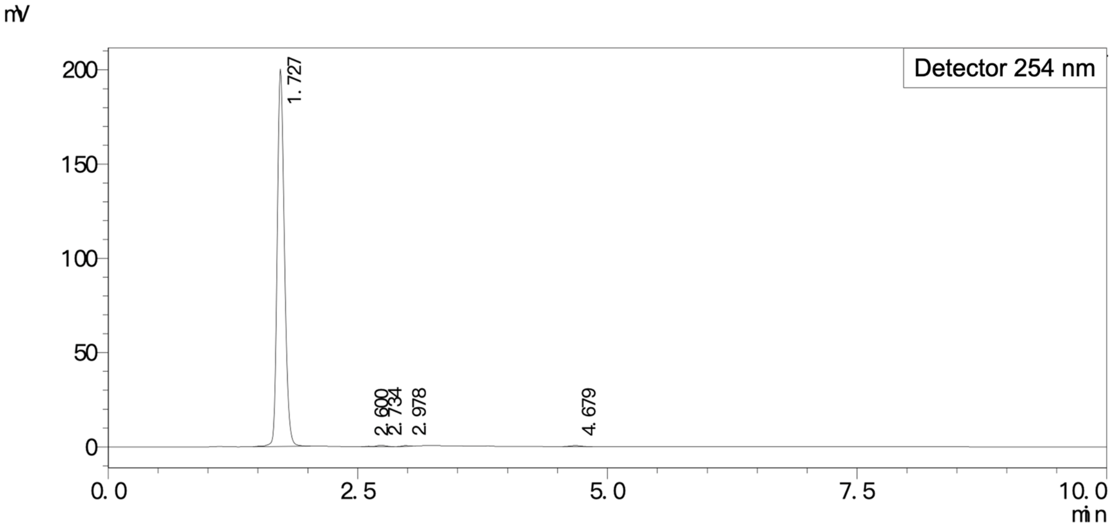

PV11

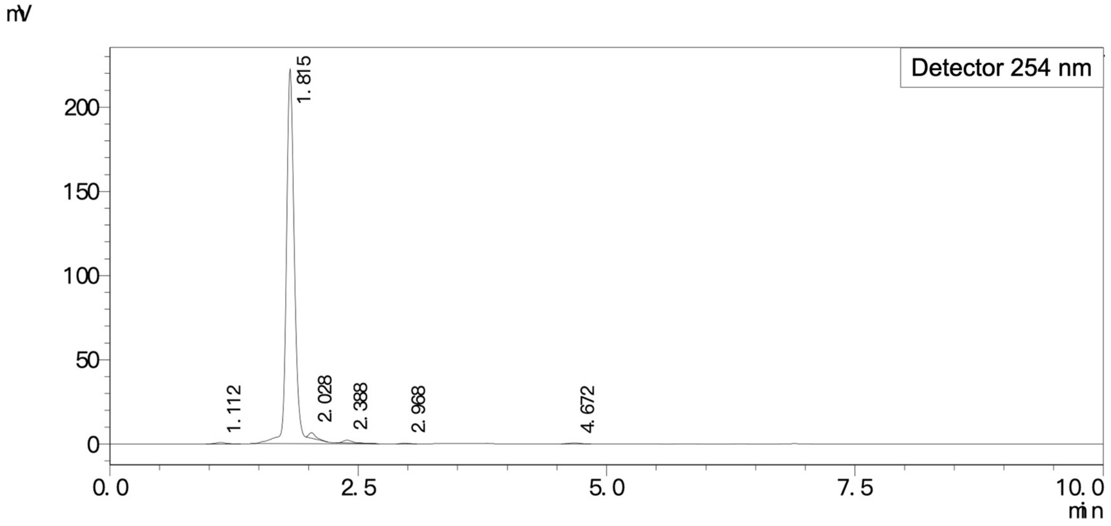

PV12

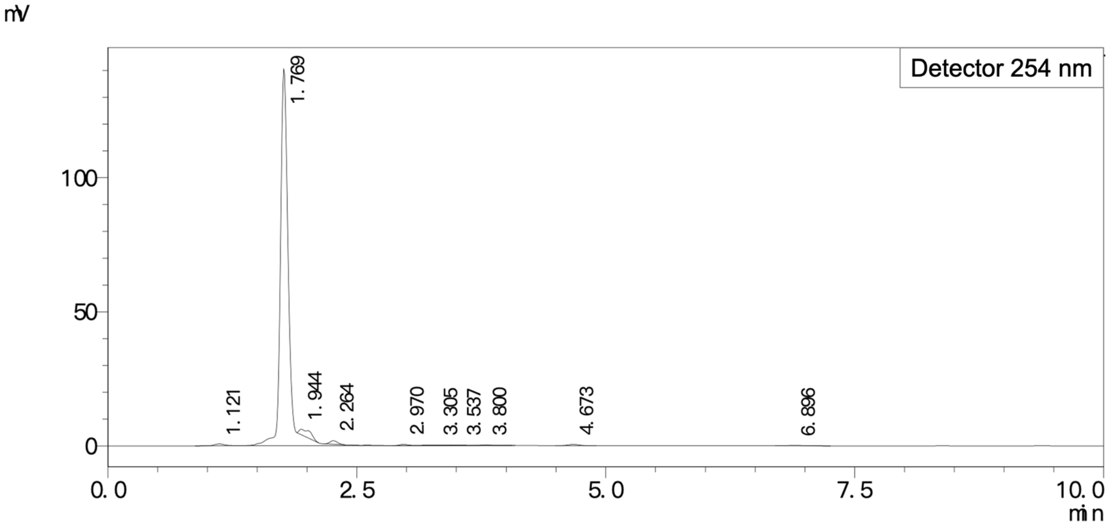

PV13

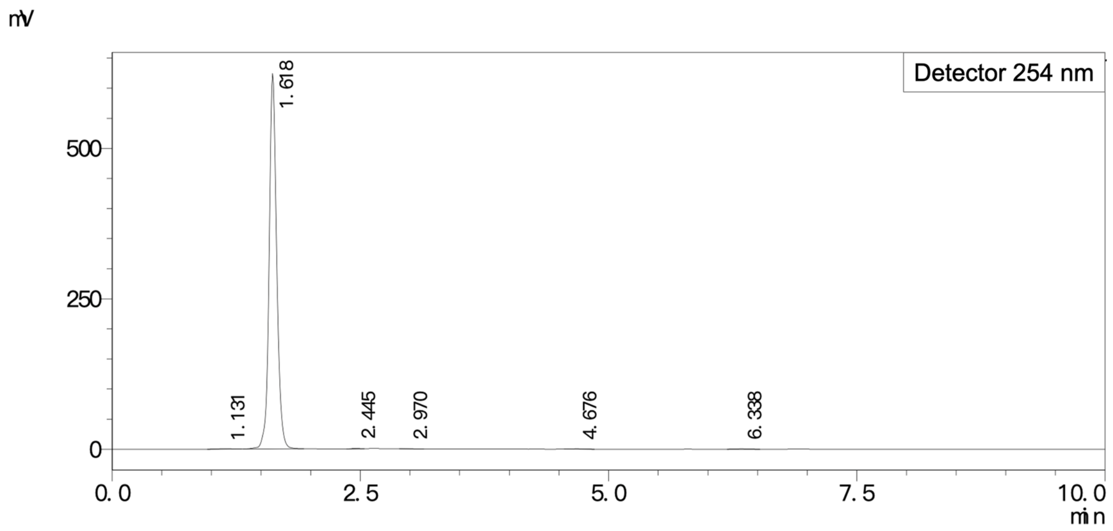

PV14

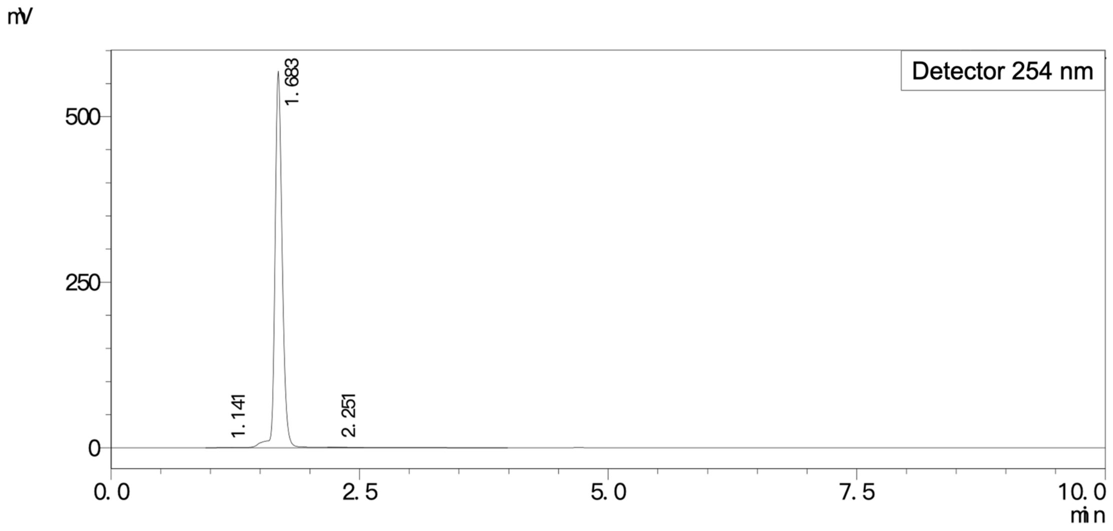

PV15

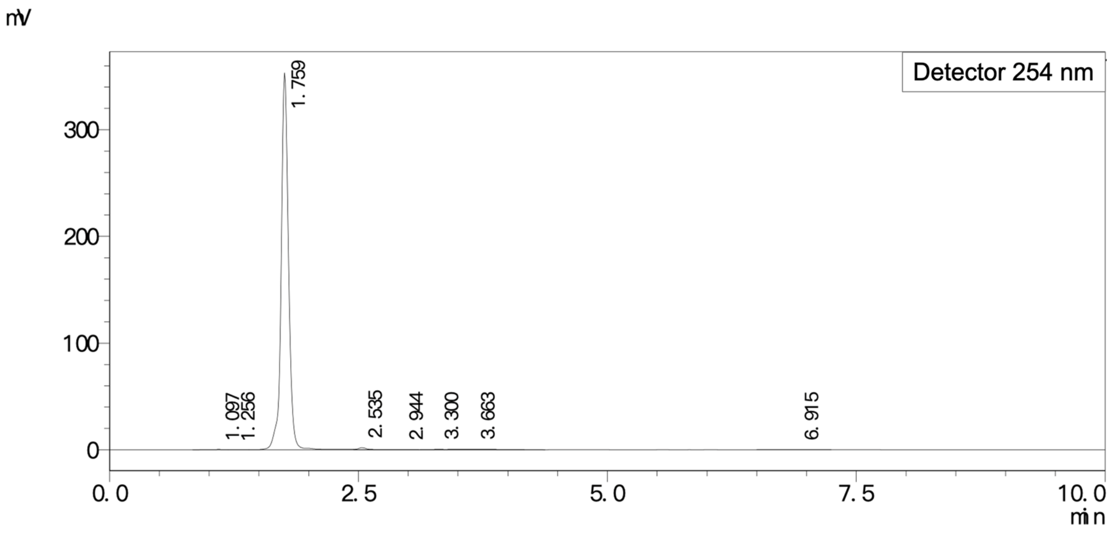

PV16

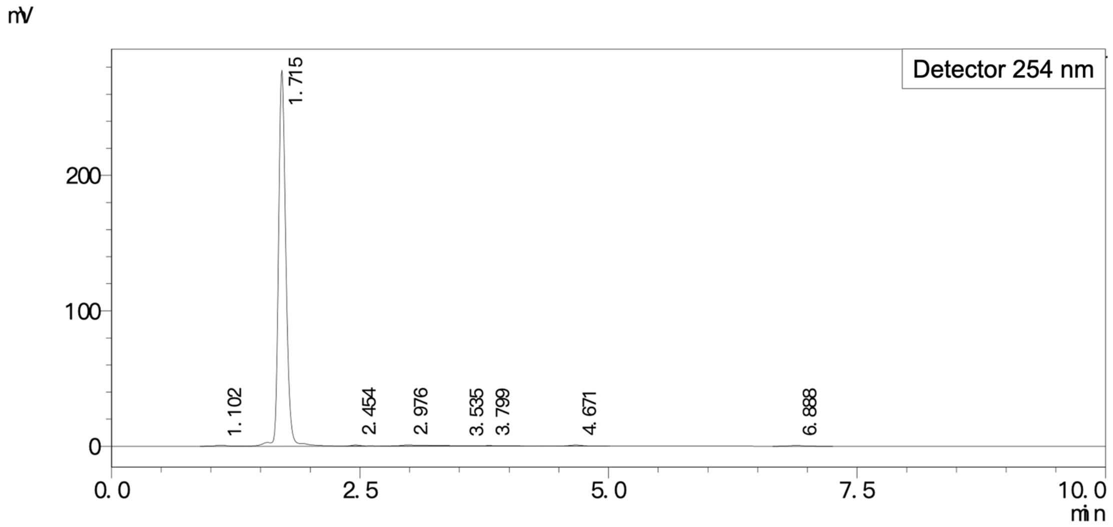

PV17

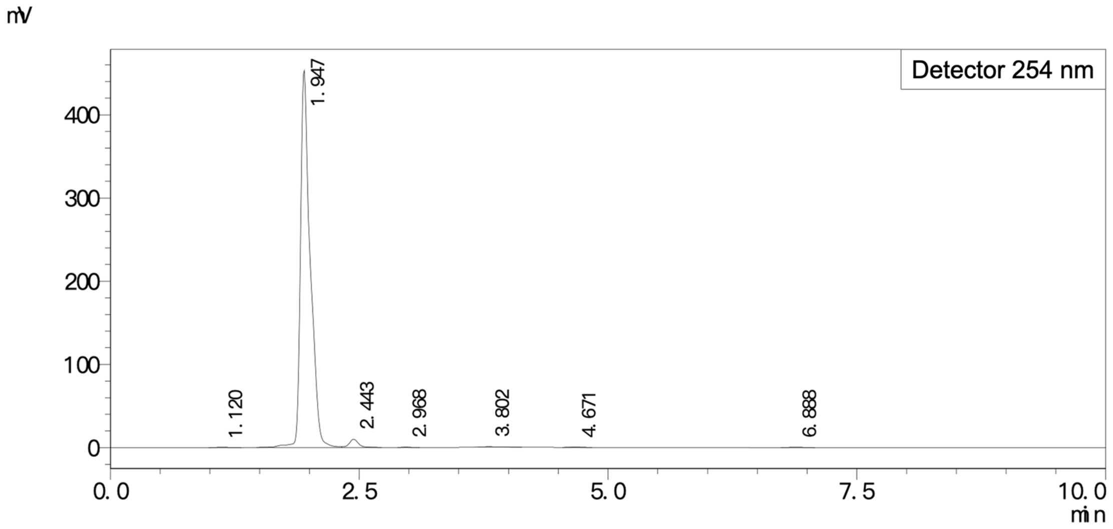

PV18

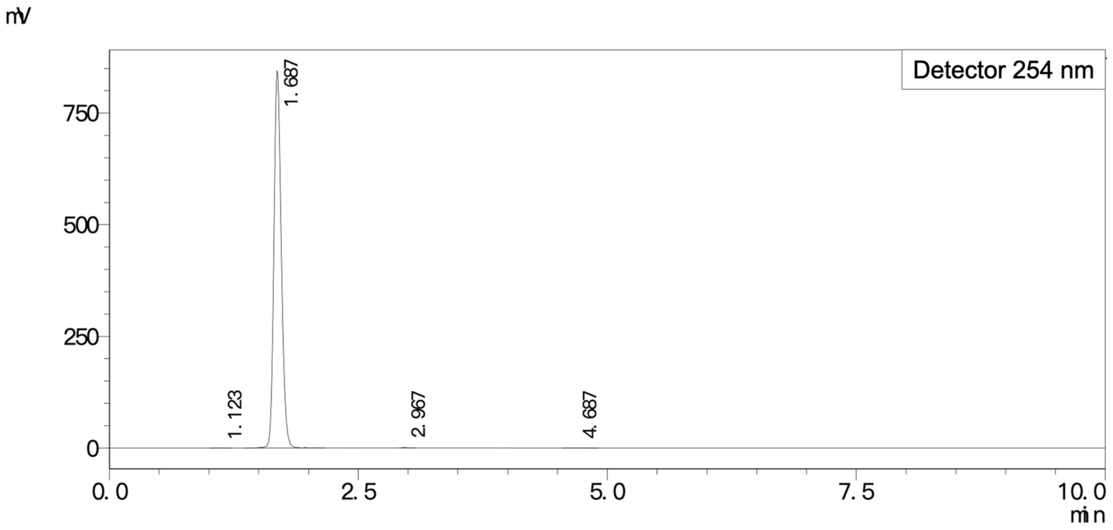

PV19

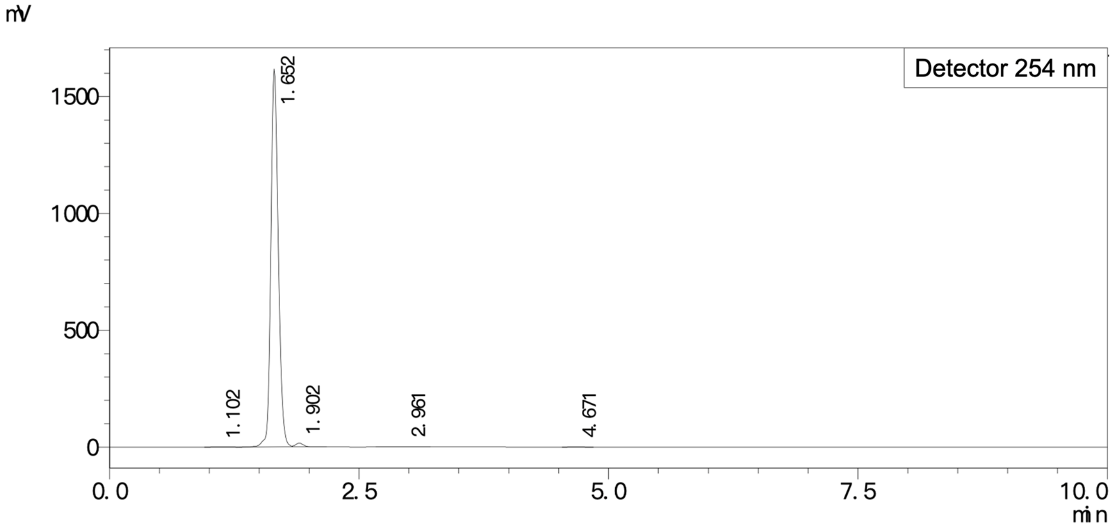

PV20

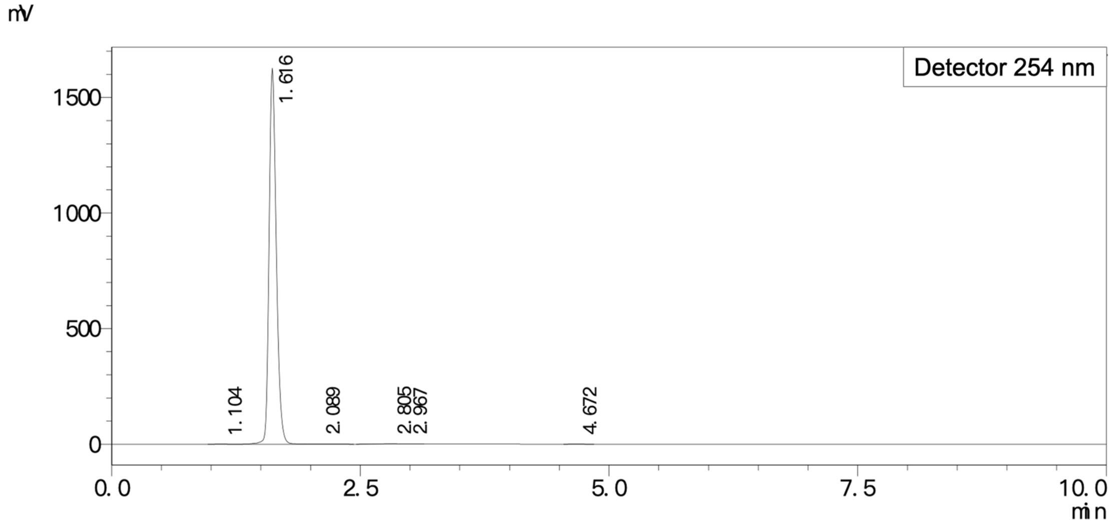

PV21

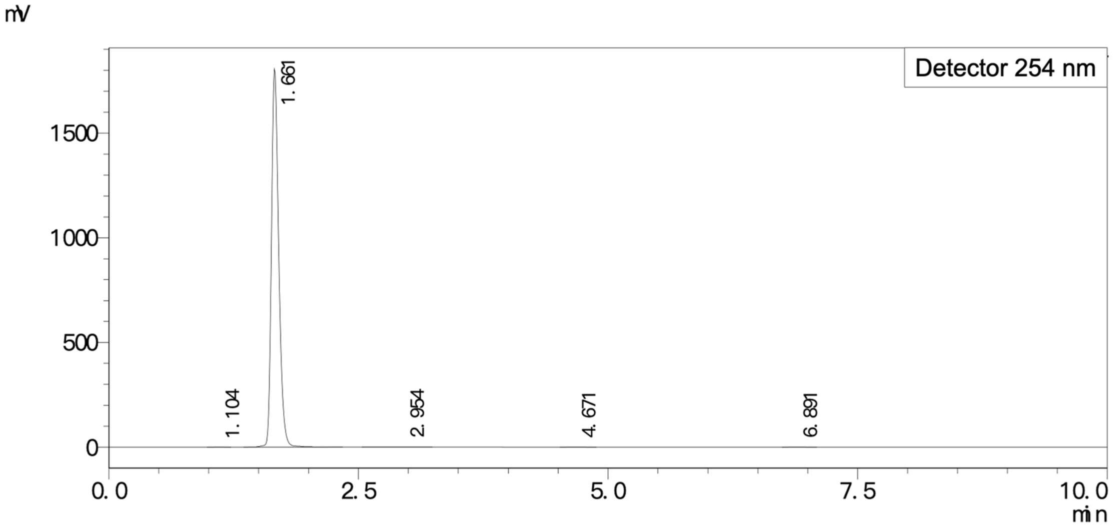

PC2-Neg

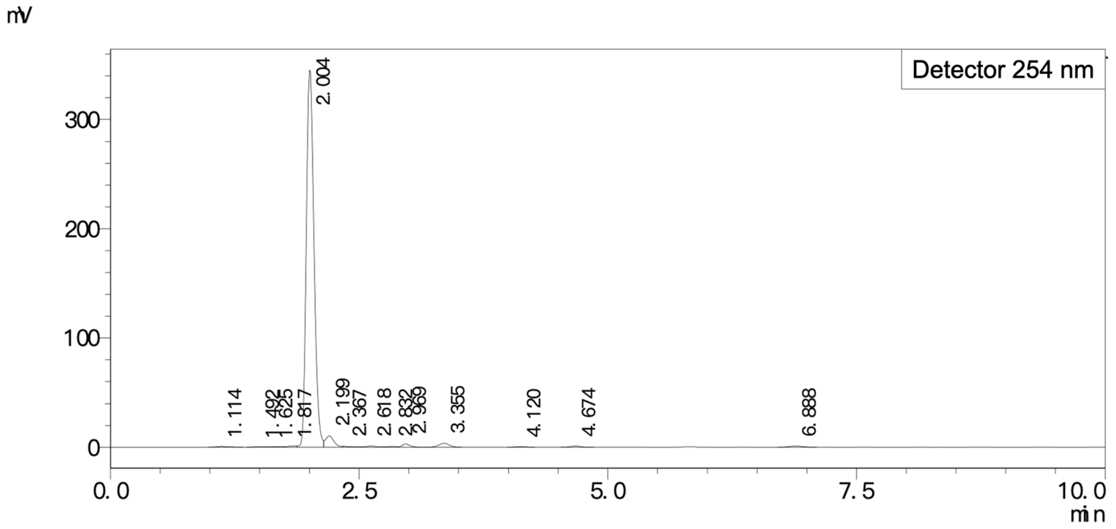
mV
Detector 254 nm
300
200
100
0
2.004
1.114
1.492
1.625
1.817
2.199
2.367
2.618
2.832
2.969
3.355
4.120
4.674
6.888
0.0
2.5
5.0
7.5
10.0
min
