# Supplementary material for: Systematic Optimization of Proteolysis-Targeting Chimeras for PIN1 Enables Selective Degradation and Antitumor Activity In Vivo
Source: Pharmaceutics. 2026 Feb 26;18(3):288. doi: 10.3390/pharmaceutics18030288 (PMC13029591; doi:10.3390/pharmaceutics18030288)
Supplement: Supplementary file 1 [file pharmaceutics-18-00288-s001.zip › pharmaceutics-Supplementary File S4-HRMS data.pdf]

pharmaceutics

MDPI

Supplementary Materials: Systematic Optimization of Proteolysis-Targeting Chimeras for PIN1 Enables Selective Degradation and Antitumor Activity In Vivo

Yuying Ma, Yang Teng, Jinjin Liu, Yuke Deng, Lingbo Xu, Ruichen Gao, Tingyu Peng, Wei Li, Yue Wei, Linfeng Li, and Zufeng Guo

HRMS spectra of final compounds

PC1

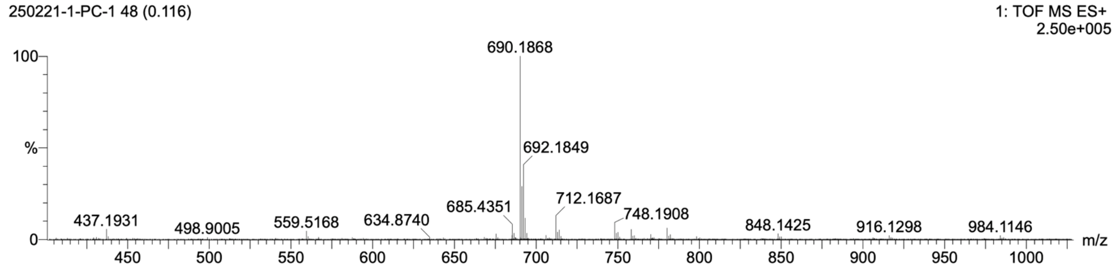

	Mass	Calc. Mass	mDa	PPM	DBE	i-FIT	Norm	Conf(%)	Formula
Minimum:					-1.5				
Maximum:			5.0	5.0	50.0				
	690.1868	690.1864	0.4	0.6	12.5	474.0	n/a	n/a	C30 H38 N3 O10 Na S Cl

PC2

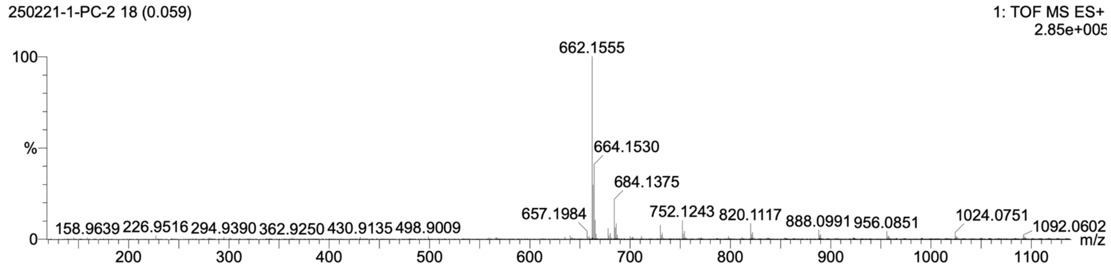

	Mass	Calc. Mass	mDa	PPM	DBE	i-FIT	Norm	Conf(%)	Formula
Minimum:					-1.5				
Maximum:			5.0	5.0	50.0				
	662.1555	662.1551	0.4	0.6	12.5	485.5	n/a	n/a	C28 H34 N3 O10 Na S Cl

PC3

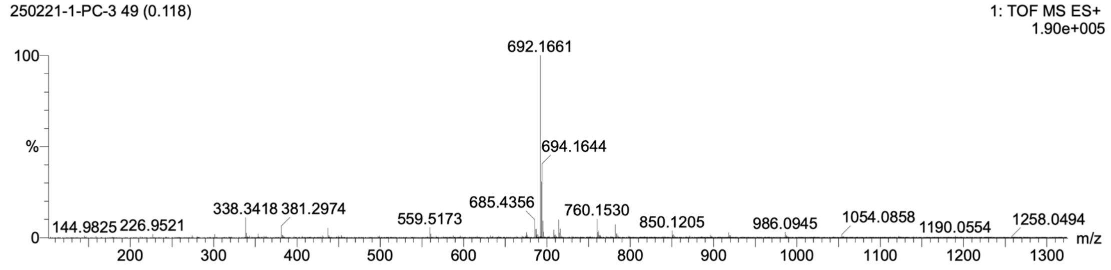

	Mass	Calc. Mass	mDa	PPM	DBE	i-FIT	Norm	Conf(%)	Formula
Minimum:					-1.5				
Maximum:			5.0	5.0	50.0				
	692.1661	692.1657	0.4	0.6	12.5	463.6	n/a	n/a	C29 H36 N3 O11 Na S Cl

PC4

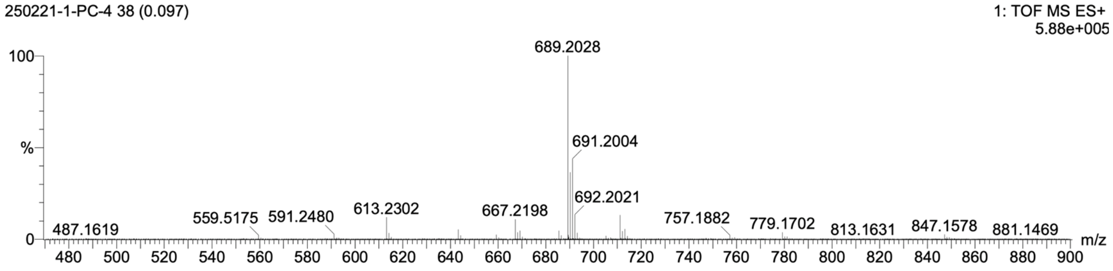

Minimum:				-1.5				
Maximum:		5.0	5.0	50.0				

Mass	Calc. Mass	mDa	PPM	DBE	i-FIT	Norm	Conf(%)	Formula
689.2028	689.2024	0.4	0.6	12.5	542.8	n/a	n/a	C30 H39 N4 O9 Na S Cl

PC5

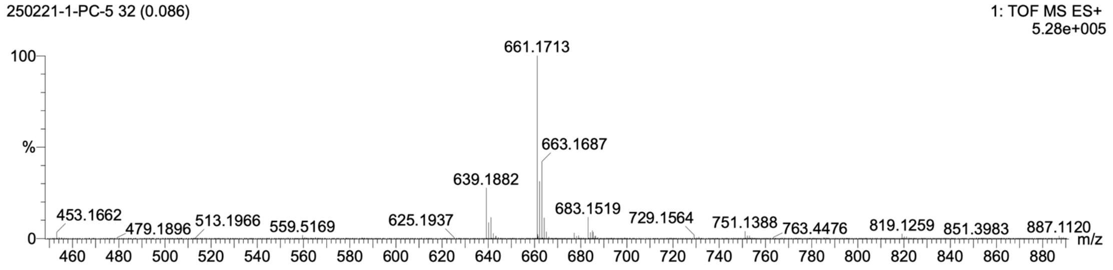

Minimum:				-1.5				
Maximum:		5.0	5.0	50.0				

Mass	Calc. Mass	mDa	PPM	DBE	i-FIT	Norm	Conf(%)	Formula
661.1713	661.1711	0.2	0.3	12.5	565.6	n/a	n/a	C28 H35 N4 O9 Na S Cl

PC6

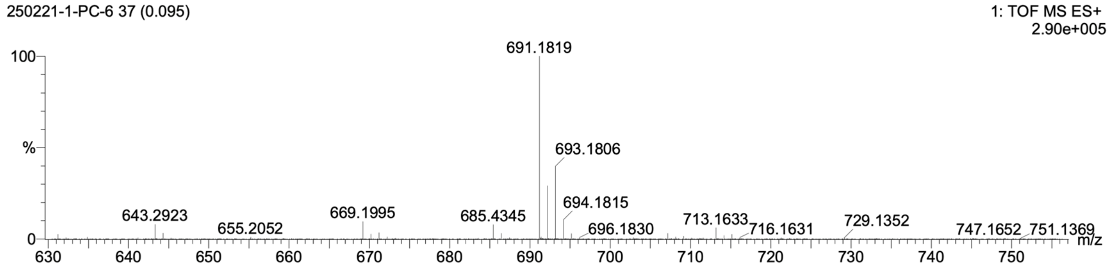

Minimum:				-1.5				
Maximum:		5.0	5.0	50.0				

Mass	Calc. Mass	mDa	PPM	DBE	i-FIT	Norm	Conf(%)	Formula
691.1819	691.1817	0.2	0.3	12.5	534.9	n/a	n/a	C29 H37 N4 O10 Na S Cl

PC7

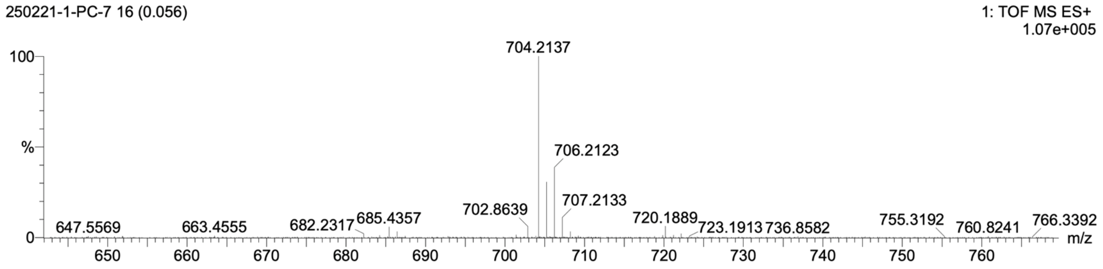

Minimum:				-1.5				
Maximum:		5.0	5.0	50.0				

Mass	Calc. Mass	mDa	PPM	DBE	i-FIT	Norm	Conf(%)	Formula
704.2137	704.2133	0.4	0.6	12.5	377.1	n/a	n/a	C30 H40 N5 O9 Na S Cl

PC8

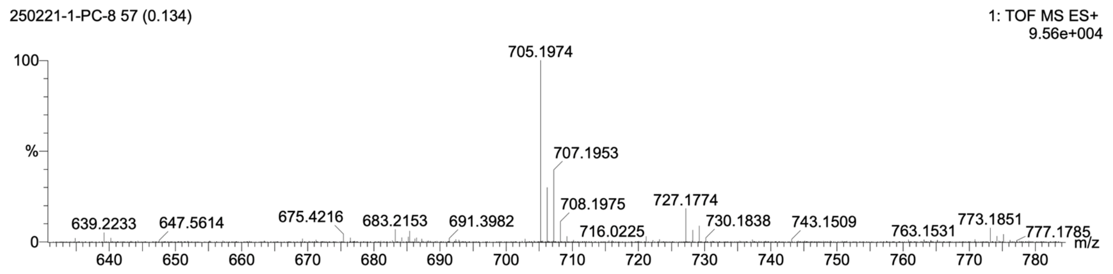

Minimum:				-1.5				
Maximum:		5.0	5.0	50.0				

Mass	Calc. Mass	mDa	PPM	DBE	i-FIT	Norm	Conf(%)	Formula
705.1974	705.1973	0.1	0.1	12.5	380.9	n/a	n/a	C30 H39 N4 O10 Na S Cl

PC9

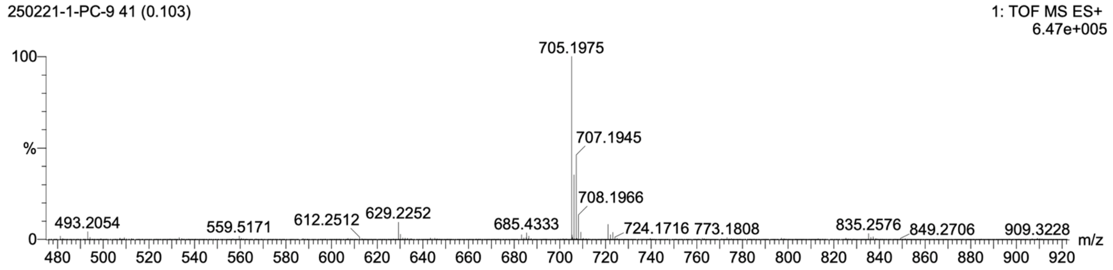

Minimum:				-1.5				
Maximum:		5.0	5.0	50.0				

Mass	Calc. Mass	mDa	PPM	DBE	i-FIT	Norm	Conf(%)	Formula
705.1975	705.1973	0.2	0.3	12.5	546.6	n/a	n/a	C30 H39 N4 O10 Na S Cl

PC10

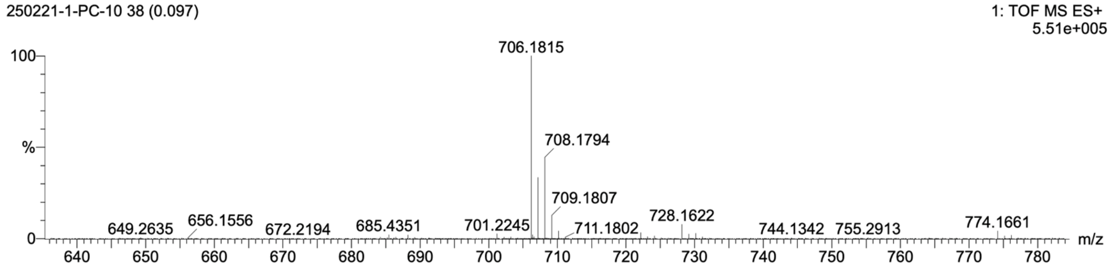

Minimum:				-1.5				
Maximum:		5.0	5.0	50.0				

Mass	Calc. Mass	mDa	PPM	DBE	i-FIT	Norm	Conf(%)	Formula
706.1815	706.1813	0.2	0.3	12.5	574.4	n/a	n/a	C30 H38 N3 O11 Na S Cl

PC11

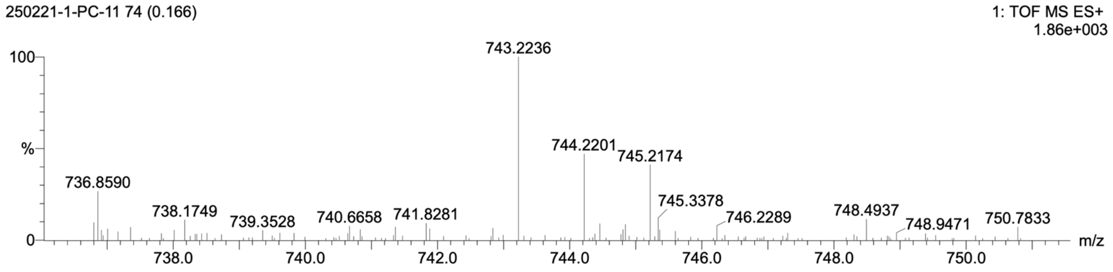

Minimum:				-1.5				
Maximum:		5.0	5.0	50.0				

Mass	Calc. Mass	mDa	PPM	DBE	i-FIT	Norm	Conf(%)	Formula
743.2236	743.2242	-0.6	-0.8	14.5	186.9	n/a	n/a	C32 H41 N6 O9 Na S Cl

PC12

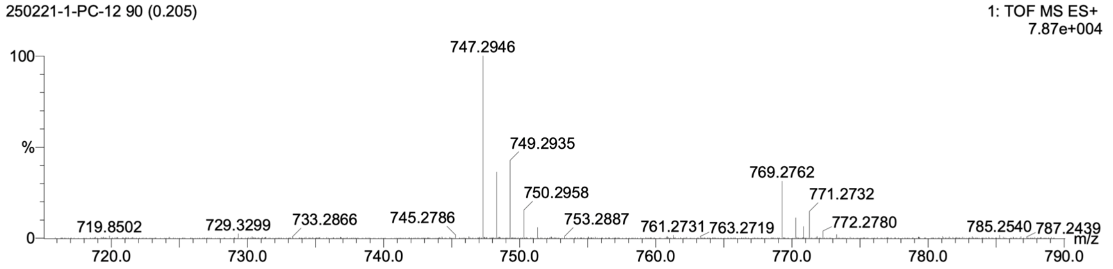

	Mass	Calc. Mass	mDa	PPM	DBE	i-FIT	Norm	Conf(%)	Formula
Minimum:					-1.5				
Maximum:			5.0	5.0	50.0				
	747.2946	747.2943	0.3	0.4	14.5	385.4	n/a	n/a	C35 H48 N6 O8 S Cl

PC13

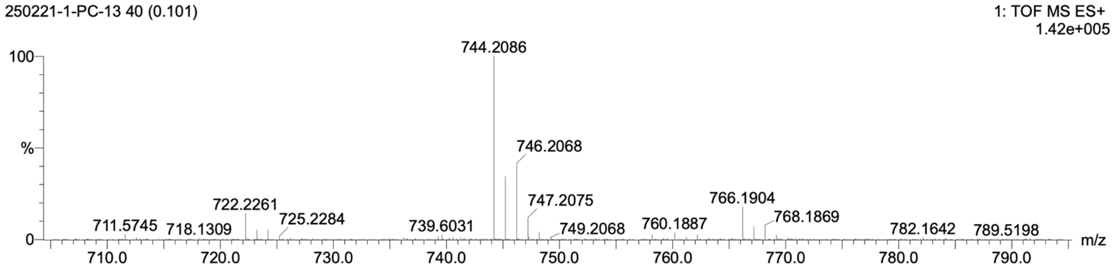

	Mass	Calc. Mass	mDa	PPM	DBE	i-FIT	Norm	Conf(%)	Formula
Minimum:					-1.5				
Maximum:			5.0	5.0	50.0				
	744.2086	744.2082	0.4	0.5	14.5	380.0	n/a	n/a	C32 H40 N5 O10 S Cl Na

PC14

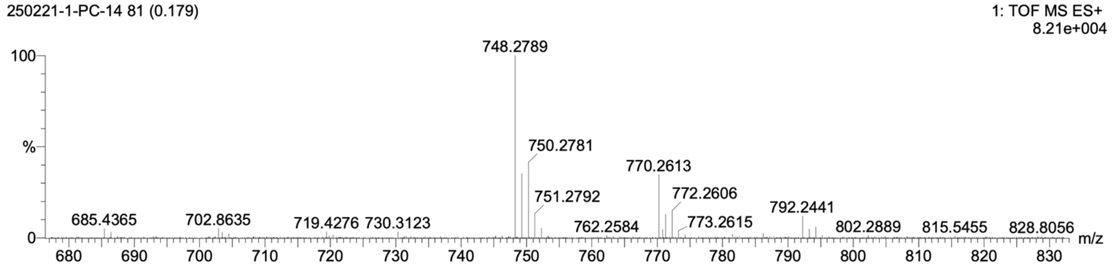

	Mass	Calc. Mass	mDa	PPM	DBE	i-FIT	Norm	Conf(%)	Formula
Minimum:					-1.5				
Maximum:			5.0	5.0	50.0				
	748.2789	748.2783	0.6	0.8	14.5	292.2	n/a	n/a	C35 H47 N5 O9 S Cl

PC2-Neg

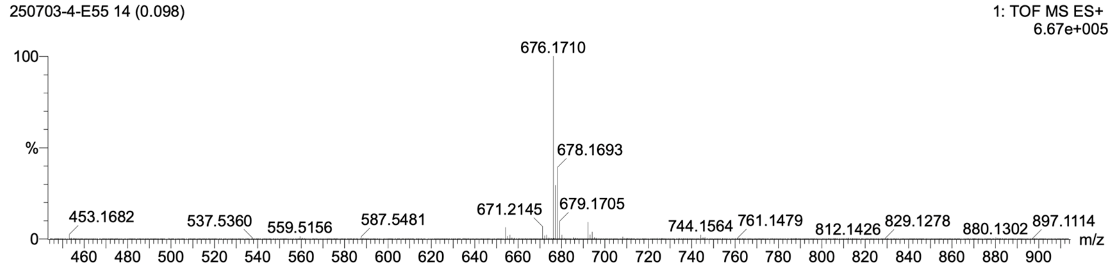

	Mass	Calc. Mass	mDa	PPM	DBE	i-FIT	Norm	Conf(%)	Formula
Minimum:					-1.5				
Maximum:			5.0	10.0	50.0				
	676.1710	676.1708	0.2	0.3	12.5	627.5	n/a	n/a	C29 H36 N3 O10 Na S Cl

PV1

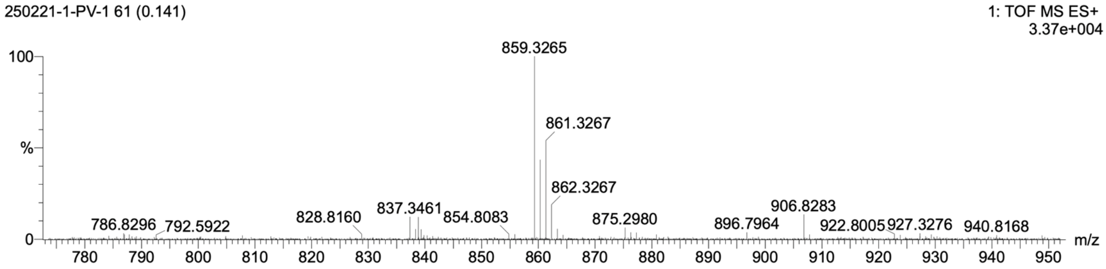

Minimum:				-1.5				
Maximum:		5.0	5.0	50.0				
Mass	Calc. Mass	mDa	PPM	DBE	i-FIT	Norm	Conf(%)	Formula
859.3265	859.3266	-0.1	-0.1	13.5	277.1	n/a	n/a	C39 H57 N6 O8 S2 Cl Na

PV2

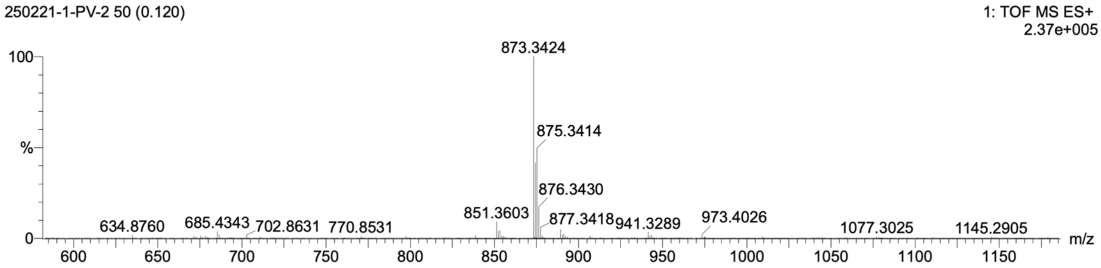

Minimum:				-1.5				
Maximum:		5.0	5.0	50.0				
Mass	Calc. Mass	mDa	PPM	DBE	i-FIT	Norm	Conf(%)	Formula
873.3424	873.3422	0.2	0.2	13.5	431.7	n/a	n/a	C40 H59 N6 O8 Na S2 Cl

PV3

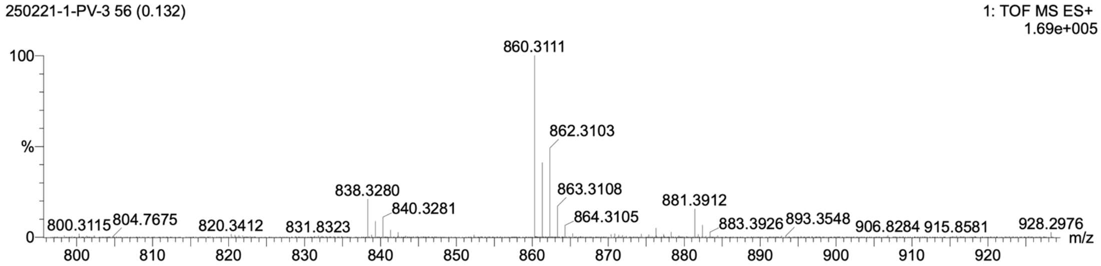

Minimum:				-1.5				
Maximum:		5.0	5.0	50.0				
Mass	Calc. Mass	mDa	PPM	DBE	i-FIT	Norm	Conf(%)	Formula
860.3111	860.3106	0.5	0.6	13.5	430.4	n/a	n/a	C39 H56 N5 O9 Na S2 Cl

PV4

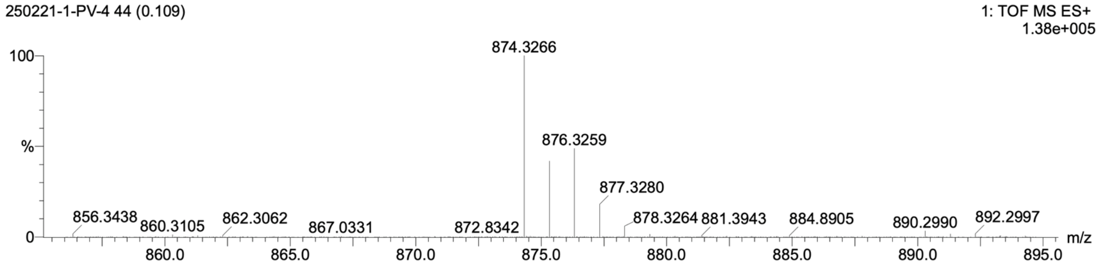

Minimum:				-1.5				
Maximum:		5.0	5.0	50.0				
Mass	Calc. Mass	mDa	PPM	DBE	i-FIT	Norm	Conf(%)	Formula
874.3266	874.3262	0.4	0.5	13.5	399.9	n/a	n/a	C40 H58 N5 O9 Na S2 Cl

PV5

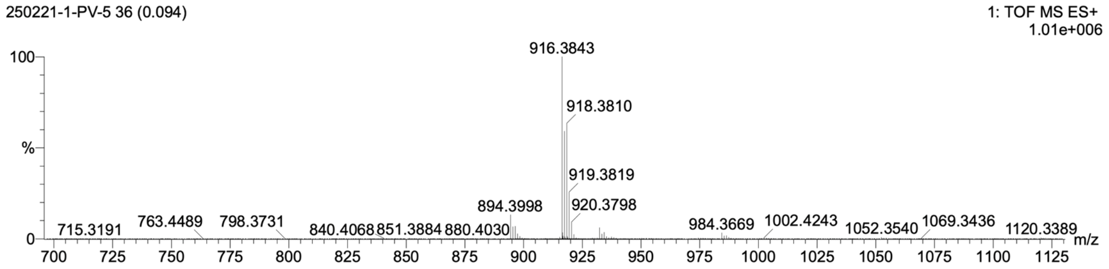

Minimum:				-1.5				
Maximum:		5.0	5.0	50.0				

Mass	Calc. Mass	mDa	PPM	DBE	i-FIT	Norm	Conf(%)	Formula
916.3843	916.3844	-0.1	-0.1	13.5	555.2	n/a	n/a	C42 H64 N7 O8 Na S2 Cl

PV6

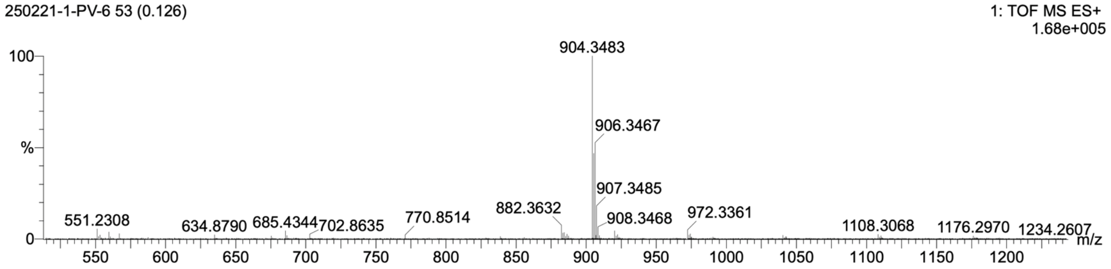

Minimum:				-1.5				
Maximum:		5.0	5.0	50.0				

Mass	Calc. Mass	mDa	PPM	DBE	i-FIT	Norm	Conf(%)	Formula
904.3483	904.3480	0.3	0.3	13.5	382.8	n/a	n/a	C40 H60 N7 O9 Na S2 Cl

PV7

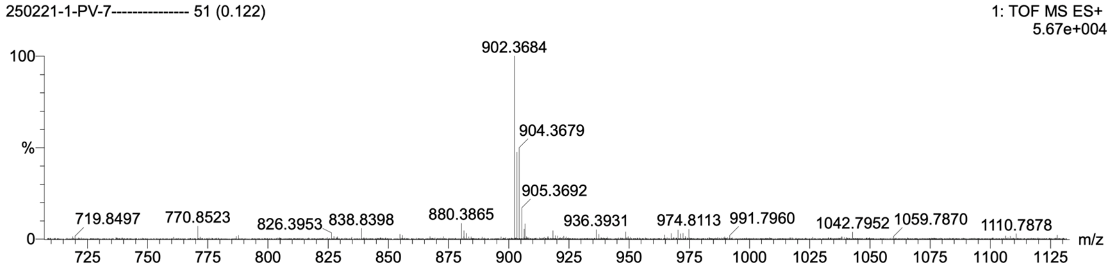

Minimum:				-1.5				
Maximum:		5.0	5.0	50.0				

Mass	Calc. Mass	mDa	PPM	DBE	i-FIT	Norm	Conf(%)	Formula
902.3684	902.3688	-0.4	-0.4	13.5	295.7	n/a	n/a	C41 H62 N7 O8 Na S2 Cl

PV8

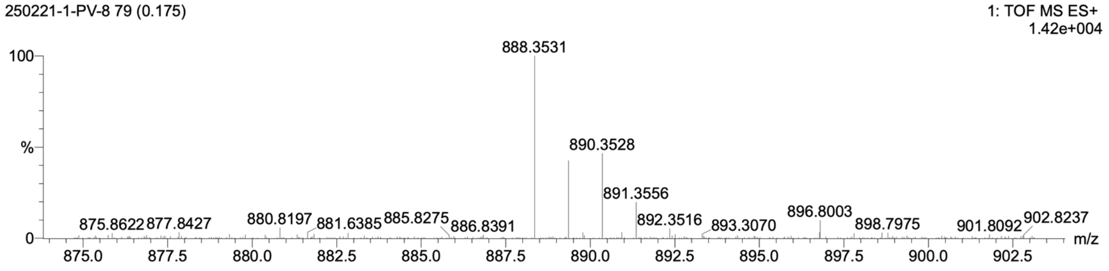

Minimum:				-1.5				
Maximum:		5.0	5.0	50.0				

Mass	Calc. Mass	mDa	PPM	DBE	i-FIT	Norm	Conf(%)	Formula
888.3531	888.3531	0.0	0.0	13.5	207.1	n/a	n/a	C40 H60 N7 O8 Na S2 Cl

PV9

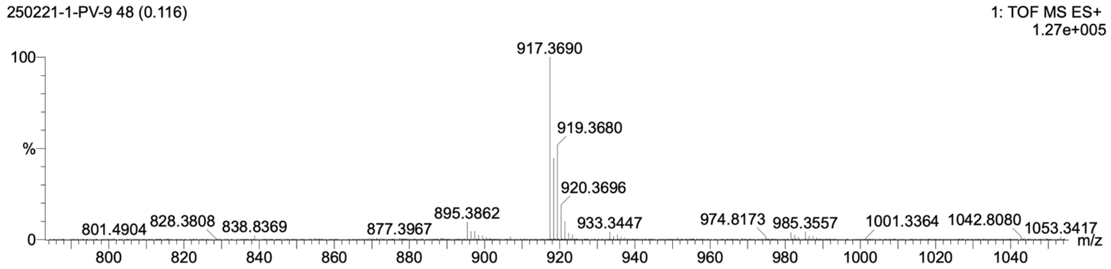

Minimum:				-1.5				
Maximum:		5.0	5.0	50.0				

Mass	Calc. Mass	mDa	PPM	DBE	i-FIT	Norm	Conf(%)	Formula
917.3690	917.3684	0.6	0.7	13.5	357.2	n/a	n/a	C42 H63 N6 O9 Na S2 Cl

PV10

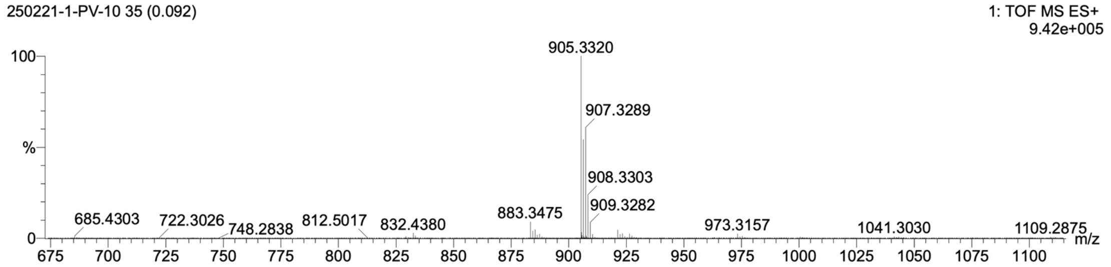

Minimum:				-1.5				
Maximum:		5.0	5.0	50.0				

Mass	Calc. Mass	mDa	PPM	DBE	i-FIT	Norm	Conf(%)	Formula
905.3320	905.3320	0.0	0.0	13.5	545.7	n/a	n/a	C40 H59 N6 O10 Na S2 Cl

PV11

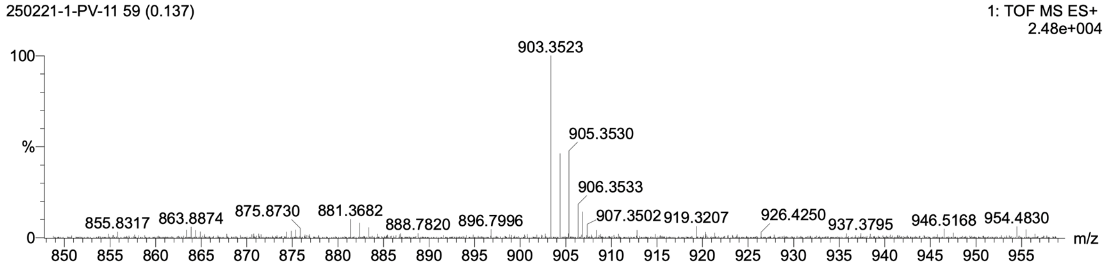

Minimum:				-1.5				
Maximum:		5.0	5.0	50.0				

Mass	Calc. Mass	mDa	PPM	DBE	i-FIT	Norm	Conf(%)	Formula
903.3523	903.3528	-0.5	-0.6	13.5	286.4	n/a	n/a	C41 H61 N6 O9 Na S2 Cl

PV12

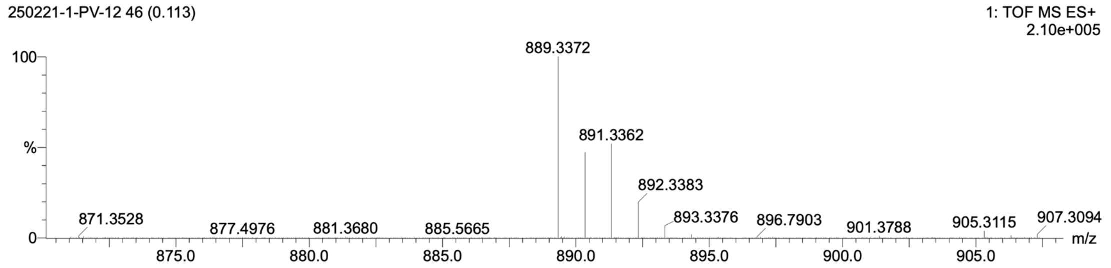

Minimum:				-1.5				
Maximum:		5.0	5.0	50.0				

Mass	Calc. Mass	mDa	PPM	DBE	i-FIT	Norm	Conf(%)	Formula
889.3372	889.3371	0.1	0.1	13.5	442.4	n/a	n/a	C40 H59 N6 O9 Na S2 Cl

PV13

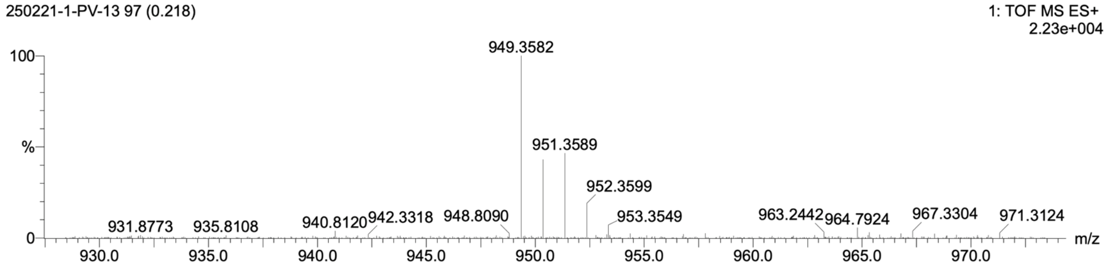

Minimum:				-1.5	
Maximum:		5.0	5.0	50.0	

Mass	Calc. Mass	mDa	PPM	DBE	i-FIT	Norm	Conf(%)	Formula
949.3582	949.3582	0.0	0.0	13.5	286.3	n/a	n/a	C42 H63 N6 O11 Na S2 Cl

PV14

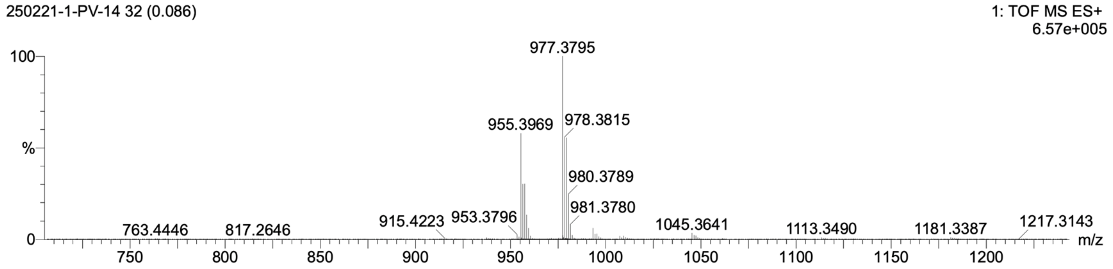

Minimum:				-1.5	
Maximum:		5.0	5.0	50.0	

Mass	Calc. Mass	mDa	PPM	DBE	i-FIT	Norm	Conf(%)	Formula
977.3795	977.3796	-0.1	-0.1	18.5	506.6	n/a	n/a	C46 H63 N8 O8 Na S2 Cl

PV15

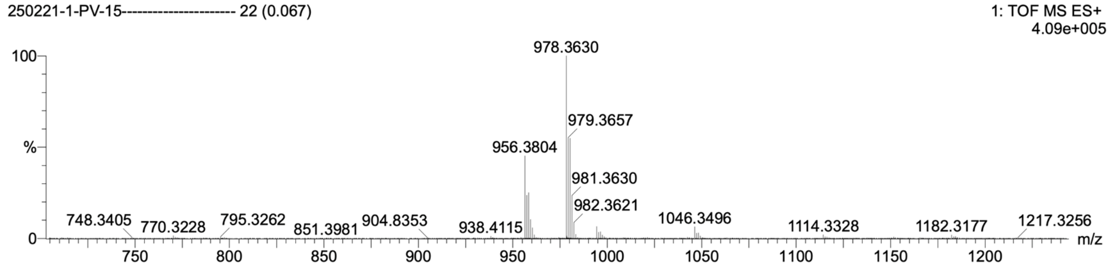

Minimum:				-1.5	
Maximum:		5.0	5.0	50.0	

Mass	Calc. Mass	mDa	PPM	DBE	i-FIT	Norm	Conf(%)	Formula
978.3630	978.3637	-0.7	-0.7	18.5	450.3	n/a	n/a	C46 H62 N7 O9 Na S2 Cl

PV16

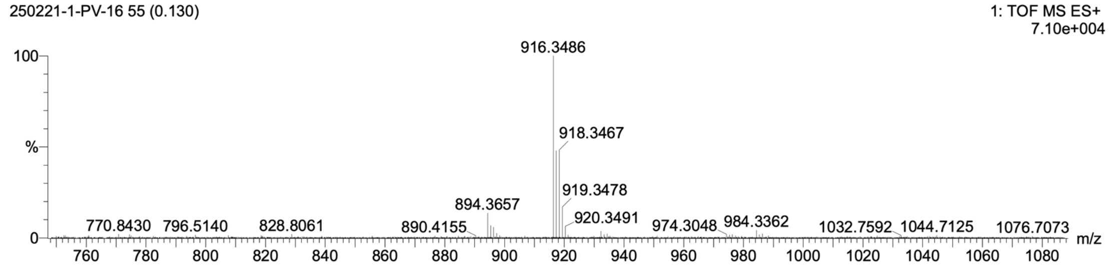

Minimum:				-1.5	
Maximum:		5.0	5.0	50.0	

Mass	Calc. Mass	mDa	PPM	DBE	i-FIT	Norm	Conf(%)	Formula
916.3486	916.3480	0.6	0.7	14.5	429.7	n/a	n/a	C41 H60 N7 O9 Na S2 Cl

PV17

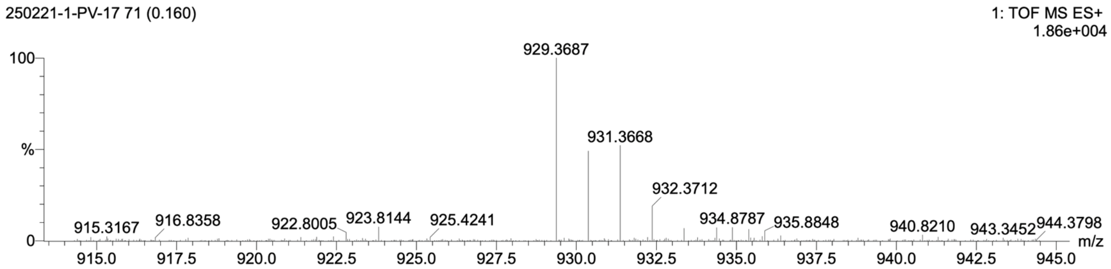

Minimum:				-1.5
Maximum:		5.0	5.0	50.0

Mass	Calc. Mass	mDa	PPM	DBE	i-FIT	Norm	Conf(%)	Formula
929.3687	929.3684	0.3	0.3	14.5	294.6	n/a	n/a	C43 H63 N6 O9 Na S2 Cl

PV18

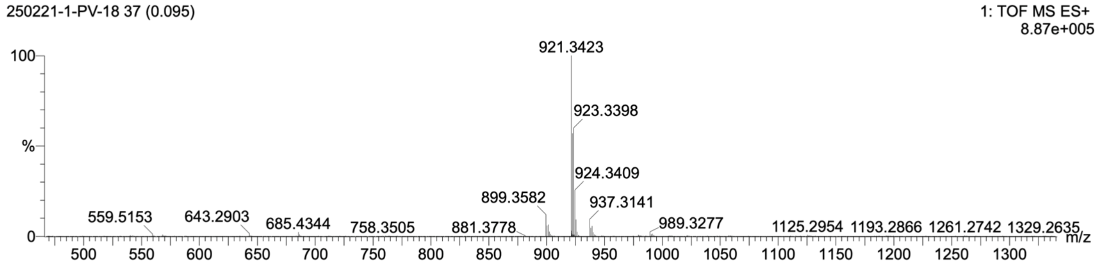

Minimum:				-1.5
Maximum:		5.0	5.0	50.0

Mass	Calc. Mass	mDa	PPM	DBE	i-FIT	Norm	Conf(%)	Formula
921.3423	921.3422	0.1	0.1	17.5	508.7	n/a	n/a	C44 H59 N6 O8 Na S2 Cl

PV19

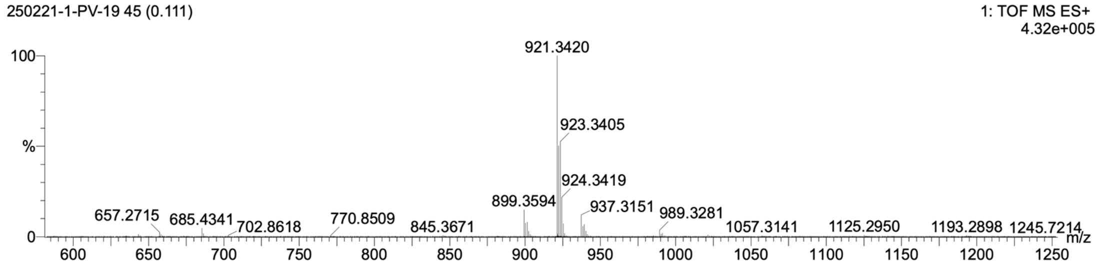

Minimum:				-1.5
Maximum:		5.0	5.0	50.0

Mass	Calc. Mass	mDa	PPM	DBE	i-FIT	Norm	Conf(%)	Formula
921.3420	921.3422	-0.2	-0.2	17.5	481.3	n/a	n/a	C44 H59 N6 O8 Na S2 Cl

PV20

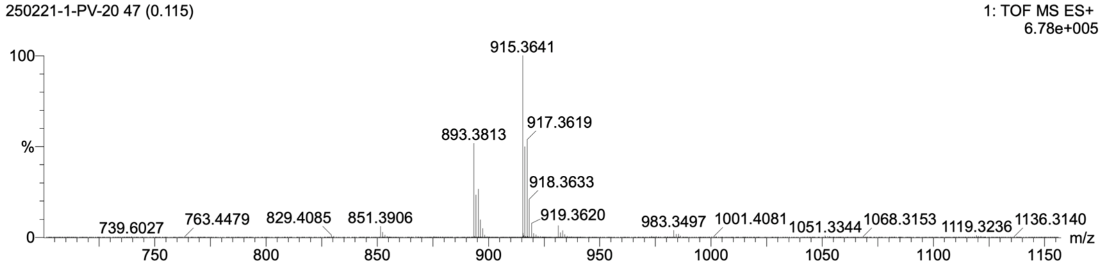

Minimum:				-1.5
Maximum:		5.0	5.0	50.0

Mass	Calc. Mass	mDa	PPM	DBE	i-FIT	Norm	Conf(%)	Formula
915.3641	915.3640	0.1	0.1	14.5	485.2	n/a	n/a	C41 H61 N8 O8 Na S2 Cl

PV21

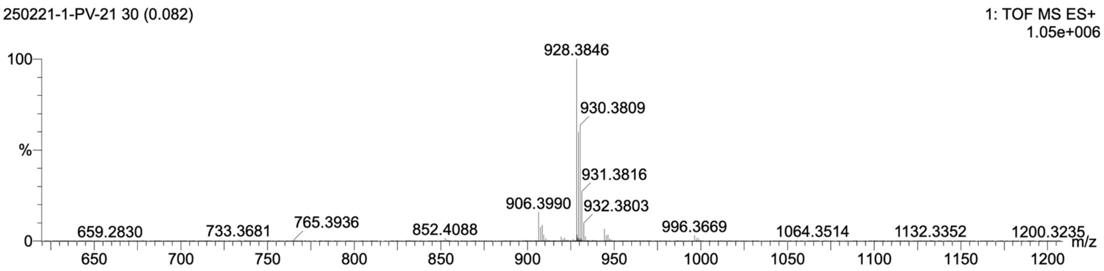

Minimum:				-1.5				
Maximum:		5.0	5.0	50.0				

Mass	Calc. Mass	mDa	PPM	DBE	i-FIT	Norm	Conf(%)	Formula
928.3846	928.3844	0.2	0.2	14.5	505.8	n/a	n/a	C43 H64 N7 O8 Na S2 Cl
